# Multigene phylogeny and cell evolution of chromist infrakingdom Rhizaria: contrasting cell organisation of sister phyla Cercozoa and Retaria

**DOI:** 10.1007/s00709-018-1241-1

**Published:** 2018-04-17

**Authors:** Thomas Cavalier-Smith, Ema E. Chao, Rhodri Lewis

**Affiliations:** Department of Zoology, University of Oxford, South Parks Road, Oxford, OX1 3PS UK

**Keywords:** Cell evolution, Chromista, Harosa, Rhizarian phylogeny, Cercozoa, Retaria

## Abstract

Infrakingdom Rhizaria is one of four major subgroups with distinct cell body plans that comprise eukaryotic kingdom Chromista. Unlike other chromists, Rhizaria are mostly heterotrophic flagellates, amoebae or amoeboflagellates, commonly with reticulose (net-like) or filose (thread-like) feeding pseudopodia; uniquely for eukaryotes, cilia have proximal ciliary transition-zone hub-lattices. They comprise predominantly flagellate phylum Cercozoa and reticulopodial phylum Retaria, whose exact phylogenetic relationship has been uncertain. Given even less clear relationships amongst cercozoan classes, we sequenced partial transcriptomes of seven Cercozoa representing five classes and endomyxan retarian *Filoreta marina* to establish 187-gene multiprotein phylogenies. Ectoreta (retarian infraphyla Foraminifera, Radiozoa) branch within classical Cercozoa as sister to reticulose Endomyxa. This supports recent transfer of subphylum Endomyxa from Cercozoa to Retaria alongside subphylum Ectoreta which embraces classical retarians where capsules or tests subdivide cells into organelle-containing endoplasm and anastomosing pseudopodial net-like ectoplasm. Cercozoa are more homogeneously filose, often with filose pseudopodia and/or posterior ciliary gliding motility: zooflagellate *Helkesimastix* and amoeboid *Guttulinopsis* form a strongly supported clade, order Helkesida. Cercomonads are polyphyletic (Cercomonadida sister to glissomonads; Paracercomonadida deeper). Thecofilosea are a clade, whereas Imbricatea may not be; Sarcomonadea may be paraphyletic. Helkesea and Metromonadea are successively deeper outgroups within cercozoan subphylum Monadofilosa; subphylum Reticulofilosa (paraphyletic on site-heterogeneous trees) branches earliest, Granofilosea before Chlorarachnea. Our multiprotein trees confirm that Rhizaria are sisters of infrakingdom Halvaria (Alveolata, Heterokonta) within chromist subkingdom Harosa (= SAR); they further support holophyly of chromist subkingdom Hacrobia, and are consistent with holophyly of Chromista as sister of kingdom Plantae. Site-heterogeneous rDNA trees group *Kraken* with environmental DNA clade ‘eSarcomonad’, not Paracercomonadida. Ectoretan fossil dates evidence ultrarapid episodic stem sequence evolution. We discuss early rhizarian cell evolution and multigene tree coevolutionary patterns, gene-paralogue evidence for chromist monophyly, and integrate this with fossil evidence for the age of Rhizaria and eukaryote cells, and revise rhizarian classification.

**Electronic supplementary material:**

The online version of this article (10.1007/s00709-018-1241-1) contains supplementary material, which is available to authorized users.

## Introduction

Eukaryotes are classified in five kingdoms: unicellular, largely phagotrophic Protozoa are ancestors of four biologically very distinct clades—osmotrophic Fungi, epithelial phagotrophic Animalia, photosynthetic Plantae and Chromista (Cavalier-Smith 1998a, 2018; Ruggiero et al. 2015). Animalia and Fungi are all heterotrophs. Almost all Plantae are photosynthetic. Their arguably sister kingdom Chromista includes all chromophyte algae (e.g. diatoms, brown algae, dinoflagellates, haptophytes), as well as some major heterotrophic protist groups, notably ciliates, sporozoa, heterotrophic heterokonts, heliozoans, and the largely non-photosynthetic infrakingdom Rhizaria, the subject of this paper. The predatory lifestyle of Rhizaria and details of motility, cell ultrastructure, and body form differ radically from those familiar to cell biologists focusing on animal, fungal or plant cells.

Rhizaria are characterised by (1) having an ultrastructurally unique ciliary transition zone [Cavalier-Smith and Karpov 2012b; earlier Cavalier-Smith et al. (2008, 2009) incorrectly thought the transitional proximal hub-lattice and distal hub-spoke structures were restricted to classical Cercozoa] and (2) the widespread presence of reticulose (net-like) or filose (thread-like) feeding pseudopodia (Bass et al. 2009a). It is argued that the ciliary transition zone hub-lattice, hub-spoke structure, and reticulose pseudopodia are three rhizarian synapomorphies (Cavalier-Smith and Karpov 2012b), though it is sometimes wrongly said they lack well-defined synapomorphies (e.g. Krabberød et al. 2017). Distinctiveness of rhizarian cell biology is emphasised by novel features of actin, myosin and tubulin evolution with unique protein paralogues absent in other eukaryotes (Krabberød et al. 2017).

As delimited by Cavalier-Smith (2003b), Rhizaria comprise phyla Cercozoa (Cavalier-Smith 1998a, b) and Retaria (Foraminifera plus Radiozoa: Cavalier-Smith 1999). A major re-evaluation of kingdom Chromista adjusted the boundary between Cercozoa and Retaria by transferring former cercozoan subphylum Endomyxa to Retaria and establishing a new retarian subphylum Ectoreta to embrace Radiozoa and Foraminifera (Cavalier-Smith 2018); in doing so, Cercozoa and Retaria became sister clades with contrasting cellular phenotypes. That transfer was made for three reasons: (1) it emphasised a primary divergence between often filose amoeboflagellates (Cercozoa) and non-flagellates with reticulose pseudopodia (Retaria); (2) 187-protein trees (Cavalier-Smith et al. 2015a) strongly showed Endomyxa plus Ectoreta as a clade (revised Retaria); and (3) it made Cercozoa phenotypically more uniform (ancestrally flagellates that move by ciliary gliding on surfaces rather than swimming, a property never seen in Retaria). More recent 255-protein trees also robustly show Endomyxa, Ectoreta, revised Retaria and revised Cercozoa as clades (Krabberød et al. 2017), and thus fully support the transfer of Endomyxa to Retaria and formal restriction of Cercozoa to what used to be called ‘core Cercozoa’ (Nikolaev et al. 2004; Pawlowski 2008) or Filosa (Cavalier-Smith 2003b, 2018). Many earlier sequence trees now appear to have been misleading with respect to relationships of Endomyxa, Cercozoa and Ectoreta owing to insufficient gene and taxon sampling. Thus some had placed either Foraminifera alone or Ectoreta as a whole within Endomyxa (Burki et al. 2010, 2013; Sierra et al. 2013, 36 gene analysis; Roy et al. 2014, 27 protein analysis), and other single- or multigene trees had shown Ectoreta and classical Cercozoa (i.e. including Endomyxa) as sister clades (e.g. Cavalier-Smith et al. 2004; Burki et al. 2010; Sierra et al. 2013, 109-gene analysis). However, improved rhizarian gene and taxon sampling for multiprotein trees and use of evolutionarily more realistic site-heterogeneous algorithms (Cavalier-Smith et al. 2015a; Krabberød et al. 2017) yielded a consensus that Endomyxa and revised Retaria and Cercozoa (Cavalier-Smith 2018) are all clades. Multiprotein trees robustly support monophyly of Rhizaria, always a strongly supported clade (Burki et al. 2007, 2008, 2009, 2010, 2012; Cavalier-Smith et al. 2014, 2015a; Krabberød et al. 2017).

Cercozoa are now recognised as ancestrally biciliate heterotrophic flagellates that typically glide on the posterior cilium and have a marked propensity to form filose pseudopodia (filopodia) for help in catching or ingesting prey (e.g. thaumatomonads); several times, some became filose amoebae by losing both cilia [often forming shell-like tests into which they can withdraw pseudopodia for protection, e.g. Euglyphida, *Lecythium, Penardeugenia* (Dumack et al. 2016a, 2017b; Heger et al. 2010; Wylezich et al. 2002)] or more rarely became planktonic flagellates by giving up the benthic gliding lifestyle [e.g. heterotrophic biciliate *Katabia* (Karpov et al. 2003); photosynthetic uniciliate *Bigelowiella*]. Cercozoa now comprise two subphyla (Cavalier-Smith 2018): early diverging Reticulofilosa (classes Chlorarachnea, Granofilosea, Skiomonadea) and later evolving Monadofilosa (classes Metromonadea, Helkesea, Sarcomonadea, Imbricatea, Thecofilosea). Unlike Retaria, reticulopodia are very rare in Cercozoa, apart from the strongly net-like granofilosean *Reticulamoeba* (Bass et al. 2012), recently discovered scaly *Kraken* (Dumack et al. 2016b, 2017a) and atypical net-forming meropodial *Chlorarachnion*. Filopodia predominate in five of the eight cercozoan classes, but are undetected in Metromonadea or Skiomonadea, and pseudopodia in amoeboid guttulinopsid Helkesea are mostly lobose (as they can be rarely in sarcomonads or Thecofilosea). Apart from the strongly supported deepest branching of the skiomonad *Tremula* on 18S rRNA trees (Howe et al. 2011a), the basal branching order of Cercozoa has always been poorly resolved on single-gene trees (Cavalier-Smith and Chao 2003a; Howe et al. 2011a); demarcation between Sarcomonadea and Imbricatea is particularly unclear (Scoble and Cavalier-Smith 2014).

Retaria ancestrally were trophically not flagellates but large amoeboid cells, usually with reticulose pseudopods (reticulopodia) rather than filopodia; they form swimming biciliate or uniciliate stages (never gliding) only transiently for dispersal or sexual reproduction and display a strong tendency to cellular gigantism and evolution of mineral skeletons (Cavalier-Smith 2018). These skeletons, notably in foraminifera (Payne et al. 2013) and polycystine radiolaria (Biard et al. 2015), provide billions of fossils important for palaeontology and economic geology. Two endomyxan lineages became reduced parasites of photosynthetic chromists or plants (class Phytomyxea) or of animals (class Ascetosporea: much more diverse than once thought: Ward et al. 2018), of economic significance respectively for agriculture and fisheries. Except for the planktonic axopodial swimmer *Sticholonche*, reticulose pseudopodia are universal in Ectoreta and were the basis for defining phylum Retaria (Cavalier-Smith 1999), but since then turned out to be widespread also in free-living members of subphylum Endomyxa. Endomyxa were initially limited to the non-reticulose, parasitically simplified Ascetosporea and Phytomyxea (Cavalier-Smith 2002a) but now also include two phylogenetically distinct free-living classes which can be filose or reticulose—marine Gromiidea (*Gromia*, *Filoreta*) and the often soil-dwelling and eukaryovorous vampyrellid amoebae (Ruggiero et al. 2015).

The position of Rhizaria within Chromista has also been debated, as Chromista are a huge mixture of morphologically extremely diverse photosynthetic, phagotrophic and osmotrophic organisms, whose exceptional disparity in gross body form long delayed recognition of their phylogenetic unity (Cavalier-Smith 1981a, 1986, 1989, 2010a, 2013a, 2018; Cavalier-Smith et al. 2015a). Chromista include (1) all organisms with chlorophyll-c containing chloroplasts of secondary red algal origin (i.e. chromophyte algae) that have a fundamentally different membrane topology and chloroplast protein-targeting machinery from Plantae (Cavalier-Smith 2000a, 2003a, 2013a, 2018); (2) all organisms with tubular ciliary hairs; (3) all protists with axopodia; and those with any combination of these characters that clearly distinguish them from the usually heterotrophic ancestral kingdom Protozoa. Cavalier-Smith et al. (2015a) argued that the first two characters arose once in the ancestral chromist, whereas axopodia evolved polyphyletically is several distinct lineages within Rhizaria, Heterokonta (= stramenopiles), and Haptista. A cytoskeletal synapomorphy for all four chromist groups has now been identified that arguably predisposes chromists to evolve axopodia and clarifies the origin of distinctive cercozoan ventral centriolar roots (Cavalier-Smith 2018); evolution of periplastid protein import was reassessed and arguments against monophyly of Chromista refuted (Cavalier-Smith 2018). Some lineages that secondarily lost some or all uniquely chromist characters were historically confused with Protozoa, where many heterotrophic chromists were long wrongly classified. Rhizaria were recognised as a distinct clade (Cavalier-Smith 2002a, 2003b; Nikolaev et al. 2004) decades after kingdom Chromista was established (Cavalier-Smith 1981a, 1986); because no Rhizaria have chlorophyll-c containing chloroplasts or tubular ciliary hairs, the defining chromistan characters, Rhizaria were originally wrongly excluded from kingdom Chromista and left in Protozoa (Cavalier-Smith 1981a). Burki et al. (2007) showed that multigene trees strongly group Rhizaria with Halvaria (Heterokonta and Alveolata) as a major eukaryote clade provisionally labelled SAR. Accordingly, Cavalier-Smith (2010a) formally grouped Rhizaria (as an infrakingdom) with Halvaria as new chromist subkingdom Harosa, which is universally accepted as a robust clade including Rhizaria.

The other chromist subkingdom Hacrobia (comprising Haptista and Cryptista: Okamoto et al. 2009; Cavalier-Smith 2010a) remains somewhat controversial because some multigene trees split Hacrobia, group Haptista and the divergent cryptist *Telonema* with Harosa as a reduced chromist clade, and place other Cryptista with Plantae (Burki et al. 2013, 2016; Krabberød et al. 2017, though this analysis omitted the important hacrobian clade Corbihelia). Some trees show both Hacrobia and Chromista as clades, with Chromista sister to Plantae and Hacrobia sister to Harosa (Burki et al. 2009; Roy et al. 2014); but others showing Hacrobia as a clade group them with Plantae not Harosa (Burki et al. 2012; Cavalier-Smith et al. 2014, 2015b). Thus multigene trees may show Hacrobia and/or Chromista as holophyletic or paraphyletic. Cavalier-Smith et al. (2015a) clarified reasons for these apparent conflicts, some related to the fundamentally chimaeric nature of chromist cells and genomes (Cavalier-Smith 2013a), concluding that Hacrobia and Haptista (haptophytes, centrohelids) are probably both clades and that overall evidence best fits chromist holophyly also. That and refutation of other criticisms of chromist monophyly (Cavalier-Smith 2018) make it unnecessary to exclude Cryptista from Chromista, a possible revision formerly considered in the light of poorly resolved 18S rDNA trees (Cavalier-Smith et al. 1994; Cavalier-Smith 1995) that the 255-protein PhyloBayes (but not ML) tree of Krabberød et al. (2017) might be supposed to favour. A site-heterogeneous analysis using 478 highly conserved genes found maximum support for both Harosa and Hacrobia being monophyletic and for Chromista plus Plantae being a clade (superkingdom Corticata) but showed Hacrobia as sister to Plantae (Ren et al. 2016; contradicting Krabberød et al. 2017), but their study undersampled Hacrobia, omitting Corbihelia studied by Cavalier-Smith et al. (2015a).

Halvaria and Hacrobia each include two phyla with chlorophyll-c containing chloroplasts of red algal origin (Cavalier-Smith et al. 2015a), but Rhizaria are almost all heterotrophic phagotrophs—only cercozoan order Chlorarachnida has true chloroplasts acquired together with a miniaturised secondary nucleus (the nucleomorph) by secondary symbiogenetic enslavement of a green alga (Cavalier-Smith 2013a; Keeling 2013), and the cercozoan testate amoeba *Paulinella* has an enslaved cyanobacterium functioning as a chromatophore unrelated to chloroplasts (Cavalier-Smith 2013a). The first site-homogeneous multigene trees (123 genes, 49 species), though robustly grouping Rhizaria with Halvaria, incorrectly put them as sisters to heterokonts only (Burki et al. 2007). Later, as evolutionarily more realistic site-heterogeneous amino acid substitution models were used, and taxon and gene sampling incrementally improved, Rhizaria more and more strongly were established as sisters of holophyletic Halvaria, not of heterokonts alone (Burki et al. 2008, 2009, 2012, 2016; Cavalier-Smith et al. 2015a), though there can still be conflict between site-homogeneous and site-heterogeneous trees on this point (Krabberød et al. 2017).

Rhizaria are megadiverse: Ruggiero et al. (2015) recognised 17 classes and 63 orders, although that classification erroneously included Pseudosporida and Rotosphaerida and omitted Axomonadida and Mikrocytida (Hartikainen et al. 2014), and Cavalier-Smith (2018) established a new order Helkesida and new class Helkesea for some distinctive flagellates and amoebae, as well as new order Minorisida for the closest heterotrophic relatives of chlorarachnids. Class and ordinal relationships within both rhizarian phyla are mostly uncertain because of contradictory or poorly resolved single-gene trees and serious taxonomic undersampling of multigene trees.

To clarify internal phylogeny of Cercozoa, and to test the monophyly of Cercozoa and Retaria more thoroughly and strengthen the basis for accurately placing Rhizaria within Harosa, we carried out partial transcriptome sequencing for eight Rhizaria: the net-like endomyxan *Filoreta marina* and seven Cercozoa in five non-chlorarachnean classes. Seven of the 8 cercozoan classes (including Imbricatea and Sarcomonadea whose monophyly has been uncertain) are now represented by 14 species as are 5 endomyxan species in our 162-taxon concatenated 187-gene alignment of 50,964 amino acids. For the first time, our multiprotein trees in conjunction with those of Sierra et al. (2016) and Krabberød et al. (2017) establish the relative branching order of all rhizarian classes, but suggest that Imbricatea may be paraphyletic (if scales evolved once) or polyphyletic (if scales arose twice). For comparison, we also show the first site-heterogeneous 18S rDNA trees to include both Helkesida and Ventricleftida as well as the recently described scaly, branching, filose amoeba *Kraken* (Dumack et al. 2016b, 2017a): *Kraken* is apparently related not to Paracercomonadida as earlier supposed but to environmental DNA clade eSarcomonad (Scoble and Cavalier-Smith 2014) and possibly also to imbricate subclass Placonuda. The non-amoeboid gliding cercozoan zooflagellate *Helkesimastix* because of its aberrantly long branch was not credibly placed on rDNA trees (Cavalier-Smith et al. 2009); it groups strongly on our multiprotein trees with the non-ciliate aggregative amoeba *Guttulinopsis* (Brown et al. 2012)—this clade, recently seen by 18S rDNA (Bass et al. 2016), representing the recently established class Helkesea (Cavalier-Smith 2018), does not belong in Sarcomonadea or Thecofilosea.

Our phylogenetic analyses support holophyly of revised phylum Retaria, and both its subphyla Ectoreta and Endomyxa, and revised Cercozoa, and show Reticulofilosa (represented by cercozoan classes Chlorarachnea and Granofilosea) branching robustly earlier than clade Monadofilosa. Within Monadofilosa, class Sarcomonadea is probably ancestral to class Imbricatea. We show that Granofilosea branch more deeply than Chlorarachnea, confirming that Reticulofilosa are ancestral to monadofilosans, and that Monadofilosa are a clade with Metromonadea the earliest branch—as some 18S rDNA trees weakly hinted (Howe et al. 2011a). We discuss cell evolutionary implications of these improved phylogenies and explain how our multigene trees illuminate processes of protein sequence evolution, especially the pervasive but neglected importance of molecular coevolution, and the relative timing of key rhizarian cellular innovations. We list 25 key conclusions at the end of the paper.

## Materials and methods

Details of culturing, RNA extraction and c-DNA library construction are in the electronic supplementary material for the reticulose non-ciliate endomyxan *Filoreta marina* Bass and Cavalier-Smith, 2009 (CCAP 1921/2) and seven separately studied Cercozoa: *Minimassisteria diva* Arndt and Cavalier-Smith in Howe et al., 2011a (CCAP 1947/1); *Micrometopion nutans* Howe et al., 2011a (CCAP 1904/1); *Neocercomonas* (= *Cercomonas*) *clavideferens* Vickerman in Bass et al., 2009 (strain 17-12-D (Bass et al. 2009b), identical in rRNA ITS1 and 2 to type strain ATCC 50319; originally a *Cercomonas* but moved to *Neocercomonas* in Cavalier-Smith and Karpov, 2012b); *Sandona ubiquita* Howe et al. 2009 (strain C19 from Chile, closely related to the type strain W36; Nies-2427; see Howe 2008); *Nudifila producta* Howe and Cavalier-Smith in Howe et al., 2011a (CCAP 1911/1); *Thaumatomonas oxoniensis* Bass and Cavalier-Smith, 2011 in Howe et al., 2011a (CCAP 1903/2); and *Helkesimastix marina* Cavalier-Smith in Cavalier-Smith et al. 2009 (ATCC 50328). *Filoreta* was sequenced by standard Sanger sequencing by Agencourt Bioscience (Beverly MA, USA) and the seven Cercozoa by multiplex 454 pyrosequencing as previously described (Cavalier-Smith et al. 2014). Supplementary Table S1 summarises the total sequences obtained. Assembly and editing of the 187-gene multiple alignment were described previously (Cavalier-Smith et al. 2014, 2015a,b). Table S2 shows how some composite pseudotaxa were made by combining sequences from two species in the same genus to increase gene representation and is a key to labelling included sequences. Table S3 shows gene representation for each sequenced taxon and Table S4 lists the full names for the analysed genes. The *Paulinella chromatophora* Lauterborn, 1895 sequences are from the partial transcriptome of Nakayama and Ishida (2009), who kindly provided them before public availability and agreed to their inclusion.

As *Micrometopion* was fed on the bodonid flagellate *Procryptobia sorokini* (supplied by Mylnikov, presumably the type strain from a Sicilian saline lake: Frolov et al. 2001), the transcriptome for this culture included genes from both species which were unambiguously separated by examining all 187 single-gene trees: of the 2 sequences present for most genes, 1 invariably grouped within bodonids, and was therefore from *Procryptobia* (confirmed by being extremely close but not identical to the sequences for another strain of *Procryptobia sorokini*: Deschamps and Moreira 2011, labelled *Procryptobia sorokini* M in the figures) and the other (*Micrometopion* itself) invariably grouped within Cercozoa and Rhizaria. As explained previously (Cavalier-Smith and Chao 2012a; Cavalier-Smith et al. 2015a; Howe et al. 2011a), the *Minimassisteria* culture was also mixed, with the also axopodial naked heliozoan *Oxnerella micra* being included in a joint transcriptome. Though these two organisms are less evolutionarily divergent than are *Micrometopion* and *Procryptobia*, because they belong to different chromist subkingdoms (haptist *Oxnerella* in Hacrobia and cercozoan *Minimassisteria* in Harosa), we were able to decide which was which using the 187 single-gene trees.

Multigene phylogenetic analyses used 50,964 amino acids and the best available site-heterogeneous amino-acid substitution model (PhyloBayes-MPI v.1b GTR-CAT-Γ 4 rates: Lartillot and Philippe 2004; Lartillot et al. 2013) using two separate chains. To verify that both components of the two mixtures were correctly identified, we ran eukaryote-wide analyses for 158 and 159 eukaryotes but excluded Plantae to avoid possible distortion by unrecognised red algal paralogues in chromists (see Deschamps and Moreira 2009; Cavalier-Smith et al. 2015a). To avoid perturbation of rhizarian branching by distant outgroups and make convergence of PhyloBayes trees easier, we also ran trees restricted to chromists (72 taxa). For these and others restricted to Rhizaria only, we also ran maximum likelihood (ML) trees using the best available amino acid substitution model (LG) by RAxML-MPI v.7.2.8 PROTGAMMALGF (Stamatakis et al. 2005) and 100 fast bootstrap resamplings (Stamatakis et al. 2008), even though this model is site-homogeneous and thus less evolutionarily realistic than GTR-CAT-Γ (henceforth called CAT for brevity) (Lartillot and Philippe 2004). The acantharian *Amphilonche elongata* included by Sierra et al. (2013) but not Burki et al. (2010) was omitted from these trees as relatively few sequences are available and it is closely related to included *Phyllostaurus sicula*, both belonging to acantharian 18S rDNA clade F (Decelle et al. 2012), but was included in trees restricted to rhizarians only.

In addition to transcriptome sequencing, we attempted to partially sequence the genome from a few cells of a uniprotist culture of the thecofilosean amoeba *Rhogostoma minus* whose DNA was amplified using a whole genome amplification protocol (see supplementary material) instead of its transcriptome. The amplified DNA was prepared for and used in multiplex 454 DNA pyrosequencing together with the trancriptomes as Cavalier-Smith et al. (2014) described in detail. Unsurprisingly, we obtained relatively few sequences for our target genes and were only able to include 897 *Rhogostoma* amino acids. We therefore exluded *Rhogostoma* from the main analyses, but included it in separate analyses of 74 and 75 chromist and 33 rhizarian taxa only.

New 454 sequences are available from GenBank under BioProject numbers SRP048653-6, SRP048658, SRP048662 and SRP053325. *Filoreta marina* ESTs are available under numbers JZ972228–JZ976734.

**Fig. 1:**
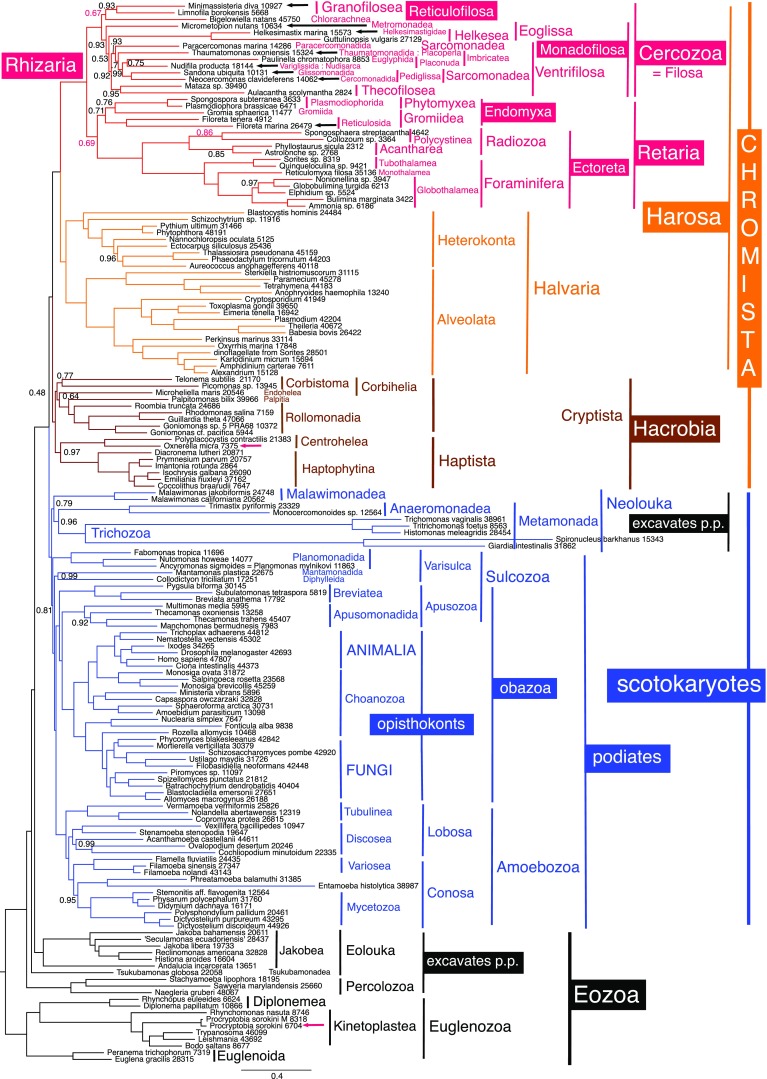
PhyloBayes GTR-CAT-Γ tree of 159 eukaryote-wide taxa, excluding Plantae, using 187 proteins (50,964 amino acid positions). Black arrows show new rhizarian sequences and red arrows the two non-rhizarian sequences from two mixed cultures that we separated phylogenetically. Numbers after species names show how many amino acids were included for each. Most bipartitions had maximal support (1); posterior probabilities are only shown if they did not—in red for the only three for which both chains did not show this topology (max.diff. 1; 5735 trees summed for two chains after removing 1674 as burnin). On all figures, Rhizaria subgroup names reflect the revised Table 1 classification

**Fig. 2:**
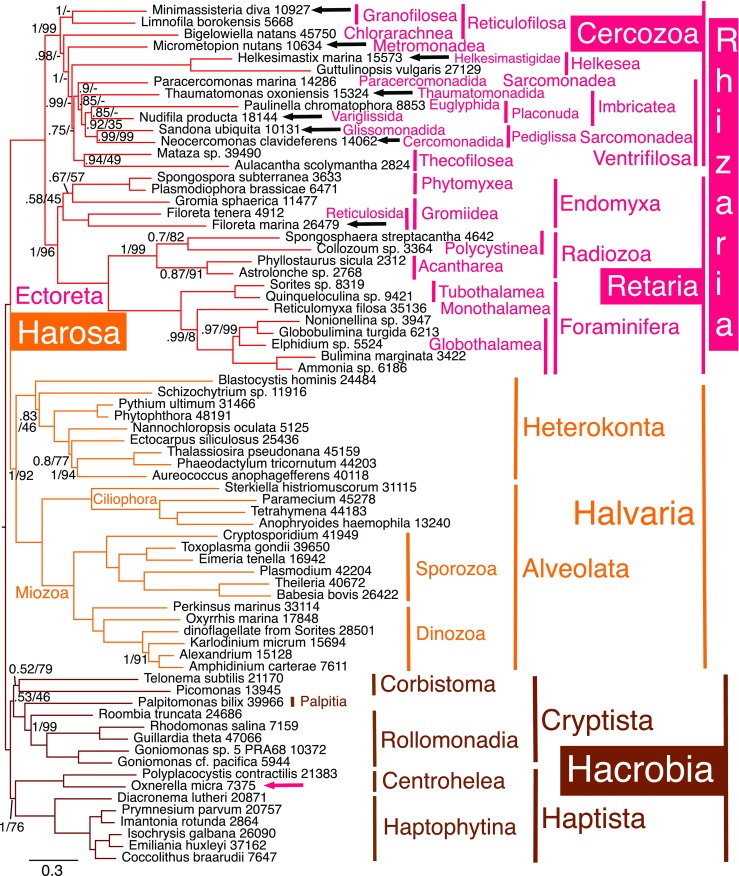
PhyloBayes GTR-CAT-Γ consensus tree of 72 chromists using 187 proteins (50, 964 amino acid positions). Black arrows show new rhizarian sequences; the red arrow highlights *Oxnerella*, whose sequences were phylogenetically separated from *Minimassisteria*. Numbers after species names show how many amino acids were included for each. Most bipartitions had maximal support by both CAT and ML (1/100); support values are only shown for those that did not (posterior probabilities left; ML 100 fast bootstraps right). Dashes indicate bipartitions not found on the corresponding ML tree (Fig. S2). The two chains converged satisfactorily (maxdiff 0.244572; 25,345 trees summed after removing 14,399 as burnin)

We excluded sequences of *Mikrocytos mackini* from alignments because of its excessively long branch on multigene trees (Burki et al. 2013), so attempted to obtain sequences from the haplosporidian, *Minchinia chitonis*, as a transcriptome under that name is publicly available (MMETSP0186; Slamovits in Sierra et al. 2016, collected from a beach in Devon, UK in 2010); we downloaded it from http://camera.calit2.net/mmetsp/details.php?id=MMETSP0186 in the hope of including data for a representative of the parasitic cercozoan class Ascetosporea; blasting this against our alignment revealed 57 potential gene matches. As our quality-control single-gene trees showed that some ‘*Minchinia*’ sequences grouped strongly with diverse protist phyla other than Cercozoa (mostly diatoms or *Micromonas*), we excluded all these and any obviously animal sequences from our alignment as probable contaminants and concatenated the rest as putative ‘*Minchinia*’ genes. Unfortunately, our multigene trees for 74 or 75 chromist taxa showed that this concatenated sequence also did not group with Cercozoa but with the cryptist *Palpitomonas*, implying that these genes were either from a single cryptist contaminant genuinely related to *Palpitomonas* or else a confusing mixture of one or more non-ascetosporean contaminants and genuine *Minchinia* genes that collectively grouped with *Palpitomonas* as an artefactual compromise. Therefore, these supposed ‘*Minchinia*’ sequences were excluded from our main trees (e.g. Figs. 1 and 2) as irrelevant to the position of haplosporidia and potentially distorting were they a mixture not one contaminant. To verify that they grouped with cryptists and not some non-chromist group, we ran 162-taxon eukaryote-wide trees with Plantae represented only by the short-branch glaucophytes.

As a further check against tree distortion by more distant outgroups and of reproducibility amongst taxon samples, we also ran Rhizaria-only trees with (32 taxa) and without *Rhogostoma* (31 taxa), and additionally without *Limnofila* (30 taxa) as Bass et al. (2009a) suggested it might have been contaminated with other protists and potentially misleading. These three alignments also included the third acantharian *Amphilonche elongata*, excluded from our other trees because only 2621 amino acids are available; *Amphilonche* grouped with *Phyllostaurus* with 100% ML and 0.99 CAT support (in agreement with Sierra et al. 2013, 2016) and did not change topology elsewhere in the tree.

We tried to obtain fast bootstrap RAxML trees for 158, 159, and 162 taxa, but calculating the final optimal ML tree exceeded the memory capacity of the 256 processors used: runs terminated without outputting an optimal tree. This is not a serious problem as it is generally accepted that site-heterogeneous trees are evolutionarily more accurate than RAxML LG as they better fit eukaryote multigene data (e.g. Brown et al. 2013; Cavalier-Smith et al. 2014, 2015a, b), so some studies now use only CAT (Derelle et al. 2015).

For comparison with the gene-rich multiprotein trees, we conducted site-heterogeneous 18S rDNA phylogenetic analyses, using PhyloBayes CAT-GTR-Γ with 4 rate classes, for the taxonomically most comprehensive rhizarian alignment to date (464–467 species, a balanced selection of all major lineages of Rhizaria), and for 316/7 species of Monadofilosa only to check the position of *Kraken* and *Cyranomonas* and see whether exclusion of more distant Rhizaria improved tree topology.

## Results

### Separation of mixed protist cultures

The eukaryote-wide 159-taxon tree (Fig. 1) shows that each component of both mixed two-protist cultures (*Minimassisteria/Oxnerella* and *Micrometopion/Procryptobia*) goes to its right phylum, indicating that their sequences were correctly assigned using the single-gene trees. *Minimassisteria* is within Cercozoa with maximal support by CAT and sister to *Limnofila borokensis*, the only other granofilosean, with slightly weaker support (0.93). The separation of *Minimassisteria* from the co-cultured centrohelid heliozoan *Oxnerella micra* (red arrow, Fig. 1) is clear; the latter branches within subphylum Hacrobia as sister to *Polyplacocystis* (the only other centrohelid on the tree) with maximal support, this clade being robustly sister to haptophytes (0.97) in agreement with Cavalier-Smith et al. (2015a). *Minimassisteria* would have to cross five maximally supported nodes (and three less well-supported nodes) to adopt a sister relationship with *Oxnerella*. *Micrometopion* branches within Cercozoa as the most divergent member of Monadofilosa, as 18S rDNA previously suggested (Howe et al. 2011a). By contrast, its food *Procryptobia sorokini* is the maximally supported sister to another culture of *Procryptobia sorokini* (labelled M) isolated by Deschamps et al. (2011) from deep Marmara Sea sediment. The *P*. *sorokini* we used to feed *Micrometopion* (presumably the type strain from a saline lake: Frolov et al. 2001) branched closely (Fig. 1 red arrow) with the deep sea Deschamps et al. strain that is sufficiently different to be an unrecognised sibling species.

### Rhizarian phylogeny

Rhizaria are always maximally supported as a clade and as sister to harosan infrakingdom Halvaria. Taxa are named here in accord with the revised classification in Table 1. Cercozoa and retarian infraphylum Foraminifera are invariably maximally or 99% supported as clades. The 159-taxon consensus tree (Fig. 1) weakly (0.69) shows Endomyxa as sister to Ectoreta (classical Retaria), chain 2 showing this with maximal support; but chain 1 contradicted that by grouping Endomyxa with Cercozoa (0.63), thus excluding Ectoreta from classical Cercozoa. Within Ectoreta, infraphylum Radiozoa is a maximally supported clade in Fig. 1 on both chains, but for a separate 158-taxon tree differing only by omitting *Microheliella* (Supplementary Fig. S1), one chain showed this with maximal support while on the other Radiozoa appear paraphyletic with Polycystinea sister to Foraminifera also with maximal support. Both chains of the 158-taxon tree excluded Ectoreta from Endomyxa with reasonably good support (0.77, 0.99; consensus 0.88), but were contradictory over whether Ectoreta are sisters to Endomyxa or to Cercozoa plus Endomyxa, which together weakly formed a classical Cercozoa clade on the consensus tree (0.56).

**Table 1:** Revised classification of chromist infrakingdom Rhizaria and its 2 phyla, 4 subphyla, 18 classes, and 65 orders

**Phylum 1**. **Cercozoa** Cavalier-Smith, 1998 em. 2018 (8 classes; 32 orders; 80 families) ** Subphylum 1**. **Reticulofilosa**^f^ Cavalier-Smith, 1997 (3 classes; 9 orders; 12 families) ** Class 1**. **Chlorarachnea** Hibberd and Norris, 1984 as Chlorarachniophyceae (2 orders; 4 families) ** Order 1**. **Chlorarachnida** Ishida et al., 1996 (as Chlorarachniales) Family 1. Chlorarachniidae Ishida et al., 1996 (Pyrenoid with shallow slit-like invagination of chloroplast envelope or more broadly by periplastid space with nucleomorph or absent: *Bigelowiella*^a^, *Chlorarachnion*, *Norisiella*, *Partenskyella*, ?*Cryptochlora*) Family 2. Gymnochloridae Cavalier-Smith fam. n**. Diagnosis:** pyrenoid stalked or embedded in plastid; pyrenoid matrix invaded by invaginations of inner plastid envelope membrane only (*Gymnochlora*) or by slit-like invaginations of both envelope membranes—if slit-like, pyrenoid embedded (*Viridiuvalis*) or cell purely amoeboid (*Amorphochlora*). Type genus *Gymnochlora* Ishida et al., 1996 (also *Amorphochlora, Viridiuvalis*) Family 3. Lotharellidae Cavalier-Smith fam. n. **Diagnosis**: Pyrenoid stalked, its matrix divided longitudinally into two halves by chloroplast envelope deep invagination. Type and only genus *Lotharella* Ishida et al., 1996. **Order 2**. **Minorisida** Cavalier-Smith, 2018 Family Minorisidae Cavalier-Smith, 2018 (*Minorisa*) **Class 2**. **Granofilosea** Cavalier-Smith and Bass in Bass et al., 2009 (5 orders; 7 families) **Order 1**. **Cryptofilida** Cavalier-Smith and Bass in Bass et al., 2009 Family 1. Nanofilidae Cavalier-Smith and Bass in Bass et al., 2009 (*Nanofila*) Family 2. Mesofilidae Cavalier-Smith and Bass in Bass et al., 2009 (*Mesofila*) **Order 2**. **Desmothoracida** Hertwig and Lesser, 1874 Family Clathrulinidae Claus, 1874 (e.g. *Clathrulina*, *Hedriocystis*) **Order 3**. **Leucodictyida** Cavalier-Smith, 1993 Family 1. Leucodictyidae Cavalier-Smith, 1993 (*Reticulamoeba, Leucodictyon*) Family 2. Massisteriidae Cavalier-Smith, 1993 (e.g. *Minimassisteria*^a^, *Massisteria*) **Order 4**. **Limnofilida** Cavalier-Smith and Bass in Bass et al., 2009 Family Limnofilidae Cavalier-Smith and Bass, 2009 (*Limnofila*^a^) ** Order 5**. **Axomonadida**^d^ Cavalier-Smith in Yabuki et al., 2012 Family Tetradimorphidae Febvre-Chevalier and Febvre, 1984 (*Tetradimorpha*) **Class 3**. **Skiomonadea** Cavalier-Smith in Cavalier-Smith and Karpov, 2012 (2 orders; currently only 1 family) **Order 1. Tremulida** Cavalier-Smith and Howe in Howe et al., 2011. Sole family Tremulidae Cavalier-Smith fam. n. **Diagnosis** as for *Tremula* Howe et al. (2011a) p. 335 (*Tremula*). **Order 2. 'Aquavolonida' **(Bass et al. 2018) (*Aquavolon*) **Subphylum 2**. **Monadofilosa** Cavalier-Smith, 1997 (5 classes; 23 orders; 68 families) **Superclass 1**. **Eoglissa** Cavalier-Smith in Cavalier-Smith and Oates, 2011 em. (3 orders; 6 families) **Class 1**. **Metromonadea** Cavalier-Smith, 2007 (2 orders; 3 families) **Order 1**. **Metopiida** Cavalier-Smith in Cavalier-Smith and Chao, 2003 Family Metopiidae Cavalier-Smith, 2003 (*Metopion*, *Micrometopion*^a^) **Order 2**. **Metromonadida** Bass and Cavalier-Smith, 2004 Family 1. Metromonadidae Bass and Cavalier-Smith, 2004 (*Metromonas*) Family 2. Kiitoksiidae Cavalier-Smith in Cavalier-Smith and Scoble, 2014 (*Kiitoksia*) **Class 2**. **Helkesea** Cavalier-Smith, 2018 (1 order; 3 families) **Order Helkesida** Cavalier-Smith, 2018 Superfamily 1. Sainouroidea Cavalier-Smith in Cavalier-Smith et al., 2009 em. Family Sainouridae Cavalier-Smith in Cavalier-Smith et al., 2008 (*Sainouron*, *Cholamonas*) Superfamily 2. Helkesimastigoidea Cavalier-Smith superfam. n. Family 1. Helkesimastigidae Cavalier-Smith in Cavalier-Smith et al., 2009 (*Helkesimastix*^a^) Family 2. Guttulinopsidae Olive, 1970 (*Guttulinopsis*^a^, *Rosculus*) **Superclass 2**. **Ventrifilosa** Cavalier-Smith in Cavalier-Smith and Karpov, 2012 em. (3 classes; 20 orders) **Class 1**. **Sarcomonadea** Cavalier-Smith, 1993 stat. n. 1995 em. (3 orders; 11 families) **Subclass 1**. **Paracercomonada** Cavalier-Smith subcl. n. **Diagnosis**: as for suborder Paracercomonadina in Cavalier-Smith and Karpov (2012) p. 57 **Order Paracercomonadida** Cavalier-Smith ord. n. **Diagnosis**: as for suborder Parcercomonadina in Cavalier-Smith and Karpov (2012) p. 57 Family Paracercomonadidae Cavalier-Smith in Cavalier-Smith and Karpov, 2012 (*Paracercomonas*^a^, *Nucleocercomonas*, *Metabolomonas*, *Brevimastigomonas*, *Phytocercomonas*) **Subclass 2**. **Pediglissa** Cavalier-Smith subcl. n. (2 orders; 10 families) **Order 1**. **Cercomonadida** Poche, 1913 em. Cavalier-Smith Family 1. Cavernomonadidae Cavalier-Smith in Cavalier-Smith and Karpov, 2012 (*Cavernomonas*) Family 2. Cercomonadidae Saville Kent, 1880/1 em. Cavalier-Smith (*Cercomonas*, *Eocercomonas*, *Filomonas*, *Neocercomonas*^a^) **Order 2**. **Glissomonadida** Howe et al., 2009 (3 suborders, 8 families) Suborder 1. Allapsina Cavalier-Smith subord. n. Family 1. Allapsidae Howe et al., 2009 (*Allapsa*, *Allantion*, *Teretomonas*) Suborder 2. Sandonina Cavalier-Smith subord. n. Family 1. Bodomorphidae Hollande, 1952 (*Bodomorpha*) Family 2. Sandonidae Howe et al., 2009 (*Neoheteromita, Sandona*^a^, *Flectomonas*, *Mollimonas*) Family 3. Proleptomonadidae Howe et al., 2009 (*Proleptomonas*) Suborder 3. Pansomonadina Vickerman in Vickerman et al., 2005 stat. n. Cavalier-Smith Family 1. Viridiraptoridae Hess and Melkonian, 2013 (*Viridiraptor*, *Orciraptor*) Family 2. Agitatidae Cavalier-Smith and Bass in Bass et al., 2009 (*Agitata*) Family 3. Acinetactidae Stokes, 1886 (*Acinetactis*) Family 4. Aurigamonadidae Cavalier-Smith in Cavalier-Smith and Oates, 2011 (*Aurigamonas*) **Class 2**. **Imbricatea** Cavalier-Smith in Cavalier-Smith and Chao, 2003 em. (10 orders 23 families) **Subclass 1**. **Placonuda** Cavalier-Smith in Cavalier-Smith in Cavalier-Smith and Chao, 2012 (5 orders 14 families) **Superorder 1**. **Nudisarca** Cavalier-Smith in Cavalier-Smith and Chao, 2012 **Order 1**. **Variglissida** Cavalier-Smith in Scoble and Cavalier-Smith, 2014 Family 1. Clautriaviidae Cavalier-Smith in Cavalier-Smith and Scoble, 2013 (*Clautriavia*) Family 2. Nudifilidae Cavalier-Smith in Howe et al., 2011 (*Nudifila*^a^) Family 3. Quadriciliidae Cavalier-Smith fam. n. **Diagnosis**: non-gliding heterotrophs with four cilia, plastic body, and thin, branching, non-granular pseudopodia; cytostome and obvious ventral groove absent. Type genus *Quadricilia* Vørs **(**1992 p. 86). **Order 2**. **Marimonadida** Cavalier-Smith and Bass in Howe et al. 2011 Family 1. Auranticordidae Cavalier-Smith fam. n. **Diagnosis**: 2 or 4 posterior gliding cilia in ventral groove; no anterior cilium. Type genus *Auranticordis* Chantangsi et al. (2008); also *Rhabdamoeba*. Family 2. Cyranomonadidae Cavalier-Smith fam. n. **Diagnosis**: Discoid non-amoeboid gliding biciliates; short anterior cilium, single, long posterior gliding cilium; anterior ciliary depression, not pit or groove. Type genus *Cyranomonas* Lee (2002). Family 3. Pseudopirsoniidae Cavalier-Smith fam. n. **Diagnosis**: anisokont biciliate parasitoids of diatoms, with trophosome; shorter anterior cilium; apicoventral ciliary depression. Type genus *Pseudopirsonia* Kühn et al. (2004). Family 4. Abolliferidae Cavalier-Smith fam. n. **Diagnosis**: biciliates with two parallel anterior-pointing centrioles; two cilia (anterior short, posterior long, recurving and gliding) emerge from deep apical pit bordered ventrally by anterior-projecting cowl-like ventral rim supported by broad microtubule band underlain by massive plate of dense fibrous material; ventral/posterior lobose pseudopodia (Shiratori et al. 2014). Type genus *Abollifer* Vørs (1992). Also includes *Cowlomonas* (Cavalier-Smith and Scoble 2014); probably also *Heterochromonas* Skuja, 1948 with about 13 species (Lee and Patterson 2000). **Superorder 2**. **Euglyphia** Cavalier-Smith superord. n. **Diagnosis**: cell body covered in large imbricate single-tier scales; apical aperture through which protrude either filose pseudopodia or two unequal non-gliding cilia (not both). **Order 1**. **Euglyphida** Copeland, 1956 em. Cavalier-Smith, 1987 Family 1. Euglyphidae Wallich, 1864 (*Euglypha*) Family 2. Trinematidae Hoogenraad and De Groot, 1940 (*Corythion*, *Trinema*) Family 3. Sphenoderiidae Chatelain et al., 2013 (*Sphenoderia*, *Trachelocorythion*) Family 4. Assulinidae Lara et al., 2006 (*Assulina*, *Placocista*) Family 5. Cyphoderiidae De Saedeleer, 1934 (*Cyphoderia*, *Corothionella*, *Pseudocorythion*) Family 6. Paulinellidae De Saedeleer, 1934 (*Paulinella*^a^, *Ovulinata*, *Micropyxidiella*) **Order 2**. **Zoelucasida**^d^ Cavalier-Smith in Scoble and Cavalier-Smith, 2014 Family Zoelucasidae Cavalier-Smith in Scoble and Cavalier-Smith, 2014 (*Zoelucasa*) **Superorder 3**. **Discomonada** Diagnosis as for sole o**rder Discomonadida**^c^ Cavalier-Smith in Scoble and Cavalier-Smith (2014 p. 302). Family Discomonadidae^b^ Cavalier-Smith in Scoble and Cavalier-Smith, 2014 (*Discomonas*) **Subclass 2**. **Placoperla** Cavalier-Smith in Cavalier-Smith and Chao, 2012 (4 orders 7 families) **Superorder 1**. **Placofila** Cavalier-Smith in Cavalier-Smith and Chao, 2012 **Order 1**. **Thaumatomonadida** Shirkina, 1987 Family 1. Thaumatomonadidae Hollande, 1952 (*Allas*, *Ovaloplaca*, *Reckertia*, *Scutellomonas*, *Thaumatomastix*, *Thaumatomonas*^a^, *Thaumatospina*, *Penardeugenia*) Family 2. Peregriniidae Cavalier-Smith in Howe et al., 2011 (*Peregrinia, Gyromitus*) Family 3. Esquamulidae Shiratori et al., 2012 (*Esquamula*) **Order 2**. **Discocelida** Cavalier-Smith, 1997 Family Discoceliidae Cavalier-Smith, 1993 orthog. em. 2012 (*Discocelia*) **Superorder 2**. **Perlatia** Cavalier-Smith in Cavalier-Smith and Chao, 2012 **Order 1**. **Spongomonadida** Hibberd, 1983 Family Spongomonadidae Karpov, 1990 (*Spongomonas*, *Rhipidodendron*) **Order 2**. **Perlofilida**^d^ Cavalier-Smith in Cavalier-Smith and Chao, 2012 Family 1. Pompholyxophryidae Page, 1987 (*Pompholyxophrys*) Family 2. Acanthoperlidae Cavalier-Smith in Cavalier-Smith and Chao, 2012 (*Acanthoperla*) **Subclass 3**. **Krakenia** Cavalier-Smith subcl. n. **Diagnosis**: The clade comprising *Kraken*, scale-bearing non-flagellates with anastomosing filopodia many times longer than cell body diameter, and environmental DNA clade eSarcomonad of unknown morphology. **Order Krakenida** Dumack et al., 2017 ex Cavalier-Smith ord. n. **Diagnosis**: as for family Krakenidae Dumack et al. (2017 p. 370) Family Krakenidae Dumack et al., 2017 (*Kraken*) **Class 3**. **Thecofilosea** Cavalier-Smith in Cavalier-Smith and Chao, 2003 em. 2012 (7 orders; 26 families) **Subclass 1**. **Ventricleftia** Cavalier-Smith subcl. n. **Diagnosis** as for sole order Ventricleftida: **Order Ventricleftida** Cavalier-Smith in Howe et al. (2011 p. 345) Family 1. Ventrifissuridae^b^ Cavalier-Smith fam. n. **Diagnosis** as for type genus *Ventrifissura* Chantangsi and Leander (2010 p. 170). Family 2. Verrucomonadidae^b^ Cavalier-Smith fam. n. **Diagnosis** as for type genus *Verrucomonas* Chantangsi and Leander (2010 p. 170). **Subclass 2**. **Eothecia** Cavalier-Smith in Cavalier-Smith and Chao, 2012 (3 orders) **Order 1**. **Matazida** Cavalier-Smith in Cavalier-Smith and Chao, 2012 Family Matazidae Cavalier-Smith in Cavalier-Smith and Chao, 2012 (*Mataza*^a^) **Order 2**. **Ebriida** Deflandre, 1936 4 families (*Ebria*, *Hermesinum*, *Botuliforma*) **Order 3**. **Cryomonadida** Cavalier-Smith, 1993 (3 families) Family 1. Cryomonadidae Cavalier-Smith, 1993 (*Cryothecomonas*) Family 2. Protaspidae Cavalier-Smith, 1993 (*Protaspa*) Family 3. Rhogostomidae Dumack et al., 2017 (*Rhogostoma*^a^, *Capsellina*, *Sacciforma*) **Subclass 3**. **Phaeodaria** Haeckel, 1879 (2 orders; 16 families) **Order 1**. **Eodarida** Cavalier-Smith in Cavalier-Smith and Chao, 2012 5 families (e.g. *Aulacantha*^a^) **Order 2**. **Opaloconchida** Cavalier-Smith in Cavalier-Smith and Chao, 2012 11 families (e.g. *Challengeron*, *Protocystis*) **Subclass 4**. **Tectosia** Cavalier-Smith in Cavalier-Smith and Chao, 2012 (6 families) **Order Tectofilosida**^g^ Cavalier-Smith in Cavalier-Smith and Chao, 2003 Family 1. Chlamydophryidae De Saedeleer, 1934 (e.g. *Chlamydophrys*^e^, *Diaphoropodon*, *Lecythium*, *Trachyrhizium*) Family 2. Psammonobiotidae Golemansky, 1974 (e.g. *Micropsamella*) Family 3. Volutellidae Sudzuki, 1979 (*Volutella*) Suborder Fiscullina subord. n. Cavalier-Smith **Diagnosis**: filose testate amoebae with organic non-scaly tests that form a clade including *Fisculla* and *Rhizaspis* on ribosomal DNA sequence trees, but excluding *Rhogostoma.* 3 families: Family 1. Fiscullidae Dumack, Mausbach and Bonkowski in Dumack et al., 2017 (*Fisculla*) Family 2. Pseudodifflugiidae De Saedeleer, 1934 (e.g. *Pseudodifflugia, Lithocolla*) Family 3. Rhizaspididae Skuja, 1948 (*Rhizaspis*)
**Phylum 2**. **Retaria** Cavalier-Smith, 1999 em. 2018 (10 classes 33 orders) **Subphylum 1**. **Endomyxa** Cavalier-Smith, 2002 (4 classes 10 orders) **Superclass 1**. **Marimyxia** Cavalier-Smith, 2018 **Class 1**. **Gromiidea**^f^ Cavalier-Smith in Cavalier-Smith and Chao, 2003 em. **Order 1. Gromiida**^a^ Claparède and Lachmann, 1856 (*Gromia*) **Order 2. Reticulosida**^a^ Cavalier-Smith in Cavalier-Smith and Chao, 2003 em. (*Filoreta*) **Class 2**. **Ascetosporea** Sprague 1979 stat. n. Cavalier-Smith, 2002 **Order 1**. **Claustrosporida**^d^ Cavalier-Smith in Cavalier-Smith and Chao, 2003 (*Claustrosporidium*) **Order 2**. **Haplosporida** Caullery and Mesnil, 1889 orth. em. Lühe, 1900 (*Bonamia*, *Haplosporidium*, *Minchinia*, *Urosporidium*) **Order 3**. **Mikrocytida** Hartikainen et al. (2014; diagnosis in electronic supplement cladistic not descriptive). Sole family Mikrocytidae Cavalier-Smith fam. n. Type genus *Mikrocytos* Farley et al. (1988 p. 589). **Diagnosis**: tiny, intracellular unicellular and plasmodial parasites of marine invertebrates, with dense haplosporosome-like inclusions; mitochondria reduced to acristate mitosome, unlike Haplosporida; spores or Golgi stacks unknown. ‘Mikrocytidiidae’ (Hartikainen et al. 2014 electronic supplement) is unavailable as a family name as no type genus was designated (contravening ICZN Article 16.2). (*Mikrocytos*, *Paramikrocytos*) **Order 4**. **Paradinida** Cavalier-Smith in Bass et al., 2009 (e.g. *Paradinium*) **Order 5**. **Paramyxida** Chatton, 1911 (*Marteilia*, *Paramyxa*, *Paramarteilia*) **Superclass 2**. **Proteomyxia** Lankester 1885 ex Cavalier-Smith, 2018 **Class 1**. **Vampyrellidea** Cavalier-Smith, 2018 **Order Vampyrellida** ord. n. West 1901 ex Cavalier-Smith. **Diagnosis**: as for 'Vampyrellida' in Hess et al., 2012 p. 10 (syn. Aconchulinida De Saedeleer 1934) Family 1. Vampyrellidae Zopf, 1885 (*Vampyrella*) Family 2. Leptophryidae Hess et al., 2012 (*Leptophrys*, *Platyreta*, *Theratromyxa*, *Vernalophrys*) Family 3. Arachnulidae Page, 1967 (*Arachnula*) Family 4. Hyalodiscidae Poche, 1913 (*Hyalodiscus*) **Vampyrellida incertae sedis**: *Thalassomyxa* Grell, 1985 **Class 2**. **Phytomyxea** Engler and Prantl, 1897 **Order 1**. **Phagomyxida** Cavalier-Smith, 1993 (*Phagomyxa*, *Maullinia*) **Order 2**. **Plasmodiophorida** Cook, 1928 (e.g. *Plasmodiophora*^a^, *Spongospora*^a^) **Subphylum 2**. **Ectoreta** Cavalier-Smith, 2018 (6 classes 23 orders) **Infraphylum 1**. **Foraminifera** (D’Orbigny 1826) Eichwald, 1830 stat. n. Cavalier-Smith, 2018 (about 15 orders) **Class 1**. **Monothalamea** Schultze, 1854 (4 orders including xenophyophores) (e.g. *Reticulomyxa*^a^, *Allogromia*) **Class 2**. **Globothalamea** Pawlowski et al., 2013 (9 orders, e.g. *Ammonia*^a^, *Bulimina*^a^, *Textularia*, *Globigerina*, *Globobulimina*^a^, *Elphidium*^a^, *Nonionella*^a^) **Class 3**. **Tubothalamea** Pawlowski et al., 2013 **Order 1**. **Miliolida** Delage and Herouard, 1896 em. Pawlowski et al. 2013 (e.g. *Sorites*^a^, *Quinqueloculina*^a^) **Order 2**. **Spirillinida** Hohenhegger and Piller, 1975 em. Pawlowski et al. 2013 (e.g. *Spirillina*, *Ammodiscus*) **Infraphylum 2**. **Radiozoa** Cavalier-Smith, 1987 em. stat. n. 2018 (7 orders) **Class 1**. **Polycystinea** Ehrenberg, 1838 stat. n. Cavalier-Smith, 1993 **Order 1**. **Collodarida** Haeckel, 1881 (as Collodaria, e.g. *Collozoum*^a^) **Order 2**. **Nassellaria** Ehrenberg, 1875 (e.g. *Lampromitra*) **Order 3**. **Spumellaria** Ehrenberg, 1875 (e.g. *Spongosphaera*^a^) **Class 2**. **Acantharea** Haeckel, 1881 stat. n. Cavalier-Smith, 1993 **Order 1**. **Arthracanthida** Schewiakoff, 1926 (e.g. *Phyllostaurus*^a^) (including Symphyacanthida Schewiakoff, 1926 (e.g. *Astrolonche*^a^) **Order 2**. **Chaunacanthida** Schewiakoff, 1926 (e.g. *Conacon*) **Order 3**. **Holacanthida** Schewiakoff, 1926 (e.g. *Acanthocolla*) **Order 4**. **Acanthoplegmida**^d^ Rechetniak, 1981 (*Acanthoplegma*) **Infraphylum 3**. **Sticholonchia** Cavalier-Smith infraphyl. n. (1 order) **Class Sticholonchea** Poche, 1913 stat. n. Petruschevskaya, 1977 **Order Taxopodida** Fol, 1883 (*Sticholonche*)

As there has been no recent comprehensive summary of cercozoan classification, families are shown for all Cercozoa except Phaeodaria and Ebriida (neither with recent changes), but only for vampyrellids in Retaria

^a^Taxa represented on our multiprotein trees

^b^Chantangsi and Leander (2010) used these names for clades but did not validly publish them as families

^c^Discomonadida were originally placed incertae sedis within Ventrifilosa and not assigned to a class (Scoble and Cavalier-Smith 2014); our more broadly sampled PhyloBayes 18S rDNA trees (e.g. Figs. S8, S12) make it even less likely that *Discomonas* are thecofiloseans. The Rhizaria-wide Figs S8–11 put them as weakly/insignificantly sister to Pediglissa plus Paracercomonadida and the probably more reliable Monadofilosa-only trees (Figs S12–15) put them within Imbricatea, grouping them with Placonuda; following the latter we place Discomonadida in Placonuda, consistent with their little known morphology

^d^The five rhizarian orders with no rDNA or other sequence evidence for their taxonomic position

^e^Without sequence data for this genus, we cannot be sure that the other three listed genera belong in this family

^f^Probably paraphyletic

^g^Amphitremida are Labyrinthulea (Gomaa et al. 2013; Takahashi et al. 2014) not Cercozoa (Cavalier-Smith and Chao 2012)

For analyses restricted to the 72 chromists from the 158-taxon trees (*Microheliella* absent), both chains converged satisfactorily (Fig. 2), and we got a corresponding ML tree (Fig. S2). Both methods strongly showed Ectoreta as sister to Endomyxa (1, 96%), forming a clade corresponding to expanded Retaria, with Endomyxa weakly (0.58, 45%) a clade. Both methods strongly showed Radiozoa as a clade (1, 99%). For Cercozoa, the (Fig. 2) CAT branching order was identical to the 158-taxon tree (Fig. S1, both chains) and to chain 1 of the 159-taxon tree (part of Fig. 1 analysis), with Granofilosea the deepest branch followed by *Bigelowiella* (Fig. 2). This is contradictory to Fig. 1 where *Bigelowiella* was weakly sister to Granofilosea (0.67 as shown in the consensus tree (Fig. 1 itself) and 0.87 in chain 2 alone).

In eukaryote-wide trees, basal branching for Cercozoa generally was more strongly supported for 158 taxa (Fig. S1 summing 25,433 trees, slightly more than for Fig. 2) than for 159 taxa (Fig. 1 summing only 5735). Monadofilosa is a maximally supported clade on 72- and 158-taxon trees and strongly so (0.93) on Fig. 1. All three trees had identical monadofilosan topology with *Micrometopion* (Metromonadea) strongly supported as the deepest branch (six independent chains agreed, i.e. both chains for the 159, 158 and 72 taxa analyses) (Figs. 1, S1 and 2). Next deepest is a clade comprising the sainouroid flagellate *Helkesimastix* and aggregative amoeba *Guttulinopsis* (order Helkesida, maximally supported as a clade on all trees by both methods: Figs. 1 and 2). Helkesida are sister to a large, moderately to strongly supported (0.92 Fig. 1; 0.97 Fig. S1; 0.75 Fig. 2) clade composed of the closely related Thecofilosea, Sarcomonadea and Imbricatea, all three of which are revised in Table 1. Overall, our chromist-only CAT trees strongly show Granofilosea as the deepest branching cercozoan lineage, in contrast to many extremely weakly supported but taxonomically much richer site-homogeneous 18S rDNA trees that tend to show *Bigelowiella* (Chlorarachnea) branching more deeply than Granofilosea (e.g. Cavalier-Smith and Chao 2003a; Bass et al. 2009a; Howe et al. 2011a; Yabuki and Ishida 2011; Shiratori et al. 2012; and our present ML trees, e.g. Fig. S9). By contrast, chromist-only ML trees grouped *Bigelowiella* and *Minimassisteria* with trivial support and misplaced *Limnofila* with similarly insignificant support; its position is more unstable than for *Minimassisteria* (see discussion regarding the conflicting positions of *Limnofila*).

### Sarcomonad phylogeny

**Fig. 3:**
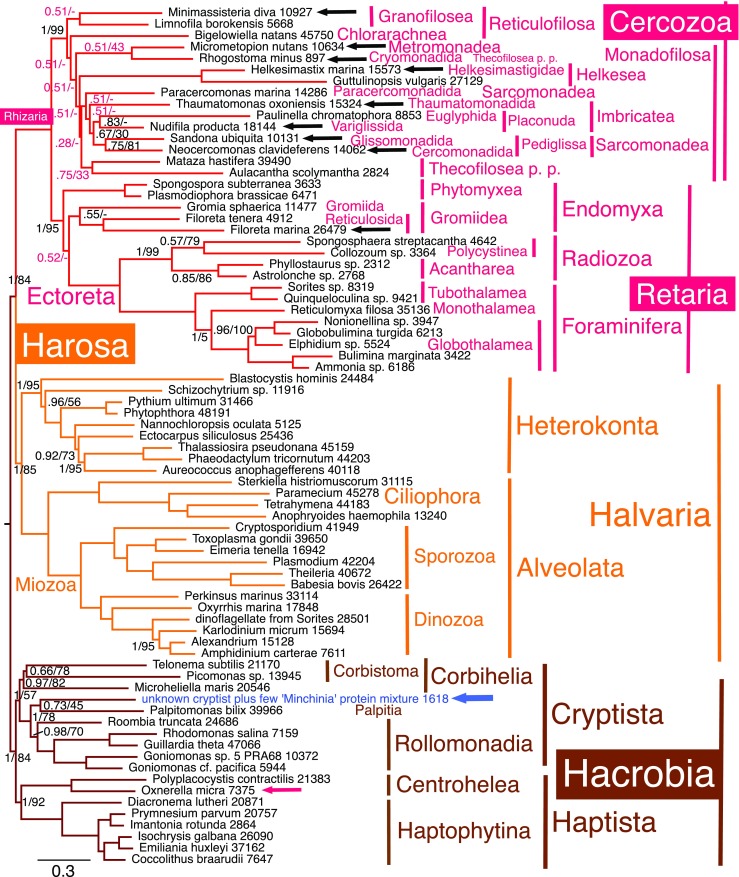
187-protein PhyloBayes GTR-CAT-Γ tree for 75 chromists; those on Fig. 2 plus *Rhogostoma*, *Microheliella* and ‘*Minchinia*’ (blue arrow). Black arrows show new rhizarian sequences; the red arrow highlights *Oxnerella*, whose sequences were phylogenetically separated from *Minimassisteria*. Numbers after species names show how many amino acids were included for each (maximum possible 50,964). Most bipartitions had maximal support by both CAT and ML; support values are only shown for those that did not (posterior probabilities left; ML 100 fast bootstraps right); in red for those where the two summed chains gave conflicting topology (max. diff. 1; 3499 trees summed after removing 1016 as burn-in; as the text explains, a third chain agreed with this consensus topology for Cercozoa, with much stronger support (except for the incorrect position of *Rhogostoma*), but placed Ectoreta as sister to Endomyxa in agreement with Fig. 2). The corresponding ML tree is Fig. S3. *P*. *p*. pro parte (in part)

**Fig. 4:**
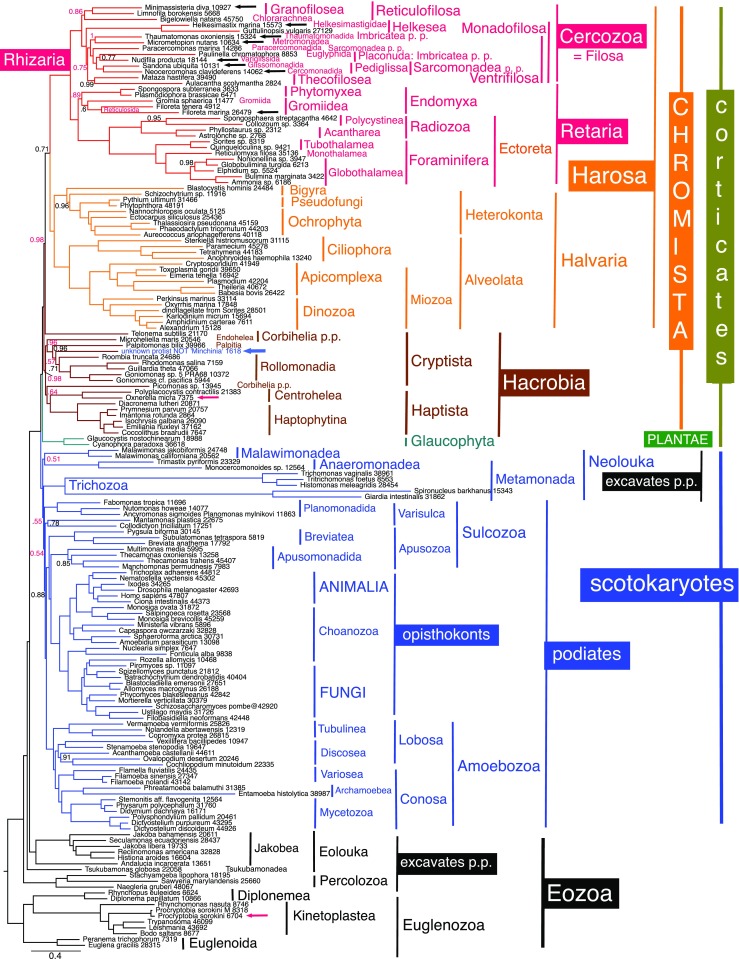
187-protein PhyloBayes GTR-CAT-Γ tree of 162 eukaryote-wide taxa, including short-branch glaucophytes to represent Plantae and ‘*Minchinia*’ (blue arrow). Black arrows show new rhizarian sequences and red arrows the two non-rhizarian sequences from the two mixed cultures that we separated phylogenetically. Numbers after species names show how many amino acids were included for each. Most bipartitions had maximal support on both chains; posterior probabilities are only shown if they did not—in red for those for which both chains did not show this topology (max. diff. 1); the tree shown is for chain 2 (3571 trees summed after removing 3148 as burn-in); in chain 1, Cryptista were rearranged, as in Cavalier-Smith et al. 2015a, with *Microheliella* forming a Corbihelia clade with *Telonema* and *Picomonas* (0.84 support) and Glaucophyta were weakly within Hacrobia (0.64, 0.56 support)

Within Sarcomonadea *Neocercomonas* is strongly sister to the glissomonad *Sandona* (maximally on Figs. 1 and 4, S1; 0.99 by CAT, 99% by ML in Fig. 2; support dropped to 0.75/81 on Fig. 3 that included the genically grossly undersampled *Rhogostoma*), corresponding to new sarcomonad subclass Pediglissa (Table 1), whereas *Paracercomonas* always branches substantially more deeply and is now put in a separate subclass in keeping with its distinctive cytoskeleton (see discussion). With CAT, the amoeboflagellate *Nudifila* and photosynthetic euglyphid amoeba *Paulinella* are sisters with moderate to strong support (0.75 Fig. 1; 0.88 Fig. S1; 0.85 Fig. 2; 0.83 Fig. 3; 0.77 Fig. 4), and this clade strongly supported (0.99, 0.99, 0.92, 0.67, 1) as sister to Pediglissa. In Fig. S2 (ML equivalent of Fig. 2), long-branch and genically sparsely represented *Paulinella* only is extremely weakly (29%) sister to Pediglissa; *Nudifila* is their immediate outgroup; this one node lower position, arguably a long-branch/missing data artefact, was similarly weakly supported on other ML trees (Figs. S3 and S4). This close relation between the imbricate *Nudifila*/*Paulinella* subclade and Pediglissa on all these trees shows that Sarcomonadea are probably paraphyletic, and cercomonads are polyphyletic, as some ML rDNA trees and ultrastructure previously tentatively suggested (Cavalier-Smith and Karpov 2012b). All (Figs. 1, 2 and 3) CAT trees put the other scaly imbricate flagellate *Thaumatomonas* (sole representative of imbricate subclass Placoperla) as sister to the Pediglissa/*Nudifila*/*Paulinella* clade, thus closer to *Neocercomonas* than is *Paracercomonas*, reinforcing cercomonad polyphyly. No trees group *Thaumatomonas* with *Paulinella*/*Nudifila* as would be expected were Imbricatea a clade. The grouping of Pediglissa instead with *Paulinella* and *Nudifila* is sufficiently strong by CAT in Figs. 1 and 2 that Imbricatea are most likely paraphyletic. Imbricate subclasses Placonuda and Placoperla each has a mixture of naked species and others secreting siliceous surface structures—scales in Placonuda, scales or perles in Placoperla. In addition, we establish here the new imbricate subclass Krakenia (see below) to include the unique scaly reticulose amoeba *Kraken* (Dumack et al. 2016b, 2017a).

The joint Sarcomonadea/Imbricatea clade is strongly supported on Figs. 1 and 2 CAT trees (weakly on Fig. 3 in the presence of grossly undersampled *Rhogostoma*). However, except for the strong clade Pediglissa, no branches within that joint clade are significantly supported by ML (showing that CAT is superior for these very close branches), though ML internal branching order of Sarcomonadea is the same except for *Nudifila*/*Paulinella* not being a clade. However by ML (Fig. S2), *Micrometopion* moved into sarcomonads to become sister to *Thaumatomonas* (trivial 25%; seen also on one CAT tree: Fig. 4) and *Limnofila* also wrongly moved into sarcomonads to be sister to *Paracercomonas* (insignificant 42%) and *Minimassisteria* became sister to *Bigelowiella* (26%). This further shows that ML LG is often less accurate than CAT, but there are no statistically significant contradictions by ML to the stabler, well-supported CAT-based topology.

Incorrect attraction of *Limnofila* and *Paracercomonas* might be an artefact of weak gene sampling or else stem from contamination of the sequenced culture by a paracercomonad, as our discussion explains. If the *Limnofila* transcriptome has contaminating paracercomonad genes, they might also artefactually pull *Paracercomonas* away from *Neocercomonas* giving a spurious position intermediate between *Micrometopion* and *Neocercomonas*. To test whether *Paracercomonas* is misplaced in Figs. 1, 2, 3 and 4 by the possible presence of paracercomonad genes in a contaminated *Limnofila* culture, we also ran trees for Rhizaria only including *Limnofila* (31 taxa Fig. S5) and excluding it (30 taxa Fig. S7). Figure S7 shows that *Paracercomonas* still does not group with *Neocercomonas* even when *Limnofila* is excluded, thus the poly/para-phyly of cercomonads is robust and not distorted by the sparsely sampled and likely contaminated *Limnofila* transcriptome.

### Phylogeny of Thecofilosea

*Mataza* and the phaeodarian *Aulacantha* are consistently a clade (class Thecofilosea) with moderate to strong CAT support on Figs. 1, 2, 3 and 4 (0.95; 0.94; 0.75; 0.99) but weak ML support (49% Fig. S2; 33% Fig. S3) probably because of low gene representation for *Aulacantha*. The *Mataza* sp. sequences (MMETSP0087) are from a previously unidentified cultured marine protist from California (downloaded from http://marinemicroeukaryotes.org/resources; strain D1 Palenik unpub.; link no longer available—http://data.imicrobe.us/sample/view/1715 is a replacement), which our cercozoan 18S rDNA tree (Supplementary Fig. S8) shows is so closely related to *Mataza hastifera* (Yabuki and Ishida 2011) that it must belong to that genus, but sufficiently different that it must represent a second undescribed species.

Partial genome sequencing from the thecofilosean amoeba, *Rhogostoma minus*, belonging to a different order (Cryomonadida) from the other thecofiloseans, *Mataza* and *Aulacantha*, provided too few sequences for our 187 genes (only 897 amino acids) to be included in Figs. 1 or 2 without risking distortions because the vast majority of genes are missing. As multigene trees can sometimes place incomplete short sequences correctly if trees include a fairly close relative with good gene sampling, we also ran chromist-only trees after adding *Rhogostoma* and sequences ostensibly from the ascetosporean parasite ‘*Minchinia*’: an analysis of 75 taxa (Fig. 3) including all 73 chromists from Fig. 1, and another of 74 taxa including all 72 chromists from Fig. S1 [as CAT did not fully converge (max. diff. 1), Fig. S4 shows only the ML analysis]. These 75- and 74-taxon trees were generally similar to the 72-taxon tree (Fig. 2) but *Rhogostoma* was contradictorily placed: one (Fig. 3) CAT chain put it in the maximally supported Thecofilosea clade as sister to *Aulacantha* (0.99) in agreement with rDNA (Howe et al. 2011a), the other probably wrongly as sister to *Micrometopion* (1). In Fig. 3, the two chains were contradictory in some places (e.g. one put Ectoreta as sister to Endomyxa as in Fig. 2 and the other placed it within Endomyxa as sister to Gromiidea as shown weakly on Fig. 3 consensus tree) probably because they were run for much less time than for Fig. 2. Therefore, we ran a third 75-taxon chain somewhat longer and summed 2194 trees; this also showed Ectoreta as sister to Endomyxa, being excluded from them with 0.61 support (also by ML: Fig. S3, 52% support).

A separate 32-taxon multigene tree restricted to Rhizaria (supplementary Fig. S6; maxdiff 0.278303) grouped *Rhogostoma* correctly but with trivial support (0.36) within Thecofilosea (as sister to the phaeodarian *Aulacantha* rather than to *Mataza* as with rDNA, all three a weak (0.44) thecofilosean clade). Thus in the absence of non-rhizarian outgroups, CAT can place *Rhogostoma* with only 897 amino acids almost as expected from taxon-rich 18S rDNA trees. Despite adding *Rhogostoma* and ostensible ‘*Minchinia*’ (Fig. 3), internal phylogeny of both rhizarian phyla by CAT is identical to Figs. 1 and 2. Thus *Rhogostoma*’s sequence shortness makes little or no difference to tree topology but prevents its reliable placement because so few amino acids cause high random errors. That is consistent with 18S rDNA trees where all three Thecofilosea mutually diverge close to the base of Thecofilosea, with *Aulacantha* deepest (Fig. S8; Howe et al. 2011a; Yabuki and Ishida 2011where the *Rhogostoma* sequence was incorrectly called *Lecythium* sp. because of earlier misidentification).

### Endomyxan phylogeny

*Minchinia* is a haplosporidian parasite, which rDNA trees established as an endomyxan cercozoan (Cavalier-Smith 2002a; Bass et al. 2009a). The fact that supposed ‘*Minchinia*’ did not group with Endomyxa (as it always does by rDNA (Figs. S8–S11) or even within Rhizaria or Harosa, but strongly (1, 84%) within Hacrobia (Fig. 3) shows that most sequences included from this transcriptome are not from *Minchinia* but unrelated contaminants from one or more deep-branching hacrobians (as methods explain, we removed obvious diatom and *Micromonas* contaminants, but our single-gene trees could not have detected all chromist contaminants as they included no certain ascetosporan proteins). As one cannot cultivate ascetosporans, their spores have to be extracted from wild marine invertebrates, making contamination by unrelated marine protists likely. In the 74-taxon tree, ‘*Minchinia*’ is weakly (0.5, 42% Fig. S4) sister to the deep-branching marine cryptist *Palpitomonas*. As ‘*Minchinia*’ groups within Cryptista, we also ran 75-taxon trees including the pseudoheliozoan *Microheliella* recently shown to branch nearby (Cavalier-Smith et al. 2015a) in case this would clarify its position. This confirmed that ‘*Minchinia*’ branches as sister to *Palpitomonas* (0.73, 45%) and that *Microheliella* is sister to Corbistoma and does not branch with ‘*Minchinia*’ (Fig. 3). This tree did not converge, chains differing in three ways: (1) one showed Endomyxa as a clade, the other made it paraphyletic with Ectoreta sister to Gromiidea only (and on consensus Fig. 3); (2) one correctly grouped *Rhogostoma* with *Aulacantha* (0.51) (but wrongly put the three-species Thecofilosea clade as sister to *Thaumatomonas* not Sarcomonadea as in Fig. 2), whereas the other wrongly put *Rhogostoma* with *Micrometopion* (1.0); (3) *Limnofila* was correctly with *Minimassisteria* on one chain, wrongly with *Paracercomonas* on the other. For CAT, cercozoan topology is otherwise identical to Figs. 1 and 2; ML topology was identical for the 72, 74, 75 taxon trees. This novel inclusion of ‘*Minchinia*’ within Cryptista did not alter its topology from that recently established (Cavalier-Smith 2015).

To rule out the possibility that ‘*Minchinia*’ belongs to a non-chromist group excluded from Fig. 3, we ran a 162-taxon CAT tree including it (and *Microheliella*) and two glaucophytes as short-branch Plantae to rule out the possibility that ‘*Minchinia*’ is closer to plants than to chromists. In this broader analysis, ‘*Minchinia*’ was not sister to *Palpitomonas* but instead to either Rollomonadia (Cryptomonada plus Leucocrypta) in chain 2 (Fig. 4) or to core Cryptista (*Palpitomonas* plus Rollomonadia) on chain 1 and the consensus tree. Although the two chains did not fully converge, their topology was identical in all except five respects: (1) they showed exactly the same conflict in the position of the two metamonad clades as did the 158-taxon analyses; (2) the exact position of Retaria differed—strongly sister to Endomyxa in chain 2 (Fig. 4) and the consensus but weakly (0.54) to Gromiidea only in chain 1; (3) *Microheliella* was sister to ‘*Minchinia*’ plus core Cryptista in chain 2 but to Corbistoma in chain 1 and the consensus tree forming a Corbihelia clade as in Fig. 3 and previously (Cavalier-Smith et al. 2015a); (4) *Micrometopion* is wrongly sister to *Thaumatomonas* in chain 2 but in the same position as on Figs. 1, 2 and 3 in chain 1 and the consensus tree. (5) Chromista were holophyletic with maximal support (with Glaucophyta maximally supported as their sister) on chain 2 (Fig. 4), but chain 1 put glaucophytes weakly (0.71) within them as weak sister to core Cryptista plus ‘*Minchinia*’. This shows that supposed ‘*Minchinia*’ is not a harosan, rhizarian or cercozoan, and not closer to any non-chromist group. We ran the analysis long enough to establish that, but given the agreement between Figs. 1, 2, 3 and 4 on the internal phylogeny of Cercozoa, it was not worth while to run them longer in the hope of reducing these conflicts, especially as these sequences are apparently predominantly from a cryptist contaminant [a core cryptist (Fig. 4) or one related to *Palpitomonas* (Fig. 3)] or from one or more contaminants mixed with some genuine *Minchinia* genes (see ‘Discussion’ section).

Figures 1, 2, 3 and 4 CAT trees show Radiozoa as a clade with maximal support, as did one chain of the 158-taxon analysis. Radiozoa are also a clade on corrresponding ML trees with strong support: 99% for 72/75-taxon trees (Figs. 2/S2; 3/S3) and 100% support for 74 taxa (Fig. S4).

Within Endomyxa, our extensive new sequences for *Filoreta marina* group with maximal support with the previously sequenced but sparsely sampled *Filoreta tenera*. However, two contradictory phylogenies were found for Endomyxa. In four CAT analyses, *Filoreta* and *Gromia* are sisters in all eight chains, a clade corresponding to revised class Gromiidea (Table 1); support was weak in chromist-wide samples that did not converge (Fig. 3 0.55; Fig. 4 (0.60 in chain 2 shown; 0.68 in consensus, chains not converged), but strong in smaller, better converged Rhizaria-only alignments—in a 31-taxon tree that excluded *Rhogostoma* (Fig. S5 max. diff. 0.290668; 0.97) and a 32-taxon one with *Rhogostoma* (Fig. S6, max. diff. 0.278303; 0.95). The 31-taxon ML tree had a strong (96%) Gromiidea clade. In four other CAT trees, Gromiidea was weakly paraphyletic with *Gromia* sister to plasmodiophorids instead (Figs. 1, 2 and S1). ML trees corresponding to Figs. 2 and 3 had weakly paraphyletic Gromiidea (Figs. S2–4: 57, 63 and 55%).

Endomyxa are most often a clade, moderately supported (0.71) on Fig. 1, weakly (0.58, 45%) on Fig. 2. However, in contrast to all our other trees, our converged 31-taxon Rhizaria-only tree (Fig. S5) placed Ectoreta between Phytomyxea and Gromiidea; if rooted as in Fig. 4, they would be sisters to Gromiidea with 0.99 and 65% support; the same contradiction is evident on Fig. S6 (that includes *Rhogostoma*), and thus is a consistent feature of Rhizaria-only trees—it might result from long-branch attraction of the very long Foraminifera/Radiozoa branch towards the gromiid branch that is longer than the phytomyxan one. In Figs. 1, 2, 3 and 4 and S1–4, the presence of non-rhizarian outgroups that break the stem between Cercozoa and Retaria might largely or entirely prevent that by enabling algorithms to reconstruct ancestral states more accurately.

### Site-heterogeneous rhizarian 18S rDNA trees

Our comprehensive site-heterogeneous Bayesian 18S rDNA trees with 467 Rhizaria (Fig. S8) agree (as do trees with fewer or no Helkesida—Figs. S10/11) with the taxonomically far sparser multiprotein trees in placing Granofilosea more deeply than Chlorarachnea. 18S rDNA shows Gromiidea as monophyletic (CAT and ML: paraphyletic because Ascetosporea are robustly sister to Gromiida only). Because in some respects these trees differ within Monadofilosa from the previous most comprehensive CAT analyses for Monadofilosa (Scoble and Cavalier-Smith 2014), and because these analyses were done before the remarkable aciliate filose amoeba *Kraken* was discovered [shown to have single-tier (?silica) scales and flat cristae, a previously unknown structural combination] (Dumack et al. 2016b, 2017a), we also did analyses restricted to 315 or 316 Monadofilosa (using 1790 amino acid positions: Figs. S12–S15) including *Kraken* and several recently discovered Thecofilosea and imbricates, with one short-branch granofilosean as an outgroup. New sequence addition enabled slight improvements in alignment in the most divergent regions (mainly in Thecofilosea and imbricates) so we could include the whole molecule (only terminal PCR primer regions and rare inserts were excluded). In one notable respect, the Monadofilosa-only 18S rDNA trees (Figs. S12–15) disagreed with the Rhizaria-wide 18S rDNA trees but agreed with the CAT multiprotein trees (Figs. 1, 2, 3 and 4): Cercomonadida are not sisters of Paracercomonadida. Yet when distant rhizarian outgroups are included in rDNA trees (Figs. S8, S10 and S11), paracercomonads are sister to cercomonads with moderate support—one of our ML tree groups them (Fig. S9) with insignificant support but the others do not; published site-homogeneous trees may group them or not depending on taxon sampling. Supplementary Material (after Fig. S15) discusses other phylogenetically important aspects of the new cercozoan-wide rDNA analyses.

**Fig. 5:**
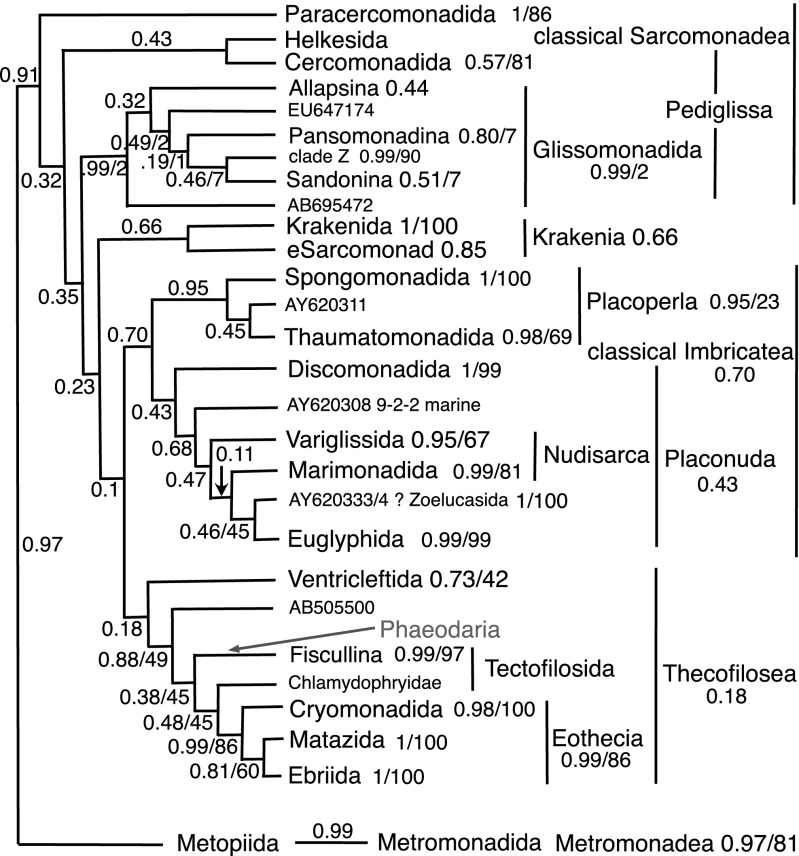
Summary of ordinal and subordinal relationships amongst Monadofilosa as shown by a site-heterogeneous tree for 317 18S rDNA sequences (complete tree is Fig. S12). Support values are posterior probabilities from Fig. S12 and on the right also for ML (Fig. S14) for the same alignment when exactly the same clades were present. Support values for terminal clades are to the right of their names. The position of Phaeodaria (excluded from these analyses in case their long branches artefactually attracted *Cholamonas*) is from the Rhizaria-wide analyses (Figs. S8–S11) where they were sister to Fiscullina irrespective of *Cholamonas* presence or absence

These Monadofilosa-only CAT analyses (Fig. S12 including *Cholamonas*, S13 omitting long-branch helkesid *Cholamonas*) were more similar in topology to the Scoble and Cavalier-Smith trees, suggesting that adding more distant, often longer branch, reticulofilosan and retarian outgroups in Rhizaria-wide trees slightly distorts branching order in the hard-to-resolve ventrifilosan basal radiation. CAT trees (summarised for the more comprehensive analysis in Fig. 5) differed from that of Scoble and Cavalier-Smith only in two phylogenetically important (but statistically insignificant) respects. First, *Spongomonas* is sister to Thaumatomonadida in agreement with some ML trees and with Rhizaria-wide analyses, and did not branch within them as sister to the deepest (Peregriniidae/Esquamulidae) clade as it did (probably incorrectly) in Scoble and Cavalier-Smith (2014). Second, *Discomonas* was not sister to classical imbricates but deeply within them as sister to Placonuda, as in an earlier Rhizaria-wide analysis that led to their classification in Imbricatea (Howe et al. 2011a), but in disagreement with our present Rhizaria-wide analyses that weakly put *Discomonas* as sister to Pediglissa plus paracercomonads.

We also did 316/7 taxon ML trees (Figs. S14 and S15); all four Monadofilosa-only trees failed to group *Kraken* with paracercomonads as taxonomically much sparser site-homogeneous trees originally found (weakly by 18S alone, strongly with both rDNAs: Dumack et al. 2016b, 2017a). By CAT *Kraken* was sister instead to environmental DNA clade eSarcomonad (on Figs. S8, S10–11 within classical imbricates, but as in Fig. S9 weakly sister to glissomonads as originally: Scoble and Cavalier-Smith 2014), not included by Dumack et al. (2016b), with marginally stronger support (0.64: Figs. 5 and S12) than they found for the paracercomonad grouping. On S12, the helkesid *Cholamonas* was insignificantly (0.43) sister to Cercomonadida and Paracercomonadia were the deepest branching sarcomonad clade. Thus as in Dumack et al. (2016b), Cercomonadida and Paracercomonadida were not sisters, but when *Cholamonas* was omitted they became sisters (Fig. S13 0.84, but only 0.5 in a 318 taxon analysis with an extra deep-branching imbricate). Omitting *Cholamonas* made eSarcomonad unstable (Fig. S13): one chain showed the same topology as Figs. 5 and S12 but in the other only the two original short-branch eSarcomonad sequences remained significantly (0.67) sisters to *Kraken*, the two longer ones that branched within them moved into pansomonads within Glissomonadida. As their intrusion into pansomonads reduced support for pansomonads, glissomonads and subclass Krakenia (established in Table 1 for *Kraken* plus eSarcomonad) on the consensus tree (Fig. S13), it is likely a long-branch attraction artefact. This confusing attraction may be specifically related to aberrant long-branch environmental DNA AY620290 which by ML grouped within eSarcomonad not within pansomonads (see additional supplementary discussion). One consistent difference from the Rhizaria-wide trees (Figs. S8–11) is that Ventricleftida did not group with or near Helkesida or become two separate clades. Ventricleftida were a moderately supported clade sister to other Thecofilosea as in Howe et al. (2011a) not to classical imbricates as in Scoble and Cavalier-Smith (2014). Our trees confirm that environmental DNA AB695519 from Antarctic lake moss is *Kraken* and also identify a soil sequence from a North American aspen forest (EF024287) as from *Kraken*. With ML *Kraken* did not group with eSarcomonad or any other specific clade but was the third deepest branch after metromonads and discomonads without significant support. eSarcomonad (probably wrongly including AY620290) was insignificantly (9%) sister to glissomonads.

All Monadofilosa-only trees rooted solely by a short-branch granofilosan showed Metromonadea as a clade (up to 98% support), indicating that including long-branch Chlorarachnea and more distant long-branch outgroups in the Rhizaria-wide (and similar published) trees probably distorts monadofilosan topology by making them wrongly appear paraphyletic. Thus our multiprotein trees including only *Micrometopion* likely accurately represent the position of the whole class as the deepest branching Monadofilosa. By CAT (not ML), rDNA Metromonadea was the deepest-branching monadofilosan clade. Our trees also more clearly than before establish branching order within Marimonadida: *Cyranomonas* is more closely related to the *Pseudopirsonia*/*Auranticordis* clade than to *Abollifer* (Figs. S12–S15). We establish new marimonad families to reflect their ultrastructural diversity and substantial branching depth on the trees (Table 1), and a new family for *Quadricilia* (rDNA sequence unavailable when we ran our trees), which goups with *Clautriavia* and *Nudifila* (weakly by ML but strongly on a site-homogeneous Bayesian tree) as the first non-gliding variglissid (Yabuki and Ishida 2018). Their ML tree weakly grouped *Kraken* with *Ventrifussura* as sister to Imbricatea, and like ours did not group them with paracercomonads as in Dumack et al. (2016b) and thus agrees with our placing *Kraken* in Imbricatea (Table 1). Figures 5, S12 and S13 trees weakly show superorder Nudisarca as a clade, as in Rhizaria-wide trees (insignificantly paraphyletic in Figs. S14–15).

### The overall eukaryote multiprotein tree

In the absence of Plantae, chromist subkingdoms Harosa and Hacrobia are sister clades, both maximally supported (Figs. 1 and S1). The maximally supported chromist clade is sister to the maximally supported scotokaryote clade (i.e. podiates plus *Malawimonas* and Metamonada). Halvaria (comprising Alveolata and Heterokonta) is a maximally supported clade by CAT in Figs. 1, 2, 3, 4, and S1, although not always by ML (95% in 2/S2, 82% in 3/S3). Alveolata is always a maximally supported clade by both methods (Figs. 1, 2, 3, 4 and S1). Heterokonta are maximally supported by CAT and usually maximally by ML (except Figs. 3 and S3). However, in the presence of glaucophytes to represent Plantae, support for Harosa reduced and *Telonema* (only) was excluded from the Hacrobia clade but Chromista remained a maximally supported clade that was maximally supported as sister to Plantae (i.e. superkingdom Corticata is a clade). Whether movement of *Telonema* was caused by adding glaucophytes or the ‘Minchinia’ sequence that might in fact be a mixture of hacrobian and harosan sequences is unclear, but does not affect Fig. 4’s clear evidence for holophyly of Chromista.

As noted above, CAT trees for the exceptionally large Fig. 1 alignment did not converge because of contradictions within Rhizaria. Topology was identical for both chains for all non-rhizarian branches, including those that gave contradictory results in published trees near the base of scotokaryotes and Hacrobia (e.g. Cavalier-Smith et al. 2014, 2015a). The hacrobian sister phyla Cryptista and Haptista were each clades in Figs. 1, 2 and 3 by both methods: Haptista were always strongly supported as a clade by both methods (maximally or nearly so by CAT), whereas CAT support for Cryptista was maximal in Figs. 1 and 3 but weak in Fig. 2. Presence of the ‘Minchinia’ putative cryptist/haplosporidian mixture disrupted Cryptista on eukaryote-wide (Fig. 4) but not on the chromist-only (Fig. 3) where cryptist subphylum Corbihelia was a clade.

Topology of Eozoa was maximally supported throughout and identical to all our previous multigene papers. Within scotokaryotes, Metamonada are strongly a clade on Fig. 1 (0.96% support) and the long-branch Trichozoa did not separate from the short-branch Anaeromonadea as on Fig. S1 and some earlier trees (Cavalier-Smith et al. 2015a, b). Figure 1 also provides stronger evidence than before that Metamonada are holophyletic. Metamonada grouped with *Malawimonas* as a moderately supported (0.79) clade, in contrast to several earlier CAT trees (Cavalier-Smith et al. 2015a, b, 2016). However, earlier CAT trees in the Metamonada/*Malawimonas* region tended not to converge or were poorly converged. Figure 1 had maximal support for planomonads being the deepest podiate clade followed by a clade comprising *Mantamonas* and *Collodictyon* (0.99, much stronger support than the 0.51 found when this clade was first seen: Cavalier-Smith et al. 2014). Within obazoa, successive branching of breviates, apusomonads and opisthokonts and their internal branching was exactly as before and (except for one apusomonad node) maximally supported.

However, a 158-taxon tree excluding *Microheliella* that was run much longer still did not converge (Fig. S1 max. diff. 1) and showed discrepancies within Rhizaria and in two other regions, suggesting that non-rhizarian inter-chain agreement with 159 taxa was fortuitous. Topology was identical for both chains on the 158-taxon trees except in three local regions with very close deep branches, all having shown similar contradictions amongst previously published trees. The first concerns basal branching within scotokaryotes (unlike Fig. 1 that for the first time in any CAT tree shows a strongly supported metamonad clade); its two deeply divergent subclades do not group together and are contradictorily placed. In chain 1, unlike Fig. 1, only anaeromonads were sister to *Malawimonas* (0.62; 0.47 in the consensus tree); the long-branch Trichozoa were one node higher as sister to all other scotokaryotes (0.61). Contradictorily in chain 2, anaeromonads were one node deeper (below *Malawimonas*; 0.49) and Trichozoa one node higher (above Planomonadida 0.46; 0.40 in the consensus tree). Secondly, *Picomonas* and *Telonema* were sister on chain 2 (0.89; 0.59 on consensus tree) as in Fig. 1, with this Corbistoma clade (Cavalier-Smith et al. 2015a) being wrongly sister to all other Hacrobia, not just to other Cryptista as correctly in Fig. 1. However, chain 1 put *Telonema* one node lower as sister to Harosa (0.51; incorrect, see Cavalier-Smith et al. 2015a) and *Picomonas* higher, as (correctly) sister to other cryptists (0.46). This less good topology for Corbihelia probably stems partly because, as previously found (Cavalier-Smith 2015), including *Microheliella* (Fig. 1 with thus three corbihelians) stabilises trees compared with having only two corbihelians [Fig. S4; in Burki et al. (2016) without *Microheliella* Corbistoma was not a clade]. Thirdly, amongst basal Rhizaria, Ectoreta were sisters to a moderately supported (0.90) classical Cercozoa clade and strongly excluded from Endomyxa (0.99) on chain 2 based on 11,912 trees but are weakly (0.74) sister to Endomyxa on chain 1 based on 13,480 trees (also excluded from Endomyxa quite weakly: 0.77). Within Ectoreta, for Radiozoa, the chains were highly contradictory, one showing it as a clade, the other as paraphyletic both with maximal support.

## Discussion

Sequence information now available places 60 of the 65 highly diverse rhizarian orders (Table 1) with reasonable confidence on the evolutionary tree. Many aspects of their cell evolution are clearer, though three orders still need sequence evidence to verify that they are truly cercozoan: helioflagellate Axomonadida; the silica-scaled filose amoeboid order Zoelucasida; and Perlofilida, filose non-flagellates with silica perles. Two retarian orders also lack sequence evidence, but morphology makes it virtually certain that Acanthoplegmida are Acantharea and likely that Claustrosporida are Ascetosporea. We here not only discuss our results but use them to clarify many aspects of rhizarian phylogeny and cell evolution.

### Distinctness of Gromiidea and Foraminifera

The idea that the highly reticulose Foraminifera might be related to the predominantly filose testate marine amoeba *Gromia* predates sequencing studies; *Gromia* was initially considered a foraminiferan despite its pseudopodia being non-granular and filose (with rare anastomoses) not granular and highly reticulose as in Foraminifera. This misconception stemmed largely from confusion with the superficially similar foraminiferan *Allogromia* (Arnold 1972) and the presence in both of a complex sexual life cycle (Arnold 1966, 1972)—though *Gromia* gametes are uniciliate not biciliate as in Foraminifera. Later, *Gromia* was placed in Testaceafilosea (de Saedeleer 1934) a now abandoned polyphyletic group that included all testate amoebae later included in Cercozoa (Cavalier-Smith 1998a; Cavalier-Smith and Chao 2003a). The first 18S rDNA trees for *Gromia* strongly grouped it with Cercozoa and showed Cercozoa and Acantharea (one of the three classes of Radiozoa) as sisters (Burki et al. 2002), but were contradictory, one weakly grouping it with Phytomyxea, the other as sister to Phytomyxea plus Filosa, neither with significant support; Foraminifera were excluded from the tree because of their long branches. The idea of a *Gromia*/foraminiferan relationship was revived when single-gene trees including both sometimes grouped them together or nearby (18S rDNA, Berney and Pawlowski 2003; RNA polymerase II, Longet et al. 2003, 2004; Longet and Pawlowski 2007; actin, Longet et al. 2004; Flakowski et al. 2005, 2006). Tekle et al. (2007) found the long-branch Foraminifera and Ascetosporea (parasites of invertebrates) grouping together by rDNA on site-homogeneous Bayesian trees (although not with ML, wrongly stated to be congruent), but on actin trees Foraminifera were sisters to *Filoreta* (= *Corallomyxa*) *tenera* (an ATCC culture earlier misidentified as *Corallomyxa*, later named *Filoreta*: Bass et al. 2009a) not to *Gromia*. More comprehensive cercozoan 18S rDNA trees showed *Gromia* as sister to the marine Ascetosporea rather than to Phytomyxea (Bass et al. 2012; Cavalier-Smith et al. 2008, 2009) and also showed a clade Endomyxa with weak support (Bass et al. 2009a) and a distinct classical Retaria clade (Acantharea, Polycystinea, Foraminifera) separate from Endomyxa and other Cercozoa. Our present ML and site-heterogeneous CAT rDNA trees where Ectoreta is represented only by the shorter branch Radiozoa (long-branch Foraminifera excluded to prevent artefacts) all grouped *Gromia* and *Filoreta* with Ascetosporea, never with Ectoreta or Cercozoa. Neither our nor any other gene-rich multiprotein trees group *Gromia* with foraminifera; all robustly exclude it from Ectoreta and place it nearer *Filoreta*, usually as sister. The idea that *Gromia* is specifically related to Foraminifera is firmly refuted.

### Demarcation and relationship between phyla Cercozoa and Retaria

Multigene analyses of Rhizaria have also been contradictory. The 167-gene tree (36,735 amino acids) of Burki et al. (2010) excluded Ectoreta (2 Foraminifera, 2 Acantharea) from classical Cercozoa with maximal support by ML LG and MrBayes WAG analyses, and showed Endomyxa (just *Gromia* and two Phytomyxea) as a clade with 99% and 1.0 support. Contradictorily, their PhyloBayes CAT (not the evolutionarily better, computationally more onerous GTR-CAT used here) placed Ectoreta as sisters to *Gromia* with 0.92 support, but support for the Endomyxa/Retaria clade was neglible (0.51). When *Reticulomyxa*, the ectoretan with the highest gene representation, was omitted (foraminifera represented only by *Quinqueloculina* with less than 32 genes or omitted altogether), ML and CAT both put Ectoreta (or Acantharea alone) as strongly sister to *Gromia* and support for an Acantharea/Endomyxa clade also increased. A 36-gene analysis of Harosa (with only haptophytes as outgroup) with 4 Endomyxa including 12 genes from *Filoreta* (under the wrong name *Corallomyxa*) and 22 Rhizaria (Sierra et al. 2013) placed Ectoreta as sister to *Filoreta* plus *Gromia*, with moderate support ML LGF (78%) and 0.99 by CAT-Poisson (chains did not converge; it is unclear whether their statement applied to both CAT-Poisson and CAT-GTR analyses). However, sampling was low (5 Cercozoa, 4 Endomyxa) and the presumably unconverged CAT 36-gene tree peculiar in that plasmodiophorids were sister to all other Rhizaria (not found on the ML tree not shown, or on their CAT-Poisson 109-gene Fig. S1 tree, that was more sensible for Cercozoa). This oddity probably stems mainly from having only nine genes for one and seven for the other plasmodiophorids (only three shared by both). Brown et al. (2012) using 159 proteins (43,615 amino acids), 11 Rhizaria but only 3 Endomyxa also found Radiozoa as sisters of *Gromia* with maximal Bayesian (CAT Poisson) and 52% ML LG support. Retaria were also sisters of *Gromia* on the 238-gene (50,293 amino acids) tree of Burki et al. (2012) but this included only 5 Rhizaria, all except *Bigelowiella* with 68–81% of amino acids missing.

A recent analysis (Sierra et al. 2016) with improved endomyxan gene and taxon sampling removes some of these problems and put Ectoreta as sister to the Ascetosporea/Gromiidea clade, agreeing with our conclusion that they are not specifically related to Gromiidea. Rhizarian multigene phylogeny has been plagued by gene sampling for Endomyxa and Ectoreta being generally low with much missing data, as well as by long branches of Retaria on sequence trees. Except for that study and a 119-protein tree (23,162 amino acids) including *Mikrocytos* (Burki et al. 2013), whose branch is so immensely long that one cannot rule out the possibility of tree distortion by attraction towards other long ones like Foraminifera, our study is the first to include an endomyxan with a fairly high gene representation (*Filoreta marina*: 26,479 amino acids). This may have stabilised some trees and helped to exclude Ectoreta; for Ectoreta, only *Reticulomyxa* has high gene sampling (35,136 amino acids). Having a second filosan, *Mataza*, with high gene representation (39,490 amino acids, albeit less than the 45,750 of *Bigelowiella*) will have helped stabilise our trees, as will now having 14 Cercozoa (only 3–5 on previous rhizarian multigene trees). Though a minority of our CAT trees had conflicting topologies, we now conclude that Ectoreta are most likely sister to Endomyxa as a whole, not to *Gromia* only or *Filoreta* only, nor to *Gromia* plus *Filoreta*, as some previous trees suggested—though Sierra et al. (2016) found paraphyletic Endomyxa by four site-homogeneous methods, with site-heterogeneous PhyloBayes CAT-GTR (seemingly not the better CAT-GTR-GAMMA we used) support for that was insignificant 0.5; thus, that tree does not significantly contradict our conclusion. Krabberød et al. (2017) have now added two important ectoretans to a 255-protein tree, a nassellarian radiozoan and *Sticholonche* that has a unique cell organisation, and increased gene sampling for foraminifera. Their analysis, though much less well sampled than ours for monadofilosan Cercozoa and Endomyxa, has maximal support for Rhizaria, Ectoreta, Endomyxa, Retaria and Cercozoa as now revised, all being clades and Ectoreta and Endomyxa being sisters. Thus, it fully supports the transfer of Endomyxa from Cercozoa to Retaria (Cavalier-Smith 2018).

Our conclusion that Ectoreta are probably sister to all Endomyxa differs from the classical idea of a direct link between Foraminifera and *Gromia* only and also from previously found endomyxan topologies, but agrees with the conclusion that classical Cercozoa are ancestral to classical Retaria and thus were paraphyletic (Burki et al. 2010, Sierra et al. 2013, 2016). Thus, similarities between *Gromia* and Foraminifera are superficial reflections of convergent evolution of a perforated test, and sharing a reticulose ancestor with other Endomyxa and Radiozoa, as has long been recognised by their separate classification. Broadly similar tests evolved several times also in Cercozoa. An 18S rDNA deletion that enabled the design of phylum-specific PCR primers (Bass and Cavalier-Smith 2004), and was uniquely shared by Endomyxa and former Filosa, previously favoured holophyly of classical Cercozoa (Cavalier-Smith 2002a); our multiprotein trees make it likely that this single nucleotide deletion arose in ancestral Rhizaria and was secondarily overwritten by new mutations in ancestral Ectoreta. Likewise, we conclude that a single amino acid insertion in polyubiquitin of Cercozoa, Endomyxa and most Foraminifera (Bass et al. 2005) and some but not all Acantharea (Burki et al. 2010) must be an ancestral rhizarian character secondarily lost in some Radiozoa and at least one foraminiferan.

Though *Gromia* is largely filose, it exhibits occasional filopodial anastomoses and is thus weakly net-like, making it likely that the last common ancestor of *Gromia* and *Filoreta* was reticulose, but without granules—unlike the reticulose Foraminifera. Vampyrellidea (see later section) also often have non-granular reticulopodia, so we may infer that the common ancestor of Endomyxa and classical Retaria was reticulose, most likely without granules. This makes their increasingly strongly supported grouping as a clade on multiprotein trees morphologically reasonable. By contrast, former cercozoan subphylum Filosa are so predominantly filose, and their filopodia so rarely anastomosing, that their last common ancestor was almost certainly non-reticulose. It was clearly trophically a flagellate with posterior ciliary gliding, whereas Endomyxa—like Ectoreta (classical Retaria)—are never trophically flagellates. Cavalier-Smith (2018) therefore formally transferred Endomyxa from Cercozoa to Retaria, broadening the circumscription of Retaria to include Endomyxa as well as Foraminifera and Radiozoa, which narrowed the circumscription of Cercozoa to apply to former Filosa only (Table 1), a circumscription informally used by Burki et al. (2010) and Pawlowski and Burki (2009). That made the rhizarian phyla exactly reflect the basal split confirmed here, dividing Rhizaria into ancestrally filose, gliding cercozoan bicilates on the one hand and trophically non-ciliate reticulose Retaria on the other hand. Cercozoa and Retaria as thus emended constitute two distinctly divergent body plans that occupy substantially different adaptive zones, both ancestrally strongly associated with solid surfaces. Etymologically, Retaria (from Gk *rete* net) applies equally well to Endomyxa and classical Retaria. Cavalier-Smith (2018) therefore abandoned Filosa as a taxon name as now synonymous with Cercozoa; if we regard the protruding posterior cilium of gliding Cercozoa as a tail, Cercozoa (from Gk *kerkos* tail) is descriptively more apposite for a higher proportion of the phylum than was Filosa—that change also eliminated all risks of confusion with filose amoebae that are neither Cercozoa nor chromists but belong to three different scotokaryote phyla (e.g. *Nuclearia*, *Fonticula*, *Rigifila*, *Sapocribrum*).

**Fig. 6:**
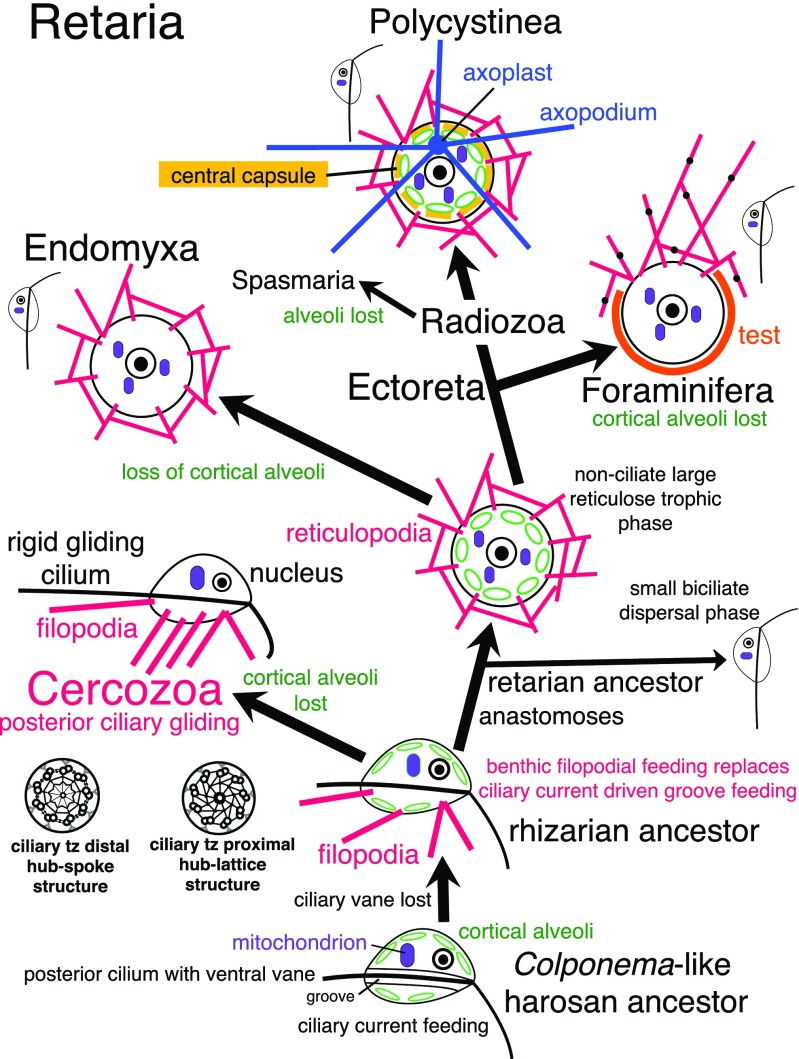
Body plan evolution in Rhizaria. The primary step in the evolution of Rhizaria from a *Colponema*-like harosan biciliate ancestor having cortical alveoli was argued to be a switch from planktonic to benthic feeding (Cavalier-Smith 2018) through origin of actin-supported filopodia for catching food instead of using ciliary currents to direct suspended prey into the feeding groove. This entailed de-emphasis of the ancestral excavate feeding groove, posterior ciliary vane loss, novel transition zone structures and subsequent divergence in two directions—yielding Cercozoa by evolving ciliary gliding and losing cortical alveoli; and Retaria by evolving larger cell size and filopodial anastomoses to form a non-ciliated trophic network of feeding reticulopodia and restriction of cilia to transient small-celled non-feeding gametes or zoospores. Early on, Retaria diverged into Endomyxa by losing cortical alveoli and Ectoreta by evolving new skeletons and much larger long-lived trophic cells that typically reproduce by multiple fission. Ectoreta probably first split to form the planktonic self-rowing *Sticholonche* that lost reticulopodia (not figured) and a reticulopodial ancestor of ancestrally benthic Foraminifera, emphasising feeding by granular reticulopodia supported by irregular microtubules and an extracellular test (and losing cortical alveoli), and planktonic Radiozoa with radiating food-trapping axopodia supported by geometrically regular microtubular axonemes. Radiozoa evolved two contrasting body plans: Polycystinea added dense material to the outer surface of cortical alveoli to form perforated central capsules separating the reticulose ectoplasm from the nucleus/mitochondria-containing endoplasm with its microtubule-nucleating axoplast (= centrosome) and evolved a radiating silica endoskeleton (for clarity not shown); their sisters (Acantharia, not figured) like Foraminifera lost alveoli but uniquely evolved a strontium sulphate endoskeleton associated with contractile myonemes and multiplied axoplasts (see text)

Recent multiprotein trees including ascetosporans and the vampyrellid *Leptomyxa* strongly confirm that *Gromia* is not specifically related to Foraminifera and that Ectoreta is a maximally supported clade (Sierra et al. 2016). Unity of Ectoreta was only tardily appreciated (Cavalier-Smith 1999) because of earlier overemphasis on the radically different skeletons of their four major subgroups. In Polycystinea, ectoplasm and endoplasm are most sharply segregated by a very thick, dense intracellular central capsule, which has pores or slits, through which axonemes pass from intracapsulum to extracapsulum in subgroups with axoplasts inside the central capsule (in some, they are nucleated outside it: Cachon et al. 1990). Hollande et al. (1970) argued that ultrastructural similarities between the polycystine central capsule and the dinoflagellate cortex suggest these two groups may be related. It is now clear that they are not sisters, though both belong to Harosa, which arguably ancestrally had cortical alveoli if those of alveolates, raphidophyte heterokonts and glaucophytes stem from a common corticate ancestor (Cavalier-Smith et al. 2015a; see also discussion below on rhizarian origins). Central capsular dense material is not topologically homologous to armoured dinoflagellate dense plates as it lies outside not inside alveoli, but it remains plausible that polycystine inner alveoli that underly the central capsule evolved from ancestral corticate alveoli (Fig. 6). Such continuity would entail four independent alveolar losses in Rhizaria—by Cercozoa, Endomyxa, Acantharia and Foraminifera (Fig. 6), fewer than needed within Heterokonta or Hacrobia (Cavalier-Smith et al. 2015a), but more than in Plantae (just one). There is no reason to think that cortical alveoli have any relationship to the extracellular test of Foraminifera; extracellular capsule of Acantharia; or the ‘central capsule’ of Phaeodaria, marine axopodial non-ciliate protists now a cercozoan subclass of Thecofilosea (Cavalier-Smith and Chao 2012a). Cercozoan Phaeodaria (e.g. *Aulacantha*) differ from Radiozoa in lacking cross links between their axonemal microtubules; in having only three large apertures in their central capsule; in species with mineralised skeleton in it being amorphous silica mixed with organic material but without any strontium sulphate; and in having specialised digestive vacuoles (phaeodia) in the endoplasm. Thus similarities between these two groups of radiolaria are convergent results of independently adopting a similar planktonic, axopodial feeding mode that differs from the putatively ancestral benthic rhizarian way of life.

Given that most genes were missing from the rhizarian alignment of Sierra et al. (2016) and the likelihood that their CAT analysis did not converge (discussed in a later section), their trees are not good evidence that Radiozoa are paraphyletic. All but two of our trees (Figs. S5 and S6) show Radiozoa as holophyletic, adding a fifth (nassellarian) radiozoan made Radiozoa consistently a clade (Krabberød et al. 2017).

### Rare ribosomal protein insertions distinguish Cercozoa and Retaria

**Fig. 7:**
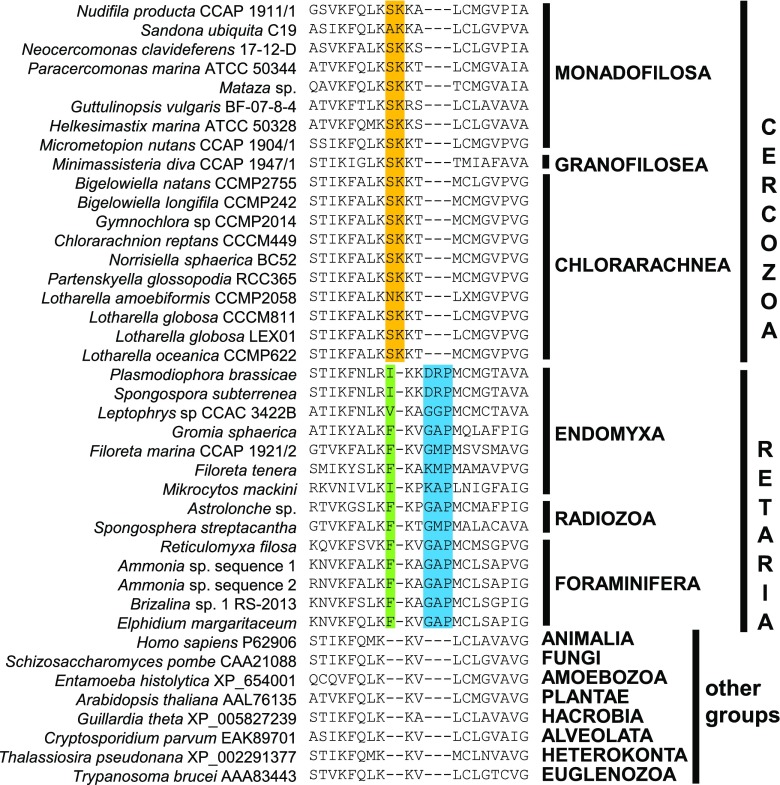
Dissimilar phylum-specific insertions in ribosomal protein L1 of rhizarian phyla Cercozoa and Retaria. All Cercozoa have homologous two-amino-acid insertions, whereas all Retaria have two separate non-homologous insertions (of one and three amino acids) at positions two amino acids apart. Sequences from GenBank or found by nucleotide BLAST against our and other transcriptomes or genomes and translation. Sequences for *Guillardia* and chlorarachneans are for cytoplasmic ribosomes and nuclear-coded—that for *Guillardia* in Burki et al. (2010) was misleadingly the periplastid nucleomorph-coded version and thus red algal in origin. In Burki et al. (2010) Fig. 8B, this region was misaligned and their transcriptome-derived ‘*Reticulomyxa*’ sequence was actually from a cercozoan contaminant (see text); that shown here is from the genome and has both authentic retarian signatures and thus likely genuinely from *Reticulomyxa*

Burki et al. (2010) thought Rhizaria are characterised by a unique two or four amino acid insertion in ribosomal protein L1 (rpl1 for which they used the obsolete human name L10a). Our new data show there is no rhizarian insertion, but separate non-homologous insertions in Cercozoa and Retaria (Fig. 7). Cercozoa have a two-amino-acid insertion (serine, lysine—SK; less often asparagine, lysine—NK), whereas Retaria have a single inserted phenyalanine (F) at this position (or rarely isoleucine—I or valine—V) and a separate three-amino-acid insertion (GAP or sometimes GMP, GGP, KAP, KMP or DRP) two amino acids downstream. These contrasting insertion patterns strongly confirm that Retaria and Cercozoa as now constituted are both clades, but as no data exist for the deepest branching skiomonads and novel clades 10 and 12, the possibility that Cercozoa are paraphyletic (weakly hinted in Fig. S8) is not yet excluded, but Retaria must be a clade.

Krabberød et al. (2017) stated that not all Rhizaria have an insertion in this region, but gave only *Minchinia* as an exception. However, as discovered here, many ‘*Minchinia*’ sequences are contaminants. Of the four rpl1 sequences we extracted from the transcriptome, one is from the green alga *Micromonas* (by rpl1 tree, not shown, which includes 19 Rhizaria), and three are from the same diatom (by sequence signatures; one does not include the insertion region); thus none is actually rhizarian, so no exceptions to our rule. Furthermore, the transcriptome-derived ‘*Reticulomyxa*’ rpl1 (Burki et al. 2010, Fig. 8B) has the cercozoan single insertion NK and groups with the cercozoan *Mataza* on our rpl1 tree, not with the significantly supported retarian subclade. Thus that ‘*Reticulomyxa* ‘rpl1 sequence is from a cercozoan contaminant, not a foraminiferan. To verify this, we deduced the authentic *Reticulomyxa* Rpl1 sequence from the *Reticulomyxa* genome (Glöckner et al. 2014), unavailable when we ran our trees, which as predicted has the retarian signatures F and GAP like all other Foraminifera. Unfortunately, we only discovered this contamination after we did our trees (which like other published ones therefore included the wrong sequence). These complications illustrate pitfalls of deducing insertion signatures from too sparse contaminated data (the Burki et al. rpl1 tree had only four sequences, which unlike ours did not even form a rhizarian clade). Likelihood of contamination in protist samples (cultured and especially uncultured) has been underestimated.

### Phylum Retaria comprises morphologically contrasting subphyla Endomyxa and Ectoreta

A 229-protein harosan tree including three ascetosporans and a vampyrellid (Sierra et al. 2016) shows Retaria sensu Cavalier-Smith (2018) as a clade with maximal or near maximal support by five methods, fully corroborating our conclusion of the holophyly of Retaria as now delimited. Our and previous multigene trees all show strongly that Radiozoa plus Foraminifera are a clade. Cavalier-Smith (2018) established new subphylum Ectoreta for this extremely robust clade to differentiate it from subphylum Endomyxa, necessary when Endomyxa were recognised as Retaria. The name Ectoreta stressed one of two key aspects of their shared body plan—an unusual subdivision of their cells into two contrasting regions: (1) an inner endoplasm (intracapsulum) containing the nucleus, mitochondria, Golgi apparatus and endoplasmic reticulum, and (in Radiozoa) one or more centrosome-like axoplasts that nucleate the axopodial axonemes; (2) an outer ectoplasm (extracapsulum) consisting essentially of reticulopodia and in Radiozoa also of the outer parts of the axopodial axonemes (Fig. 6). The second key shared feature is their much larger trophic cells than Endomyxa—or any Cercozoa except Phaeodaria. Non-ciliated trophic cells can often grow manyfold while division is suppressed (either multinucleate or with giant polyploid nuclei) and eventually (after weeks or months) undergo rapid multiple fission to produce much smaller biciliate gametes (Foraminifera) or zoospores (Radiozoa) with tiny nuclei with highly compressed chromatin. Such elaborate multiple fission life cycles evolved independently in several K-selected protist groups (Cavalier-Smith 1980a) and in Rhizaria independently in Ectoreta and Phaeodaria. Craigie and Cavalier-Smith (1982) explained how temporary division suppression modifies cell cycles to cause indefinite growth and multiple fission into numerous daughter cells in numbers proportional to the growth factor. This novel cell structure, life cycle and very large cells that they facilitate enabled Ectoreta to become dominant marine benthic protists. Independent evolution of silicified skeletons in Polycystinea and calcareous ones in Foraminifera gave the best fossil record of any rhizaria.

### Retarian subphylum Endomyxa is probably a clade

In contrast to Sierra et al. (2013) and CAT trees of Burki et al. (2010), our multiprotein trees mostly show Endomyxa as holophyletic (Figs. 1, 2, 4 and S1–S3), most strongly supported on Fig. 4. This is likely to be correct as site-heterogeneous trees including Ascetosporea and a vampyrellid also weakly show endomyxan holophyly (Sierra et al. 2016), despite their less reliable site-homogeneous trees often putting Marimyxia as sister to Ectoreta with Proteomyxia one node lower. Vampyrellids and *Filoreta* were formerly (Cavalier-Smith and Chao 2003a; Bass et al. 2009a) put in Lankester’s ancient class Proteomyxidea (Lankester 1885), a polyphyletic assemblage including *Pseudospora*, a confused genus that may not even be cercozoan (Hess and Melkonian 2013). In view of repeated non-grouping of vampyrellids and *Filoreta* on rDNA trees, Ruggiero et al. (2015) abandoned class Proteomyxidea. Hess et al. (2012) treated Vampyrellida as an order (but as Vampyrellida of West (1901) had no diagnosis it is invalid, so Table 2 validates it using the revised diagnosis of Hess et al.), as sole members of a class ‘Vampyrellidea’—as no diagnosis was given, Cavalier-Smith (2018) validated class Vampyrellidea. Exclusion of Pseudosporida from Endomyxa means that Vampyrellidea are exclusively non-ciliate, so presumably lost cilia (? also sex) after diverging from Phytomyxea, now their firmly established sisters.

As *Filoreta* are reticulose, and *Gromia* capable of rare pseudopodial fusion, and each of the three major vampyrellid clades (A-C in Berney et al. 2013) contains some reticulose amoebae, Endomyxa were probably ancestrally reticulose marine protists; if so, non-reticulose Vampyrellida became secondarily filose. However, as vampyrellid clades A (Vampyrellidae, Leptophryidae) and B (*Thalassomyxa*) both have some purely filose amoebae we cannot exclude the possibility that Endomyxa were ancestrally filose and secondary anastomoses evolved reticulose lineages polyphyletically. Reticulose morphotypes evolved independently of Endomyxa and Retaria at least seven times (Berney et al. 2015 Table 1)—in haptophytes (*Reticulosphaera*), heterokonts (*Leukarachnion*), Amoebozoa (independently in *Stereomyxa, Leptomyxa* and Variosea: Cavalier-Smith et al. 2016), Cercozoa (independently in Granofilosea and Chlorarachnea—both Reticulofilosa)—so multiple origins of filopodial anastomosis may be almost as easy evolutionarily as multiple losses of filopodial fusibility. We cannot use the reticulose nature of Ectoreta to argue for a specific relationship to either Gromiidea or Endomyxa. However, the most reliable site-heterogeneous multiprotein trees now make it reasonably certain that Ectoreta are indeed related to Endomyxa (as independently do the rpl1 insertions). They also clearly show that Ectoreta are not sisters of Gromiidea; site-heterogeneous trees weakly support Endomyxa being a clade and sister (not ancestral) to Ectoreta. The earlier branching of Phytomyxea on homogeneous trees (sometimes with as little as 63% support: Sierra et al. 2016) is likely an artefact of algorithms with false substitution models.

A 255-protein tree (Krabberød et al. 2017) representing Endomyxa only by *Gromia* and *Filoreta tenera* showed them as a maximally supported clade excluded both from Ectoreta and Cercozoa (as now revised)—both maximally supported. Adding here a genically better sampled *Filoreta marina* to multiprotein analyses that include also Phytomyxea and many more Cercozoa consistently gives 187-protein trees that agree with the 255-gene trees in Ectoreta and Cercozoa sensu stricto both being maximally supported clades, from which Endomyxa (usually a clade) are always maximally excluded. Thus completely ignoring subphylum Endomyxa and dispersing it as five unrelated groups within Cercozoa (Adl et al. 2012) was taxonomically unsound.

### Phylogeny of subphylum Endomyxa

Table 1 follows Ruggiero et al. (2015) in grouping the naked reticulose order Reticulosida (*Filoreta*) with Gromiida in class Gromiidea. Ribosomal DNA trees group *Filoreta*, *Gromia* and the animal-parasitic Ascetosporea together as a marine clade (Bass et al. 2009a; Hess et al. 2012; Berney et al. 2013). By contrast, the invariably parasitic Phytomyxea (terrestrial plant parasitic plasmodiophorids and often marine parasitic phagomyxids) and free-living marine and freshwater Vampyrellida group together by rDNA with moderate to strong support (Figs. S6–S9 and Bass et al. 2009a; Hess et al. 2012; Berney et al. 2013; Gong et al. 2015). Our well-converged Rhizaria-only 187-protein trees (Figs. S5 and S6) also strongly grouped *Filoreta* and *Gromia* by CAT (0.97, 0.95), but overall half our CAT trees (72, 74, 158, 159 taxa) weakly grouped *Gromia* with plasmodiophorids instead. Above, we attributed this contradictory alternative to long-branch attraction, but it may be exacerbated by sparse endomyxan and gene sampling.

Our analyses excluded the acestosporean haplosporidian-relative *Mikrocytos* as its proteins have evolved ultrarapidly (like those of microsporidia) and its branch on a 119-gene tree using 23,162 amino acids (Burki et al. 2013) was 20 times longer than its sister *Filoreta tenera* (there wrongly called *Corallomyxa* sp.), making it possible that its presence would have artefactually distorted our rhizarian trees. We suspect that this ultra-long branch, coupled with sparse gene sampling of plasmodiophorids, is why the latter did not group with other Endomyxa on that tree. Though *Mikrocytos* was sister to *Filoreta* on the CAT tree and this clade sister to *Gromia*, neither clade was found by ML (Burki et al. 2013), which is more sensitive to long-branch artefacts. The grouping of *Mikrocytos*, *Filoreta* and *Gromia* is probably correct as Ascetosporea group strongly with *Gromia* and *Filoreta* as a marine subclade on rRNA trees with much less severe long-branch problems (Fig. S8–S11; Bass et al. 2009a) as they do with variable support on the recent 229-protein 56-corticate tree (Sierra et al. 2016). However, rRNA shows very strongly that Ascetosporea are sisters of *Gromia* not *Filoreta*, which is one node lower (Figs. S6–9: Bass et al. 2009a; Hess et al. 2012; Berney et al. 2013). Multiprotein trees with two shorter-branch ascetosporans (both a 56 taxon tree including *Mikrocytos* and a 55-taxon one excluding it) and the vampyrellid *Leptomyxa* (Sierra et al. 2016) contradictorily consistently grouped *Gromia* and the genically sparsely represented *Filoreta tenera* (improperly labelled only *Filoreta* sp.) as a clade on site-homogeneous trees, but their site-heterogeneous trees show even more strongly than ours that Gromiidea are paraphyletic, not a clade as they assumed because of their systematically contradictory ML trees (Sierra et al. 2016: Fig. S3, 0.98; Fig. S8, 0.97). To test that further, we need richer gene and taxon sampling for Endomyxa, especially for Vampyrellida and short-branch Ascetosporea; the 56-taxon tree of Sierra et al. had 45% missing data on average and far more missing for Retaria (up to 85% missing for some genes and often 80–96% missing for Retaria).

The wisdom of excluding *Mikrocytos* is shown by the site-heterogeneous tree of Sierra et al. (2016) (Fig. 3) not grouping *Mikrocytos* at all with the other two Ascetosporea but placing it deeper than them plus Gromiidea, even though Ascetosporea were a clade on all homogeneous trees. Furthermore, presence of *Mikrocytos* changed the topology of endomyxan branches and probably prevented chain convergence (we infer that from two nodes having exactly 0.5 support, they gave no maxdiffs) even though its branch was interestingly not as disproportionately long as in Burki et al. (2013) perhaps because more genes and other haplosporidians were included. In all the Sierra et al. (2016) trees, *Minchinia* was sister to the haplosporidian *Bonamia* with 83–100% support, despite having 96% missing data, indicating that at least some of the 27 genes they included were actually haplosporidian. However, several of them might have been diatom or green algal contaminants according to our single-gene trees, so were not amongst the only 14 we included. Sierra kindly sent us nine sequences they included as having a rhizarian signal (using BLAST against their database). For Hsp90 in particular their sequence appears to be a discontinuous in silico chimaera of four segments, one corresponding to a sequence that we retrieved but excluded as it is 99% identical to that of the green alga *Micromonas commoda*, two probably being from *Minchinia*, the fourth from a diatom/heterokont relative; we included no ‘*Minchinia*’ Hsp90. Their gene selection protocol was not exactly the same as ours, so if both authentic and contaminant versions were present in the original data, the same gene name from the same transcriptome need not represent the same sequence; neither their nor our protocols could have eliminated all contaminants. Because the ‘*Minchinia*’ genes in their alignment only partially overlap with those in ours, it is unsurprising that ‘*Minchinia*’ went to a different place in their and our analyses. Because unlike us they included *Mikrocytos* (52% missing genes) and *Bonamia* (only 46% missing), their far more numerous haplosporidian genes would have completely overridden the paltry sample for ‘*Minchinia*’ and therefore enable Ascetosporea to be correctly placed within Endomyxa even if some of their ‘*Minchinia*’ genes were non-rhizarian contaminants or chimaeras of *Minchinia* plus contaminant segments as is the case for Hsp90. Without the two other haplosporidian taxa, even a few non-harosan contaminant sequences could have caused ‘*Minchinia*’ to have been misplaced in comparison with the Sierra et al. results (probably more reliable in this respect).

If the likely Ascetosporea/*Gromia* relationship is confirmed, Gromiidea would be the paraphyletic free-living ancestors of parasitic Ascetosporea. As no ciliate phase is known in *Filoreta* or Ascetosporea, they presumably lost cilia independently, after diverging from *Gromia*. Though exclusively marine, Gromiidea are an ancestral group (Cavalier-Smith 2010b) not a clade, but the only two orders (predominantly filose testate Gromiida, and naked reticulose Reticulosida) are not sufficiently different phenotypically to merit separate classes so for simplicity it is best to include both in Gromiidea, as did Ruggiero et al. (2015). The new ‘clade’ name Apofilosa (Sierra et al. 2016) was premature (their appearing as a ‘clade’ was probably an artefact of the evolutionarily inaccurate site-homogeneous models used for the analyses); unnecessary (as Gromiidea already existed for this group); and descriptively wrong (they do not lack thread-like pseudopodia).

If our Rhizaria-only endomyxan topology (*Gromia*/*Filoreta* an apparent clade because of absence of Ascetosporea) is correct, Ascetosporea and Phytomyxea became parasites independently, which Sierra et al. (2016) also conclude from multiprotein trees including three Ascetosporea. Though Plasmodiophorida are exclusively parasites of terrrestrial plants, their sister order Phagomyxida parasitises marine green and brown algae (Maier et al. 2000; Bulman et al. 2001), and the gromiidean sisters of Phytomyxea are all marine, so like Ascetosporea ancestral phytomyxids were probably marine parasites. The non-grouping of Phytomyxea and Gromiidea on a less well-sampled tree (Sierra et al. 2013), where Phytomyxea wrongly branched more deeply than other Rhizaria, and their deep separation on our trees are both consistent with rDNA trees showing clades Vampyrellida/Phytomyxea and Gromiidea/Ascetosporea diverging immediately after the monophyletic origin of Endomyxa (Figs. S6–S9; Bass et al. 2009a; Hess et al. 2012; Berney et al. 2013). Taxonomically comprehensive 229-gene trees (Sierra et al. 2016) confirm the early divergence of these two maximally supported major endomyxan clades (Vampyrellida/Phytomyxea and Gromiidea/Ascetosporea). Cavalier-Smith (2018) established superclasses Marimyxia and Proteomyxia respectively for these robust clades (Table 1). Sierra et al. (2016) label the Proteomyxia clade Phytorhiza, an unnecessary new name, but did not establish a taxon.

### Diversity of Cercozoa sensu stricto; multiple character losses hide a basic unity

Arguably, the ancestral (filosan) cercozoan (see below) was a biciliate that during its trophic phase glided on its posterior cilium, at least, and did not have reticulopodia (it might even have had no pseudopodia). By contrast, ciliary gliding is unknown in any Retaria, whose trophic state is always rhizopodial, never flagellate as it was ancestrally for Cercozoa as here revised. Of the 32 orders of Cercozoa recognised here (Table 1), 17 are partly or entirely amoeboid, usually filose; and 15 have a predominant posterior ciliary gliding phenotype, only 1 of which also simultaneously glides on its anterior cilium (Tremulida, the most divergent on rDNA trees, but no transcriptome available to test this). Of these orders, nine include both phenotypes, usually within a species, mostly filopodial; only five are non-amoeboid gliding flagellates. *Reticulamoeba* in Leucodictyida is unusual in being reticulose and in its ciliated phase being able to glide or swim (Bass et al. 2012). An 11th order (Helkesida) has a mixture of non-amoeboid gliders and non-ciliate amoebae. The uncharacterised deepest-branching rhizarian lineages (novel clades 10, 12: Figs. S8–11), previously assumed to be Cercozoa (Howe et al. 2011a), could also include posteriorly gliding biciliates they do: discovery of swimming and occasionally gliding biciliate Aquavalon, with clear proximal ciliary transition zone hub-lattice (Bass et al. 2018), corroborates our thesis that ciliary gliding evolved in ancestral Cercozoa. but even our outgroup-rooted 416-taxon CAT trees could not establish whether their joint clade is sister to Cercozoa, Retaria or both together (see Fig. S8 legend).

Non-gliding cercozoan phenotypes are more diverse. Eleven orders are non-flagellates, 6 purely filose amoebae (Euglyphida, Zoelucasida, Perlofilida, Tectofilosida, Cryptofilida, Krakenida) and 4 axopodial (Eodarida, Opaloconchida, Desmothoracida, Axomonadida) and are so thoroughly scattered on rDNA trees (only 5 currently represented on rDNA trees) and so morphologically varied in their affinities that they must represent about nine independent losses of cilia and gliding. Only seven are non-gliding flagellates in the trophic phase, one axopodial (Axomonadida), two pseudopodial (Limnofilida filose; Chlorarachnida filose or meroplasmodial, though *Bigelowiella* is secondarily a planktonic non-amoeboid swimmer) and surface-associated like ancestral Cercozoa as is the reticulose or filose Leucodictyida whose ciliated phase may be purely a swimmer (e.g. the filose *Massisteria*, *Minimassisteria*) or can swim or glide (*Reticulamoeba*). All sequenced non-gliding flagellate orders branch in different places on 18S rDNA trees so lost gliding independently. As only three sequenced orders are non-pseudopodial non-gliders, Ebriida being (‘drunken’) swimmers, the other two (Spongomonadida, Matazida) being predominantly surface-associated, loss of both gliding and of filopodia is rarer than just losing one. Thus phenotypically Cercozoa (= former Filosa) are essentially posterior gliding flagellates and/or filose amoebae or axopodial protists that lack centrosomes (unlike axopodial Hacrobia) or central capsules (unlike Radiozoa) and thus surface-associated rather than swimming protists. Only *Kraken* and the derived Leucodictyida represented in our trees by *Minimassisteria* have genuinely reticulose species (e.g. *Reticulamoeba*: Bass et al. 2012); both clearly became so independently of Ectoreta and Endomyxa, as some Leucodictyida are granulofilose; other orders of Granofilosea are exclusively granulofilose amoebae. Thus the basal rhizarian bifurcation on our multigene trees corresponds with an adaptive morphological bifurcation between posterior gliding cercozoan flagellates with strong filose amoeboid tendencies whilst feeding and Retaria whose trophic body plan is essentially net-like and non-ciliate, cilia growing only in dispersal phases or gametes (Phytomyxea and *Gromia* only within Endomyxa).

### Granofilosea probably diverged before Chlorarachnea

Previously, site-homogeneous 18S rDNA trees did not resolve the relative branching order of these classes, yielding three contradictory results: (1) they grouped as sisters (e.g. Cavalier-Smith and Chao 2003a), (2) Chlorarachnea diverged earlier (e.g. Howe et al. 2011a) or (3) later (e.g. Bass et al. 2012). Sometimes, apparently early diverging Chlorarachnea grouped with some or all metromonads (e.g. Cavalier-Smith et al. 2008; Bass et al. 2009a), but on later, more comprehensive trees Metromonadea were sister clade to other Monadofilosa (Howe et al. 2011a; Bass et al. 2012). In most cases, there was no significant support for these conflicting positions, though support for Granofilosea being deepest was moderate to good in Bass et al. (2012). Our three new, extremely taxon-rich, 18S rDNA trees, the first site-heterogeneous analyses to include basal Cercozoa, all also show Granofilosea as the deeper branch with moderate support; they show *Metopion* as strongly sister to other Monadofilosa but *Micrometopion* and *Metromonas* as sisters to Chlorarachnea with insignificant support (Figs. S8–11).

Our most convincing well-converged multigene CAT tree (Fig. 2) shows Granofilosea as the deepest branch followed by Chlorarachnea, with *Micrometopion* sister to other Monadofilosa, as also did Fig. S1. Even when *Rhogostoma*, supposed ‘Minchinia’, and *Microheliella* were added (Fig. 3), this relative branching order was also seen, with much weaker support. This topology is probably correct, but as ML gave partly discordant results, we recommend more extensive taxon sampling of these three classes and a *Tremula* transcriptome to check basal cercozoan branching order.

The position of *Limnofila* is particularly unstable. On ML trees, it groups with *Paracercomonas* with insignificant support, as it does with moderate support on CAT trees restricted to Rhizaria. We suspect this position is a tree reconstruction artefact stemming from *Limnofila* having nearly 90% missing data, perhaps exacerbated by contamination by a paracercomonad. Burki et al. (2007) who obtained the partial transcriptome of *Limnofila borokensis* (under the incorrect name *Gymnophrys cometa*) implied that the sequenced culture was the same one from A. P. Mylnikov (Borok, Russia) whose 18S rDNA was sequenced by Nikolaev et al. (2003). Mylnikov gave us also apparently the same culture for 18S rDNA sequencing; our 18S rDNA amplifications and cloning yielded a considerable variety of other protist sequences evidently present in that culture before we eventually got the authentic *Limnofila* sequence (Cavalier-Smith and Chao 2003a). Thus when we extracted DNA from this Mylnikov culture, it clearly had several different contaminants. Our 18S rDNA sequence differed only slightly from that of Nikolaev et al. (2003); both were included in Fig. 2 of Bass et al. (2009a) who formally described this species and attributed the differences to sequencing errors, not to the cultures being different. Bass et al. (2009a) explained that our Mylnikov-derived *Limnofila borokensis* type strain we submitted to ATCC had a visible *Filamoeba* contaminant (a non-ciliate amoebozoan) and raised the possibility that the rare flagellates also present in it (as Mikrjukov and Mylnikov (1998) who misidentified it as *G*. *cometa* originally noted) might be contaminants not a *Limnofila* life cycle phase as Mikrjukov and Mylnikov (1998) had assumed.

Bass et al. (2009a) stressed that caution is necessary in interpreting the *Limnofila borokensis* EST data, especially as no flagellate stage was detected in the other three *Limnofila* species, including *L*. *oxoniensis* that is so close to *L*. *borokensis* in morphology and rDNA sequence that it may be conspecific (if *L*. *borokensis* lacks a genuine flagellate stage as we suspect). Very likely, when Burki et al. (2007) extracted their *Limnofila* RNA, their culture was also contaminated by other protists, including perhaps a paracercomonad that could have been mistaken for a hypothetical flagellate stage. If some paracercomonad genes are present, most could not have been reliably separated on our ML single-gene trees as basal cercozoan resolution is poor even on multigene ML trees and much worse on single-gene trees; paracercomonad contaminants would tend to pull *Limnofila* away from *Minimassisteria* towards *Paracercomonas*. This interpretation may also help explain contradictory positions of *Limnofila* on the two sparsely sampled trees of Sierra et al. (2013): their 36-gene tree grouped it with *Bigelowiella* as a reticulofilosan clade with no support, but with 109 genes (perhaps including proportionally more contaminating paracercomonad proteins) it grouped weakly with *Paracercomonas* and *‘Heteromita*’ (actually *Sandona campae*—see later section).

More transcriptomes from better purified cultures of other Granofilosea with more complete gene coverage are essential to check our conclusion that Granofilosea are probably sisters of Chlorarachnea plus Monadofilosa. Experience with the initially undetected *Minimassisteria*/*Oxnerella* mixture (Cavalier-Smith and Chao 2012a; Cavalier-Smith et al. 2015a) exemplifies how easily overlooked morphologically similar contaminants can be in protist cultures, as does our discovery that the transcriptome of the discosean amoeba *Stygamoeba regulata* (Grant and Katz 2014) was heavily contaminated with genes from another discosean, *Cunea* (Cavalier-Smith et al. 2016).

### Monadofilosan class Helkesea

Cercozoa are divided into eight classes (Cavalier-Smith 2018), three forming the putatively ancestral Reticulofilosa and five the putatively derived Monadofilosa (Cavalier-Smith 1997; Cavalier-Smith and Chao 2003a; Bass et al. 2009a; Cavalier-Smith and Karpov 2012b). Monadofilosa and Reticulofilosa are now ranked as subphyla, their original rank when clade Rhizaria was first established as a separate phylum under the old name Rhizopoda (Cavalier-Smith 1997). This subdivision into classes has been fairly robust to the discovery of novel Cercozoa, which simply slot into one or other of them (e.g. Hoppenrath and Leander 2006a, b; Howe et al. 2011a, b, Yabuki and Ishida 2011; Shiratori et al. 2012, 2014; Chantangsi and Leander 2010; Chantangsi et al. 2010; Bass et al. 2012; Vickerman et al. 2005). One exception to this has been the unusual aggregative non-ciliate amoeba *Guttulinopsis*, which Brown et al. (2012) showed to branch robustly within Cercozoa on a 159-protein tree, but included only three other Cercozoa making it impossible to assign it to a class though it was robustly sister to the only included monadofilosan, *Paracercomonas marina*. Burki et al. (2013, 2016) confirmed that *Guttulinopsis* was sister to *Paracercomonas marina* (wrongly named *Cercomonas longicauda* in their tree) when *Aulacantha* was included instead of *Limnofila*. A second group not confidently assigned to a class was Sainouroidea, a superfamily of zooflagellates (*Sainouron*, *Cholamonas* and *Helkesimastix*), whose 18S rDNA was so extremely divergent from other Cercozoa that trees could only assign it to Monadofilosa, not place it confidently in any class (Cavalier-Smith et al. 2008, 2009). Based on our present multiprotein trees and rDNA trees of Bass et al. (2016), Cavalier-Smith (2018) established new order Helkesida and class Helkesea to embrace guttulinopsid amoebae and classical sainouroid flagellates, subdividing them into two superorders (Table 1).

Our multiprotein trees invariably show the amoeba *Guttulinopsi*s and the essentially non-amoeboid sainouroid flagellate *Helkesimastix* as robust sisters, as independently convincingly shown by 18S rDNA trees in Bass et al. (2016, published after we completed our multigene analyses). Bass et al. (2016) show that Guttulinopsidae (*Guttulinopsis* and a closely related non-ciliate non-aggregating amoeba *Rosculus*) are a robust rDNA clade maximally supported as sister to *Helkesimastix*, i.e. more closely than to the also related Sainouridae (*Sainouron*, *Cholamonas*). Clearly, Guttulinopsidae evolved from sainouroids, whose ancestor had already lost the anterior cilium, by losing also the posterior one and becoming amoeboid. Later still *Guttulinopsis* alone evolved cell aggregation and a multicellular fruiting body, unlike any other Cercozoa. The guttulinopsid rDNA branch is over twice as long as for *Helkesimastix*; Brown et al. (2012) showed no 18S rDNA for assigning it to a class or order. Though most cercozoan amoebae are filose (rarely reticulose, e.g. *Reticulamoeba*: Bass et al. 2012, which also has a long-branch aberrant rDNA making it intractable for many standard protocols), the fact that those of *Guttulinopsis* and *Rosculus* are lobose (hence once wrongly put in Heterolobosea) is unusual but not the first cercozoan example: *Rhogostoma* (Howe et al. 2011a) and some cercomonads have lobose pseudopods (Bass et al. 2009b). Ultrastructurally, the broad flat mitochondrial cristae of Guttulinopsidae are indistinguishable from those of sainouroids, making it reasonable that they group together. This well-defined cercozoan subclade evolved flat cristae independently of Percolozoa. Guttulinopsidae differ from other Cercozoa in having unstacked Golgi cisternae; those of *Sainouron* are clearly stacked (Cavalier-Smith et al. 2008), whereas although *Helkesimastix* has a clearly defined Golgi region with at least one large Golgi cisterna, clear evidence of Golgi stacking was not seen. Possibly therefore *Helkesimastix* Golgi is unstacked, which would be unsurprising as *Helkesimastix* is more closely related to Guttulinopsidae than to *Sainouron* or *Cholamonas*, making the original superfamily Sainouroidea paraphyletic.

Though *Cholamonas* did not group with *Sainouron* and *Helkesimastix* on a homogeneous (MrBayes) 18S rDNA tree (Bass et al. 2016), it did so with maximal support in a taxonomically more strongly sampled one (Cavalier-Smith et al. 2009) and on the far more richly sampled site-heterogeneous Fig. S8, confirming superiority of site-heterogeneous analyses for difficult long-branch taxa (e.g. Cavalier-Smith 2015). Figure S8 suggests that Sainouridae may be paraphyletic but this could be an artefact if *Sainouron* with a longer branch than *Cholamonas* groups incorrectly with the ultralong-branch Helkesimastigoidea.

Our multiprotein CAT trees all confirm that helkesids are monadofilosans and that Monadofilosa are a robust clade. They also confirm the rDNA results that helkesids do not group with Sarcomonadea or Thecofilosea or *Micrometopion* (our sole representative of Metromonadea). Thus Helkesida are a genuinely distinct lineage that branches deeply in Monadofilosa immediately after divergence of *Micrometopion* but before the bushlike radiation of Ventrifilosa to which most known Cercozoa belong. Adl et al. (2012) wrongly excluded helkesids from Cercozoa, disregarding the evidence that they are monadofilosan Cercozoa (Cavalier-Smith et al. 2008, 2009); our multiprotein trees unambiguously correct that mistake.

The non-aggregative amoeba *Rosculus* that ultrastructrally resembles *Guttulinopsis* more than either do Heterolobosea (Brown et al. 2012) was clearly correctly classified in Guttulinopsidae: they group together strongly by rDNA (Bass et al. 2016). On Rhizaria-wide trees, Ventricleftida are a clade by CAT when Helkesida are excluded (0.51 Fig. S11); adding Helkesida places them within eVentri and pushes *Verrucomonas* away from other ventricleftids (Figs. S8 and S10), but Monadofilosa-only trees all show Ventricleftida as a well-supported clade with no tendency to group with helkesids (Figs. S12–15).

Helkesid 18S rDNA is extremely resistant to PCR amplification presumably because it is highly divergent (Cavalier-Smith et al. 2008, 2009); that of *Guttulinopsis* came not by PCR but from the transcriptome (Bass et al. 2016). Extreme rDNA sequence divergence explains why helkesids were absent from environmental surveys by Cercozoa-specific primers (Bass and Cavalier-Smith 2004). Bass et al. (2016) using helkesimastigoid-specific PCR primers for the 18S rDNA V6 region showed that helkesimastigoids are a highly diverse group of mostly coprophilic, sometimes marine Cercozoa.

Having established the basal branching order of Cercozoa more clearly, we can now identify the second amino acid insertion in polybiquitin (Bass et al. 2005; Chantangsi et al. 2010) as an ancestral shared character for all Cercozoa (except possibly Skiomonadea, where it is unsequenced) that was never present in Retaria, but was lost by Chlorarachnea and independently by *Helkesimastix* within Helkesida and the pansomonad *Agitata* (= *Cercobodo*) *agilis*. Presence of a double polyubiquitin amino acid insertion in *Ventrifissura* and *Verrucomonas* (Chantangsi et al. 2010), as in *Sainouron*, strengthens the inference that one amino acid was lost in *Helkesimastix*.

### Convergent body form evolution of Cercomonadidae and Paracercomonadida?

Cercomonads are distinguished from other gliding Cercozoa by their soft, highly deformable cells which are drawn out at the rear end of gliding cells as a tail that sticks to the posterior cilium and/or substratum. Originally, all such species were in the single genus *Cercomonas* (Dujardin 1841; Mylnikov and Karpov 2004). Ribosomal DNA trees suggested that *Cercomonas* may be paraphyletic ancestors of thaumatomonads and euglyphids (Cavalier-Smith and Chao 1997). After phylum Cercozoa was established (Cavalier-Smith 1998a,b), more extensive rDNA sequences showed it to be remarkably diverse and that cercomonads formed two distinct clades (A and B: Karpov et al. 2006) that sometimes grouped together (Bass et al. 2009b) but more often did not (Cavalier-Smith 2000b; Cavalier-Smith and Chao 2003a). rDNA sequencing for 51 Cercomonadidae and description of new species in both clades confirmed their phylogenetic distinctness and very deep divergence, so clade B was separated as new genus *Paracercomonas*, and a new genus *Eocercomonas* established for the then most divergent clade A lineage, which differs in cytoskeletal ultrastructure from typical *Cercomonas* (Karpov et al. 2006). Further new species descriptions and sequencing led to discovery of *Cavernomonas*, a posterior gliding biciliate with rigid cell surface and no posterior tail, which nonetheless grouped robustly in clade A, diverging near its base (Bass et al. 2009b). This immediately raised the question whether *Cavernomonas* had secondarily lost a soft body and tail or represents a rigid ancestral state for clade A. If clade A was ancestrally rigid with no tail, tailed clade A cercomonads (*Cercomonas*, *Eocercomonas*) must have evolved amoeboid feeding and cytoplasmic tails independently of *Paracercomonas*, i.e. the cercomonad phenotype would be polyphyletic and tails convergent.

This became increasingly likely when *Paracercomonas* ultrastructure showed simpler microtubular ciliary roots than clade A cercomonads (Bass et al. 2009b) and a different symmetry of the posterior roots supporting the tail relative to the posterior ciliary plane, as well as ciliary transition zone differences (Cavalier-Smith and Karpov 2012b). Cavalier-Smith and Karpov (2012b) placed *Paracercomonas* in a separate family and suborder (Paracercomonadina) from clade A cercomonads because of these substantial ultrastructural differences and argued that they best fit the idea that Paracercomonadina evolved tails independently of Cercomonadina. They also separated rigid *Cavernomonas* into new family Cavernomonadidae, restricting Cercomonadidae to the amoeboid, tailed Cercomonadina. Our multiprotein trees support the idea that thus revised Cercomonadidae and Paracercomonadina evolved protoplasmic tails independently, as *Neocercomonas clavideferens* never groups with *Paracercomonas marina* but always strongly with the non-amoeboid, untailed glissomonad *Sandona ubiquita*. Our multiprotein trees invariably group this *Neocercomonas*/glissomonad clade with the placonudan *Paulinella* and *Nudifila*, with *Paracercomonas* excluded from their joint clade with maximal or near maximal support.

We noted above that the *Limnofila* transcriptome probably had a paracercomonad contaminant whose overlooked genes may tend to pull *Limnofila* artefactually towards *Paracercomonas*; conversely, they would also tend to pull *Paracercomonas* away from *Neocercomonas*, but this effect would be weaker because the nearly three times as many *Paracercomonas* as *Limnofila* genes in the alignment would dominate *Paracercercomonas*’s position compared with a rare contaminant. In accordance with this prediction, our 30-taxon Rhizaria-only trees excluding *Limnofila* (Fig. S7) still place *Paracercomonas* more deeply than Pediglissa/*Nudifila*/*Paulinella*. Thus polyphyly of classical cercomonads on our multiprotein trees cannot be attributed to such a contamination artefact. Multiprotein analyses for more paracercomonads and cercomonads, and additional sarcomonad orders, are needed to confirm the deeper branching of paracercomonads as our three heterogeneous 18S rDNA trees without Krakenida group the long-branch paracercomonads strongly with cercomonads (Figs. S8, S10 and S11); only the one including both Krakenida and *Cholamonas* (Fig. S12) shows deeper-branching paracercomonads in conformity with multiprotein trees. Nonetheless, multiprotein evidence that tailed cercomonads are polyphyletic, as Cavalier-Smith and Karpov predicted, is strong enough for paracercomonads now to be made a separate order. Our most comprehensive 18S rDNA ML trees support this separation. As the *Neocercomonas*/glissomonad multiprotein clade is so robust, we also establish a new sarcomonad subclass Pediglissa grouping them together and place Paracercomonadida on its own in a separate subclass as it does not even group specifically with Pediglissa:

#### Diagnosis of new subclass Paracercomonadia and order Paracercomonadida

Cavalier-Smith: exactly as for suborder Paracercomonadina (Cavalier-Smith and Karpov 2012b p. 56–7).

#### Diagnosis of new sarcomonad subclass Pediglissa

Cavalier-Smith: soil or freshwater biciliate phagotrophic heterotrophic protists that glide on their posterior cilium and have a strong tendency to become amoeboid during feeding (unlike metromonads); pseudopodia much more often rounded lamellae than finger-like or filose (unlike Paracercomonadia); hub of transition zone lattice indistinct and often obscured by dense material (unlike helkesids and paracercomonads). Anterior cilium usually well developed (unlike Helkesida) and motile by undulating or semi-rigid oarlike beat, though sometimes short in glissomonads. Dorsal posterior centriolar microtubular root (dp1) absent, unlike paracercomonads. Trophic cells naked; lack theca (unlike Thecofilosea) or scales or perles (unlike many freshwater imbricates). **Etymology:** *pedon* Gk. soil, *glisser* F*.* to glide. Pediglissa include the majority of known cercozoan soil flagellates; all glide on a single posterior cilium only; none is known to be marine; the only known marine tailed cercozoan is the non-pediglissan *Paracercomonas marina* (Bass et al. 2009b). **Comment:** Includes the largely bacterivorous Cercomonadida, and classical glissomonads, plus pansomonads and the algivorous Viridiraptoridae. 18S rDNA trees clearly show that pansomonads and Viridiraptoridae are related to glissomonads, and close to cercomonads on trees but their relative branching order varies and needs to be established by multigene trees. Because the CAT model for more comprehensive rDNA trees groups viridiraptorids with Pansomonadida (Scoble and Cavalier-Smith 2014; see also Figs. S8–S15) not classical Glissomonadida as earlier by ML (Hess and Melkonian 2013), we do not accept the latter authors’ inclusion of viridiraptorids alone in glissomonads. Our taxonomically most comprehensive ML trees also group viridiraptorids with pansomonads within Glissomonadida, but without significant support (Figs. S14 and S15). Scoble and Cavalier-Smith (2014) noted that reducing pansomonads and viridiraptorids to suborders of Glissomonadida might be appropriate. Our most comprehensive site-heterogeneous 18S rDNA tree (Fig. S8; 467 rhizarian taxa) confirms fairly strongly (0.80) that Viridiraptoridae are sisters to clade Y (previously seemingly within glissomonads) and more weakly (0.64) that this joint clade is the sister of pansomonads not to any phenotypically characterised glissomonads. This clade is strongly supported by *Viridiraptor* having two prominent dense rhizoplasts anchoring each ciliated centriole (Hess and Melkonian 2014), exactly as in the amoeboid pansomonad *Aurigamonas* (Vickerman et al. 2005), which contradicts the corresponding ML tree (Fig. S9) that put pansomonads as sister to Glissomonadida plus Viridiraptoridae (as in Hess and Melkonian 2013). All our probably more accurate Monadofilosa-only trees show Viridiraptoridae as sister to clade Y and this clade as sister to classical pansomonads (Figs. S12–15: strong CAT, weak ML support). We therefore transfer Viridiraptoridae to pansomonads, now unified by this novel ultrastructural character absent from classical non-amoeboid glissomonads. As Fig. S12 also places pansomonads within Glissomonadida (albeit extremely weakly, as do all our rDNA trees: Figs. S8–S15), we reduce Pansomonadida in rank to a suborder of glissomonads (Table 1), and make new suborders Allapsina and Sandonina for classical essentially non-amoeboid glissomonads:

#### Diagnosis of new glissomonad suborder Pansomonadina

Vickerman ex Cavalier-Smith: biciliate gliding soil phagotrophs that (unlike Allapsina) typically form rounded lamellar pseudopodia that spread over surfaces and have two dense rhizoplasts associated orthogonally with each mature centriole; ciliary transition zone with distal dense plate; bacterivorous or algivorous; bacterivorous forms may have haptopodia; centrioles orthogonal or parallel.

#### Diagnosis of new glissomonad suborder Allapsina

Cavalier-Smith: usually non-amoeboid naked biciliate gliding soil flagellates with two orthogonal centrioles and ciliary transition zone having a distal dense transverse plate significantly above proximal hub-lattice; if slightly pseudopodial, pseudopods not lamellate; haptopodia, dense rhizoplasts and ciliary hairs absent. **Etymol:** *allapsus* L. gliding, contrasting their gliding not amoeboid trophic state. **Diagnosis of new glissomonad suborder** Sandonina Cavalier-Smith: biciliates ancestrally gliding on posterior cilium (motion jerky or jiggly or if smooth with rostrum), some slightly amoeboid; rarely (*Proleptomonas* only) non-gliding elongate swimmers with posterior cilium adherent to body and longer anterior cilium.

#### Comment:

Figure S8 suggests that Allapsina may be paraphyletic and thus the ancestral state for glissomonads, but support is too weak to exclude that they are a clade; to better define early glissomonad evolution, we need a comprehensive multigene tree and to determine the phenotype of DNA clades Y, Z and Q (Figs. S8–9; S12–S15).

The 36-gene tree of Sierra et al. (2013) tree was misleading in that the phaeodarian *Aulacantha* grouped strongly with ‘*Heteromita* sp.’; *Heteromita* is an old name formerly used for glissomonads, but is not valid for any Cercozoa as Howe et al. (2009) explained. Sierra et al. did not say where they got the ‘*Heteromita*’ sequences, but Sierra (personal communication) told us he obtained them from the then publicly available Camera database, now discontinued. As some of the ‘*Heteromita* sp.’ sequences are identical to those of Parfrey et al. (2010) from ATCC strain PRA-74 (there called *Heteromita globosa*), we presume that all are from the *Heteromita* sp. EST project for ATCC strain PRA 74 (http://gmod.mbl.edu/) but our request to Marine Biological Laboratory (MBL) at Woods Hole, MA to obtain these sequences for adding to our alignment (not on their website) received no answer. ATCC PRA-74 is actually *Sandona campae* (Howe et al. 2009). Currently, the original data for these sequences appear not to be in public databases, and therefore were not included in our trees nor those of Sierra et al. (2016). As their ‘*Heteromita*’ sequences were almost certainly from *Sandona campae*, they ought to have grouped with *Paracercomonas marina* (wrongly labelled *Cercomonas longicauda* in Sierra et al. 2013) as a sarcomonad clade in their 36-gene tree. Contradictorily, their 109-gene tree grouped ‘*Heteromita* sp.’ with an insignificantly supported *Limnofila*/*Paracercomonas* ‘clade’, and *Aulacantha* wrongly grouped with *Bigelowiella*, but none of its five branches were signifcantly resolved. If ‘*Heteromita* sp.’ is *S*. *campae*, it would be a close sister to *Sandona ubiquita* that we included using many more genes than the 28 Sierra et al. used for *S*. *campae*.

Though it is odd that such glissomonad sequences grouped so strongly with *Aulacantha*, that may be just because that Sierra et al. tree included only eight ‘*Heteromita*’ genes, several only partial, and only 12 from *Aulacantha,* with only two being the same as for ‘*Heteromita*’. Paring their 109-gene alignment down to only 36 genes (based on assumptions of what single-gene trees should show) probably removed too much useful data for accuracy, especially when taxon sampling was also very low. Our 187 single-gene trees also showed that several of their included ‘*Aulacantha*’ and ‘*Collozoum*’ genes probably came from non-rhizarian protists (Cavalier-Smith et al. 2015a) which also may have distorted their trees (see below); we excluded these from our alignments for a cleaner, more reliable analysis. Their criteria for recognising contaminants (using just BLAST, not our superior single-gene tree method) may not have been sufficiently stringent.

### Broader evolutionary significance of Pediglissa

If Harosa are ~ 750 My old (Cavalier-Smith 2013b; Cavalier-Smith et al. 2015a), the proportions of the Fig. 1 tree suggest that Pediglissa evolved ~ 330 My ago, reasonably consistent with previous estimates for the origin of glissomonads ~ 350 My based on rDNA trees (Howe et al. 2009, 2011b). Thus both estimates put the bush-like basal radiations of Pediglissa in the Carboniferous when land vegetation first became extremely rich and would have provided a huge infusion of soil organic matter stimulating growth of soil bacteria and enriching the adaptive zone for soil protist predators, of which Pediglissa are the most important and speciose amongst Cercozoa—and together with Amoebozoa the dominant protist soil predators. By similar reasoning from Fig. 1, paracercomonads diverged distinctly earlier, ~ 490 My ago (Late Ordovician). The first embryophyte land plants arose ~ 470 My ago (Gensel 2008), so paracercomonads probably arose as the first major group of gliding soil predators in the pre-forest Late Ordovician close to the transition time from terrrestrial green algae to the first liverwort-like lowly embryophytes when land vegetation was a mere algal crust. Later, Carboniferous forests and swamps stimulated the origins of Cercomonadida, glissomonads, pansomonads and alga-eating viridiraptorids. Thus, lush Carboniferous forests gave us not only our major coal supplies but the most numerous predators of bacteria on earth—pediglissans.

In marked contrast to paracercomonads, pseudopodia of *Cercomonas* and *Neocercomonas* (the most speciose Cercomonadidae) are rounded, broadly spreading lamellae closely similar to those of pansomonads, including Viridiraptoridae. Thus, it is possible that this is the ancestral state for Pediglissa. However, early branching Cercomonadida seldom have such broad pseudopodia; early branching *Cavernomonas* is non-amoeboid, *Filomonas* filose and *Eocercomonas* more often filose or finger-like than lamellate and thus more like paracercomonads and other ventrifilosan outgroups than like the *Cercomonas*/*Neocercomonas* clade. Classical glissomonads, here placed in new suborder Allapsina to contrast them with the lamellately pseudopodial pansomonads, are essentially non-amoeboid (Howe et al. 2011b). Some trees suggest that they may be paraphyletic and thus ancestral to pansomonads and viridiraptorids (e.g. Howe et al. 2009, 2011b), whereas others suggest they may be sister to pansomonads in the now broader sense including viridiraptorids (Scoble and Cavalier-Smith 2014). Overall, the slightly simpler scenario is that lamellar pseudopodia evolved independently in parallel in *Cercomonas*/*Neocercomonas* and pansomonad/viridiraptorid clades because of particular advantages of broad pseudopodia in moving over soil particles amongst narrow interstices and harvesting bacteria adhering to their surfaces. It is perhaps significant that the thecofilosean *Rhogostoma* common in soil independently evolved lamellar pseudopodia (Howe et al. 2011a) unlike its marine relatives. To clarify pediglissan evolution further, numerous deep-branching environmental DNA clades (Scoble and Cavalier-Smith 2014) need to be cultured to establish their phenotypes and multigene trees obtained for representatives of the earliest diverging lineages.

Previously, algivorous viridiraptorid-like flagellates were placed in order Pseudosporida (Cavalier-Smith 1993a) based on ultrastructure by Swale (1969). However, as Patterson and Zölffel (1991) implied and Hess and Melkonian (2013) detail, the concept of *Pseudospora* is historically very confused, lumping together such biciliate flagellates with uniciliates that may be closer to chytrid fungi. As the *Pseudospora* type species is uniciliate and almost certainly not a pediglissan, quite likely not even a cercozoan, we here exclude Pseudosporida from Cercozoa, leaving it incertae sedis as a potentially useful name for true uniciliate *Pseudospora* when properly characterised.

### Monophyly of Thecofilosea

Grouping of the phaeodarian *Aulacantha* with the thecate marine planktonic zooflagellate *Mataza* on our trees is consistent with segregation of thecate cercozoans without silica scales as class Thecofilosea (Cavalier-Smith and Chao 2003a) and with rDNA evidence that Phaeodaria belong in Thecofilosea and are not Retaria (Fig. S8 and Polet et al. 2004; Yuasa et al. 2006; Shiratori et al. 2014). Thecofilosean monophyly is weakly supported, probably because Matazida and Phaeodaria diverged very early in the Thecofilosea radiation and relatively few genes have been sequenced for the uncultured *Aulacantha*. This grouping is morphologically reasonable, unlike the grouping of *Aulacantha* and ‘*Heteromita*’ (Sierra et al. 2013) discussed above. We obtained a few protein genes by partial genome sequencing of the thecofilosean thecate amoeba *Rhogostoma minus*. Our chromist-only 74–75-taxon trees including only 897 *Rhogostoma* amino acids have so much missing data for it that we do not trust them, but allowing for these problems they are consistent with holophyly of Thecofilosea, which was weakly recovered on our well-converged Rhizaria-only 32-taxon CAT trees (Fig. S6).

In Sierra et al. (2016), *Aulacantha* was the deepest of all cercozoan (= filosan) branches, below *Bigelowiella* and in Sierra et al. (2016) and Burki et al. (2013) was sister to *Bigelowiella*. Both these contradictory positions are too deep and probably artefacts of the significant contamination of this transcriptome by non-rhizarian sequences that would have pulled them too low, as discovered by Cavalier-Smith et al. (2015a). In that study, we found that if we included all ‘*Aulacantha*’ genes of Sierra et al. (2013), *Aulacantha* wrongly branched between Filosa or Endomyxa, but when we removed contaminants identified by our single-gene trees, *Aulacantha* moved into Cercozoa and became sister of *Mataza*, this thecofilosean clade being usually sister to Sarcomonadea. *Aulacantha* should not be included in future multiprotein trees without removing these contaminants.

Our new site-heterogeneous 18S rDNA trees are the most comprehensive yet for Cercozoa and Endomyxa (Figs. S8 and S10) and the first to include both the long-branch basically uniciliate helkesids and the clearly biciliate, rigid bodied Ventricleftida. Previously, Ventricleftida were included in Thecofilosea because *Ventrifissura* grouped with them on a MrBayes 18S rDNA tree (Howe et al. 2011a) as did *Verrucomonas* on a sparser 18S rDNA tree (Chantangsi et al. 2010) and both *Ventrifissura* and *Verrucomonas* did so on a joint 18S/28S rDNA tree (Chantangsi et al. 2010). However, Ventricleftida were later put incertae sedis within Monadofilosa by Scoble and Cavalier-Smith (2014) whose 18S rDNA CAT-GTR PhyloBayes trees found no evidence for a grouping with Thecofilosea—or with Sarcomonadea as some MrBayes 18S rDNA trees suggested (Chantangsi et al. 2010, Fig. 2; Chantangsi and Leander 2010, Fig. 2). Bass et al. (2016) omitted Ventricleftida from their trees. Rhizaria-wide 18S rDNA analyses excluding Krakenia showed Helkesida within Ventricleftida, as sister to *Ventrifissura* and an array of environmental DNA lineages of unknown phenotype (group eSarcomonad Scoble and Cavalier-Smith 2014) both by PhyloBayes CAT-GTR (Figs. S8 and S10) and ML GTR (e.g. Fig. S9). However, the probably more reliable Monadofilosa-only trees including Krakenia never grouped them together but consistently put Ventricleftida as sister to Thecofilosea (Figs. S12 and S14) where they were previously classified. Ultrastructure is needed for ventricleftids to see if ciliary transition zone and roots are more similar to those of Helkesida than to Ventrifilosa or Metromonadea, whether cristae are flat as in *Sainouron* and *Helkesimastix* and *Kraken* or tubular as in most Cercozoa, and whether their cell surface is more like Helkesida, Metromonadea or Thecofilosea.

### Evolution of Sarcomonadea and imbricates

Class Sarcomonadea originally embraced posteriorly gliding biciliate flagellates, largely amoeboid whilst feeding, that are now assigned to four cercozoan orders: Paracercomonadida, Cercomonadida, Glissomonadida and Thaumatomonadida (Cavalier-Smith 1995, 1997), which 18S rDNA trees first showed were closely related to the non-flagellate euglyphid filose testate amoebae (Cavalier-Smith and Chao 1997). Even then, trees implied (more strongly on distance than ML trees) that sarcomonads were paraphyletic and ancestral to euglyphids that presumably evolved from them by evolving a test and losing cilia, and also showed that their joint clade was related first to Chlorarachnida and secondly to *Plasmodiophora*, providing the first strong evidence for the clade later called Rhizaria (Cavalier-Smith 2003b); the name Rhizaria when introduced slightly earlier (Cavalier-Smith 2002a) wrongly included the more distantly related Heliozoa as well as gliding flagellates now in phylum Sulcozoa, both being swiftly removed after their phylogenetic distinctiveness was better appreciated (Cavalier-Smith and Chao 2003a, b, c). After many more Cercozoa were added to trees, ML (but not distance) trees very weakly suggested that thaumatomonads and euglyphids might be more closely related to each other than to cercomonads (Cavalier-Smith and Chao 2003a). As thaumatomonads and euglyphids are both covered by silica scales, we postulated that scales originated in their common ancestor and established class Imbricatea to unite them, leaving non-scaly gliding amoeboflagellates only in Sarcomonadea (Cavalier-Smith and Chao 2003a). Unfortunately, we lack ultrastructure for Discomonadida, now in Placonuda and Imbricatea (Table 1) because of our site-heterogeneous 18S rDNA trees: do they have scales?

Subsequently, a large array of non-scale-bearing gliding amoeboflagellates of previously unclear affinities have been found by 18S rDNA to be related more closely to either thaumatomonads or euglyphids than to cercomonads and were thus added to Imbricatea (Hoppenrath and Leander 2006a,b; Chantangsi and Leander 2010; Chantangsi et al. 2010; Howe et al. 2011a; Shiratori et al. 2012, 2014; Yabuki and Ishida 2011), and are assigned to five distinct orders (Scoble and Cavalier-Smith 2014; Ruggiero et al. 2015). Spongomonadida are non-gliding relatives of thaumatomonads while Marimonadida, most Variglissida, Discocelida and Discomonadida are all gliders mostly closer to euglyphids than to thaumatomonads. This means that if the common ancestor of imbricates had scales, they must have been lost by at least four of these five orders. Cavalier-Smith and Chao (2012a) added two more non-flagellate silicified orders of filose protists to Imbricatea because one (Perlofilida) has silica perles resembling the thecal granules that surround Spongomonadida and the other (Rotosphaerida) has two-tier silica scales resembling those of thaumatomonads; we now exclude rotosphaerids from Cercozoa because of unpublished evidence that at least one belongs in Amoebozoa. They also subdivided the by then numerous imbricate orders into two morphologically and phylogenetically coherent subclasses (Placonuda including euglyphids and putative relatives; Placoperla with thaumatomonads and their putative relatives). Scoble and Cavalier-Smith (2014) added to Placonuda a second order with imbricate single-tier scales distinguished from euglyphids by being non-amoeboid, non-gliding flagellates (Zoelucasida; rDNA sequences unavailable). Apart from the position of Discomonadida being variable, but sisters to Placonuda on Monadofilosa-only CAT trees, latest rDNA trees are reasonably consistent with the monophyly of both Placonuda and Placoperla (Figs. S8 and S12; Scoble and Cavalier-Smith 2014; Shiratori et al. 2014). However, whether Placonuda and Placoperla are sisters or not is unclear as some rDNA trees group them weakly but others do not (Scoble and Cavalier-Smith 2014 and Figs. S8–15).

Our multiprotein trees clearly favour a non-sister relationship between these two subclasses, but still include only three classical imbricates (*Nudifila*, *Paulinella*, *Thaumatomonas*). The naked filose amoeboflagellate *Nudifila* (Variglissida) is moderately to well supported as sister to the euglyphid *Paulinella*, making it likely that these two orders are quite closely related and both rightly in Placonuda. As Marimonadida consistently group (sometimes with high support) with them by rDNA (Fig. S8), it is highly likely that all three are a genuine clade, though multigene trees are needed to test whether Marimonada are sisters of Variglissida (making Nudisarca a clade) or not (making Nudisarca paraphyletic ancestors of euglyphids). Our multiprotein trees also group the *Nudifila*/*Paulinella* clade so strongly with the *Neocercomonas*/*Sandona* Pediglissa clade, and thus exclude *Thaumatomonas*, that we conclude that Placoperla and Placonuda are not sisters. Thus Imbricatea as recently circumscribed appear not to be a clade, making the distinction between them and Sarcomonadea unsatisfactory.

We considered correcting this problem by down-ranking Imbricatea to subclass and transferring them (with Placonuda and Placofila ranked lower as infraclasses) to Sarcomonadea so Thaumatomonadida would again be sarcomonads as in the original class (Cavalier-Smith 1995 1997); if thus expanded Sarcomonadea would likely be a clade. However, as the position of thaumatomonads is contradictory amongst trees, it seemed wiser to delay changing circumscription of these classes until more and genically still better sampled transcriptomes become available. Sound decisions should also be easier when we have rDNA sequences for two more imbricate orders [silica scale-bearing Zoelucasida (which might correspond with the environmental DNA sequences that are sister to Euglyphida) and perle-secreting Perlofilida] and more transcriptome sequences (and better genically sampled ones) for divergent imbricate lineages, including spongomonads and the remarkable filose cercozoan *Kraken* that has single-tier scales somewhat like those of Euglyphia (Dumack et al. 2016b, 2017a). *Kraken* failed to group with even moderate support with any other cercozoan orders and differs so much in morphology from all other Cercozoa, including a unique cell surface groove indenting the associated nucleus, that we establish a new order Krakenida for it. Our monadofilosan CAT trees identified environmental DNA clade eSarcomonad (unknown morphology) as its likely closest relative rather than paracercomonads as previously supposed, so we group them as new subclass Krakenia within Imbricatea (Table 1). Our extremely taxon-rich site-heterogeneous 18S rDNA trees (Figs. S12 and S13) group Krakenia weakly as sister to classical Imbricatea and are likely more reliable than published more sparsely sampled (especially for 28S rDNA-gene) homogeneous ones. This position of Krakenia is more consistent with the presence of scales than would be a relationship with paracercomonads weakly seen previously on site-homogeneous trees (Dumack et al. 2016b, 2017a); scales are unknown in paracercomonads. Scoble and Cavalier-Smith (2014) discussed imbricate scale evolution in detail; there is clear evidence that scales were lost in the thaumatomonad *Esquamula*. If the deeper-branching position of *Thaumatomonas* on some of our trees were to prove correct, then scales may have been more frequently lost in imbricates than once thought. But it is hard to decide whether scale absence in Nudisarca results from such losses or reflects separate origins of scales in Placonuda and Placoperla.

### Monadofilosan superclasses Eoglissa and Ventrifilosa

Thecofilosea and Imbricatea were grouped as superclass Ventrifilosa on the assumption that their common ancestor had ventral filopodia emanating from a groove (Cavalier-Smith and Karpov 2012b). As our trees suggest that thus-defined Ventrifilosa may be polyphyletic because imbricate subclasses probably nest within Sarcomonadea, we now extend circumscription of Ventrifilosa to include Sarcomonadea (Table 1), which likely ancestrally had ventral filopodia, though a ventral groove is generally less pronounced than in many original Ventrifilosa. Thus revised, Ventrifilosa are a strongly supported (0.92) clade on Fig. 1, but weakly so on Fig. 2 (the discordant position of genically weakly sampled *Rhogostoma* on Fig. 3 outside that clade is not a significant contradiction). This transfer also makes basal monadofilosan superclass Eoglissa comprising the two, now demonstrably early-branching, ancestrally gliding flagellate classes Helkesea and Metromonadea, more homogeneous than when it also included sarcomonads (Cavalier-Smith and Oates 2012c). Eoglissa are monophyletic in the classical sense (Cavalier-Smith 2010b) and their common ancestor may have been the first cercozoan to glide solely on its posterior cilium (no known Reticulofilosa have this phenotype, as Skiomonadea glide on both cilia simultaneously, whereas Chlorarachnea and Granofilosea do not glide) and constitute the ancestral group of Monadofilosa from which the much more speciose Ventrifilosa later evolved.

#### Revised diagnosis of Eoglissa

Cavalier-Smith: zooflagellate Cercozoa that glide on posterior cilium only; anterior cilium very short (Metromonadea), reduced to a vestige or papilla lacking 9 + 2 structure or absent (Helkesida); or secondarily non-ciliate aggregative amoebae (*Guttulinopsis*). Microtubular centriolar roots simpler than in Ventrifilosa, only one or two microtubular bands in ciliate helkesids.

### Phylogeny and evolution of Retaria

Our trees include the tubothalamean foraminiferan *Sorites* with reasonable gene sampling (8319 amino acids), which would have been much higher had its transcriptome not been contaminated by far more dinoflagellate genes (presumably endosymbiont) as discovered by Cavalier-Smith et al. (2015b). Nonetheless, *Sorites* groups strongly with *Quinqueloculina* forming a robust miliolid clade. All our trees show all three foraminiferan classes as clades with the same internal branching order, with *Reticulomyxa* sister to Globothalamea (represented only by rotaliids), and thus clearly secondarily naked without a shell, contradicting the idea that Monothalamea are the most ancient foraminiferan class (Pawlowski et al. 2013). This finding agrees with Sierra et al. (2013), but Sierra et al. (2016) curiously omitted both Tubothalamea, a taxonomic bias that may partly explain why they did not recover a Radiozoa clade or the *Bulimina*/*Ammonia* clade that all our trees by both methods found with 99–100% support and the supplementary 109-taxon tree of Sierra et al. (2013) showed with strong support (their main, genically too sparse 36-gene tree did not). Krabberød et al. (2017) also omitted Tubothalamea but globothalamean genes were better sampled and the class maximally supported. Reticulopodia of Foraminifera unlike typical cercozoan filopodia are not simply supported by actin filaments but also by irregularly arranged microtubules (present also in filopodia of at least some Granofilosea).

By contrast, all Radiozoa have axopodia supported by axonemes of cross-linked microtubules, typically in open hexagonal array, nucleated by a prominent centrosome-like axoplast (or several), which long made it likely that they are a clade. Five out of six of our eukaryote-wide trees, all three 72–75-taxon chromist-only trees and most of our 32–3 taxon Rhizaria-only trees show Radiozoa as a robust clade; only a small minority show Radiozoa as paraphyletic as on the more sparsely sampled trees of Sierra et al. (2013). Thus concluding that Radiozoa are paraphyletic (Sierra et al. 2013) was premature, most likely incorrect (their 36- and 109-gene trees showing paraphyly were mutually contradictory with respect to internal phylogeny of Radiozoa and thus unstable). Krabberød et al. (2017) found Radiozoa as a clade with maximal CAT and weak ML support, in full agreement with our trees; they also showed that nassellarian *Lithomelissa setosa* is sister to *Collozoum*—thus all three polycystine radiolarian orders form a clade Polycystinea with high CAT and moderate ML support.

The common exclusion on our trees of Ectoreta from Endomyxa (represented by five species) agrees with the ML tree of Burki et al. (2010) that had a strongly supported endomyxan clade; however, our ML trees only weakly supported Endomyxa being a clade. These trees contradict the 36-gene ML tree of Sierra et al. (2013) that included four Endomyxa and only 22 Rhizaria, and the 119-gene trees of Burki et al. (2013) also with 5 Endomyxa but only 20 Rhizaria, and the CAT tree only of Burki et al. (2010) with 3 Endomyxa and 10 Rhizaria, which all placed Ectoreta within Endomyxa as sister to Gromiidea. They also contradict the 229-gene ML trees where Endomyxa were paraphyletic, because Ectoreta were sister to Marimyxia, but support their likely more reliable site-heterogeneous trees that weakly support clade Endomyxa with Marimyxia and Phytomyxia sisters (Sierra et al. 2016). The main analysis of Krabberød et al. (2017, Fig. 1) only had two Endomyxa, but their subsidiary analysis with seven endomyxans (their Fig. 6) strongly supported Endomyxa being a clade that exclude Ectoreta, in accordance with our conclusion.

Our observations on actin and tubulin sequences of the endomyxan *Filoreta marina* supplement those of Krabberød et al. (2017) for Retaria. We found only one β-tubulin and two closely related α-tubulin genes (protein sequences probably identical) but no substantially different paralogues for either. Our single-gene trees including many more Cercozoa than theirs are consistent with their conclusion that both tubulins underwent duplication in the ancestor of Ectoreta after it diverged from Endomyxa, and therefore these extra tubulins likely played a key role in the novel microtubular cytoskeletal properties of Ectoreta. We found three distinct actins in *F*. *marina* (sequences in the electronic supplement) including one (actin3) so extremely divergent that its relationship to other actins is unclear. Of the others, the shorter branch one is related to actin1 of *F*. *tenera*, whereas the other appears in an ML tree sampling all protist groups as sister to polycystine actin2. This is consistent with the actin2 duplication having occurred in the common ancestor of Ectoreta and Endomyxa, as Krabberød et al. (2017) indicated, after Retaria diverged from Cercozoa, and is thus a molecular synapomorphy for Retaria in the now broader sense additional to the rpl1 insertions discussed above. Actin2 might be involved in the novel reticulose cell character of Retaria, and was likely lost in the parasitic Ascetosporea and Phytomyxea when their body form was simplified by losing reticulopodia; its apparent absence from *Filoreta tenera* may be a misleading artefact of gross undersampling of that transcriptome. Confusingly, references to actin1 and 2 in the text of Krabberød et al. (2017) appear to be reversed compared with their tree labelling and GenBank annotations; here, we followed their tree labelling. Based on their and our results, their calling actin1 ‘a synapomorphy for Retaria [i.e. Ectoreta on the revised classification of Cavalier-Smith 2018] and Endomyxa’ also seems incorrect, as actin1 is apparently the ancestral paralogue for all Rhizaria and eukaryotes. Labelling of Fig. 6 of Krabberød et al. (2017) is confusing and incorrect within Cercozoa: (1) *Limnofila* is not a chlorarachniophyte and granofiloseans ‘*Gymnophrys*’ and *Massisteria* are not Monadofilosa; (2) ‘*Gymnophrys*’ (source unspecified) is probably essentially the same strain as *Limnofila*, so their strong placement by evolutionary placement algorithm (EPA) in two very different positions undermines the reliability of EPA—‘*Gymnophrys*’ position apparently depends on just one or two sequences (?Hsp90 alone)—or suggests that key genes may be from contaminants that these cultures clearly had (see above); (3) our trees do not support *Limnofila* being sister to chlorarachnids as shown, and it is likely that the label for myosin and arp duplications at the base of this likely spurious clade is misplaced by one node and relates only to Chlorarachnida—as no *Limnofila*/'*Gymnophrys*’ sequences are in their Fig. 3 arp or Fig. S8 myosin trees, presumably they have no evidence for *Limnofila* sharing these duplications or chlorarachnid-specific myosins.

Of special interest for rhizarian cell biology is the pelagic *Sticholonche* with unique oar-like rowing axopodia moved by Ca^++^-stimulated contactile protein fibres (Cachon et al. 1977), whose numerous microtubules nucleate on the nuclear envelope (leading some incorrectly to consider it a heliozoan: Cachon and Cachon 1978; Febvre-Chevalier 1990; Mikrjukov et al. 2002), and which also has rosettes of radial silicious spicules. *Sticholonche* cell structure is so bizarre that it has long been in a separate class and was most recently classified with Radiozoa (Cavalier-Smith 1993a, 2018) and was grouped with Acantharea as subphylum or superclass Spasmaria since both groups share contractile Ca^++^-stimulated cross-striated fibres. rDNA trees strongly supported grouping *Sticholonche* with Radiozoa not Heliozoa, but only weakly grouped it with Acantharea (Nikolaev et al. 2004). Krabberød et al. using 10,190 amino acids concluded it is probably sister to Radiozoa plus Foraminifera (shown also by ML only if fast evolving amino acid sites removed from the analysis) and thus sister to all other Ectoreta. That suggests that the spasmin-like contractile fibres were ancestral to Ectoreta and were lost by Foraminifera. Conversely, *Sticholonche* must have lost filopodia and reticulopodia when it evolved rowing axopodia for planktonic life. If it is genuinely the most divergent ectoretan, it may never have evolved a central capsule, but we conjecture that proteins of the reinforced nuclear envelope may be related to those of central capsules. Very likely, the ancestral ectoretan had axonemes with a lattice of open hexagons with 6 microtubules, which increased to 12 in Polycystinea only and became more disorganised in the foraminiferan reticulopodial skeleton. Given its deep branching and ultrastructural distinctiveness, we remove *Sticholonche* from Radiozoa and make Sticholonchia a third infraphylum of Ectoreta (Table 1):

#### Diagnosis of Sticholonchia

Cavalier-Smith infraphyl. n. Large planktonic cells that swim by rowing axopodia driven by spasmin-like Ca-stimulated contractile fibres, with axoneme microtubules in open hexagonal arrays basally attached to dense plates that by flexible joints are associated with heavily reinforced nuclear envelope. Small biciliate zoospores with strontium sulphate crystals. Rosettes of radial spines containing amorphous silica. Reticulopodia absent.

For comparison, we provide a revised diagnosis for infraphylum Radiozoa.

#### Revised diagnosis of new infraphylum Radiozoa

Cavalier-Smith 1987 stat. n. 2018: cells with radiating axopodia supported by axonemes nucleated by one or many intracapsular axoplasts and supporting radiating axopodia (with axonemes with cross-linked microtubules ancestrally in open hexagonal array, each hexagon with 6 (Acantharea) or 12 microtubules (Polycystinea—in some subgroups, hexagons are incomplete on one side, thus resemble branching palisades); axopodia thicker and longer than reticulopodia. Mineralised skeleton usually present; largely strontium sulphate (Acantharea) or amorphous silica (Polycystinea). Ectoplasm separated from endoplasm by cortical alveoli and intracellular dense multiperforated central capsule sandwiched between cortical alveoli and plasma membrane invaginations (Polycystinea) or by extracellular fibrous capsule (Acantharia) or test (Foraminifera). Zoospores biciliate.

### Origin and early divergence of Rhizaria

Our trees strongly confirm the holophyly of Rhizaria and its sister group Halvaria (Alveolata, Heterokonta). Those of Krabberød et al. (2017) similarily show rhizarian holophyly with maximal support by both methods, but only their site-heterogeneous trees give maximal support to Halvaria being a clade. Heterokonts like Raphidophyceae have well-developed cortical alveoli like those of alveolates, as do haptophytes and *Ancoracysta* in Hacrobia, and Glaucophyta within Plantae; if these structures are homologous, they must also have been present in the common ancestor of Harosa and thus in the harosan ancestor from which Rhizaria evolved. Though cortical alveoli are absent in Cercozoa and Endomyxa, we postulate that the membranes that surround the central capsules of polycystine Radiozoa (Cachon et al. 1990) are relics of these cortical alveoli, retained because depositing dense material between them and the plasma membrane was a simple and useful way of evolving a central capsule as supportive skeleton for these giant cells, and also protective as reticulopodia could be withdrawn within them, and so protect their large cells in adversity or from smaller predators.

By contrast, ancestral Cercozoa remained flagellates like their harosan ancestors but evolved posterior gliding and generally remained much smaller and more mobile cells than Retaria, especially well adapted to interstitial habitats amongst sand grains or soil particles, where their putatively ancestral dual ability to glide on surfaces and extend long filopodia around such particles to trap bacteria on the other side is probably a key to their evolutionary success and large megadiversity. This mode of feeding became highly successful and diversified as one of the earth’s major adaptive zones for micropredators [for the concept of an adaptive zone, essentially a group of related niches fitting a particular body form, and the evolutionary importance of megaevolution (the origin of higher-ranked taxa) often associated with radically novel adaptive zones, see Simpson (1944, 1953) and Mayr (1988a, b)]. The 32 cercozoan orders are more than for mammals, birds or insects. We suggest that the ancestral cercozoan simplified its cortical structure by losing cortical alveoli when it evolved filopodia for feeding as this would allow filopodial growth anywhere on its surface. Endomyxa did not evolve ciliary gliding, suggesting that this evolved after filopodia.

As Endomyxa are sisters of Ectoreta, they presumably lost cortical alveoli independently of Cercozoa. Endomyxa lost the flagellate trophic stage, by complete ciliary loss (Vampyrellidea, Ascetosporea, Reticulosida) or by confining cilia to specialised non-trophic uniciliate agametes (*Gromia*) or biciliate zoospores for dispersal in two life cycle phases (Phytomyxea) as in Ectoreta. Restriction of cilia to short-lived phases may have been associated with hypertrophy of reticulopodia in the ancestral retarian.

Crucial to better understanding the cercozoan/retarian divergence is to establish the phenotype of early diverging rhizarian clade NC 10/12 known only from environmental DNA sequencing. Is it cercozoan, retarian or a third unexpected phenotype? As noted above, rDNA trees are too unresolved to say whether it is sister to Retaria, to Cercozoa or to both. Culturing and obtaining hundreds of gene sequences from a representative should answer this and further clarify rhizarian origins; as we go to press, one NC10 lineage (‘Aquavolonida’) turns out to be a non-amoeboid biciliate that can both swim and glide (Bass et al. 2018), so this is classified as cercozoan in Table 1; a similar, only partially surface-asociated, phenotype could be ancestral for Cercozoa—possibly also for Rhizaria.

### Key importance of surface-associated lifestyle and pseudopodial diversification in Rhizaria

Previously, it was argued that the corticate body plan evolved in the common ancestor of Plantae and Chromista by origin of cortical alveoli as a skeleton for cortical support, thus allowing larger alga-eating cells to invade the photic zone as active swimmers (Cavalier-Smith 2004, 2007, 2009, 2018), thereby differentiating them from their smaller-celled mainly bacterivorous excavate ancestors. That presumed ancestral corticate mode of life was essentially retained by four of the five corticate groups (Plantae plus three chromist groups: Alveolata, Heterokonta, Hacrobia), which became algae by symbiogenesis (Cavalier-Smith 2013a, 2018); amongst them, a pseudopodial mode of feeding is entirely absent in Plantae and rare and always secondary in these Chromista (e.g. *Chrysamoeba*, ‘*Chlamydomyxa*’ within heterokont ochrophytes; *Dinamoeba* within alveolates).

By contrast, Rhizaria are the sole major corticate group of clearly ancestrally heterotrophic benthic and surface-associated protists, which is why they were only recently recognised as chromists rather than Protozoa (Cavalier-Smith 2010a). This marked lifestyle change from the presumed ancestral planktonic cortical condition was effected by evolution of filopodia and ciliary gliding in Cercozoa and of reticulopodia in Retaria. We argue that filopodia evolved first (essentially an actomyosin-associated cytoskeletal innovation) and that several lineages independently added pseudopodial fusion (a membrane change) to establish a network that could spread on surfaces for feeding. The ancestral retarian alone almost immediately evolved even greater cell size that became gigantism in most Ectoreta. The frequency with which Retaria harbour algal symbionts (most Ectoreta inhabiting the photic zone) or feed on algae (both Endomyxa and Ectoreta) suggests that in this respect, they retained the ancestral corticate trophic preference and large cell size, whereas Cercozoa by becoming interstitial filose gliders often focus on bacterial prey and cell size reduction. Ancestrally, marine vampyrellids lost cilia but retained a preference for eating algae (one major subclade adapting to soil by adding fungi to the menu). *Gromia* also retained algivory and evolved an extracellular test by modifying the cercozoan ancestral resting cyst to make an aperture. As Rhizaria diverged from other chromists immediately after the ancestral red algal secondary enslavement (see Cavalier-Smith 2013a; Cavalier-Smith et al. 2015a; 2018), their last common ancestor was able to lose the chromist chloroplast almost immediately it first evolved filopodia, but many lineages retained the capacity to harbour oxygenic symbionts. In Cercozoa, two marine groups re-evolved photosynthetic lifestyles: Chlorarachnida by enslaving a green alga (Cavalier-Smith 2013a) and the euglyphid *Paulinella* by enslaving a cyanobacterium (Nakayama and Ishida 2009). A third marine cercozoan *Auranticordis* suspected to harbour cyanobacterial endosymbionts (Chantangsi et al. 2008) needs further study to see if they are integrated or dispensible. As Cavalier-Smith (2018) explained, origin of ciliary gliding by Cercozoa may have favoured the conversion of the ancestral bypassing microtubule band of Chromista into ventral posterior ‘root’ vpr2, which is probably not really a centriolar root as it bypasses both centrioles and is probably not nucleated by either. It would have helped strengthen the ventral groove alongside the gliding posterior cilium which has to support the cell body during gliding. As Cavalier-Smith (2018) also explained, axopodia of desmothoracid Cercozoa and of Radiozoa and *Sticholonche* may have evolved from the ancestral chromist bypassing band that is present relatively unchanged in many Halvaria. If that band is not nucleated by centrioles, it could have been preadapted for nuclear nucleation as in Radiozoa and *Sticholonche*, thus the ancestral condition for Ectoreta. Non-reliance on centrioles for axopodial nucleation is compatible with the complete loss of centrioles and cilia in vegetative cells of Retaria and thus enables an aciliate axopodial lifestyle.

### Position of Rhizaria within Harosa and Chromista

Our trees strongly support Halvaria being a clade, and thus establish a robust deep phylogeny for chromist subkingdom Harosa: Rhizaria are always sister to Halvaria. Burki et al. (2016) using 250 proteins and 55,554 amino acids (excluding all positions missing in more than 20% of taxa) and 148 or 150 eukaryote-wide taxa likewise found maximal support for this topology on site-heterogeneous trees. Their ML trees showed the same topology with weak support using 55,554 positions. When only the most conservative positions were used (36,507 amino acids), Rhizaria were sisters of heterokonts only with strong support by both methods (a contradiction not mentioned in their text). Oddly, Sierra et al. (2016) using 229 proteins (64,107) amino acids, but only 56 taxa contradictorily put Rhizaria instead as sister to Alveolata on ML trees. Neither of these alternative topologies for Harosa was found in any of our other multigene papers (Cavalier-Smith et al. 2014, 2015a, b).

A likely reason for these major discrepancies is the extremely high fraction of missing data in all Ectoreta (noted above) that dominated the relatively small taxon sample of Sierra et al. (2016) and high missing data in most Rhizaria. No Ectoreta had less than 56% missing positions, and all but 2 has at least 70% missing; *Bigelowiella* and *Plasmodiophora* were the only rhizarians with less than 20% missing and only 19 of the 56 taxa met the criterion of < 20% used by Burki et al. to exclude weakly sampled taxa. Even some species with complete genomes were surprisingly undersampled (*Arabidopsis* 43% missing; *Toxoplasma* 25% missing, *Blastocystis* 27% missing, *Plasmodium* 24% missing). Sierra et al. (2016) and Burki et al. (2016) did not include the foraminifera *Sorites* or *Quinqueloculina*, two of the three genetically most richly sampled ectoretans in our analyses. As nine of the ten new rhizarian sequences added here are genetically more richly sampled than the vast majority of Rhizaria in Sierra et al. (2016), it is unsurprising that we found the same topology as did the most sequence-rich trees of Burki et al. (2016), who carefully excluded grossly undersampled taxa which can cause serious systematic errors in tree reconstruction (Roure et al. 2013). A second problem with the Sierra et al. (2016) analysis is arbitrary and suboptimal outgroup choice: using only Viridiplantae as outgroup for Harosa was unwise, as they are not the closest outgroup (Hacrobia are, as used in our Figs. 2 and 3) and also have much longer branches than Glaucophyta that we therefore used instead to represent Plantae in our eukaryote-wide trees. In general, it is safest to use a balanced spread of diverse outgroups for rooting trees, as a single arbitrary one (far too often used) could by chance have features that pull it to the wrong position in the ingroup, especially if it is distant or rapidly or oddly evolving, and especially if the ingroup is genetically undersampled. That clearly happened in Sierra et al. (2016) if compared with other recent studies using eukaryote-wide outgroups that all found monophyletic Halvaria by both CAT and ML (Burki et al. 2012, 2013; Cavalier-Smith et al. 2014, 2015a, b, 2016). Sierra et al. (2016) also used PhyloBayes CAT, less prone to such artefacts; that also strongly gave the same discordant phylogeny when *Mikrocytos* was excluded but did not significantly support it in the presence of *Mikrocytos*.

Grant and Katz (2014) using 232 eukaryotes and the 150 most evenly sampled genes across these taxa also found the Halvaria clade by ML though with under 80% support. However, they found the same discordant Alveolata/Rhizaria topology when using 207 genes with a mere 17,220 positions for eukaryotes plus prokaryotes, but the basal branching of eukaryotes in this 3-domain tree is obviously grossly distorted by long-branch artefacts; using ML alone rather than CAT for 3-domain trees is technically inadequate (Lasek-Nesselquist and Gogarten 2013; Raymann et al. 2015) and cannot be considered reliable. Nonetheless, their eukaryote-only ML tree that agrees with ours and Burki et al. (2012, 2013, and most trees in 2016) for harosan deep phylogeny confirms the importance of good gene sampling and a broad and sensibly chosen set of outgroups and the reproducibility of clade Halvaria in the best analyses. Grouping Rhizaria with Alveolata alone by Sierra et al. (2016) is therefore no more correct than the first ML-only multigene tree that clearly showed the harosan clade but incorrectly put Rhizaria as sister to Heterokonta (Burki et al. 2007).

A multiprotein phylogenetic study of the imbricate cercozoan filose amoeba *Euglypha* found maximal support for clade Halvaria using site-heterogeneous trees for 147 proteins (He et al. 2016) in agreement with all our analyses; but unlike ours, their ML tree contradictorily grouped Rhizaria with Alveolata—as by both methods did genically sparse trees restricted to 34 or only 27 proteins. They claimed that their sparse 34- and 27-gene trees were correct and that their genically well-represented 147-gene site-heterogeneous tree was wrong and distorted by a long branch artefact, despite recognising that in theory it should be more accurate than the corresponding ML tree. This belief was based on the assumption that their 27-protein set had selectively removed genes most likely to cause artefactual grouping of Halvaria. However, the method aimed at that goal evidently failed to achieve it, as their 27-protein tree (their Fig. 1) shows Rhizaria and alveolates both with systematically substantially longer branches than any other groups; therefore, this tree itself may suffer from a long-branch attraction artefact directly between these two groups; we therefore suggest that their unusual grouping was indeed a long-branch artefact and that trees using more genes [like ours and even better protein sampling by Krabberød et al. (2017)] are in general more reliable. Their 27-protein tree was also based on a very different protein set from their 147-protein tree and our 187-protein trees and most other published multiprotein trees. Of the 27 included proteins, 19 were from genes of eubacterial origin (He et al. 2014), and thus were not genes from the eukaryotic host that enslaved mitochondria as are our 187 proteins—their 34 protein trees included 14 eubacterial-origin proteins. As their selection method certainly did not remove proteins whose tree branches are systematically longer in alveolates and rhizaria than in heterokonts, it obviously failed to remove proteins with systematically above average evolutionary rates in some but not all harosan groups as they had erroneously expected. It may even have introduced a long-branch problem by eliminating numerous phylogenetically informative genes, and retaining a small residue of systematically biased ones.

The protein selection method used by He et al. (2016) should not have been expected to eliminate such biases because to obtain the 27-protein set it selectively removed proteins for which within one of the three harosan groups at least three species had branches at least twice as long as average for that subgroup on single-protein trees. Thus they did not remove proteins with a systematic bias in branch lengths between groups (as would have been necessary to reduce the supposed problem of biases between groups) but proteins that had more variable rates within just one subgroup, which is irrelevant to the question of systematic bias amongst groups. Many such genes could have had a lower average rate of evolution than genes not removed, but would have been wrongly removed just because some non-representative lineages had fast rates. Thus the claim that the 27-protein trees were technically superior was based on a fallacy and a confusion between systematic cross-group variation in evolutionary rates and random within group variation. Conversely, their method would have retained genes for which all genes within a group were systematically biased by extra rapid evolution, which potentially could cause long-branch attraction, but would have eliminated many proteins where the majority within a group were well-behaved short branches just because some branches were longer—both the exact opposite of what the method was supposed to achieve. Fast site removal by Krabberød et al. (2017), a more reliable way of detecting long-branch problems, showed the exact opposite of what He et al. (2016) concluded, i.e. that the ML trees are more affected by rapid evolutionary rates than CAT trees and thus less, not more, reliable.

As explained above, we found that much of He et al. (2016) raw data for Rhizaria included contaminants. He et al. (2016) recognised that, but instead of just removing protein sequences for particular species recognisable as reasonably unambiguous contaminants, as we did, they generated a 34-protein alignment by removing all proteins from the alignment where more than one species did not group with other Rhizaria. That was unwise as it must have removed large numbers of genes from the analysis for both rhizarian and non-rhizarian taxa for which individually there was no reason whatever to suspect contamination. In our experience, it is often the case that single-gene trees do not show monophyly of groups that from other evidence are known to be monophyletic, even in samples where possibility of contamination can be excluded; that apparent polyphyly is probably just because many genes (e.g. ribosomal protein genes) are too short or erratically variable in evolutionary rates to retain enough information to place all branches accurately. Their excessively loose criterion for identifying ‘contamination’ would inevitably have excluded vast numbers of sequences that were not contaminants and left some that were; the authors appear to have had far too much faith in the accuracy of single-protein trees, and as a result discarded almost all the probably useful data and in all likelihood severely degraded the phylogenetic signal. It was illogical to remove all the genes from other taxa just because two were considered to be misplaced; they ought to have removed just the individually suspect genes. It was equally illogical to remove genes where two rhizarian lineages failed to group appropriately but to retain those where only one sequence was misplaced, because contamination affects samples individually; therefore, one might expect it to be more likely that a single outlier will be a contaminant than a group of two, which might reflect a different kind of error. If He et al. (2016) really believed that all proteins thus (mis)identified as contaminants really did include potentially misleading contaminants, they ought to have excluded them from the 27-protein set, but curiously did not. The 27 proteins included 14 (a majority) that they claimed to have been contaminants and ought to have excluded; that would have left only 13 proteins for analysis, so few to have been useless. In sum, the authors’ claim to have identified and eliminated a long-branch artefact affecting harosan deep phylogeny is seriously flawed.

To support their entirely unwarranted conclusion, He et al. (2016) asserted that the numerous previous studies that contradicted it ‘mostly either lack substantial data for Rhizaria ... and/or have only extremely long rhizarian branches to work with’. Neither criticism is applicable to our present study, which adds massive data for nine rhizarians (eight cercozoan), every one of which has shorter branches than any Ectoreta; the majority have shorter branches than in alveolates and several have shorter branches than in many heterokonts. In number of genes and taxa, the shorter-branch Cercozoa and Endomyxa now dominate our trees compared with the more sparsely sampled longer branch Ectoreta. Our trees for 56 Harosa using 187 proteins are therefore much more convincing than theirs for just 26 Harosa using only 27 or 34 proteins selected for entirely erroneous reasons. It is now clear that neither cercozoan nor rhizarian proteins are systematically fast-evolving compared with those of Halvaria; of the four rhizarian subphyla, only Ectoreta has systematically longer branches than harosans generally, which can cause long-branch artefacts on sparsely sampled trees such as those of He et al. (2016) that are deficient in cercozoan data. Those sparse trees included only nine rhizarian branches and only three for Cercozoa, as many taxa were lumped as in silico composites, and therefore told us nothing more than had 18S rDNA trees about the position of *Euglypha*, the initial target of their study; they merely confirmed that *Euglypha* is closer to *Paracercomonas* and *Aulacantha* than to *Bigelowiella*, which our trees with 14 Cercozoa sensu stricto show much more decisively using *Paulinella* as the euglyphid representative. As rDNA trees had already indicated that *Euglypha* is probably phylogenetically closer to *Aulacantha* than to *Paracercomonas* (Scoble and Cavalier-Smith 2014), lumping the two latter as a single false taxon was phylogenetically illogical and would necessarily have prevented establishing *Euglypha*’s true position. Fast site removal by Burki et al. (2016) was theoretically sounder than the He et al. method and yielded a contradictory tree with Rhizaria sister to heterokonts, but their tree was far less well sampled than ours for Rhizaria (6 versus 31 species). We deliberately omitted the longest branch alveolates to reduce artefacts, but the Burki et al. tree included three alveolates with much longer branches than the other two harosan groups, which might have led to artefactual long-branch exclusion of alveolates in their shorter-sequence trees causing Rhizaria to group wrongly with the relatively short-branch heterokonts; their choice of proteins also gave greater inter-group branch length disparity than ours.

Further evidence for monophyly of Halvaria and against the interpretations of Sierra et al. (2016) and He et al. (2016) comes from Krabberød et al. (2017) who enriched ectoretan taxon sampling by adding a nassellarian and *Sticholonche* and extra foraminiferan genes (*Sorites* still omittted) to reduce missing data and whose 255-protein CAT tree has maximal support for a halvarian clade. Because their ML LG tree contradictorily grouped Rhizaria with alveolates as in the sparse trees of Sierra et al. (2016), Krabberød et al. removed the four fastest evolving rate clases of amino acid sites and found that support for this almost certainly incorrect topology dropped from 96 to 50%. That suggests that rapidly evolving sites misleadingly enhance the incorrrect topology seen on some ML trees. Perhaps the main reason why Krabberød et al. (2017) were unable to eliminate the ML likely artefactual grouping of rhizaria with alveolates entirely may have been serious undersampling of shorter-branch Cercozoa and Endomyxa on their tree compared with ours, and possibly their including *Aulacantha* contaminants [earlier, we found that its raw data set from Sierra et al. (2013) included many alveolate genes (Cavalier-Smith et al. 2015a)] which presumably made *Aulacantha* (one of their only two cercozoa) a much longer branch than on our trees. Finally, the 351-protein tree of Brown et al. (2018) has maximal support by ML (two methods) and CAT for clade Halvaria. These authors took great care to test effects of removing fast-evolving sites by reliable methods, not those of He et al. Agreement of the well-sampled CAT trees of Krabberød et al. (2017) for 24 Rhizaria and genically richest ones of Brown et al. (2018) with ours (31 Rhizaria: 14 Cercozoa, 17 Retaria) strongly favours Rhizaria being sister to Halvaria, and argues against both contradictory results from the less well-sampled analyses of Burki et al. (2016), Sierra et al. (2016) and He et al. (2016).

Previously, we suggested that in corticate multigene trees, the deepest branching within kingdoms Chromista and Plantae may be mutually distorted when red algae are included (Cavalier-Smith et al. 2015a). The evidence for mutual interkingdom distortion is that basal topology of each kingdom differs when the other is included or excluded without any other changes in taxon or gene sampling or analytic method (Cavalier-Smith et al. 2015a). In the absence of chromists Plantae are a robust clade, with Viridiplantae strongly sisters of Rhodophyta, and Glaucophyta the deepest branch, as all other evidence indicates is evolutionarily correct; when chromists are added, Rhodophyta are commonly pulled away from Viridiplantae to become the deepest branch of Plantae with some or all Hacrobia often intruding into Plantae or becoming their apparent sisters or ancestors. Conversely, in the absence of Plantae, Chromista are a robust clade with Hacrobia a clade and sister to Harosa; but when Plantae including Rhodophyta are added, they intrude into or become mixed with it. This mutual topological distortion is a fact irrespective of how it can be explained (Cavalier-Smith et al. 2015a). We suggested it is caused by chromists being chimaeras of two eukaryotes, one a red alga, coupled with the practical impossibility of distinguishing all chromist genes as being of host or red algal origin, because the host and red algae diverged so closely in time and so long ago that phylogenetic signal, which in theory might allow their identification, has been either erased or become so faint as to prevent their accurate placement on single-gene trees (Cavalier-Smith et al. 2015a).

Figure 4 is a novel test of this explanation of the mutual distortion phenomenon. In addition to testing the position of the possibly mixed ‘*Minchinia*’, we wanted to see if we represent Plantae solely by the shortest branch Glaucophyta, whether Chromista and Hacrobia remain holophyletic as they are in the complete absence of Plantae. The answer is that on one CAT chain (Fig. 4), Chromista was a maximally supported clade, and (except for exclusion of the single undersampled, highly divergent species *Telonema subtilis* that lacks close relatives to stabilise its position) Hacrobia is a strongly supported (0.98) clade; but on the other chain, Glaucophyta intrude into Chromista and group with Hacrobia (only 0.64 support) and even more weakly group with core Cryptista (0.54). Burki et al. (2016) agree that relatively poorly sampled *Telonema* and *Picomonas* single-species lineages are currently hard to place accurately on trees and excluded them from some of theirs—though their excluding the third corbihelian *Microheliella* (Cavalier-Smith et al. 2015a, genically better sampled than *Picomonas*) would hinder correct placement of this cryptist subphylum. Together, our Figs. 1 and 4 completely refute the criticism by Burki et al. (2016) that we get well-supported holophyletic Hacrobia only when ‘a large part of the diversity’ is removed. These 159–162 taxon trees have more deep eukaryote diversity than the 150-taxon tree of Burki et al. (2016), differing in removing just one clade (Plantae in Fig. 1 and Rhodophyta/Viridiplantae in Fig. 4). In marked contrast to Burki et al. (2016) whose trees all included both red algae and chromists, we found no convincing evidence for the deeper branching of Cryptista compared with other chromists as their trees suggested (some with insignificant and some with strong support). Their trees were mutually contradictory with respect to the exact position of Cryptista. Their full data set split them into three with *Telonema* grouping with other chromists as in our Fig. 1, core Cryptista being sisters of the probably false Viridiplantae/Glaucophyta clade (by ML and 2 CAT chains but sister to holophyletic Plantae on chain 3) and *Picomonas* sister to Rhodophyta. When *Telonema* and *Picomonas* are excluded, ML still groups core Cryptista with Viridiplantae/Glaucophyta, but only one of the three CAT chains did so the other two showing clade Plantae with core Cryptista their sisters. When faster sites were removed, ML still showed the same Viridiplantae/Glaucophyta pseudoclade (without support) with Cryptista their sisters (no support by one ML method) as does one CAT chain, the other showing holophyletic Plantae.

As site-heterogeneous trees are evolutionarily more realistic, the Burki et al. (2016) results on balance argue that Plantae are a clade and intrusion of Cryptista into Plantae on some of their trees (and one of our Fig. 4 chains) is phylogenetically misleading. Neither we nor they have any compelling evidence against holophyly of Plantae. All their trees agree in showing a Viridiplantae/Glaucophyta clade that is absent when Chromista are excluded, and thus probably arteficial. Interestingly, their earlier trees using slightly more (258) genes but only 68 eukaryotes did not show that clade (Burki et al. 2013) and strongly support (both ML and site-heterogeneous trees) the Viridiplantae/Rhodophyta clade that we found when chromists were omitted and distortion was impossible (Cavalier-Smith et al. 2015a). We do not understand why their two papers are so strongly contradictory for basal topology of Plantae, but note that for ML the 2016 paper used IQ-TREE and bootstrap approximation methods not yet widely used, not RAxML as in 2013, and the 2016 CAT trees did not converge. Until reasons for this major discrepancy are understood, we should be cautious and not overconfident in either. Possibly, they exemplify our argument that observed distortion effects are expected to be highly sensitive to taxon and gene sampling (Cavalier-Smith et al. 2015a) and the principle that resolution is expected to be extremely difficult and disturbable by the slightest biases (that can so easily add up in multigene trees to give strong support for the wrong tree) when several taxa diverge almost simultaneously, as is so for Hacrobia and Plantae.

Nonethless, our previous and present work establish that adding chromists to the tree distorts the branching order of Plantae, often pulling red algae away from Viridiplantae to the base in multigene trees (e.g. Burki et al. 2007, 2009, 2016), though not invariably (e.g. Burki et al. 2012, 2013; Cavalier-Smith et al. 2014, all with the arguably correct Rhodophyta/Viridiplantae clade). It is important for kingdom exclusion tests (as in Cavalier-Smith et al. 2015a) to be repeated with the Burki et al. (2016) data set and algorithms. If they omit all chromists, we predict topology of Plantae would change to move red algae one node higher to become sister of Viridiplantae; conversely, omitting all Plantae should produce a Hacrobia clade. Only if neither happened and well-supported Harosa/Haptista and Viridiplantae/Glaucophyta clades remained could their belief that the chimaeric chromists are not distorting the topology of Plantae on their trees and red algae not distorting that of chromists be defensible. The fact that all genes in their dataset showed ‘no strong affinity between Cryptista and red algae’ does not, as Burki et al. (2016) mistakenly assert, ‘suggest that this relationship [i.e. core Cryptista/Plantae] is a reflection of vertical inheritance rather than owing to a cryptic contamination of endosymbiont genes’. As Cavalier-Smith et al. (2015a) more fully explained, one cannot reasonably expect single-gene trees (mostly entirely unresolved at the base of corticates) to show such a relationship strongly, because basal divergence times within Hacrobia and Plantae were so close together in time and so long ago. Single-gene trees are good for detecting distant contaminants or recent lateral transfers, but not for discriminating amongst groups that diverged nearly simultaneously around 750 My ago. The problem with the founding chromist symbiogenesis is that host and symbiont were so closely related at the time. This is entirely unlike green algal plastid enslavement by euglenoids long after these groups diverged or the symbiogenetic origins of plastids and mitochondria where the symbionts were even more distantly related to the hosts, so single-gene trees easily strongly resolve their foreignness.

Furthermore, the assertion that if Cryptista have such contaminants one would expect all other chimaeric chromists to have many such genes and all to be equally attracted to red algae (Sierra et al. 2016) was also erroneous and refuted in detail previously (see Cavalier-Smith et al. 2015a); the key points are (a) that independent losses in diverging lineages need not be quantitatively similar and (b) the more rapid protein evolution of Harosa will erode the phylogenetic signal for any that remain much more than in slower evolving Hacrobia. These considerations should not continue to be ignored; doing so gives excessive faith in the reliability of basal branching orders on corticate multigene trees. It is imperative to test these distortions further and reach a consensus explanation for them. As they did no reciprocal kingdom removal tests, Burki et al. (2016) provided no convincing disproof of the likely holophyly of Chromista and Hacrobia. However, they provided further evidence for holophyly of Haptista (contrary to Burki et al. 2009, 2012) and that Haptista are related to other chromists (i.e. subkingdom Harosa), not to Plantae. Our present and previous taxon exclusion analyses (Cavalier-Smith et al. 2015a) provide strong evidence that the grouping of core Cryptista with Plantae in Burki et al. (2012, 2016) and the branching order of the three Plantae clades in Burki et al. (2016) only are both phylogenetically seriously misleading.

In contrast, Krabberød et al. (2017) with 255 genes (almost as many as Burki et al. 2016 and slightly more amino acid positions) found holophyletic Plantae without Cryptista intruding and with the expected branching order by both CAT and ML (more strongly supported than the contradictory Burki et al. results) without excluding any major chromist lineages except Corbihelia and Centrohelia (included by us and some by Burki et al.). Perhaps when these groups’ taxa and genes are better sampled, the effects of potentially misleading sequences and undersampling will be reduced enough to recover holophyly of Plantae, Hacrobia and Chromista more consistently. Whatever the reasons for the marked contradictions between their trees and those of Burki et al. (2016) with respect to plant holophyly and basal branching, they reinforce our thesis that effects of different taxon and gene sampling are widely underestimated and poorly understood, and more caution is needed in using the latest multigene tree with extremely short basal branches for both Plantae and a Plantae/Cryptista (likely pseudo)clade (see especially Krabberød et al. 2017) as an excuse for rejecting well-corroborated ideas like monophyly of Plantae, Hacrobia and Chromista (for Chromista, see discussion in Cavalier-Smith 2018). Brown et al. (2018) using 351 proteins found 2 contradictory CAT consensus tree pairs for 61 eukaryotes both with every node maximally resolved: in 1 Hacrobia is a maximally supported clade, in the other it is maximally supported as polyphyletic with Cryptista wrongly branching within Plantae, which ultrastructure, periplastid derlin evolution and the shared hacrobian lateral gene transfer all strongly contradict (Cavalier-Smith 2018). This decisively shows that a maximally supported bipartition on a CAT multiprotein tree can sometimes be wrong and that sometimes synthetic cladistic arguments can be more reliable than maximally supported 351-protein trees. Contrary to Janouškovec et al. (2017), ‘phylogenomics’ (an often misleading catchphrase; their study was not genomic but partial transcriptomic) is sometimes less reliable and less decisive than such cladistic reasoning. Exclusion of Cryptista from Chromista as first done by Cavalier-Smith (1995, giving too much emphasis to poorly resolved 18S rDNA trees) is not justified by present evidence.

### Probable holophyly of Metamonada and scotokaryote basal branching order

Figures 1 and 4 are the most broadly sampled eukaryote-wide trees to date using a CAT-GTR-Γ model. Though Metamonada are not our main focus, Fig. 1 provides the first strong site-heterogeneous tree evidence for monophyly of Metamonada (0.96) and more weakly suggests they are sister to *Malawimonas*. This confirms previous evidence for excavate paraphyly (Cavalier-Smith et al. 2014, 2015a) and supports the view that failure of both metamonad clades to group together on previous CAT trees (and our 158- and 162-taxon trees) is more likely statistical artefact than true phylogeny (Cavalier-Smith et al. 2015a). Often low support for conflicting positions of the metamonad clades makes more extensive data necessary to confirm this. All present trees support the conclusion that the putative *Malawimonas*/metamonad clade is sister to podiates, forming a scotokaryote clade, and does not belong in Eozoa with Eolouka, Percolozoa and Euglenozoa (Cavalier-Smith et al. 2015a). They also confirm that *Mantamonas* and *Collodictyon* are related and planomonads branch more deeply than any other Sulcozoa (Cavalier-Smith et al. 2015a), both initially less clear (Cavalier-Smith et al. 2014) and both independently strongly confirmed by Brown et al. (2018) with 351 proteins.

All previous CAT trees including *Malawimonas*, Anaeromonadea and Trichozoa showed them as the deepest scotokaryote branches but their relative branching order varied. Brown et al. (2013) first saw a clade comprising *Malawimonas* and metamonads (represented solely by *Trimastix*). Anaeromonadea (*Trimastix*, *Monocercomonoides*) alone were sisters of *Malawimonas* with Trichozoa one node higher in Cavalier-Smith et al. (2015b; Fig. 3 of 2015a, Fig. 2 showing all Metamonada and Figs. 6 and 2 of 2014 both included metamonads one node below *Malawimonas*). Support for holophyly of *Malawimonas* plus Metamonada, though only 0.79 on Fig. 1, is higher than previously. Previously, Neolouka (*Malawimonas* only) and Metamonada were cautiously treated as distinct phyla (Cavalier-Smith et al. 2015a), but as they are not morphologically distinct enough for separate phylum rank [as Cavalier-Smith (2013b) explained], we hereby formally reverse this and retain Metamonada and Neolouka as subphyla of phylum Loukozoa (as in Cavalier-Smith 2013b p. 121) but Loukozoa is here revised by excluding former subphylum Eolouka (Jakobea and *Tsukubamonas*) and raising it to phylum rank, with **Eolouka Cavalier-Smith phyl. n.** having the same diagnosis and composition as subphylum Eolouka (Cavalier-Smith 2013b p. 121). This formalises revisions that Cavalier-Smith (2018) informally made when redefining neokaryotes as comprising scotokaryotes, Neozoa and Corticata, and more precisely defining excavates as a purely informal group (organisational grade, not clade) comprising only Loukozoa and Jakobea. Thus Loukozoa (in protozoan subkingdom Neozoa) are now split into only two morphologically and phylogenetically distinct subphyla, whereas Eolouka is one of only three phyla of protozoan subkingdom Eozoa, now more narrowly defined than in Ruggiero et al. (2015) in recognition of thus-revised Loukozoa being probably the deepest branch of the scotokaryote clade and thus sister to podiates.

### The centriole/ciliary transition zone junction in Rhizaria

The ciliary transition zone (tz) is distinctively different in Rhizaria from other eukaryotes but varies significantly in length and detailed structure amongst lineages (Cavalier-Smith et al. 2008, 2009; Cavalier-Smith and Karpov 2012b; Hess and Melkonian 2014). Cavalier-Smith et al. (2008, 2009) discovered a highly distinctive hub-lattice structure in *Sainouron* and *Helkesimastix* at the base of the tz just distal to where centriolar triplets start and argued that similar structures can be seen less distinctly in all other Rhizaria where this region is not completely obscured by dense material and argued that it is a synapomorphy for Rhizaria. Hess and Melkonian (2014) demonstrated an asymmetric acorn/Y-filament complex at the extreme distal end of the centriole of the glissomonad *Viridiraptor* indistinguishable from that discovered in *Chlamydomonas* and other green algae, and previously certainly otherwise known only in the metamonad *Pseudotrichonympha* and chytrid fungus *Phlyctochytrium* (Geimer and Melkonian 2004). Geimer and Melkonian (2004) proposed that the acorn/Y-filament complex is present in all centrioles, giving them the asymmetry essential for morphogenesis of non-radially symmetric centriolar roots and the ciliary axoneme, but has been overlooked in most organisms because its very thin, tenuous filaments are generally obscured by other structures.

However, if Metamonada are scotokaryotes, and if the eukaryote tree is correctly rooted within Eozoa (whether as in Figs. 1 and 4 or between *Tsukubamonas* and all other eukaryotes as Cavalier-Smith (2018) suggested, or between jakobids and other eukaryotes as mitochondrial genomes might suggest), then we cannot take the *Pseudotrichonympha* acorn/Y-filament as evidence for its universality in eukaryotes. This is because it has not yet been found in any authentic Eozoa—hypothetically, it might be absent and evolved only in the common ancestor of scotokaryotes and corticates, though the idea that it is the universal determinant of centriolar asymmetry originating in the ancestral centriole (Cavalier-Smith 2014) is more attractive. What is clear is that an acorn/Y is probably universal in scotokaryotes, corticates and specifically Rhizaria. Though the acorn-Y overall is asymmetric, a granule at the Y’s fork is at the centre of rotational symmetry, immediately proximal to the hub of the hub-lattice structure in Helkesida and arguably all Rhizaria (but see caveat in Hess and Melkonian 2014). We suggest that in Rhizaria, this Y-granule is the binding/nucleation site for hub assembly of the Rhizaria-specific hub-lattice complex at the onset of ciliary growth; if so, this universal eukaryotic distal centriolar structure preadapted the rhizarian ancestor for evolving the unique hub-lattice structure. All four non-alveolate corticate groups evolved their own specialised and contrasting ciliary tz structures ancestrally absent in simpler transition zones of alveolates, scotokaryotes and Eozoa, e.g. the star-cylinder of Viridiplantae (Cavalier-Smith 1974, 1981a), transitional helix of Heterokonta (actually just distal to the tz: Hibberd 1979) and double transition plate of many Hacrobia (Cavalier-Smith et al. 2015a), whose evolution Cavalier-Smith (2018) discussed, so ciliary changes played a key role in their basal divergences from the corticate last common ancestor that would have had a simple tz like *Colponema* and ciliates.

### Alveids confirm rhizarian origin from chromists with cortical alveoli and ciliary vanes

Of key importance for understanding the phenotype of the early chromist ancestor of Rhizaria is the new biciliate protist *Ancoracysta twista* that branches strongly by ML as sister to Haptista on a 201-protein tree for eukaryote ‘host’ genes and as sister to haptophytes on a 36 mitochondrial protein ML tree (that lacked centrohelid data) (Janouškovec et al. 2017). *Ancoracysta* represents a fourth, previously unrecognised haptist lineage for which we establish new order Alveida and class Alveidea, as it differs from all others sufficiently to merit this. Alveid ultrastructure has a unique combination of characters that greatly illuminate early chromist diversification, but contrary to these authors is not directly relevant to the root of eukaryotes. Unfortunately, the authors misinterpreted aspects of its structure and failed to notice it was not a novel kind of protist, as claimed, but morphologically so close to ‘*Colponema*’ *marisrubri* that two of them discovered earlier (Mylnikov and Tikhonenkov 2009) as to be in the same genus. Given this ultrastructural near identity and the fact that rDNA does not group *Ancoracysta* with authentic *Colponema* (Janouškovec et al. 2013) or any other alveolates or Harosa, but weakly with the hacrobian biciliate *Telonema* (Janouškovec et al. 2017), *C*. *marisrubri* was undoubtedly misidentified as a *Colponema*; we rename it *Ancoracysta marisrubri* and group it with *A*. *twista* in new hacrobian family Ancoracystidae.

The name Alveida refers to the most striking shared character of both *Ancoracysta* species: a large inflated cortical alveolus (CA) that underlies the entire cell surface except the ciliary pits and cytopharynx. Unlike all other CAs, its lumen has numerous smooth vesicles and a very extensive dense sheet, somewhat thicker than a cell membrane but (unlike membranes) no trilaminar substructure. The inner face of the alveolar membrane was incorrectly labelled “plasmalemma” (pl = plasma membrane, PM) in Janouškovec et al. (2017 Fig. 1 C, E, F); the true PM is the outermost of the two layers labelled envelope (en) in their Fig. 1E, F, the inner of these being the intralumen sheet; it was wrong to lump these completely dissimilar structures together as envelope or theca. The second trilaminar membrane just inside the PM is the outer face of the CA. The authors refer to these layers as four in *A*. *twista* but incorrectly as three in *A. marisrubri*; however, all four are obvious in Mylnikov and Tikhonenkov (2009) Figs. 2 Г, Д and the sheet and some lumenal vesicles are also clear in Figs. 3Д and 3Ж where the CA outer membrane and PM appear as one thicker unresolved line. Disposition of the CA is essentially the same as in haptophytes (discussion and references in Cavalier-Smith et al. 2015a) which differ primarily in lacking the lumenal sheet and vesicles, which corroborates alveid placement in Haptista.

Both alveids have a ventral groove with similar lips, which is shallower than that of colponemids and underlain by a curved sheet (*A*. *twista* Fig. 1d; *A*. *marisrubri* Figs. 3Б, B), dissimilar from colponemid posterior centriolar roots. This transversely C-shaped band (Cb in *A*. *twista*) apparently has a substructure of microtubules associated with dense fibrillar material that often obscures them; at the apical end, it is associated both with the cytostome and with smooth cortical and/or alveolar membranes and apparently enters the zone between the ciliary pits. Thus the anterior extension of Cb is positioned similarly to the axoneme of the haptophyte haptonema and like it is associated with smooth cortical membranes. This organellar complex is therefore an ultrastructurally credible precursor of the haptonemal skeleton and we suggest evolved into it when the groove was lost and the haptonema evolved instead for prey capture, as discussed by Cavalier-Smith et al. (2015a). Our conclusion that *Ancoracysta* are deep branching Haptista thus greatly strengthens our earlier theory of haptonemal origin and inference that the ancestral haptist had extensive CA (Cavalier-Smith et al. 2015a).

A third distinctive alveid-shared feature is the lateral vane on the posterior cilium only, which in *A*. *marisrubri* points away from the cell surface (Mylnikov and Tikhonenkov 2009) and is thus a ventral vane like those of alveolate *Colponema* and neoloukan *Malawimonas*, not dorsal as in jakobid Eozoa. Janouškovec et al. (2017) say that the *A*. *twista* vane points into the groove, which would make it a dorsal vane, but none of their micrographs shows that. Perhaps that is a misinterpretation: their Fig. 1U shows it pointing sideways and slightly out from the groove, marginally consistent with it being ventral as in *A*. *marisrubri*. Such inferences need care as the posterior cilium can probably twist distally on its axis (see drawing of *Colponema loxodes*: Mignot and Brugerolle 1975). Vane morphology and size is indistinguishable between the two *Ancoracysta* species, both narrower than in *Colponema loxodes* or *vietnamica* (Mignot and Brugerolle 1975; Janouškovec et al. 2013), but similar to *C*. aff. *loxodes* (Myl’nikova and Myl’nikov 2010).

The fourth distinctive alveid-shared feature is their ciliary tz, which is essentially identical with prominent well-separated proximal and thicker distal plates, but clearly differs from all other protists and radically so from *Colponema*! As tzs are invariably first class phylogenetic markers, it is puzzling that Janouškovec et al. (2017) did not realise that *A*. *marisrubri* cannot be a *Colponema* and must have been misidentified and be congeneric with *A*. *twista*, especially as they mentioned that *A*. *twista* cell cortex is more like that of *A*. *marisrubri* than any other protist. Prominent double, well-separated, tz transverse plates are found in the majority of hacrobian lineages but not in *Colponema* or most Myzozoa, so are consistent with alveid grouping with Haptista on ML trees.

They also pointed out that *A*. *twista*’s extrusome is more like that of *A*. *marisrubri* than any other protist! In fact, they are so similar that we see no qualitative difference not attributable to technical artefacts of differential fixation (less good for *A*. *marisrubri*). Only their relative proportions differ sufficiently to make the extrusome slightly more elongated and pear shaped in *A*. *marisrubri*. The exaggerated claim that *A*. *twista* has a novel type of extrusome is wrong. Both species share an extrusome that closely resembles the toxicyst of *Colponema loxodes* (Mignot and Brugerolle 1975) and *C*. *vietnamica* and aff. *loxodes* in its mid and posterior parts, but as it differs in its anchor-like anterior, its new name ancoracyst is not altogether superfluous, even though the anterior part seems like a larger version of the anterior part of kinetocysts of centrohelid haptists: the ‘ancoracyst’ appears not radically different from kinetocysts of the centrohelid *Raphidiophrys* (Brugerolle and Mignot 1984) and substantially more like it than *Colponema* toxicysts. They are much less similar to cryptist extrusomes.

*Ancoracysta twista* ciliary hairs are restricted to the anterior cilium. Those labelled mn on Fig. S1O of *A*. *twista* near its base are thin simple hairs like those at the base of some cryptomonad cilia or more extensively in *Colponema* and many Myzozoa; however, there are hints that thicker possibly tubular ones may be present more distally in Figs. S1O and 1T (Janouškovec et al. 2017). Not noted in *A*. *marisrubri*, hairs could easily have been overlooked, but this might be a genuine difference between the species. It is unfortunate that both cultures are lost as it is important to know if tubular hairs are present, which would be the first example in putative Haptista; tubular hairs are found in heterokonts and several cryptist lineages and were postulated as present in the hacrobian ancestor and lost by haptophytes when the haptonema evolved and ventral groove was lost (Cavalier-Smith et al. 2015a) and to have been also lost independently in early Rhizaria and Alveolata.

Janouškovec et al. (2017) said mitochondrial cristae are lamellar, but that is misleading as some micrographs showed narrow tubular cristae in cross section (e.g. Fig. S1X); in *A*. *marisrubri*, narrow tubular cristate and more lamellar ones are also evident (Mylnikov and Tikhonenkov 2009). Thus both *Ancoracysta* species are unusual in having a mixture of tubular and flat cristae. If they are the deepest branching haptist lineage (as multiprotein ML trees for both host- and mitochondrial-origin proteins concordantly show), this odd mixture is unsurprising as haptophyte cristae are tubular and centrohelid ones strongly lamellar. Hacrobian mitochondria generally have undergone more changes in crista shape than other major eukaryote lineages, as Cryptista also include lineages with flattened tubules (cryptomonads) or rounded tubules (others).

In size, cell shape and light microscope morphology, position of nucleus and ciliary pits and ciliary length and orientation both *Ancoracysta* are indistinguishable. Both are marine and from warmer seas, whereas known *Colponema* species are all from fresh water or soil. Overall, apart from the possible (unproven) absence of hairs in *A*. *marisrubra*, the only difference between *A*. *twista* and *marisrubri* is their slight difference in extrusome shape, just enough to justify retaining them as separate species, but not genera. Calling *A*. *twista* ‘structurally unique’ was exaggerated.

Janouškovec et al. (2017) underplayed the consistent evidence from their ML trees for *Ancoracysta* being the deepest branching haptist lineage, giving too much emphasis to their non-converged CAT trees that placed them one node lower as sister to Haptista plus Harosa (‘between’ Haptista and Cryptista), and did not even assign it to superkingdom Corticata to which its CA prove it belongs or to Chromista within which it grouped on all their trees. It was misleading to say it did not branch within a recognised lineage: Chromista has been recognised since 1981 and evidence for its holophyly grows stronger and stronger (Cavalier-Smith 2018). Their trees excluded the deepest branching cryptist group, Corbihelia, so did not strictly demonstrate that *Ancoracysta* is really a novel lineage distinct from Corbihelia. Though on our best CAT trees Corbihelia was sister to other cryptists, on some other trees some or all of its representatives sometimes move down one node to lie between haptists and cryptists or even become sisters of haptists similarly to *Ancoracysta* (Cavalier-Smith et al. 2015a). Nonetheless, on ultrastructural grounds, we do not consider *Ancoracysta* a corbihelian or other deep branching cryptist, as it is more like Haptista in extrusome structure and having an extensive CA system—only one cryptist spcies, *Lateronema antarctica* has cortical flat cisternae, which are probably not CA though sometimes suggested to be related (see Cavalier-Smith et al. 2015a). As *Ancoracysta* is ultrastructurally and concordantly phylogenetically significantly closer to Haptista than to Cryptista, we classify it within chromist subkingdom Hacrobia and phylum Haptista in a third subphylum Alveidia.

#### Diagnosis of new family Ancoracystidae

Cavalier-Smith: Biciliate, uninucleate, heterotrophic chromists with heterodynamic cilia with a long transition zone having widely spaced distal and proximal dense transverse plates; posterior cilium with short lateral vane in mid region associated with shallow-lipped ventral groove supported by promient C-shaped microtubular/microfibrillar sheet; cilia in deep pits; cytopharynx with cytostome opening into anterior pit associated with one or more large extrusomes similar to kinetocysts but with larger anterior anchor-shaped region. All cell surface other than pits and cytopharnyx underlain by largely continuous inflated cortical alveolar sytem whose lumen contains a broad dense fibrillar sheet or sheets and numerous smooth membrane vesicles. Type genus *Ancoracysta* Janouškovec et al. (2017 electronic supplement p. e1). Other species *Ancoracysta marisrubri* comb. n. Cavalier-Smith; basionym *Colponema marisrubri* Mylnikov and Tikhonenkov (2009 p. 6). **Diagnoses of new order Alveida** Cavalier-Smith, **new class Alveidea** Cavalier-Smith, and **new subphylum Alveidia** Cavalier-Smith all as for family Ancoracystidae. **Etymol**. *Alveus* L. cavity, referring to CA system, a much more distinctive feature than the extrusome used for genus; ‘alveids’ avoids confusion with alveolates.

As alveids belong in Hacrobia, they are as distantly related to *Colponema* in Harosa as is possible for any chromists. As both retain *Malawimonas*-like posterior ciliary vanes and ventral feeding groove and have an extensive cortical alveolar system, both characters are arguably ancestral for all chromists, greatly reinforcing earlier arguments that chromists evolved from a vaned *Malawimonas*-like excavate ancestor by the evolution of CA in the common ancestor of Chromista and Plantae (i.e. the common ancestor of superkingdom Corticata) and that chromist phyla that lack CA (Cercozoa, Cryptista) lost them (Cavalier-Smith 2013a, b; Cavalier-Smith et al. 2015a; Cavalier-Smith 2018). This makes the idea that Ectoreta retained CA as their central capsule forming alveoli more plausible also.

### Strong new mitochondrial protein-paralogue evidence for chromist monophyly

Discovery that *Ancoracysta* mitochondria retain 47 α-proteobacterial genes (Janouškovec et al. 2017) and the deep-branching scotokaryote sulcozoan *Diphylleia* kept 51 (Kamikawa et al. 2016) implies that the neokaryote ancestor had at least 55 protein-coding mitochondrial genes, but jakobid Eozoa with 65 have the most gene-rich mitochondrial genomes. The greater primitiveness of jakobids than any neokaryotes is shown most strongly by their being the only eukaryotes that retain eubacterial 4-protein RNA polymerases and SecY. Eozoa also remain the only supergroup that has no known lineages having the neokaryote-specific derived single-molecule cytochrome c haem lyase (holocytochrome c synthase: HCCS) inferred to have evolved in the last common ancestor of corticates and scotokaryotes (Cavalier-Smith 2010a). This argument from cytochrome c biogenesis for the eukaryote root lying within Eozoa or between Eozoa and neokaryotes (Cavalier-Smith 2010a, b) is as strong as ever. Even then, it was known that some chromist mtDNA (i.e. ciliates) and some plants retain genes for some subunits of the ancestral eubacterial multiprotein cytochrome lyase (Ccm) subunits in addition to the novel HCCS (Allen et al. 2008), so the ancestral neokaryote must have had both mechanisms. Discovery that *Ancoracysta* has four Ccm proteins and also has HCCS more directly confirms the idea that ancestral neokaryotes had both HCC and multiprotein lyases and that the α-proteobacterial Ccms were differentially lost independently in different neokaryote lineages (Cavalier-Smith 2010a). Janouškovec et al. (2017) unconvincingly argue that discordances in their single-molecule HCCS tree do not support this, assuming that the tree is dominated by lateral gene transfer (LGT). The tree does have two plausible examples of LGT: one from a chromist to the apusomonad scotokaryote *Thecamonas trahens* and one from a chromist to the percolozoan eozoan *Percolomonas cosmopolitus*. But setting these aside, the rest of the HCCS tree is consistent, given its inevitably poor basal resolution, with vertical inheritance and the origin of the HCCS in the last common ancestor of neokaryotes (i.e. Corticata and scotokaryotes). Paralogy, misrooting and weak resolution of any single-gene tree collectively explain most seeming ‘discordances’ of their HCCS tree much better than does LGT.

Janouškovec et al. (2017) wrongly rooted the tree within Chromista which cannot be the ancestral eukaryote group. If rooted between scotokaryotes and corticates as it should have been, we see that all major groups of Chromista (Cryptista, Haptista, Alveolata, Heterokonta and even Rhizaria) have at least two distinct paralogues, whereas many groups within scotokaryotes and Plantae seem to have only one (as animals at least are known to) and Eozoa none (except for the acknowledged *Percolomonas* LGT). If properly rooted, it fits predominantly vertical inheritance of the two major HCCS paralogues throughout neokaryotes. Janouškovec et al. (2017) overlooked the evidence that fungi have separate paralogues for cytochromes c and c1 and heterokonts and apicomplexa have two paralogues (Allen et al. 2008). Their tree showed for the first time that at least some red algae have HCCS and that most hacrobian groups have two, yet they failed to realise that if host and red algal symbiont each had two, one for each mitochondrial cytochrome c, the ancestral chromist would have had four. Inevitably, this redundancy would lead to random losses of one c- and one c1-specific HCCS as lineages diverged, which would yield four different combinations of ancestral paralogues, so every chromist lineage should have two of the four original paralogues and chromists as a whole would lie on four different paralogue branches on the tree, essentially as Janouškovec et al. (2017) found. Their tree did not fully sample all paralogues for each group as it shows only one paralogue for chlorophytes, choanoflagellates, apicomplexa and diatoms, yet all were known to have two (Allen et al. 2008), so some other groups with only one on their tree may also actually have two. Differential paralogue sorting following chromist-chimaera generated redundancy, not LGT, is likely the major reason for complexity of their HCCS tree for heterokonts and Hacrobia. Treating the five seemingly distinct heterokont clades as evidence for polyphyletic LGTs into heterokonts from other chromists was unwarranted.

We suggest that most chromists have two paralogues because each lineage still uses different paralogues for cytochrome c and c1. Chromist paralogues with slightly shorter branches and sparser taxonomic representation group with the also shorter branch obazoan scotokaryotes (opisthokonts/Amoebozoa); the other paralogues with slightly longer branches group with Plantae as a corticate clade, in which only *Thecamonas* is an intruding scotokaryote. Presence of heterotrophic oomycetes in four places and of cryptists in four (with heterotrophs in three) is best seen as evidence for ancestral chromist chimaerism and plastid loss by oomycetes, goniomonads and kathablepharids, not LGT. The longer branch corticate cluster is highlighted as having four distinct groups but these represent the four different deepest branching heterokont subgroups (Ochrophyta, Pseudofungi, Labyrinthulea, Bicoecia) which often do not group together on single-gene trees and whose correct branching order (more clearly shown than hitherto in their 201-protein tree, including holophyly of phyla Bigyra and Gyrista) is rarely recovered by rDNA, so there being three ‘separate’ clades simply reflects poor basal resolution on all single-gene chromist trees.

Allen et al. (2008) debated whether the c/c1 paralogue divergence occurred in the ancestral neokaryote and was lost by the animal ancestor or evolved polyphyletically. The generality of at least two paralogues in chromists, even in Rhizaria, makes it more likely that HCCS duplication ocurred immediately after this protein originated and was lost independently in animals and perhaps some other groups. More thorough taxon sampling, especially in Rhizaria, red algae, glaucophytes and deep branching scotokaryotes, and biochemical studies to test whether dual paralogues in hacrobians (e.g. *Roombia*, *Guillardia* and *Emiliania* even in the same species) do indeed function separately for c and c1 (as in *Saccharomyces*) should clarify evolution of mitochondrial cytochrome c biogenesis and also have the potential to provide clear evidence from differential paralogue retention that even Rhizaria retain proteins from both the chromist host and red algal symbiont despite retaining no chromophyte plastid. That already appears to be so for the oomycetes (which collectively appear to have three HCCS paralogues, though as expected no one genus has more than two), whose ancestry by plastid loss from an ancestral heterokont eukaryote-eukaryote chimaera has often been doubted. Presence of deep-branching heterotrophic cryptists (*Roombia*, *Goniomonas*) in three different paralogue clades (Janouškovec et al. 2017) is also best explained as differential retention from the ancestral chromist eukaryote-eukaryote chimaera and thus evidences ancient multiple plastid loss in early diverging cryptist lineages. The sole retarian sequence (*Plasmodiophora*) groups strongly with a cryptist sequence (*Goniomonas*), whereas the sole cercozoan one (*Lotharella*) groups strongly with the more distant of the two haptophyte paralogue clusters, suggesting that both rhizarian paralogues share closer common ancestry with hacrobian chromist paralogues than with Plantae or scotokaryotes, consistent with chromist monophyly and no LGT.

Janouškovec et al. (2017) suggested that HCCS arose in the ancestral eukaryote, but their tree instead strongly supports the original idea that it arose in the common ancestral neokaryote (Cavalier-Smith 2010a). Its complete absence in Eozoa except for the likely *Percolomonas* LGT favours absence as the ancestral eukaryote condition, and still favours Eozoa as the earliest branching eukaryotes (Cavalier-Smith 2010a), contrary to Janouškovec et al.’s (2017) interpretation. Ciliates, which have none of the putatively four ancestral chromist paralogues, and *Malawimonas* (both previously known to have the multiprotein prokaryote system like jakobids: Allen et al. 2008) appear to be the only major neokaryote lineages that lost all HCCS, apart from secondary anaerobes like metamonads that lost both cytochromes. (Janouškovec et al. (2017) ought to have excluded the sequence ostensibly from the ciliate *Pseudokeronopsis* (wrongly labelled ‘ochrophytes’) as it is likely contamination from the ciliate’s diatom food not a unique LGT into ciliates.)

### Timing of rhizarian origins and basal radiations

If Harosa are ~ 750 My old (Cavalier-Smith 2013b; Cavalier-Smith et al. 2015a), the proportions of the Fig. 1 tree suggest that Rhizaria may have originated and diverged into Cercozoa and Retaria ~ 620 My ago. This closely fits the earliest fossil evidence as the foraminiferan biomarker 24-*n*-propylcholestane was first found ~ 650 My ago between the two Cryogenian snowball global freezing episodes (Brocks et al. 2017). It is sometimes suggested that eukaryotic algae could not have survived below a kilometre of ice during the ‘snowball earth’ Cryogenian glaciations—Sturtian (717–660 Mya) and Marinoan (640–635 Mya). However, contrary to that assumption, eukaryotic algae, notably chromist diatoms and green algal Plantae within sea ice and glacial ice respectively, survive far better and are much more abundant there than cyanobacteria (Boetius et al. 2015). Moreover, presence of simple macroalgae in Marinoan age rocks (Ye et al. 2015) implies that there were local patches of open sea maintained by volcanic heating at least during the shorter Marinoan snowball.

If the complex phosphatic scales from the Fifteenmile Group that must be at least 717 My old (Cohen and Knoll 2012) are from an extant corticate group—possible but not certain (Cavalier-Smith 2013b suggested they are Viridiplantae), Plantae and Chromista probably originated shortly before the Sturtian glaciation; their origin may even have triggered that glaciation (Feulner et al. 2015; Tziperman et al. 2011). The last Cryogenian glaciation ended ~ 635 My ago (Le Heron 2012; Rooney et al. 2015) so Rhizaria may have originated immediately Snowball Earth melted. If our estimated date for the origin of Rhizaria (~ 620 Mya) is approximately correct, their secondarily heterotrophic harosan ancestors possibly originated by losing the chromist plastid and evolving pseudopodia for a benthic existence below the near-global ice cap at a time when being a benthic predator and consumer of ocean floor detritus may have been more advantageous than an algal mode of life that must have become almost impossible under that global km or more thick ice for scores of millions of years. As the ocean was largely anoxic (Hood and Wallace 2015) but probably well mixed (Ashkenazy et al. 2013), this presumably happened in local exceptional, somewhat separated regions of above average oxygenation, probably shallower zones in volcanic areas of thinner and periodically broken and melted ice that must have existed to allow survival of aerobic protists, and whose genuine existence on ocean margins is independently indicated by local glacier-associated banded iron formations (Guilbaud et al. 2015). However, neither chromists nor scotokaryotes could have become abundant and diversified into most modern groups till after the global thaw and reoxygenation at the beginning of the Ediacara period 635 My ago. No undoubted fossil Rhizaria are known before deep oceanic waters also became oxic after 580 My ago (Canfield et al. 2007). The oldest generally accepted fossil Foraminifera (~ 545 My ago) and Polycystinea (~ 505 My ago) date only from close to the Precambrian/Cambrian boundary (542 My ago) (Groussin et al. 2011; Maletz 2011; Won and Below 1999), which is consistent with the relative time of origins of scotokaryote and corticate deepest lineages on Fig. 1. The 100 My older biomarkers may therefore have come from stem Rhizaria rather than from Foraminifera which are not the deepest branch on the trees.

However, we can interpret Fig. 1 sensibly in relation to geological time only if we contract the excessively long retarian branches so their termini line up with more averagely evolving clades. Without such contraction, basal crown Foraminifera and Polycystinea would appear to have not yet originated (!) in comparison with the pediglissid or phytomyxid clades that have more typical rates of sequence evolution for eukaryotes. Contracting the whole ectoretan branch including its stem uniformly by a factor of two would roughly line up branch termini with those of other Rhizaria. But that would not solve the problem of unequal evolutionary rates, as the base of crown Foraminifera and crown Polycystinea would still be many times younger than the origin of Pediglissa inferred above to be only ~ 350 My ago. However, simply contracting the bare stem of Ectoreta to almost zero would be almost enough to reduce the acantharean branch to the same length as most Endomyxa. Further contractions of the stems alone of Polycystinea and Foraminifera and of their common stem would then be sufficient to bring the base of each fossilogenic group to a date in line with the base of the bilateral animal clade whose origin effectively defines the Precambrian/Cambrian boundary (543 My ago). Thus comparison of our multigene tree with the fossil record for Ectoreta provides direct evidence that rates of protein evolution were episodically many times higher in the stems at the base of major branches than in the later terminal branches after their primary radiations. This conclusion is strongest for Foraminifera as we now have data for all three classes and their base (last common ancestor) is earlier than that for the so-far sequenced polycystines, *Spongosphaera* and *Collozoum*, which probably do not represent the full phylogenetic depth of extant Polycystinea (Gilg et al. 2010; Krabberød et al. 2011), *Collozoum* especially belonging to the rather young order Collodarida (Ishitani et al. 2012).

The final three sections below further discuss timing of the origin and major diversifications and cell innovations of Rhizaria. We emphasise that sequence trees contain information not only about cladistic aspects of phylogeny, upon which most authors focus, but also about historical patterns of innovations and timing, but are liable to misinterpretation unless using fossil evidence critically also. Multiprotein trees are superior for this purpose to rDNA or other single-gene trees, but can be properly understood only by mapping them onto the fossil record and carefully assessing the likelihood of accelerations and decelerations in sequence evolution across the tree, using objective fossil dates as controls.

### Marked novelty, explosive radiations, and molecular coevolution affect sequence rate changes

Darwin (1859) argued that major innovations would immediately cause much more rapid evolutionary radiation than at other times and also stressed under the rubric ‘correlation of growth’ that different organismal features necessarily coevolve, writing ‘when slight variations in any part occur ... other parts become modified’ (Darwin 1859 p. 143). This is highly relevant to multigene trees, because a majority of the 187 proteins in our trees are parts of very few large macromolecular complexes—they are not 187 functionally independent proteins (nor 50,964 independent amino acids). Their ability to bind to each other must be amongst the most important influences on their evolution. As supplementary Table S4 shows, 16 form the proteasome; 43 are part of the large ribosomal subumit; 30 part of the small ribosomal subunit (so must bind specifically to 18S rDNA and/or each other); 2 part of the signal recognition particle (effectively a third ribosomal subunit for secreted/membrane proteins); 13 more function in translation, so several will bind temporarily to ribosomes; and 8 are part of the chaperonin TCP complex. Thus, 88 are ribosome-associated and 24 more with proteasomes or TCP—so these 112 proteins are concerned with the function of just 3 macromolecular complexes, a very narrow range of cell functions all related to the synthesis or proper folding of proteins or their degradation if improperly folded. Many others are parts of smaller macromolecular complexes: microtubules, chaperones like Hsp70 and 90 also related to protein folding so have many hundreds of interactors, or chromatin histones or Mcms, their close associations explaining the coevolutionary constraints on their evolution that generally make them evolve so slowly as to be useful deep phylogenetic markers.

The roughly equal branch lengths in most clades on Fig. 1 probably reflect the normal constraints where mutations changing components of what Mayr called ‘coadapted gene complexes’ too much are removed by purifying selection, but small intrinsically deleterious changes evade such purification if compensated by reciprocal changes in interactor molecules (probably themselves mildly deleterious in the absence of phenotypically corrective suppressor mutations). Such regions of trees appear to evolve in semi-clock-like fashion giving rise to the always extremely oversimplified and sometimes grossly false idea of a ‘molecular clock’. Sometimes, radical changes in adaptive zone cause major changes during protist organismal and molecular evolution, affecting so many genes that genuine ‘genetic revolutions’ (Mayr 1954, 1960) are associated with them: transitions from aerobic to anaerobic life and to parasitism are two such revolutions. When both occur at the same time, the outcome is particularly dramatic. The anaerobic retarian haplosporidian parasite *Mikrocytos* (Burki et al. 2013) and Microsporidia (protozoa related to Fungi) are both anaerobic animal parasites that independently converted their mitochondria to mitosomes, losing their mitochondrial genomes, and miniaturised their cells and reduced their nuclear genome size to a greater extent than any other eukaryotes (both came from ancestors that also had lost cilia and thus also around a thousand genes encoding their proteins). Not surprisingly, both have the longest branches by far on eukaryote multigene trees, so we omitted them from our trees to avoid distorting other branches—such omission is rational and permissible, but it conceals the grossly non-clock-like nature of much protein evolution. In microsporidia at least, not only stems of sequence trees are grossly stretched, but so clearly are many branches. Thus when genomes become unusually small, they can attain equilibrium at permanently elevated evolutionary rates compared with other eukaryotes, and still survive. It is likely that reducing the number of gene product interactions weakens the conservative effect of coadapted gene complexes. Molecular evolutionary rates are immensely more variable and unpredictably erratic than assumed by even the best computer-based ‘molecular clock’ dating efforts (e.g. Eme et al. 2014), which makes them probably less accurate for backward extrapolation than direct careful comparisons of branch lengths on trees with the more reliable fossil ages interpreted critically (e.g. Cavalier-Smith 2013b).

### Multigene trees inform us about macroeveolutionary processes as well as phylogeny

Cavalier-Smith (1980b) argued that rRNA evolved nothing like a molecular clock and that the radical differences between eukaryotic 18S rDNA on the one hand and eubacterial and chloroplast and mitochondrial 16S rRNA are attributable to an episodic short term dramatic increase in the rate of molecular evolution in the ancestor of eukaryotes when the nucleus originated and transcription and translation were first segregated into separate cellular compartments. This idea was developed in more detail subsequently (Cavalier-Smith 1981b, 1987, 2002a, b) in relation to details of the radical transformations during eukaryogenesis, the greatest megaevolutionary destabilisation and restabilisation under a novel structural and adaptive regime in the history of life (Cavalier-Smith 2014). Unfortunately, these arguments are often overlooked or ignored, especially by mathematicians and biochemists specialising in sequence evolution, often too deeply imbued with the seductive but grossly oversimplified idea of a molecular clock, and less familiar with the extensive evidence from fossils and organismic evolution that overall evolutionary change on the macroscale is highly episodic with long periods of relative stasis punctuated by short periods of ultrarapid, so-called quantum evolution (Simpson 1944, 1953; Eldredge and Gould 1972; Gould and Eldredge 1977, 1986; Mayr 1988b). Stackebrandt and Woese (1981) admitted there was no evidence that archaebacterial and eubacterial rDNA has evolved at the same rate, yet their assumption that a uniform rate can be applied not only within their three ‘domains’ of life, but (even more doubtfully) to the stems that connect their bases to one point, has been widely adopted as the core of the erroneous Woesian paradigm of their equal antiquity, always grossly contradicted by the fossil record, as Cavalier-Smith (1981b, 1987, 2002a, b, 2006a, b, 2014) argued in detail—and has never been refuted by serious argument or specific evidence.

We raise this issue here because it is extremely important to proper evolutionary interpretation of multigene trees, and because Fig. 1 provides the first compelling relatively direct evidence for the idea that long bare stems on sequence trees must at least sometimes be explained by relatively short periods of many times more rapid evolution than usual, followed by a relatively quick return to more normal rates. In other words, macroevolutionary ideas of punctuated equilibria and quantum evolution are indeed applicable to molecular sequence evolution as well as to cell and organismal evolution. This important molecular evolutionary conclusion has been evident for decades from 18S rDNA trees; for example Pawlowski and Berney (2003) stressed the episodic nature of stem lineage rDNA evolution of Foraminifera, but its radical contradiction of many prevailing assumptions, especially about the large-scale structure and root of the tree of life (Cavalier-Smith 2014) and how sequence trees should be mapped onto the fossil record, is not widely realised.

Cavalier-Smith et al. (1996) argued that within animals the long stem at the base of Bilateria, and within plants the long stem at the base of florideophyte red algae, are the prime reasons why these branches are so much longer than those of their radiate animal and lower red algal relatives respectively. The good fossil record for bilaterian and radiate animals and floridiophytes necessitates the conclusion for both groups of a temporary elevated and later reversed high rate of sequence evolution. In these cases, unlike the even more dramatic long stem at the base of eukaryotes in rDNA trees, there is no specific cell evolutionary explanation for such an episodic acceleration, so they may be essentially evolutionary accidents.

As similar long bare stems are found at the base of many rDNA trees, Cavalier-Smith et al. (1996) argued that in most cases these are probably better explained by episodic dramatic elevations in rate in a single lineage followed by return to normal low rates subsequently, rather than by a permanent more moderate rate increase that persisted in all daughter lineages. Distinguishing hugely accelerated episodic quantum evolution (frequent on taxonomically broad sequence trees) from a single more permanent evolutionary increase can be difficult, but for lineages with a good fossil record it can be done by critically cross-correlating fossil dates derived from the atomic decay clock that dates igneous rocks with great accuracy (and by interpolation objectively gives necessarily somewhat less accurate dates for interbedded fossiliferous ones) with taxonomically well-sampled phylogenetic trees. The example of Retaria here does this for our protein trees, just as the fossil evidence that eukaryotes are much younger than eubacteria did for the universal small subunit rDNA tree (Cavalier-Smith 1991, 1997, 2002b) and Bilateria and Floridiophytina did for 18S rDNA (Cavalier-Smith et al. 1996).

However, for some even more dramatic rate elevations for rDNA for which no fossils are available, we cannot at present get such direct evidence for episodic quantum evolution; two recent examples from Cercozoa for immensely long branches for 18S rDNA of Helkesida (Cavalier-Smith et al. 2008, 2009; Bass et al. 2016), and *Reticulamoeba* (Bass et al. 2012). Both seem most likely examples of grossly stretched stems, followed in *Reticulamoeba* by no higher rates than usual within the subsequent clades but remaining high at least in the basal three branches in helkesids. Our multiprotein trees show that Helkesimastigoidea also have the longest branch of all Cercozoa, but the evolutionary rate for *Helkesimastix* is much less inflated than for rDNA; this milder branch distortion enabled us to establish their correct position confidently for the first time. Separate trees could be run for the numerous ribosomal proteins and non-ribosomal proteins to test whether coevolution of ribosomal proteins and ultrarapidly evolving rDNA contributed disproportionally to the length of the helkesean branches in Figs. 1, 2, 3 and 4, or whether the acceleration is genome-wide. Cavalier-Smith (2015) discussed numerous other equally extreme examples of clade-specific rDNA accelerated evolution in other phyla, especially Percolozoa and gregarines within Miozoa. As all five cases with a good fossil record support quantum evolution in stem lineages and none supports the more widespread assumption of a single unreversed more moderate increase in rate as the explanation for exceptionally long branches, the idea that stem quantum evolution is the best general explanation of bare stems at the base of ultralong branches (Cavalier-Smith et al. 1996) has held up rather well. We therefore suggest that it is also the most likely explanation for the bare basal stem of the long helkesean branch on the multiprotein trees (Figs. 1, 2, 3 and 4).

### Combining fossil and sequence evidence for the age of Rhizaria

Within Rhizaria, the 200 My discrepancy between the fossil-calibrated clock-inferred date for the origin of Foraminifera (650–920, mean 770 My) and the oldest accepted cell fossils (545 My) (Groussin et al. 2011) (the former markedly lower than an earlier estimate of 690–1150 My (Berney and Pawlowski 2003) suggests that clock estimates may still be inflated, possibly by the long bare stems at the base of most foraminiferan orders—only the minimum 650 My date coincides with the biomarker date. Their trees are also probably topologically distorted by the long unbroken branch of *Reticulomyxa*, which our multiprotein trees show to be more closely related to rotaliids than to miliolids and not to branch well below their common ancestor as their 4-gene trees arguably incorrectly show. However, the age discrepancy would be less if fossil agglutinated tubes dated 635–716 My ago really are Foraminifera (Bosak et al. 2012). Unfortunately, though they resemble extant Foraminifera more than other known protists, we cannot rule out the possibility that they were made by other amoeboid Rhizaria or Amoebozoa, as the later date for the foraminferan biomarker suggests—identifying agglutinated protist fossil tests is problematic (Streng et al. 2005). Such tests tell us when pseudopodia able to manipulate particles to create them evolved, but are often not unambiguously assignable to Rhizaria or Amoebozoa, as noted previously (Cavalier-Smith 2006a, 2013b; Cavalier-Smith et al. 2016). The stem at the base of Rhizaria is longer than for any other major eukaryote group (Figs. 1 and 4) and might indicate temporarily accelerated protein sequence evolution—useful for making monophyly of Rhizaria far easier to recover than for a group like Hacrobia or scotokaryotes with extremely short stems, but possibly distorting apparent relative timing of evolutionary events.

Several recent reviews give inflated dates for the origin of Rhizaria and crown eukaryotes, and ignore arguments for there being no compelling fossil evidence for any crown eukaryotes before ~ 800 My ago (Cavalier-Smith 2002b, 2006a, 2013b). Showing Rhizaria and Amoebozoa as 760 My old in Fig. 1 of Lenton et al. (2015) is presumably based on the assumption that vase-shaped marine fossils belong to these groups, which is questionable for both groups and almost certainly wrong for those misidentified as euglyphids. Not only is the morphological argument for Precambrian euglyphids totally unconvincing (see Cavalier-Smith 2006a, 2013b), but as previously explained euglyphids nest so shallowly in rDNA trees that it is likely that they originated only in the Mesozoic not in the Proterozoic (Fig. S8). Our multiprotein trees, the first with good cercozoan taxon sampling to include a euglyphid (*Paulinella*), are consistent with euglyphids being much younger than Rhizaria and animals.

Unsurprisingly, both Lenton et al. (2015) and Knoll (2014) persist in regarding the possibly 1.1–1.2 Gy old *Bangiomorpha* (Butterfield 2000) as a bangiophyte red alga, despite reasoned morphological arguments against that (Cavalier-Smith 2002b, 2006a; there is no compelling evidence it is not a cyanobacterium) and despite arguments from sequence trees that such an identification/age is highly discordant with other evidence (Berney and Pawlowski 2006; Eme et al. 2014), as earlier trenchantly stressed for rDNA trees (Cavalier-Smith 2002b). For both *Bangiomorpha* and the claimed 760 My ‘euglyphids’, the discrepancy is two-fold, much too great to justify it being repeatedly ignored. The arguments of Feulner et al. (2015) for a temporal and hypothetically causal link between eukaryote algal origins and the Sturtian glaciation are weakened by their Fig. 1 exclusive comparison with a multigene ‘clock’ study (Parfrey et al. 2011) that probably erroneously placed their origin hundreds of millions of years earlier than did the best analyses of Berney and Pawlowski (2006) and Eme et al. (2014) and discordantly gave a crown eukaryote ‘date’ twice as old. That gross discrepancy arises only partly because Parfrey et al. (2011) ignored the valid criticisms of Berney and Pawlowski (2006) over widespread use of doubtful Proterozoic ‘calibrations’, unwisely using more than earlier studies: six of their seven earliest fossil ‘calibrations’ are highly dubious, arguably all substantially too early. One is *Bangiomorpha*; the other five are: claimed sponge biomarkers probably are not specific (Antcliffe 2013; see also Cavalier-Smith 2017); *Palaeoarcella*, unlikely actually to be arcellinid (see Cavalier-Smith 2013b; Cavalier-Smith 2015); *Kimberella* that may not be a bilaterian (Brasier 2009); *Palaeastrum* is not obviously a green alga, and could be another eukaryote or a cyanobacterium (as could other claimed green algae in the same reference); gammacerane is not a reliable biomarker for ciliates, as it is also made by proteobacteria (Cavalier-Smith 2006a; Kleemann et al. 1990) as well as by a fungus and a fern. Eme et al. (2014) rejected the ciliate calibration but retained the others; they found that using soft bounds for calibration points that can allow the programme to overide discordant wrongly dated/identified fossils gives markedly younger dates for most models but noted that the autocorrelated lognormal relaxed clock model is insensitive to whether calibration is soft or hard and also gives the lowest ages, most consistent with the fossil record. Eme et al. (2014) noted: ‘all recent molecular clock analyses seem to concur that the *Bangiomorpha* fossil date or taxonomic assignment is problematic’. The Parfrey et al. trees used only 3–15 genes, not 159 as in the more thorough Eme et al. (2014) analysis; primarily used an uncorrelated clock model (less accurate than correlated ones: Lepage et al. 2007); and inferior site-homogeneous models (often not even including gamma distribution intersite rate variation); and thus were necessarily less reliable, quite apart from almost all earliest ‘calibrations’ systematically biasing their scale: omitting them reduced most clade dates only about 200–300 My. The age conflicts amongst their methods covered a 2–3-fold range, making conclusions virtually meaningless. Eme et al. showed that uncorrelated clock models and site-homogeneous models (used by Parfrey et al. and most other investigators) can systematically inflate inferred ages by hundreds of millions of years and have severalfold wider error bars than trees using an autocorrelated lognormal clock model that yields much younger dates closer to those of unambiguously identified fossils, and that the CIR method for PhyloBayes (the only autocorrelated model used by Parfrey et al.) yields dates that are similarly much older than by the lognormal model even though under simulation CIR appeared as reliable (Lepage et al. 2007).

This suggests that for real data, a lognormal relaxed clock model may be greatly superior, but it is still unlikely that any statistical model can reliably reflect unique evolutionary events like the grossly non-clock like phenomenon of idiosyncratic episodically dramatically increased rates discussed above. Neither analysis included any Retaria (the Rhizaria with the best fossil record), as they too blatantly infringe the assumption of a clock. Figure 2 of Parfrey et al. inferred far too early dates for Cercozoa (1100 My) and Harosa/haptophytes (1600 My—older than that inferred for red algae despite using an inflated ‘calibration’ for the latter), and incorrectly claimed that their analysis was congruent with the fossil record; their Fig. 2 shows all major crown eukaryote clades older than the dates of any unambiguous fossils, in many cases 2–3 times as old! Corticates are shown as ~ 1650 My old, whereas the oldest acceptable body fossil corticate calibration point was 550 My (Doushanto red algae) a 1.1 billion year discrepancy—the error was double the size of the signal. Omitting the dubious Proterozoic calibrations (their Fig. S1) reduced inferred ages for Cercozoa to 900 My, Harosa/haptophytes to 1250 My and corticates to 1350 My, but all deep nodes were still seriously inflated by unrealistic models. Yang et al. (2016) also grossly overestimated corticate age as ~ 1693 My, red algae as ~ 1500 My and subphylum Eurhodophytina (likely sisters of chromist nucleomorphs) at ~ 1194 My. Post-Marinoan steranes ~ 622 ± 20 Mya have reasonably been interpreted as produced by a red algal plankton bloom (Elie et al. 2007), but if early chromists had similar steranes to red algae, they might be from early chromophytes. Absence of detectable stigmastane or ergostane before ~ 650 My ago (Brocks et al. 2017) shows that green or red algae were not abundant before then, making it unlikely that the slightly deeper-branching Glaucophyta and therefore chloroplasts and Plantae evolved substantially before the beginning of the Sturtian glaciation ~ 717 My ago. If the *Bangiomorpha* fossils are little older than their minimum inferred date of ~ 722 Mya, as Yang et al. (2016) recognise is likely, their original identification as eukaryotic would be less problematic than if they were 1200 My old as claimed for decades, but even if they are eukaryotic (which their morphology allows but does not require), there is no reason to consider them as Bangiophyceae rather than an earlier-branching group of red algae, e.g. Compsopogonophyceae or Stylonematophyceae with analogous filaments, or as an extinct group of stem Plantae. Using *Bangiomorpha* to ‘calibrate’ sequence trees when its identity is unknown and its date uncertain by 500 My is ridiculous (Cavalier-Smith 2002b). There is no good reason to think that red algae or chromists originated before ~ 750 Mya; both could be more recent.

Fossil-calibrated dates of Eme et al. (2014) using site-heterogeneous phylogenetic and lognormal clock models for Plantae, Hacrobia and Harosa (~ 900 My ago) agree more than the contradictory Parfrey et al. (2011) trees with a reasonable statement of Feulner et al. (2015) that the first major eukaryote algal radiation was around 800 My ago, but even 900 My could be somewhat inflated for reasons noted above, steranes suggesting ~ 650 My only. All palaeontologists agree there was a major eukaryotic radiation 800–750 My ago of more complex fossils than previously (e.g. Butterfield 2015; Knoll 2014); we now suggest this reflects basal radiation of the scotokaryote and corticate eukaryote clades (Cavalier-Smith 2013b; Cavalier-Smith et al. 2015b) collectively denoted neokaryotes (Cavalier-Smith 2018), not the very first eukaryote radiation as Cavalier-Smith (2002b, 2006a) had argued before concluding that the eukaryote root probably lies within Eozoa, making them somewhat older than corticates and scotokaryotes (Cavalier-Smith 2010a). The now more critical sterane biomarker work increasingly suggests that the first major rise of eukaryote algal abundance did not occur before ~ 650 My in the interglacial between the Sturtian and Marinoan glaciations (Brocks et al. 2017). Comprehensive fossil-calibrated site-heterogeneous multigene trees place excavates and the crown eukaryote root close to 1 Gy (Eme et al. 2014 Fig. 4), which closely fits the thesis that the putatively basal eukaryote supergroup Eozoa is no older than ~ 1 Gy and that no fossils older than 1.1–1.2 Gy provide significant evidence even for stem eukaryotes (Cavalier-Smith 2013b), which assumed that the eukaryote root was within Eozoa.

However, if the root of the eukaryote tree were between Eozoa and neokaryotes (all eukaryotes except Euglenozoa, Percolozoa and Eolouka: Cavalier-Smith 2018) not within Eozoa, then crown eukaryotes might be no older than 800–850 My as previously argued (Cavalier-Smith 2002b, 2006a); so far, the oldest biomarker age for archaebacteria (likely sisters of eukaryotes, not their ancestors: Cavalier-Smith 2014) is ~ 820 My (Schintele and Brocks 2017) but earlier evidence is wanting. Irrespective of the position of the root of the eukaryotic tree, it is likely that Rhizaria are substantially more than half as old as eukaryotes and could be over three quarters of their age, significantly older than animals and other opisthokonts.

### Twenty-five major conclusions

1. Classical Retaria, recently made new subphylum Ectoreta, generally branch as sisters to Endomyxa, and are probably not sisters of Gromiidea alone; the recent transfer of Endomyxa from Cercozoa to Retaria fits their shared reticulose pseudopodia and absence of biciliate trophic phase.
2. Radiozoa is a clade on most trees; their occasional apparent paraphyly with only Polycystinea sister to Foraminifera may be a sparse sampling artefact.
3. Endomyxa are a clade, contrary to some multigene trees.
4. Monadofilosa are a clade but Reticulofilosa are probably paraphyletic with Chlorarachnea (not Granofilosea as some 18S rDNA trees suggested) sister to Monadofilosa.
5. Metromonadea are the deepest branching monadofilosan class, probaby a clade.
6. The non-ciliate non-filose amoeba *Guttulinopsis* is sister to the essentially non-amoeboid uniciliate gliding zooflagellate *Helkesimastix* (clade Helkesida). Episodic dramatic increases in sequence evolutionary rates in stem helkesids affected both rDNA and most protein-coding genes.
7. Thecofilosea (including ventricleftids as the most divergent lineage) are probably a clade.
8. Cercomonads are probably polyphyletic, so their suborders are made separate orders: Cercomonadida and Paracercomonadida.
9. Cercomonadida are sisters of Glissomonadida, forming a robust clade of gliding soil flagellates here made new sarcomonad subclass Pediglissa.
10. Pediglissa are probably sister to euglyphids plus Nudisarca, not to Paracercomonadida, which are therefore assigned to a separate new sarcomonad subclass Paracercomonadia.
11. Imbricatea appear to be nested within Sarcomonadea; subclasses Placonuda and Placoperla are possibly not sisters.
12. The earlier possibility that scaly, filoreticulose *Kraken* are sister to paracercomonads is not supported by site-heterogeneous rDNA trees or ultrastructure, so we establish new order Krakenida for them, grouping them with environmental DNA clade eSarcomonad as new subclass Krakenia within possibly ancestrally scaly Imbricatea.
13. The position of *Thaumatomonas* (Placoperla) is inconsistent on multiprotein trees, but on trees without distant outgroups it is within clade Monadofilosa and sister to Placonuda (here revised by adding Discomonadida).
14. As protein trees suggest that Sarcomonadea/Imbricatea are a sister clade to Thecofilosea, we transferred Sarcomonadea from the basal monadofilosan superclass Eoglissa to derived superclass Ventrifilosa to make Ventrifilosa a robustly supported, ancestrally filose, clade. This also makes Eoglissa phenotypically more homogeneous as it now contains only the non-filose Metromonadea plus Helkesea.
15. Some transcriptome sequences of ‘*Minchinia*’ sp. are not from *Minchinia* (an endomyxan haplosporidian) but from contaminants; after eliminating human, green plant and diatom contaminants, the residue group on trees as a distant relative of core Cryptista; instead of being a previously unknown deep-branching hacrobian lineage close to *Palpitomonas*, it is more likely to represent a mixture of a cryptist and/or other contaminants and *Minchinia* genes that collectively group in that position on multiprotein trees.
16. New strongly holophyletic superclasses Proteomyxia and Marimyxia are established for Endomyxa.
17. Endomyxan *Filoreta marina* has both actin1 and actin2, suggesting that this gene duplication occurred earlier than previously thought and that actin2 may contribute to the reticulose body form of all Retaria, not just Ectoreta, providing the first molecular synapomorphy for the now expanded Retaria.
18. Combining fossil evidence for Cambrian Foraminifera with the remarkable length of retarian and especially foraminiferan branches on multigene trees, and cross-correlation of dates with outgroups provides strong evidence for episodic dramatic increases in molecular evolutionary rates in stem lineages of both Ectoreta and Foraminifera, with similarly large rate reductions to relatively normal levels within crown Foraminifera.
19. Similar episodically grossly accelerated evolutionary rates in stem lineages are probably the major cause of long bare stems at the base of ultralong branches on sequence trees; we discuss their implications for fossil-calibrated ‘relaxed-clock’ statistical phylogenetic estimates of extrapolated dates for the origins of deep-branching clades like Retaria.
20. Patterns of evolution on multigene trees provide evidence for extensive molecular coevolution; these make it unrealistic to consider molecular evolution solely in terms of individual molecules and illuminate debates about megaevolution and quantum evolution
21. We discuss the origins of Rhizaria and the contrasting phenotypes that arose during their earliest divergences, including the evolutionary significance of the basic contrast between the recently revised phyla (trophically reticulose non-flagellate Retaria; largely filose predominently gliding flagellate Cercozoa) and within Retaria between subphylum Endomyxa without and subphylum Ectoreta (Foraminifera, Radiozoa) with marked segregation by tests or capsules of endoplasm and reticulose ectoplasm.
22. Evidence that the alveid biciliate *Ancoracysta* is sister to other Haptista and has extensive cortical alveoli and a posterior ciliary vane and simple anterior ciliary hairs and a ventral feeding groove (similarly to the alveolate *Colponema*) strengthens the idea that these characters were all present in the common ancestor of both chromistan subkingdoms Harosa and Hacrobia and also in the stem lineage from which infrakingdom Rhizaria evolved. Thus Cercozoa likely lost the ancestral neokaryote ventral ciliary vane when they evolved posterior ciliary gliding, just as happened convergently in ancestral Sulcozoa. We correct misinterpretations of *Ancoracysta* ultrastructure.
23. Rhizaria and other chromists have at least two paralogues of the unimolecular neokaryote c-cytochrome lyase suggesting that one is used for cytochrome c, the other for c1. Their distribution in four clades across the sequence tree probably results from differential retention and loss from the four paralogues deduced to have been present in the ancestral chromist eukaryote-eukaryote chimaera.
24. Cercozoa and Retaria have non-homologous insertions in ribosomal protein L1 which show each is a clade; contrary to previous ideas, there is no shared rhizarian insertion.
25. Contamination by foreign protists is much commoner than often realised in rhizarian samples, especially uncultured ones, so more effort is needed to remove it before analysing sequences.

## Supplementary data

Supplementary text, figures and tables associated with this article can be found in the online version. ESM 1 (PDF 1443 kb).
