# Supplementary material for: Multigene phylogeny and cell evolution of chromist infrakingdom Rhizaria: contrasting cell organisation of sister phyla Cercozoa and Retaria
Source: Protoplasma. 2018 Apr 17;255(5):1517–74. doi: 10.1007/s00709-018-1241-1 (PMC6133090; doi:10.1007/s00709-018-1241-1)
Supplement: ESM 1 — (PDF 1443 kb). [file 709_2018_1241_MOESM1_ESM.pdf]

Supplementary Material For:

Multigene phylogeny and cell evolution of chromist infrakingdom Rhizaria: monophyly and internal deep phylogeny of phyla Cercozoa and Retaria having contrasting cell organisation

Thomas Cavalier-Smith, Ema E. Chao, and Rhodri Lewis

Department of Zoology, University of Oxford, South Parks Road, Oxford OX1 3PS, UK.

CONTENTS:

Culturing, RNA extraction, and c-DNA library preparation
Whole genome amplification protocol for *Rhogostoma minus*
Supplementary Fig. S1. PhyloBayes CAT GAMMA 4 rate PhyloBayes phylogeny for 187 proteins and 158 taxa
Supplementary Fig. S2. RAxML LGF GAMMA 4 rate tree for 187 proteins for 72 chromists
Supplementary Fig. S3. RAxML LGF GAMMA 4 rate tree for 187 proteins for 75 chromists
Supplementary Fig. S4. RAxML LGF GAMMA 4 rate tree for 187 proteins for 74 chromists
Supplementary Fig. S5. PhyloBayes CAT GAMMA 4 rate PhyloBayes CAT phylogeny for 187 proteins and 31 Rhizaria
Supplementary Fig. S6. PhyloBayes CAT GAMMA 4 rate PhyloBayes phylogeny for 187 proteins and 32 Rhizaria: *Rhogostoma* added
Supplementary Fig. S7. PhyloBayes CAT GAMMA 4 rate PhyloBayes phylogeny for 187 proteins and 30 Rhizaria: *Limnofila* excluded
Supplementary Fig. S8. 18S rDNA PhyloBayes CAT phylogeny for 467 Rhizaria
Supplementary Fig. S9. 18S rDNA RAxML phylogeny for 467 Rhizaria
Supplementary Fig. S10. 18S rDNA PhyloBayes CAT phylogeny for 465 Rhizaria (excluding two longest branch Helkesida)
Supplementary Fig. S11. 18S rDNA PhyloBayes CAT phylogeny for 464 Rhizaria (excluding all three Helkesida)
Supplementary Fig. S12. 18S rDNA PhyloBayes CAT phylogeny for 316 monadofilosan Cercozoa including helkesid *Cholamonas*
Supplementary Fig. S13. 18S rDNA PhyloBayes CAT phylogeny for 315 monadofilosan Cercozoa excluding helkesid *Cholamonas*
Supplementary Fig. S14. 18S rDNA RAxML phylogeny for 316 monadofilosan Cercozoa including helkesid *Cholamonas*

Supplementary Fig. S15. 18S rDNA RAxML phylogeny for 315 monadofilosan Cercozoa excluding helkesid *Cholamonas*

Discussion on 18S rDNA trees (Figs S8-15)

Table S1 Sequence reads and genes recovered for each species

Table S2 Key to taxon names and abbreviations for sequences and composites

Table S3 Gene representation for the 10 species

Table S4 Gene abbreviations for Table S3

***Filoreta marina* actin sequences**

****

Culturing, RNA extraction, and cDNA library preparation

Mono-protist cultures (bi-protist in the case of *Micrometopion* and *Minimassisteria*) were grown and maintained as in the works that originally described them (references cited in Materials & Methods, Section 2, of the main text):

For *Cercomonas and Thaumatomonas*: in order to harvest RNA, the barley grains were removed, the bulk of the culture medium decanted, and the biofilm layer on the bottom of the Petri dish (together with any residual medium) scraped into pre-chilled 15 ml centrifuge tubes. Cells were pelleted using a bench top centrifuge at 3000 rpm for 25-30 min at 5°C and total RNA extracted as detailed in the supplementary material of Cavalier-Smith et al. (2015).

For *Helkesimastix* (grown in ASW1.0 with the addition of diluted LEMCO broth) and *Micrometopion* (with *Procryptobia*): cells were harvested as described above but spun down in pre-chilled 1.5 ml microcentrifuge tubes at 127,000 g for 32 sec at room temperature. Total RNA was then extracted from the cell pellets as above.

For *Nudifila* and *Sandona*: cultures were harvested as above but cells were spun down in pre-chilled 1.5ml microcentrifuge tubes at 112,000g for 10 minutes, and the cell pellet was resuspended in RNAlater solution (Ambion) according to the manufacturer’s instructions.

As *Minimassisteria* was co-cultured with the centroheliozoan *Oxnerella* (as explained in the main text and by Cavalier-Smith & Chao 2012 and Howe et al. 2011); culturing and RNA extraction is fully described in Cavalier-Smith et al. (2015).

For all cercozoan (=filosan) samples mRNA isolation, cDNA library construction, and 454-pyrosequencing were performed as detailed in Cavalier-Smith et al. (2014). cDNA libraries for *Thaumatomonas* and *Sandona* were normalized (procedure given in Cavalier-Smith et al. 2014).

For *Filoreta*: a mono-protist culture of *Filoreta marina* was grown in 50% volvic/50% ASW (1.0) medium with the addition of 4 half barley grains (to provide nutrients for the bacterial food of *Filoreta*). Total RNA was extracted by the same method as for *Cercomonas*/*Thaumatomonas* and was shipped to Agencourt Bioscience (Beverly, MA, USA) for mRNA selection and cDNA library

construction. The cDNA library was constructed according to their “microquantity cDNA synthesis protocol”. mRNA was purified from total RNA samples using paramagnetic beads coated with poly-d(T) fragments. cDNA synthesized from the mRNA was size-selected by agarose gel electrophoresis and cloned into the pAGEN-1 vector (Not I - Eco RV) to create a directional library within DH10B *E.coli* cells, T1 phage resistant. 93% of clones within the library contained inserts with an average length of 1.2kb. Agencourt Bioscience also provided conventional directional 5’-end (using an M13R primer) Sanger sequencing of the completed cDNA library. A total of 4992 reads were sequenced with a 96-97% success rate; 4507 of these reads were submitted to dbEST with an average length of 755bp. Individual *Filoreta* EST reads were labeled, trimmed, assembled and added to the alignments using the methods described in Cavalier-Smith et al. 2014.

Whole genome amplification protocol for *Rhogostoma minus*

A mono-protist culture of *Rhogostoma minus* was maintained as in Howe et al. (2011). To try to maximize the proportion of *Rhogostoma* DNA within the sample, a fresh culture of cells was serially diluted and then further diluted under a microscope in small droplets of sterile medium (Volvic mineral water) using a P2 micropipette. The *Rhogostoma* cells were progressively diluted between water droplets to reduce the number of bacteria present (in the original culture as food for *Rhogostoma*) compared with the original culture. The final whole drop (approx. 1µl, containing 1-5 *Rhogostoma* cells, together with a mixture of associated bacteria, remaining in the residual culture medium) was then transferred directly into pre-chilled sample buffer from a GenomiPhi kit (version 1) (GE Healthcare), mixed by pipetting and the sample treated according to the manufacturer’s instructions. In order to create additional material, the amplified products were used as starting material for multiple second round reactions which were then pooled together. As the *Phi29* DNA polymerase in the GenomiPhi kit creates a branched DNA network, the sample was de-branched using S1 nuclease (Invitrogen) (as recommended by Liverpool AGF), then purified using a MinElute Enzyme Reaction Cleanup Kit (Qiagen) following the manufacturer’s instructions. 3.4µg of the resulting DNA was used for pyrosequencing as detailed in Cavalier-Smith et al. 2014.

References:

Cavalier-Smith, T., Chao, E.E., 2012. *Oxnerella micra* sp. n. (Oxnerellidae fam. n.), a tiny naked centrohelid, and the diversity and evolution of Heliozoa. Protist 163, 574-601.

Cavalier-Smith, T., Chao, E.E., Snell, E.A., Berney, C., Fiore-Donno, A.M., Lewis, R., 2014. Multigene eukaryote phylogeny reveals the likely protozoan ancestors of opisthokonts (animals, fungi, choanozoans) and Amoebozoa. Mol. Phylog. Evol. 81, 71-85.

Cavalier-Smith, T., Chao, E.E., Lewis, R., 2015. Multiple origins of Heliozoa from flagellate ancestors:

new cryptist subphylum Corbihelia, superclass Corbistoma, and monophyly of Haptista, Cryptista, Hacrobia and Chromista. Mol. Phylog. Evol. 93, 331-362.

Howe, A.T., Bass, D., Scoble, J.M. Lewis, R., Vickerman, K., Arndt, H., Cavalier-Smith, T., 2011. Novel cultured protists identify deep-branching environmental DNA clades of Cercozoa: new genera *Tremula*, *Micrometopion*, *Minimassisteria*, *Nudifila*, *Peregrinia.* Protist 162, 332-372.

Supplementary Fig. S1. PhyloBayes CAT GAMMA 4 rate PhyloBayes phylogeny for 187 proteins and 158 taxa (on next page). Black arrows show new rhizarian sequences and red arrows the two non-rhizarian sequences (*Oxnerella* and *Procryptobia*) from two mixed cultures that we separated phylogenetically. Numbers after species names show how many amino acids were included for each. Consensus ttree for two chains (max.diff. 1; 5,735 trees summed for two chains after removing 1,674 as burnin). Most bipartitions had maximal support (1); posterior probabilities are only shown if they did not – in red for the 11 for which both chains did not show the same topology. The scale bar represents 0.4 substitutions per site.

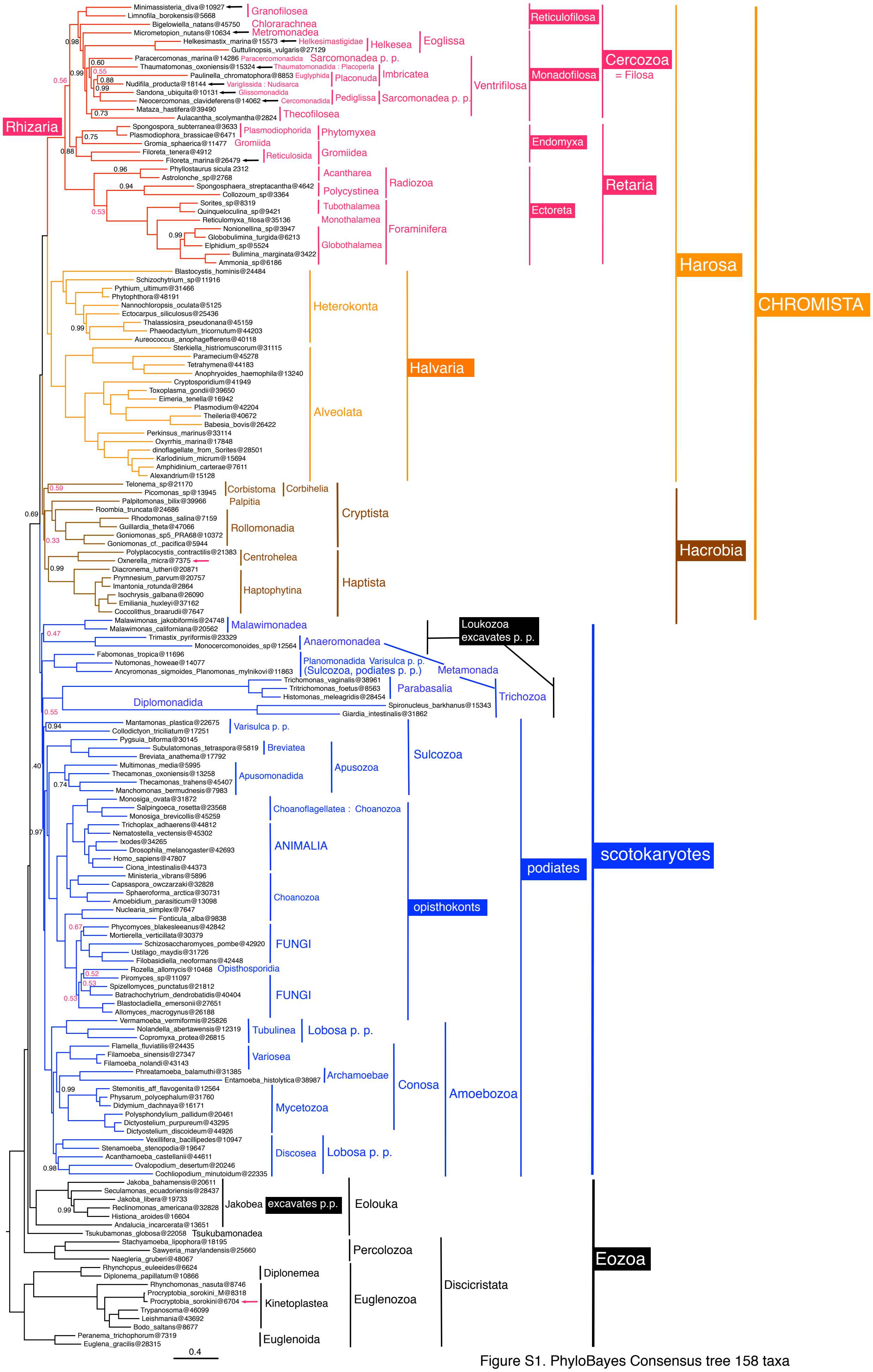

Figure S1. PhyloBayes Consensus tree 158 taxa

<u>Supplementary Fig. S2.</u> RAxML LGF GAMMA 4 rate-classes for 187 proteins for 72 chromists. The ML tree corresponding to the well converged CAT tree in Fig. 2. Support values are for 100 fast bootstraps; the scale bar represents 0.2 substitutions per site:

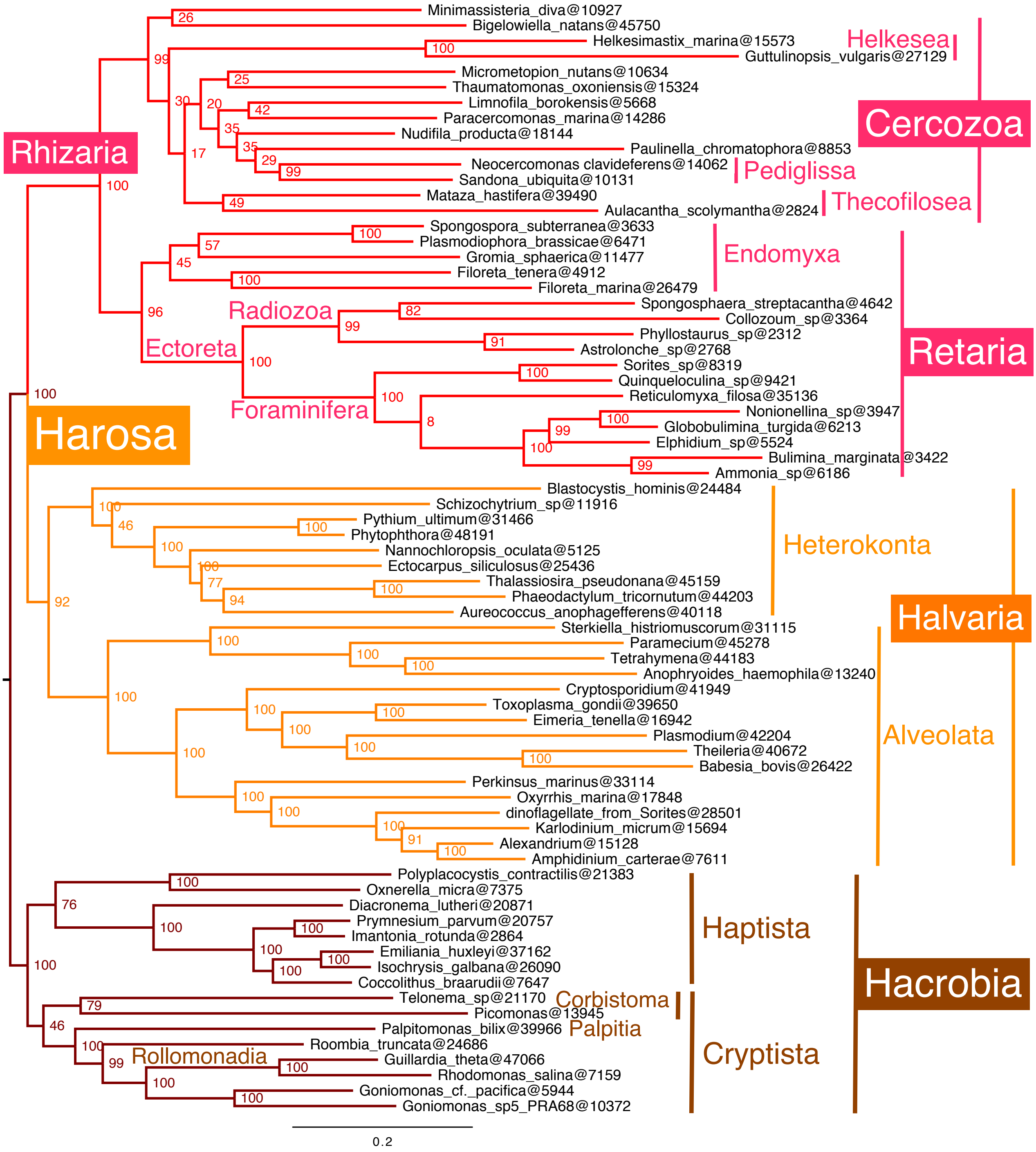

<u>Supplementary Fig. S3.</u> RAxML LGF GAMMA 4 rate-classess for 187 proteins for 75 chromists. This is the ML tree corresponding to the well consensus PhyloBayes CAT tree in Fig. 3 showing support for 100 fast bootstraps. The scale bar represents 0.2 substitutions per site. *Cercomonas clavideferens* is now called *Neocercomonas clavideferens*. Note that bootstrap support drops from 100 to 84% for the Hacrobia/Harosa bipartition compared with Fig. S2, consistent with the '*Minchinia*' sequences being a mixture of a contaminant cryptist and some genuine (i.e. harosan) genes as explained in the text:

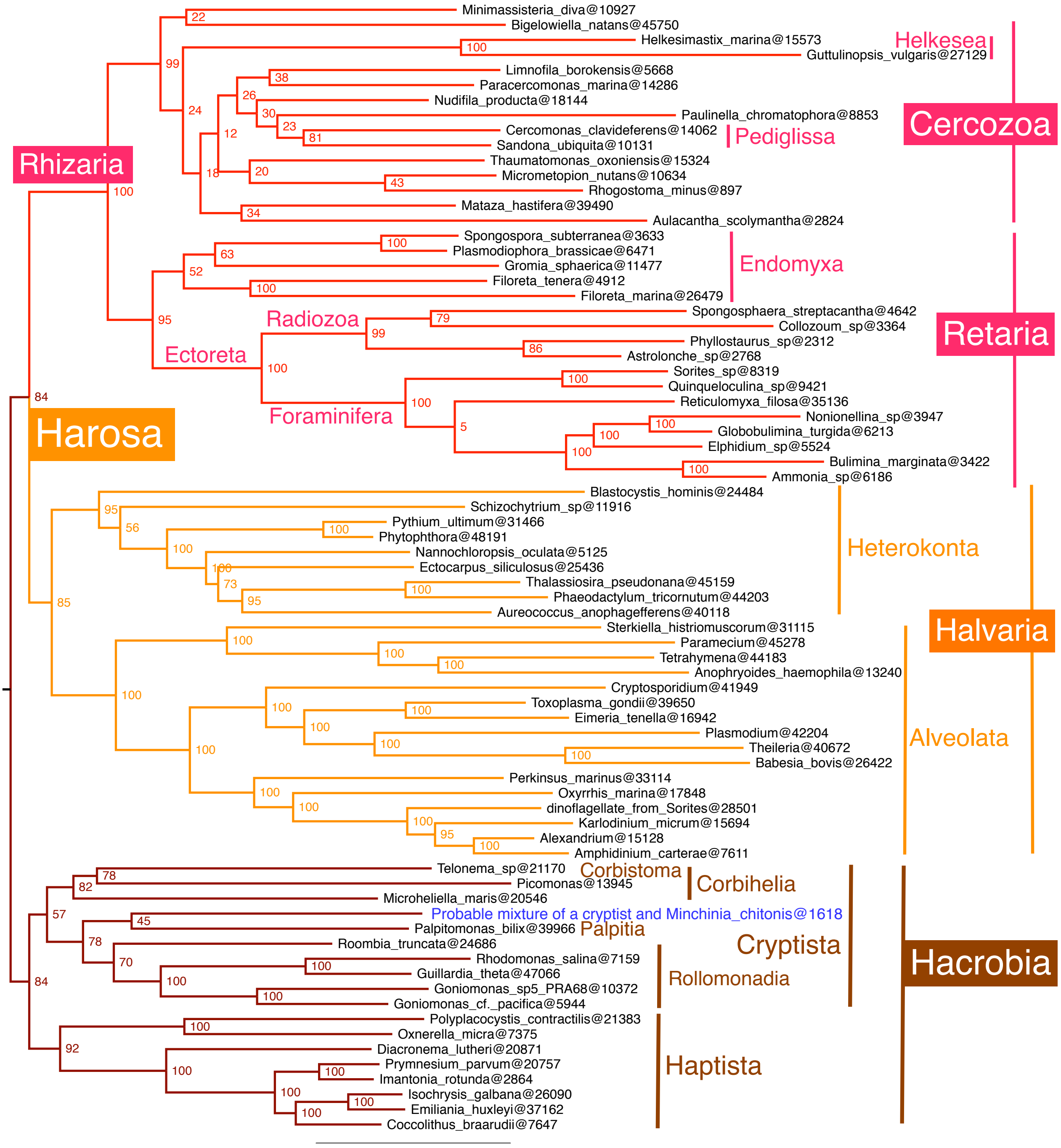

<u>Supplementary Fig. S4.</u> RAxML LGF GAMMA 4 rate tree for 187 proteins for 74 chromists. This ML tree uses the same method as Fig, S3 and differs only by excluding the corbihelian microhelid cryptist *Microheliella* (as did Burki et al. 2016). In marked contrast to Burki et al. (2013, 2016), but as in Cavalier-Smith et al. (2015) *Telonema* and *Picomonas* group together as a fairly strongly supported Corbistoma clade that is weakly sister to the other Cryptista (Palpitia plus Rollomonadia). That might partly be because we added numerous sequences for *Telonema* and *Picomonas* not included by Burki et al. (2013). Comparison of Figs S2 and S4 without *Microheliella* with Fig. S3 that included *Microheliella* shows that excluding *Microheliella* (as in Burki et al. 2016) also reduces support for the Corbihelia clade and for clades Cryptista *sensu* Cavalier-Smith et al. (2015) and Haptista, and to a lesser extent for the Hacrobia/Harosa bipartition.

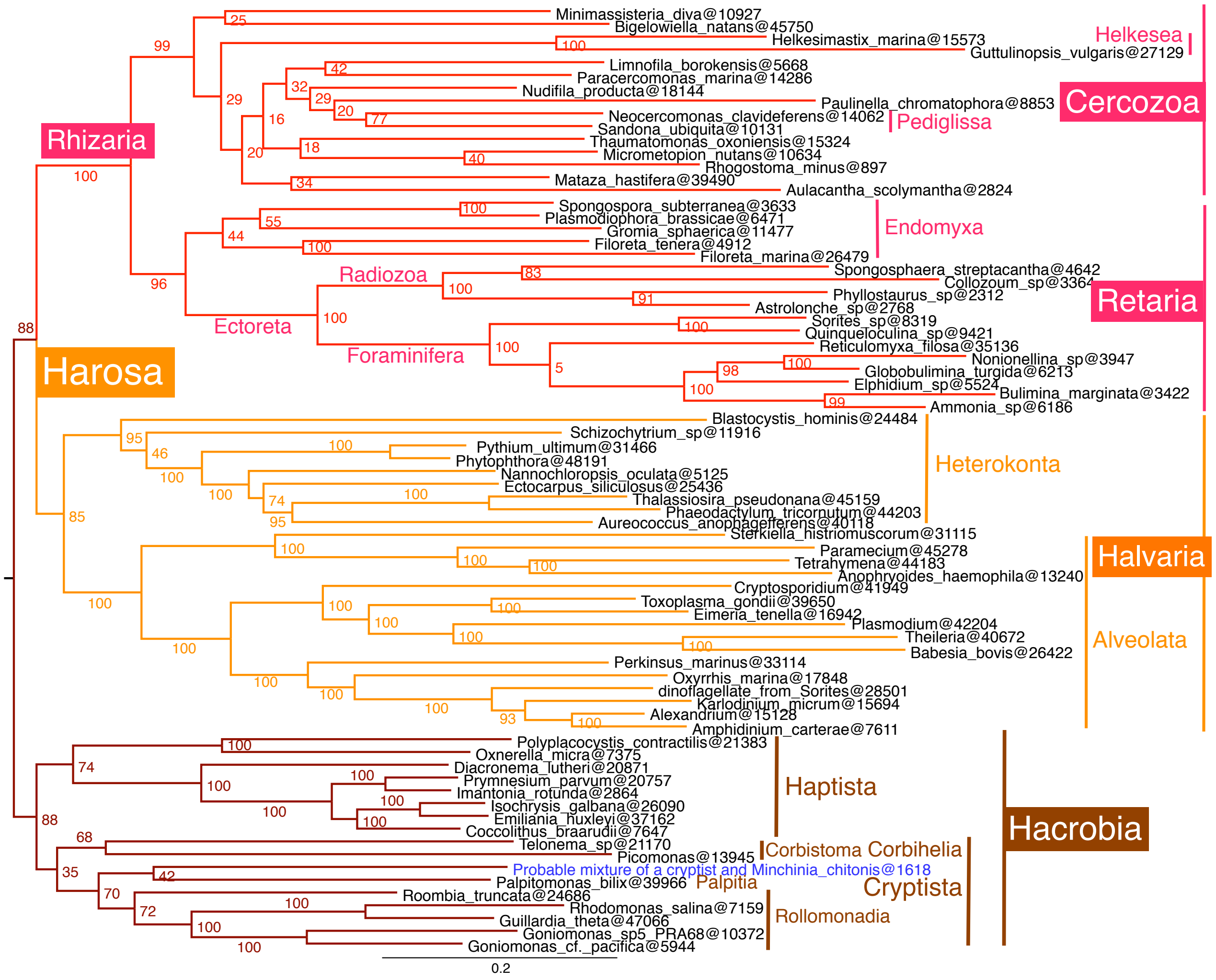

<u>**Supplementary Fig. S5.**</u> **PhyloBayes CAT GAMMA 4 rate PhyloBayes phylogeny for 187 proteins and 31 Rhizaria.** Rhizarian tree rooted between Cercozoa and Retaria as shown by Figs 2, 3, and S1-3 with close outgroups. Note that in this consensus tree for two chains (maxdiff 0.290668; topology identical in both chains) tree and in Fig. S5 although the bipartition between Cercozoa and Retaria is maximally supported there are some marked differences in basal branching order within the two phyla compared with trees that include halvarian and hacrobian outgroups (Figs 2, 3, S1-3). In principle including such outgroups should provide more information for algorithms to reconstruct ancestral sites more accurately so ought to be more evolutionarily correct so long as outgroups are not too distant or excessively long-branches. *Cercomonas clavideferens* is

now called *Neocercomonas clavideferens*. Scale bar is 0.3 substitutions per site.

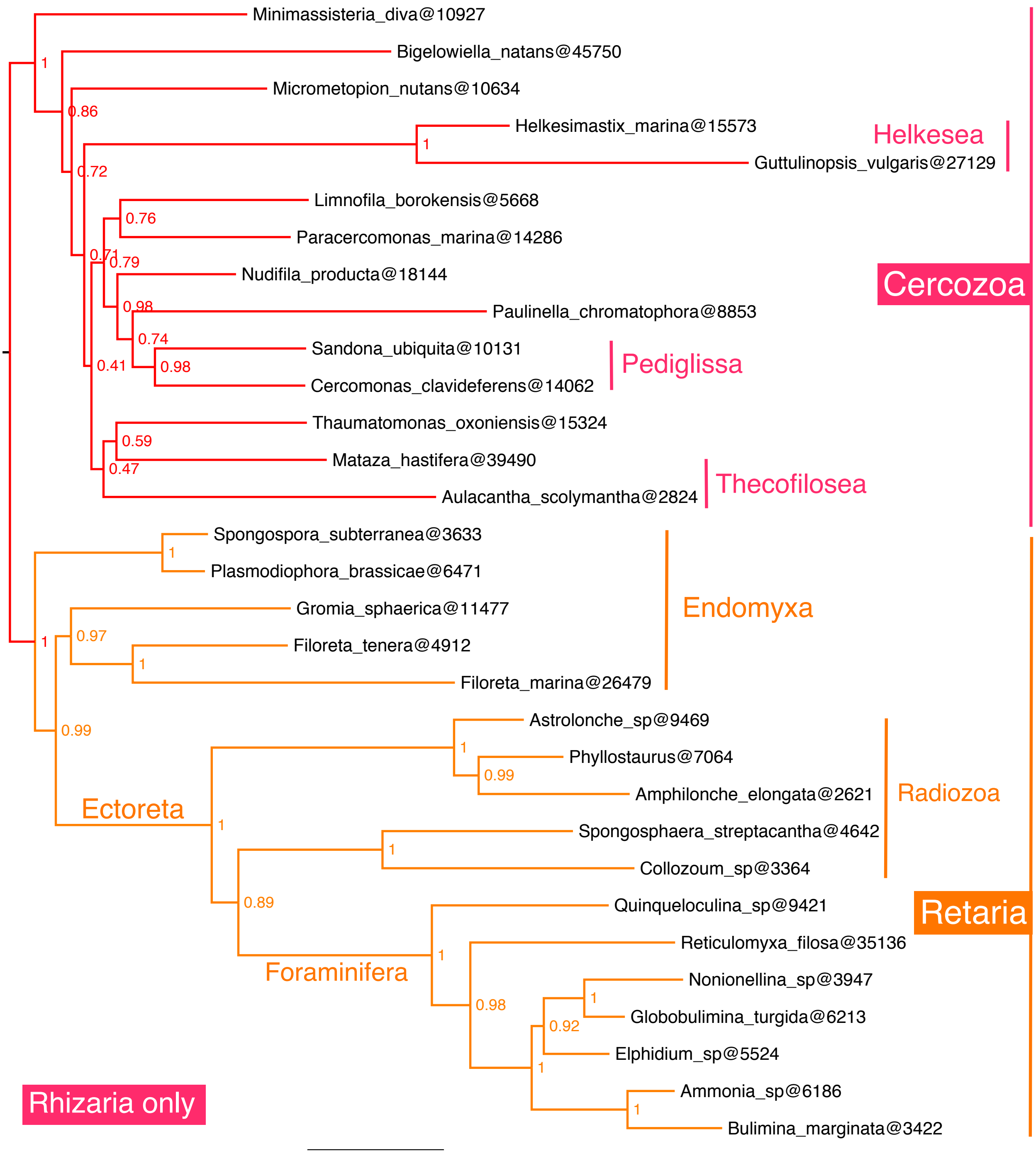

<u>Supplementary Fig. S6.</u> PhyloBayes CAT GAMMA 4 rate PhyloBayes phylogeny for 187 proteins and 32 Rhizaria. Consensus tree for two chains (maxdiff 0.278303). Scale bar is 0.3 substitutions per site. As for Fig. S4 except for addition of *Rhogostom*a which makes Thecofilosea a clade despite its extremely limited gene sampling. *Cercomonas clavideferens* is now called *Neocercomonas clavideferens*. **Next page:**

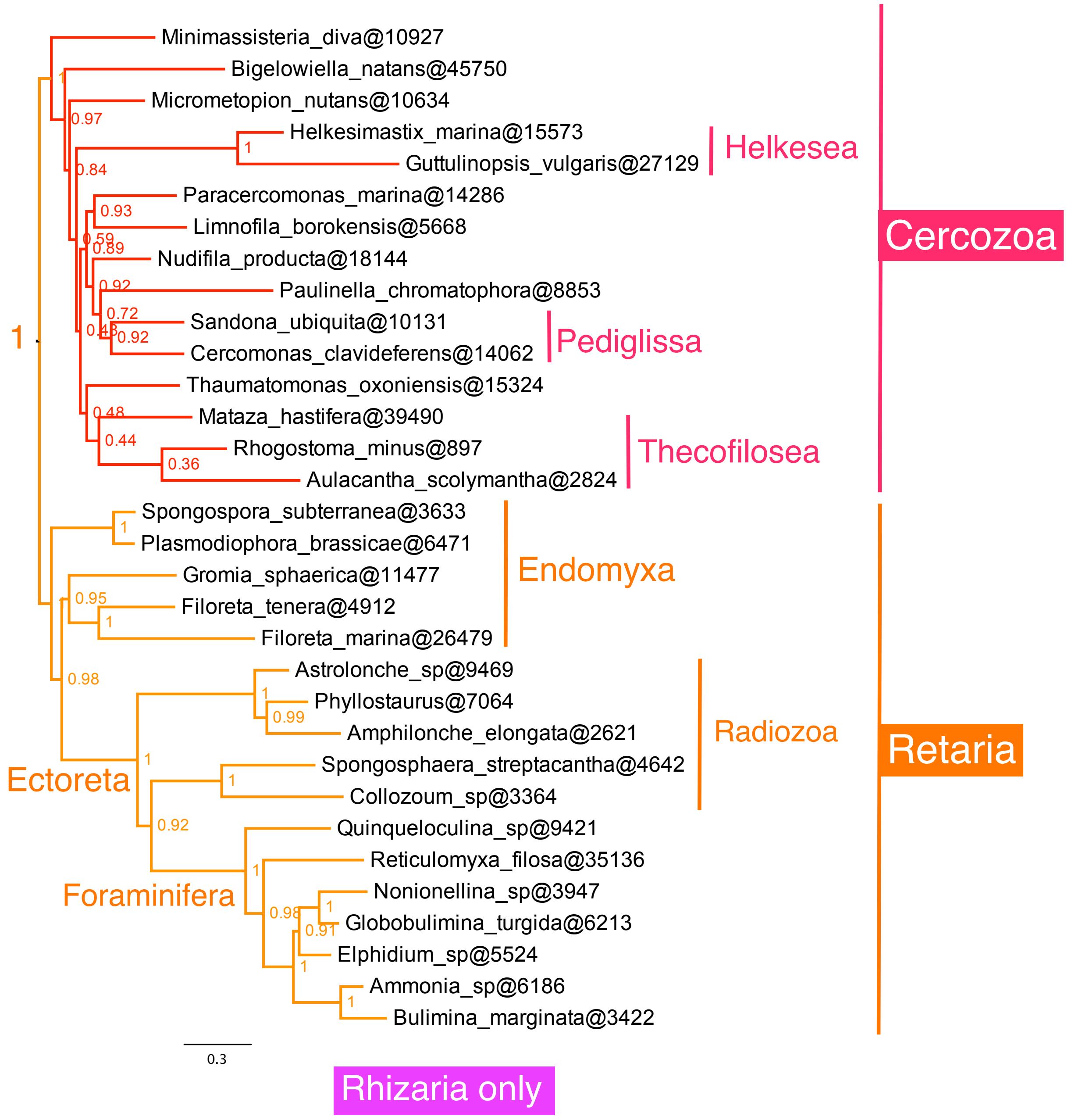

Supplementary Fig. S6.

<u>Supplementary Fig. S7.</u> PhyloBayes CAT GAMMA 4 rate PhyloBayes phylogeny for 187 proteins and 30 Rhizaria, omitting *Limnofila*. Consensus tree for two chains with identical topology (maxdiff 0.115257; burnin 741; 14,887 trees summed). Note improved support for basal cercozoan branching.

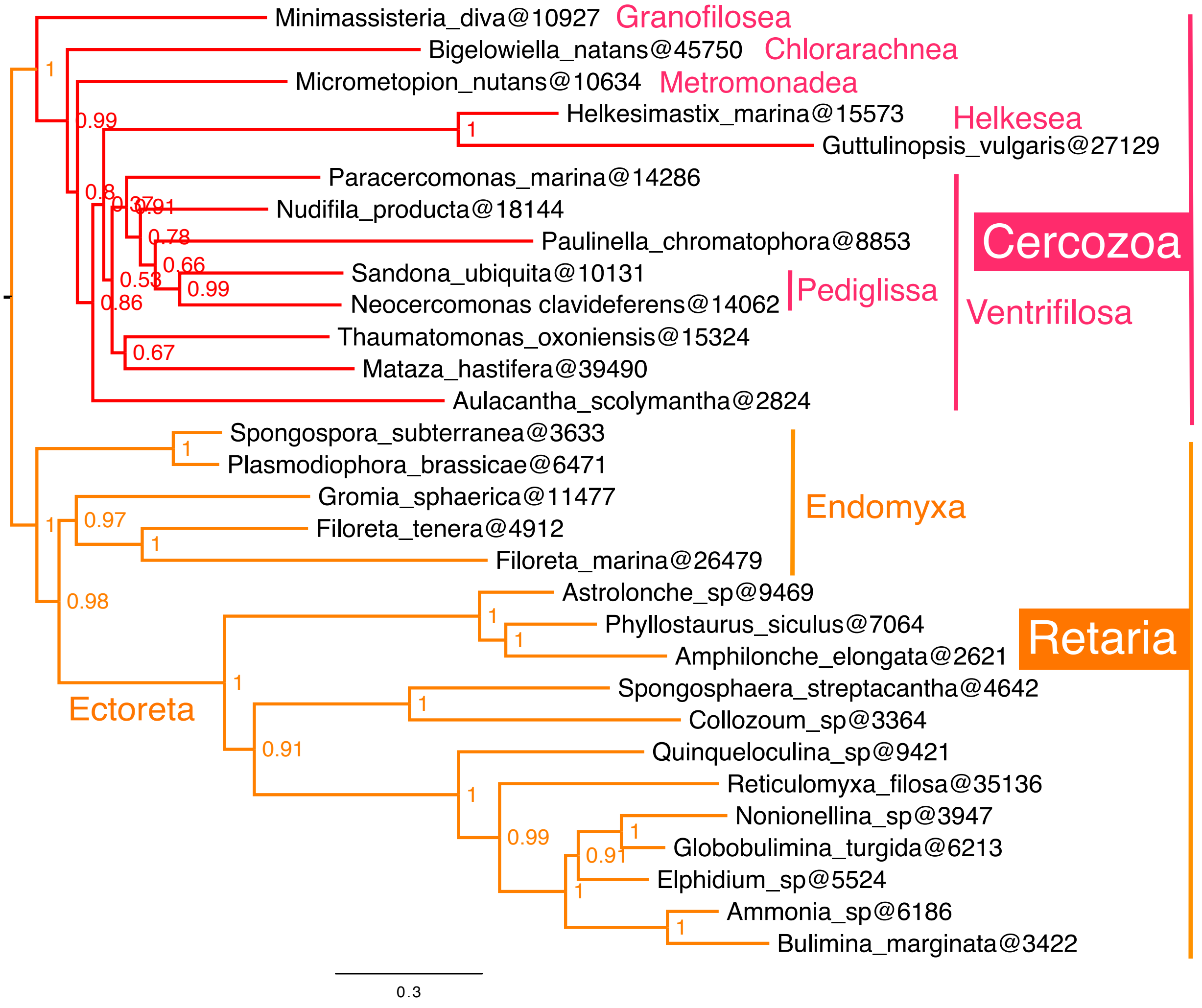

<u>Supplementary Fig. S8.</u> 18S rDNA PhyloBayes CAT-GTR-Γ (4 rate classes) phylogeny for 467 Rhizaria using 1788 positions (3 more than in a similar 273-taxon tree Monadofilosa-only tree: Scoble and Cavalier-Smith 2014). Two chains were run which converged well to the same topology (Maxdiff 0.190343, meandiff 0.00225341); 38,865 trees were summed after removing the first 3000 as burn in. This is the most comprehensive rhizarian rDNA tree to date and for Monadofilosa is largely similar to that of Scoble and Cavalier-Smith (2014), which was not rooted correctly because of the absence of Reticulofilosa and Retaria. For the first time it includes Helkesida and Ventricleftida in the same tree; helkesids were excluded by Cavalier-Smith and Scoble (2014) because of their exceptionally long branch. Support values for some major groups are shown in larger type; for these the posterior probability for the CAT trees are on the left and for the corresponding RAxML GTR-Γ tree on the right (Fig. S9 shows the complete ML tree).

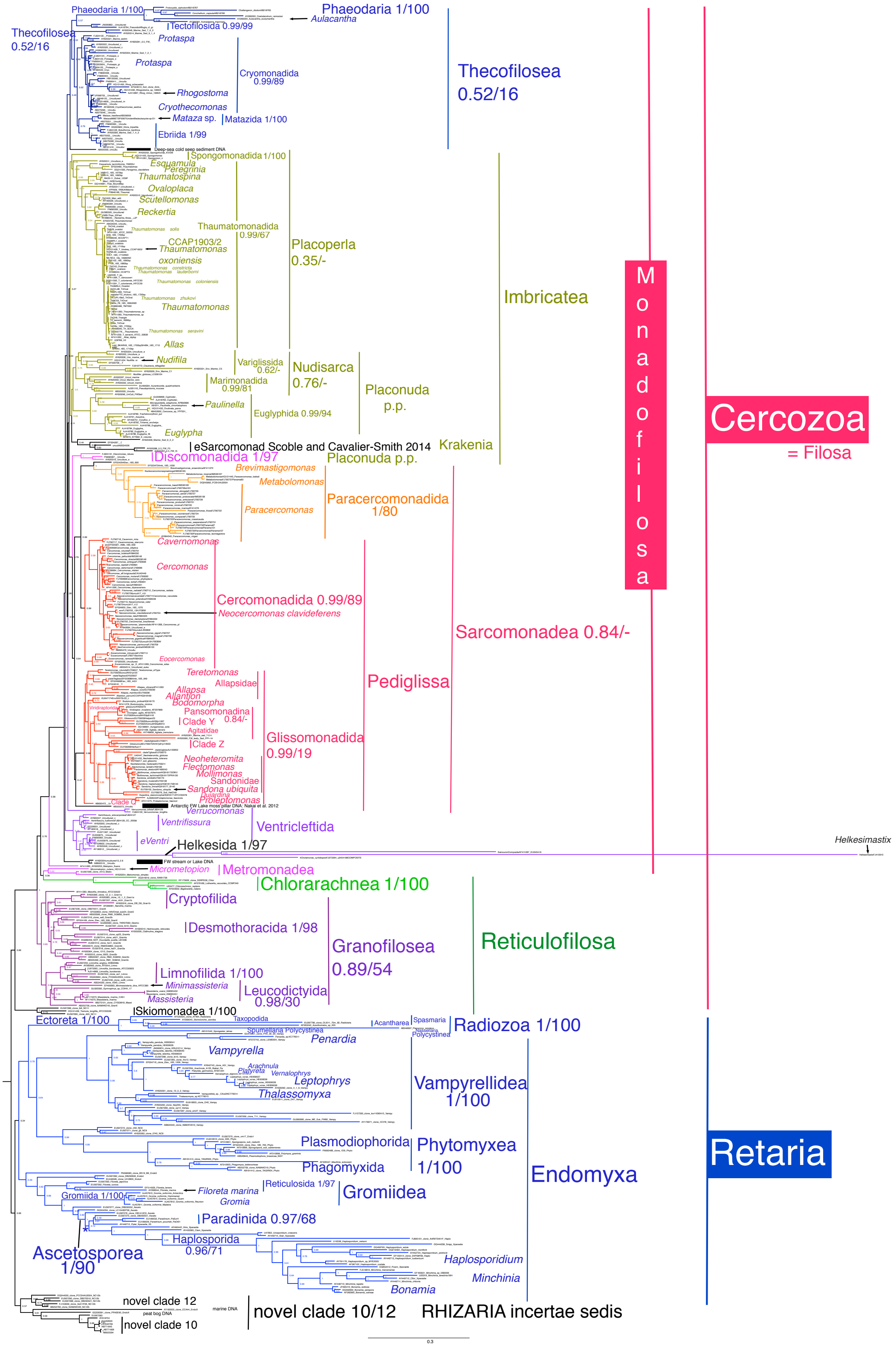

Fig. S8

Note: To root Fig. S8 a separate 481-taxon tree was run using the same rhizarian taxa plus 14 non-rhizarian chromists as outgroup, short branch taxa only being included: 10 Halvaria (5 heterokonts, 5 alveolates), four Hacrobia (3 haptophytes, *Telonema*). Despite summing nearly as many trees (35,182 after removing 1608 trees as burnin) this 481-taxon tree (not shown) converged less well (maxdiff 1, meandiff 0.0111421) and tree topology of the chains differed slightly, though in nearly

all respects they were the same as Fig. S8. One key difference was that in the 481-taxon consensus tree Radiozoa did not branch within Endomyxa but were sister to Endomyxa plus Filosa: Endomyxa was a clade with 0.24 support (its topology differed by having environmental DNA sequence FN598385 as deepest diverging rather than as sister to the Gromiidea/Ascetosporea subclade). This contradiction confirms that rDNA cannot clearly establish the branching order of these three major rhizarian groups. Novel clades 10 and 12 formed a joint clade on the 481-taxon tree with weak support (0.52) that was weakly sister to Endomyxa with insignifacnt support (0.18). Previously using only 148 sequences, 1539 positions Howe et al. (2011) put this joint clade as weakly sister to Filosa (0.59 Mr Bayes, 40% RaxML), but that tree included no non-Rhizaria so its root was simply assumed to be between Radiozoa and Endomyxa, which our multiprotein trees show is probably incorrect – it therefore had no objective way of establishing the rhizarian root and therefore whether clade 10/12 is sister to Filosa (as the rooting of Howe et al. (2011) Fig. 1 assumes) or to Retaria (as our CAT Figs S8 and S10, S11 all strongly show) or to Endomyxa (as in the 416-taxon consensus tree). As support for the position of clade 10/12 is so weak, any of these three could be correct. The most important unresolved questions for the deep phylogeny and early evolution of Rhizaria is what is the phenotype of the putative clade 10/12 and which of these three topologies is correct, for which multigene trees are essential.

Supplementary Fig. S9. 18S rDNA RAxML phylogeny for 467 Rhizaria

This figure (**next page**) shows the corresponding ML tree for the Fig. S8 alignment. Key differences are that Spongomonadida do not group with the ultrastructurally related Thecomonadida, Nudisarca are not a clade, Helkesea are a weakly supported clade, Metromonadea are broken into three, not two clades (and Chlorarachnea do not group with any of them but are lower than two). Despite these insignificantly supported contradictions, topological agreement between ML and CAT is generally good even though the basal branching order of both is very weakly supported compared with our protein trees.

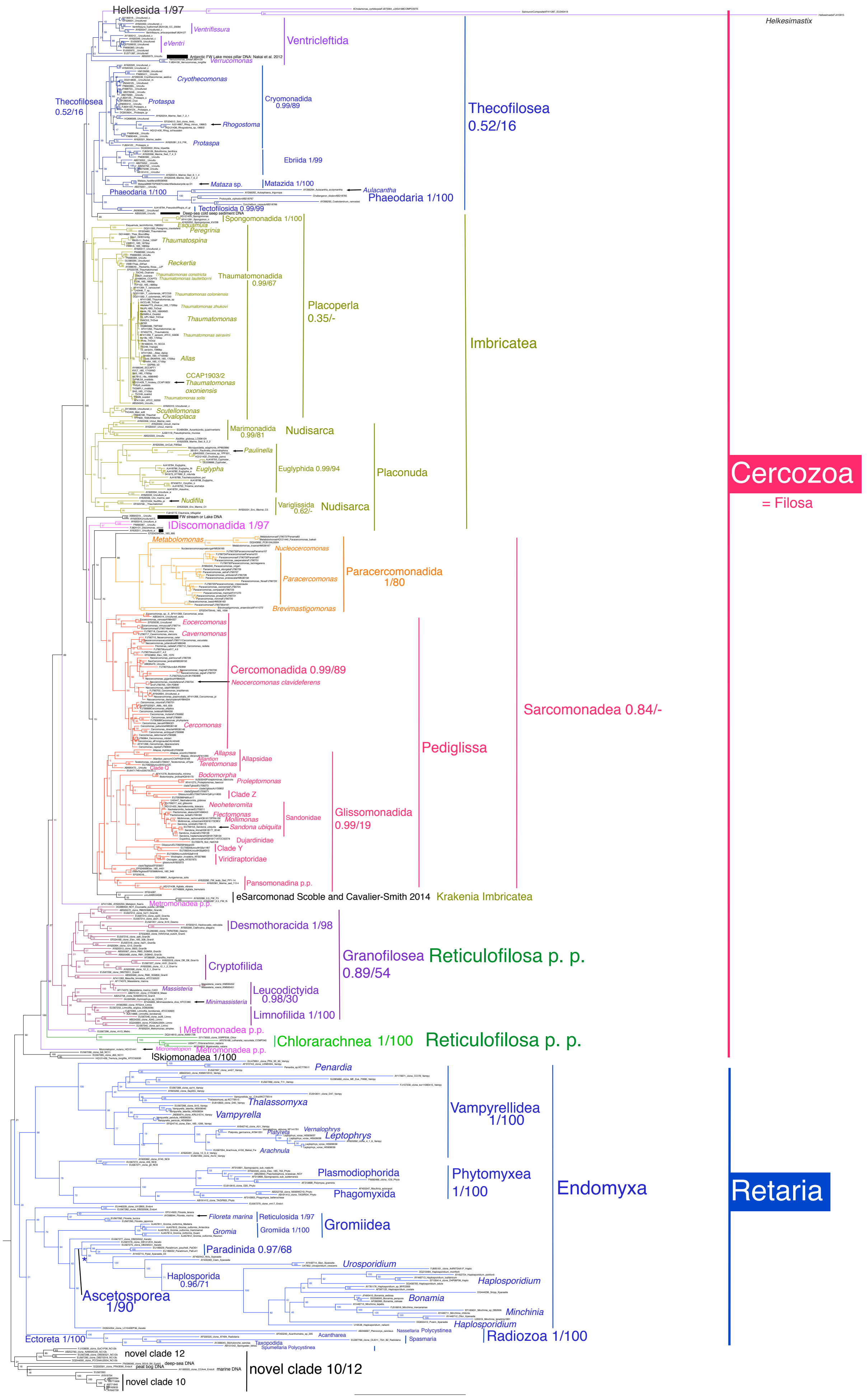
Helkesida 1/97
Helkesimastix
Ventrifissura
eVentri
Ventricleftida
Verrucomonas
Antarctic FW Lake moss pillar DNA: Nakai et al. 2012
Thecofilosea
0.52/16
Cryothecomonas
Protaspa
Cryomonadida
0.99/89
Rhogostoma
Protaspa
Ebriida 1/99
Mataza sp.
Matazida 1/100
Aulacantha
Phaeodaria 1/100
Phaeodaria 1/100
Tectofilosida 0.99/99
Deep-sea cold seep sediment DNA
Thecofilosea
0.52/16
Spongomonadida 1/100
Esquamula
Peregrinia
Thaumatospina
Reckertia
Thaumatomonadida
0.99/67
Thaumatomonas
Placoperla
0.35/-
Imbricatea
Allas
CCAP1903/2
Thaumatomonas
oxoniensis
Scutellomonas
Ovaloplaca
Marimonadida
0.99/81
Nudisarca
Paulinella
Euglyphida 0.99/94
Euglypha
Placonuda
Nudifila
Variglissida
0.62/-
Nudisarca
FW stream or Lake DNA
Discomonadida 1/97
Metabolomonas
Nucleocercomonas
Paracercomonadida
1/80
Paracercomonas
Brevimastigomonas
Eocercomonas
Cavernomonas
Cercomonadida 0.99/89
Neocercomonas clavideferens
Cercomonas
Sarcomonadea 0.84/-
Allapsa
Allantion
Teretomonas
Allapsidae
Clade Q
Bodomorpha
Proleptomonas
Clade Z
Neoheterormita
Flectomonas
Mollimonas
Sandona ubiquita
Sandonidae
Glissomonadida
0.99/19
Pediglissa
Dujardinidae
Clade Y
Viridiraptoridae
Pansomonadina p.p.
eSarcomonad Scoble and Cavalier-Smith 2014
Krakenia Imbricatea
Metromonadea p.p.
Desmothoracida 1/98
Granofilosea
0.89/54
Reticulofilosa p. p.
Cryptofilida
Massisteria
Leucodictyida
0.98/30
Minimassisteria
Limnofilida 1/100
Metromonadea p.p.
Chlorarachnea 1/100
Reticulofilosa p. p.
Micrometopion
Metromonadea p.p.
Skiomonadea 1/100
Cercozoa
= Filosa
Penardia
Thalassomyxa
Vampyrella
Vampyrellidea
1/100
Platyreta
Vernalophrys
Leptophrys
Arachnula
Plasmodiophorida
Phytomyxea
1/100
Phagomyxida
Endomyxa
Retaria
Filoreta marina
Reticulosida 1/97
Gromia
Gromiida 1/100
Gromiidea
Paradinida 0.97/68
Urosporidium
Haplosporida
0.96/71
Haplosporidium
Ascetosporea
1/90
Bonamia
Minchinia
Haplosporidium
Ectoreta 1/100
Acantharea
Nassellaria Polycystinea
Taxopodida
Spumellaria Polycystinea
Spasmaria
Radiozoa 1/100
novel clade 12
deep-sea DNA
peat bog DNA
marine DNA
novel clade 10/12
novel clade 10

S9

<u>Supplementary Fig. S10.</u> 18S rDNA PhyloBayes CAT phylogeny for 465 Rhizaria (excluding two longest branch Helkesida, *Helkesimastix* and *Sainouron*). Support values are posterior probabilities for the consensus tree from two chains; a few bootstrap support values are added on the right for major clades for the corresponding RAxML tree (not shown):

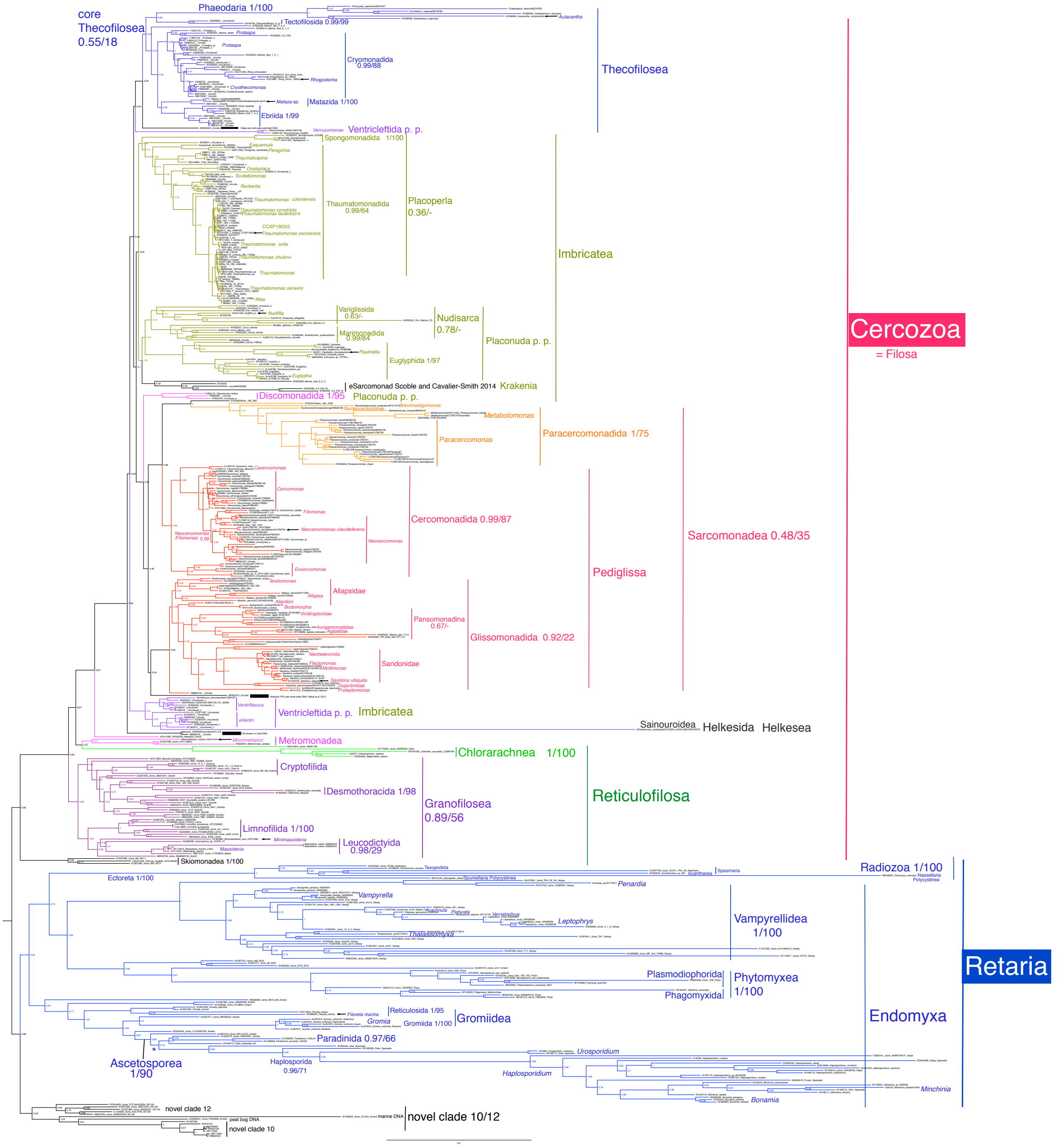

<u>Supplementary Fig. S11.</u> 18S rDNA PhyloBayes CAT phylogeny for 464 Rhizaria (excluding all three long-branch Helkesida (*Helkesimastix*, *Sainouron*, *Cholamonas*) (next page). Support values are posterior probably for the consensus tree from two chains; a few bootstrap support values are added on the right for major clades for the corresponding RAxML tree (not shown). Comparison with Fig. S8 suggests that adding the long-branch Sainouroidea artefactually pushed *Verrucomonas* one node higher but was otherwise harmless to topology in these very taxon- and position-rich site-

heterogeneous PhyloBayes trees.

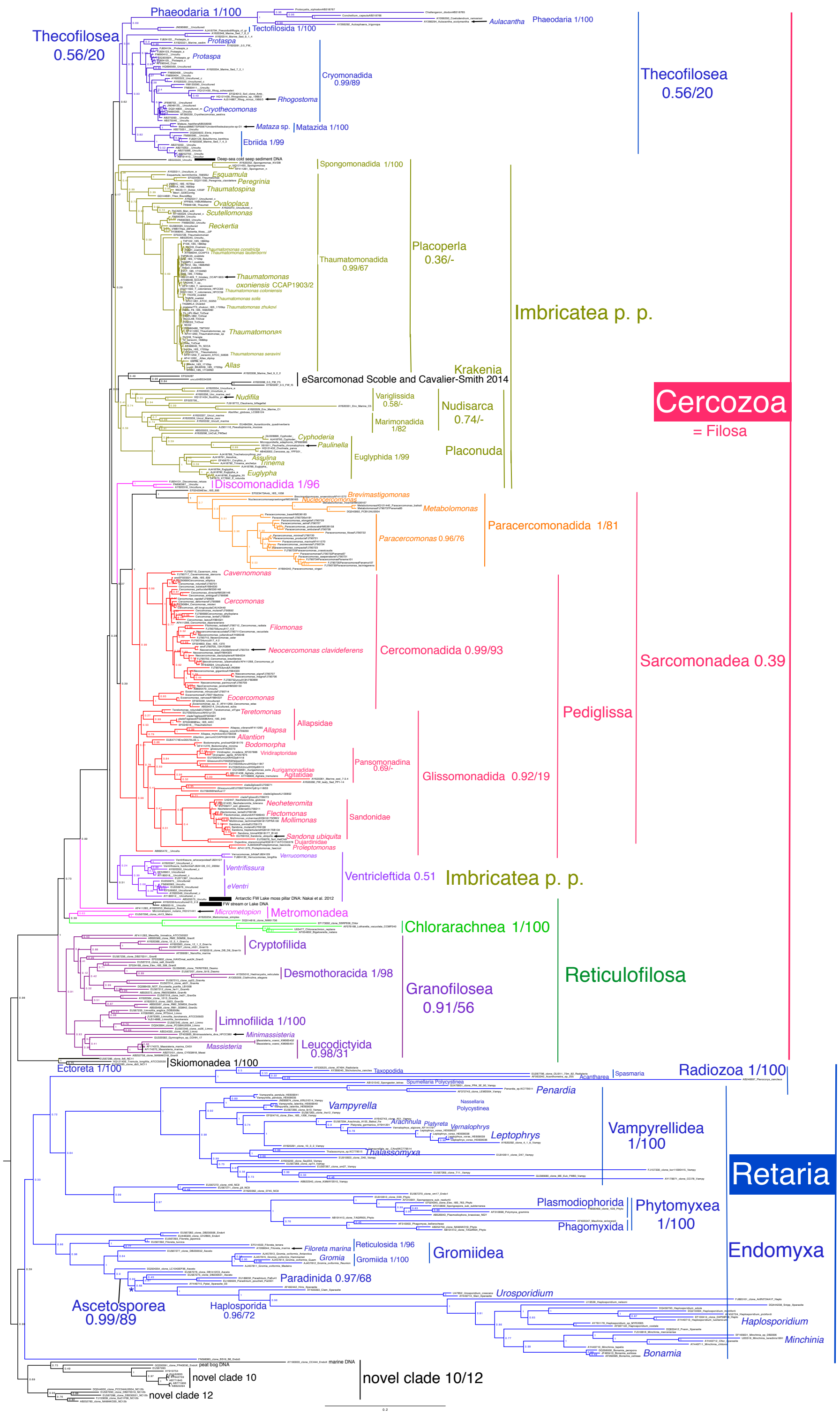

__Supplementary Fig. S12.__ **18S rDNA PhyloBayes CAT-GTR-Γ (4 rate classes) phylogeny** for 316 Monadofilosa plus EF024169, an especially short branch representative of Granofilosea (the closest outgroup) to root the tree, using 1790 positions (5 more than the 1785 in the previously most taxon-rich 273-taxon Monadofilosa-only tree: Scoble and Cavalier-Smith 2014). The two chains converged well (Maxdiff 0.143037); 153,615 trees were summed after removing the first 28,560 as burn in. Support values are posterior probability; for key clades only, bootstrap percentages for the corresponding ML tree (Fig. S14) are added on the right. Fig. S13 is a CAT tree for the same taxa minus *Cholamonas* excluded because of its much longer branch.

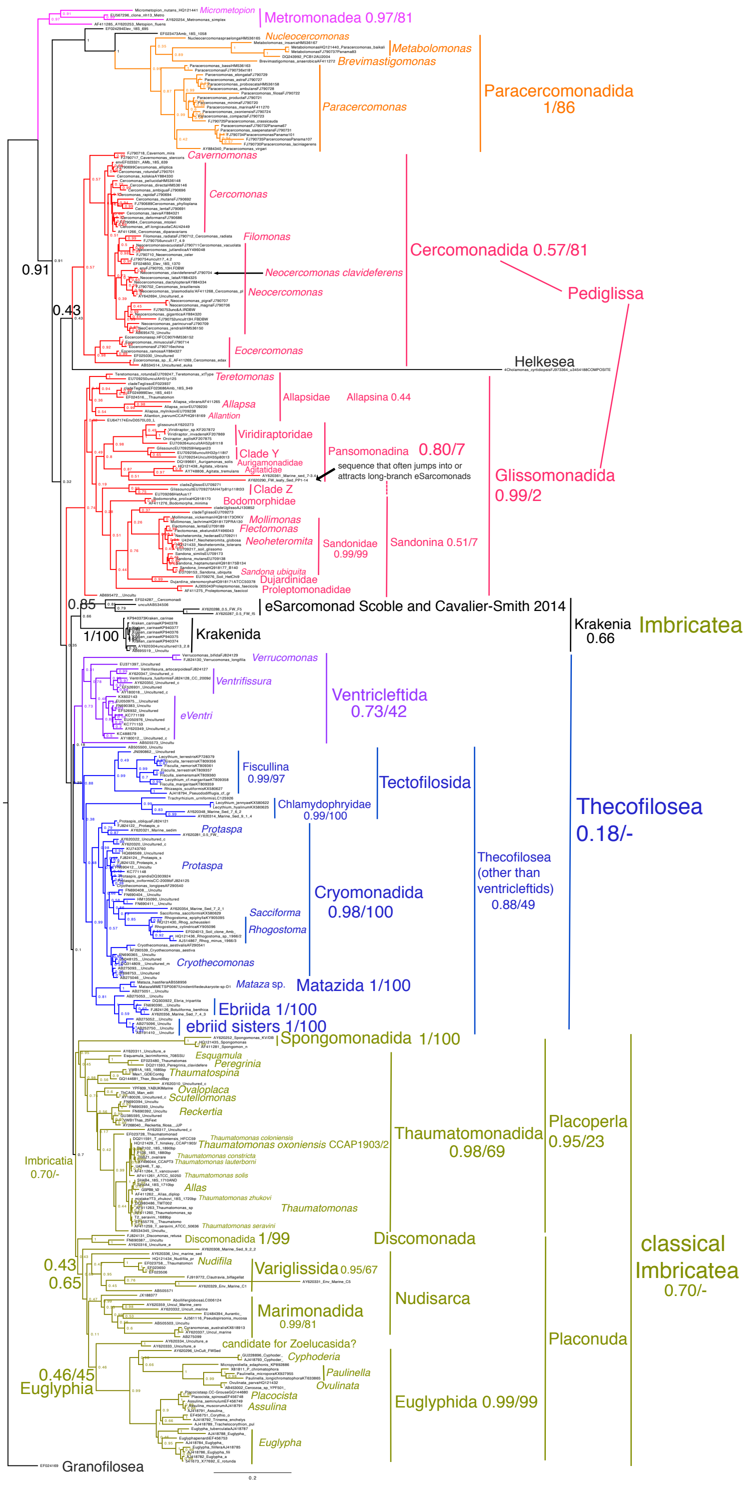

Supplementary Fig. S12. **18S rDNA PhyloBayes CAT-GTR-Γ (4 rate classes) phylogeny**

__Supplementary Fig. S13.__ **18S rDNA PhyloBayes CAT phylogeny for 315 monadofilosan Cercozoa excluding helkesid *Cholamonas* (otherwise same taxa as Fig. S12), rooted using a granofilosean as outgroup (next page).** Consensus tree for two chains (maxdiff. 0.0760963; 152,964 trees summed after removing burnin); support values posterior probabilities (plus bootstrap percentages on the right for a few key clades from the corresponding RAxML tree: Fig. S15). This is the only one of the four Monadofilosa-only trees where Cercomonadidida are sisters of Paracercomonadida. In none of them are paracercomonads sister to Krakenida or Krakenia. Imbricatea are a clade by CAT but not by ML. Compared with Fig. S12 where *Cholamonas* was present, the two long-branch eSarcomonad sequences have jumped into pansomonads to join sequence AY620690, which in the Fig. S15 ML tree jumped out of pansomonads/glissomonads into eSarcomonads, lowering support values for both. That jumping of AY620690 into eSarcomonads was also seen on the ML tree without *Cholamonas* (Fig. S15) so is a systematic difference between ML and CAT.

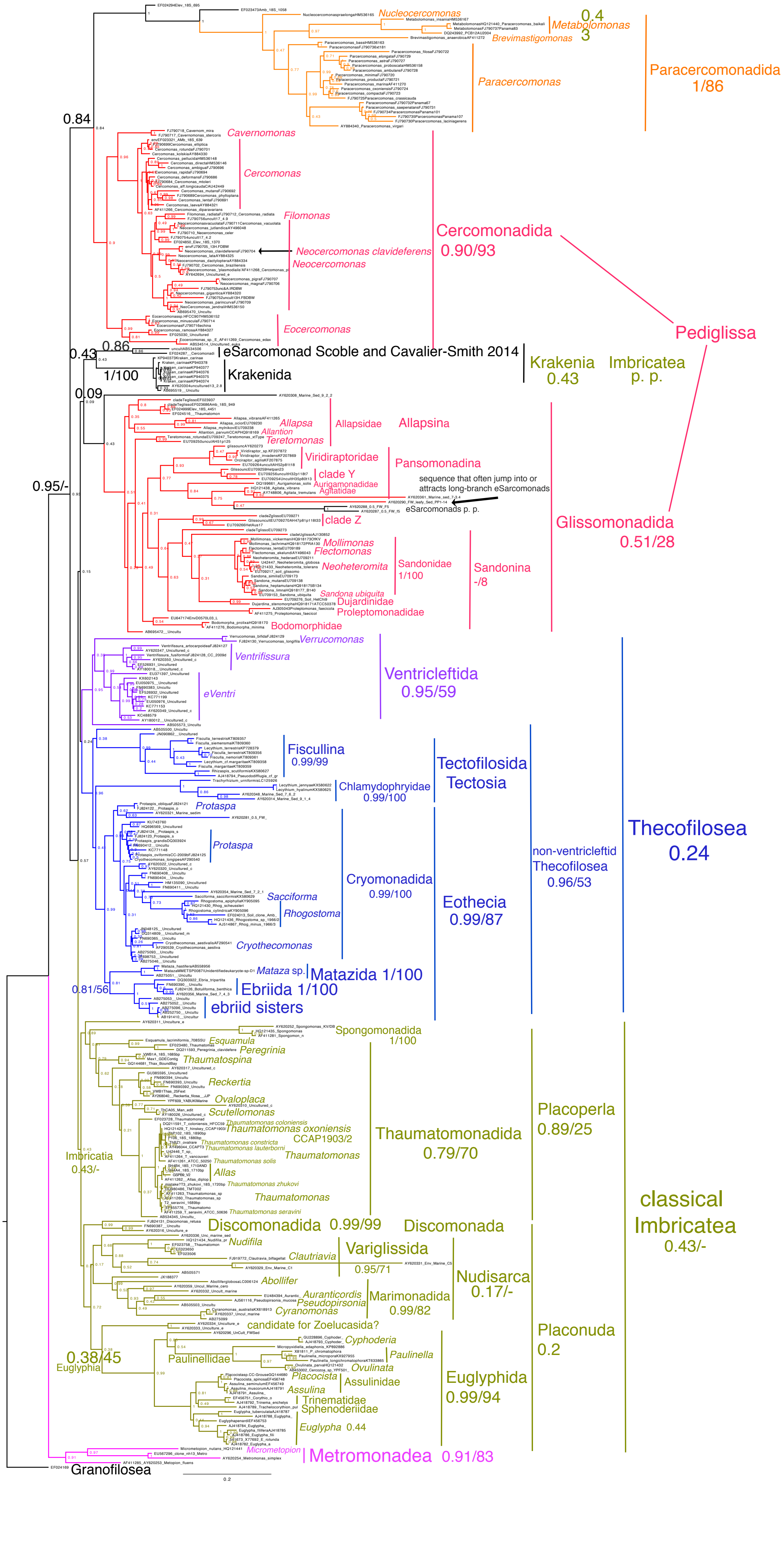
Nucleocercomonas
Metabolomonas
0.4
3
Brevimastigomonas
Paracercomonas
Paracercomonadida
1/86
0.84
Cavernomonas
Cercomonas
Filomonas
Neocercomonas clavideferens
Neocercomonas
Cercomonadida
0.90/93
Eocercomonas
Pediglissa
0.86
0.43
eSarcomonad Scoble and Cavalier-Smith 2014
Krakenia
0.43
Imbricatea
p. p.
1/100
Krakenida
0.09
Allapsa
Allantion
Teretomonas
Allapsidae
Allapsina
Viridiraptoridae
Pansomonadina
clade Y
Aurigamonadidae
Agitatidae
sequence that often jump into or
attracts long-branch eSarcomonads
eSarcomonads p. p.
clade Z
Glissomonadida
0.51/28
0.95/-
Mollimonas
Flectomonas
Neoheteromita
Sandonidae
1/100
Sandonina
-/8
Sandona ubiquita
Dujardinidae
Proleptomonadidae
Bodomorphidae
Verrucomonas
Ventrifissura
Ventricleftida
0.95/59
eVentri
Fiscullina
0.99/99
Tectofilosida
Tectosia
Chlamydophryidae
0.99/100
Protaspa
Thecofilosea
0.24
non-ventricleftid
Thecofilosea
0.96/53
Cryomonadida
0.99/100
Sacciforma
Rhogostoma
Eothecia
0.99/87
Cryothecomonas
Mataza sp.
Matazida 1/100
0.81/56
Ebriida 1/100
ebriid sisters
Spongomonadida
1/100
Esquamula
Peregrinia
Thaumatospina
Reckertia
Ovaloplaca
Scutellomonas
Thaumatomonas coloniensis
Thaumatomonas oxoniensis
CCAP1903/2
Thaumatomonas constricta
Thaumatomonas lauterborni
Thaumatomonas
Thaumatomonadida
0.79/70
Placoperla
0.89/25
Imbricatia
0.43/-
Thaumatomonas solis
Allas
Thaumatomonas zhukovi
Thaumatomonas
Thaumatomonas seravini
classical
Imbricatea
0.43/-
Discomonadida
0.99/99
Discomonada
Nudifila
Clautriavia
Variglissida
0.95/71
Nudisarca
0.17/-
Abollifer
Auranticordis
Pseudopirsonia
Cyranomonas
Marimonadida
0.99/82
candidate for Zoelucasida?
Placonuda
0.2
Cyphoderia
0.38/45
Euglyphia
Paulinellidae
Paulinella
Placocista
Ovulinata
Assulinidae
Assulina
Trinematidae
Sphenoderiidae
Euglyphida
0.99/94
Euglypha 0.44
Micrometopion
Metromonadea 0.91/83
Granofilosea
0.2

<u>**Supplementary Fig. S14.**</u> **18S rDNA RAxML phylogeny for 316 monadofilosan Cercozoa including helkesid *Cholamonas* (next page).** Unlike the corresponding CAT tree (Fig. S12) Imbricatea appear as paraphyletic, not a clade, and Metromonadea are not the deepest clade, both being contradicted by the multiprotein trees, consistent with the general greater accuracy of the evolutionarily more realistic CAT model. Rooted by a short-branch granofilosean as outgroup.

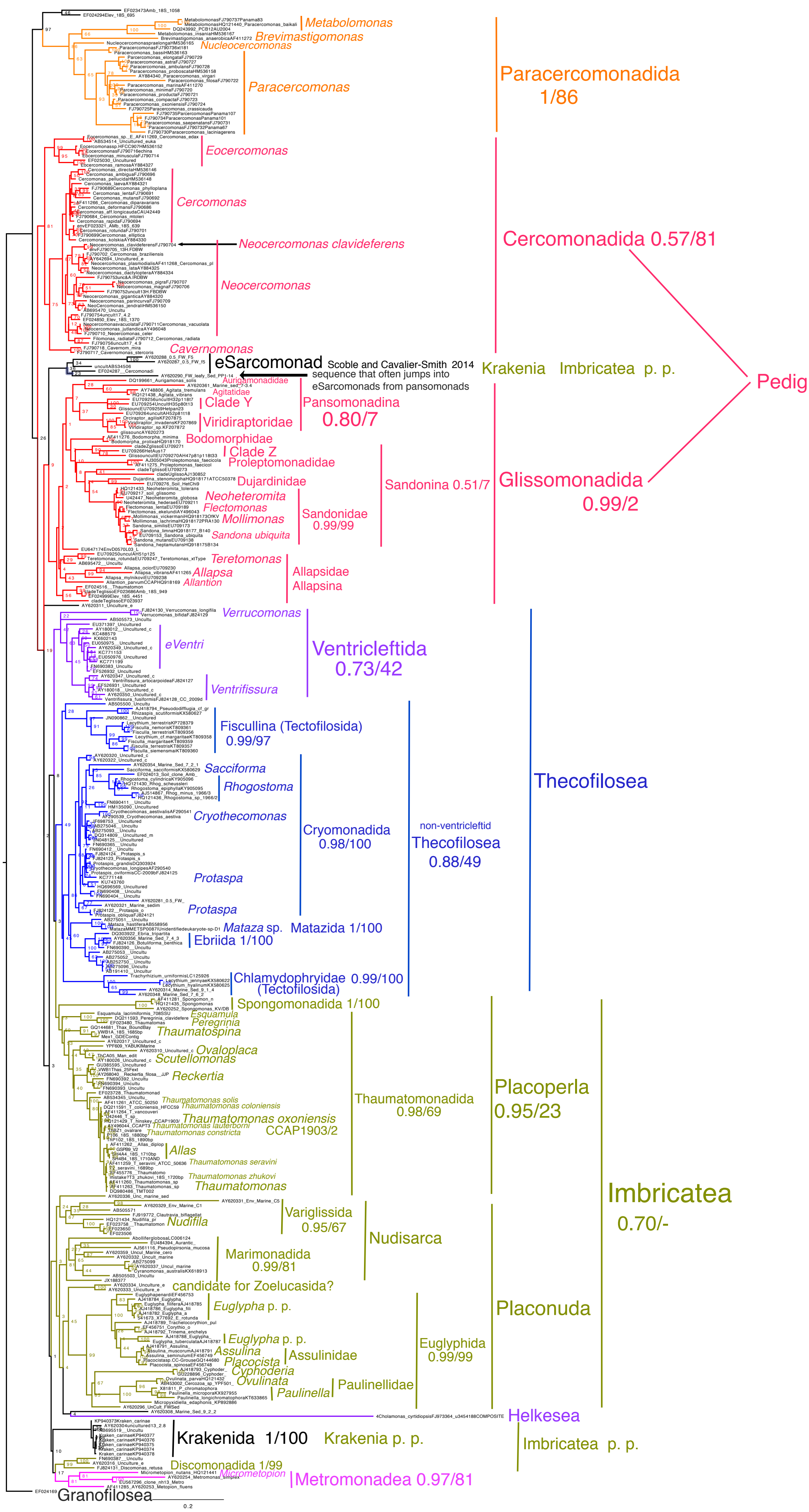
Metabolomonas
Brevimastigomonas
Nucleocercomonas
Paracercomonas
Paracercomonadida
1/86
Eocercomonas
Cercomonas
Neocercomonas clavideferens
Neocercomonas
Cercomonadida 0.57/81
Cavernomonas
eSarcomonad
Scoble and Cavalier-Smith 2014
sequence that often jumps into
eSarcomonads from pansomonads
Krakenia
Imbricatea p. p.
Pediglissa
Aurigamonadidae
Agitatidae
Clade Y
Viridiraptoridae
Pansomonadina
0.80/7
Bodomorphidae
Clade Z
Proleptomonadidae
Dujardinidae
Neoheteromita
Flectomonas
Mollimonas
Sandonidae
0.99/99
Sandona ubiquita
Sandonina 0.51/7
Glissomonadida
0.99/2
Teretomonas
Allapsa
Allantion
Allapsidae
Allapsina
Verrucomonas
eVentri
Ventricleftida
0.73/42
Ventrifissura
Fiscullina (Tectofilosida)
0.99/97
Sacciforma
Rhogostoma
Cryothecomonas
Cryomonadida
0.98/100
non-ventricleftid
Thecofilosea
0.88/49
Thecofilosea
Protaspa
Protaspa
Mataza sp.
Matazida 1/100
Ebriida 1/100
Chlamydophryidae 0.99/100
(Tectofilosida)
Spongomonadida 1/100
Esquamula
Peregrinia
Thaumatospina
Ovaloplaca
Scutellomonas
Reckertia
Thaumatomonas solis
Thaumatomonas coloniensis
Thaumatomonas oxoniensis
Thaumatomonas lauterborni
Thaumatomonas constricta
CCAP1903/2
Thaumatomonadida
0.98/69
Placoperla
0.95/23
Allas
Thaumatomonas seravini
Thaumatomonas zhukovi
Thaumatomonas
Variglissida
0.95/67
Nudifila
Nudisarca
Imbricatea
0.70/-
Marimonadida
0.99/81
candidate for Zoelucasida?
Euglypha p. p.
Euglypha p. p.
Assulina
Placocista
Assulinidae
Euglyphida
0.99/99
Placonuda
Cyphoderia
Ovulinata
Paulinella
Paulinellidae
Helkesea
Krakenida 1/100
Krakenia p. p.
Imbricatea p. p.
Discomonadida 1/99
Micrometopion
Metromonadea 0.97/81
Granofilosea
0.2

Figure S14

<u>**Supplementary Fig. S15.**</u> **18S rDNA RAxML phylogeny for 315 monadofilosan Cercozoa excluding helkesid *Cholamonas* (next page).** Rooted by a short branch granofilosean as outgroup. In contrast to the corresponding CAT tree (Fig. S13) classical Imbricatea appear paraphyletic because of the likely artefactual intrusion of Thecofilosea, Krakenida and Metromonadea. For key clades PP support values from Fig. S13 are inserted to the left of the magnified ML bootstrap support percentages. On neither ML tree do paracercomonads group with cercomonads or krakenids.

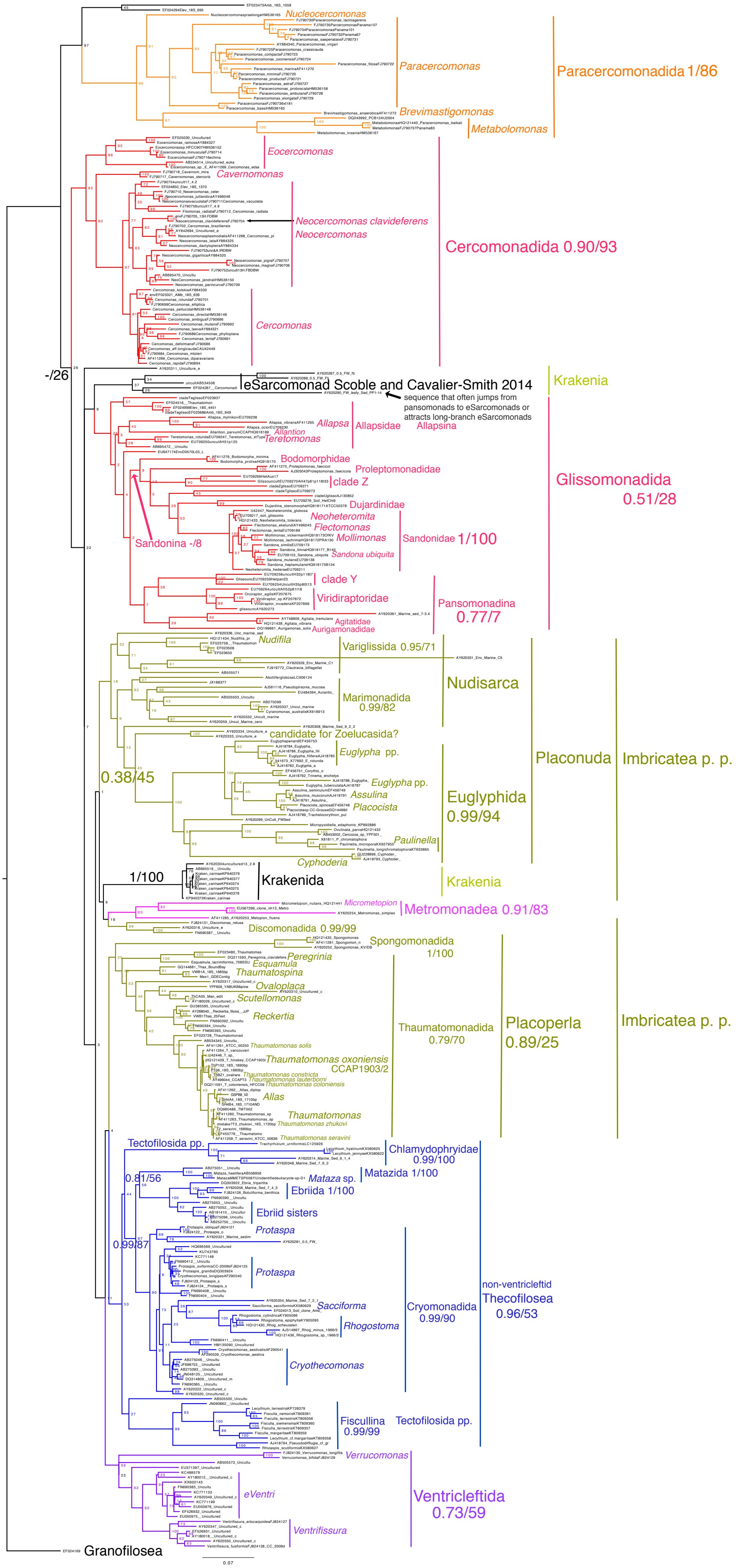

Figure S15. ML 316 taxa

Supplementary discussion of the rDNA trees

(a) New Rhizaria-wide rDNA trees (Figs S8-S11)

Fig. S8 and the corresponding ML tree (Fig. S9) both place Helkesida within the environmental DNA branch eVentri found by Scoble and Cavalier-Smith (2014) to be sister to the ventricleftid flagellate *Ventrifissura*. In Fig. S8 *Ventrifissura* is sister to this eVentri/Helkesida clade. Thus by CAT 18S rDNA Helkesida including *Helkesimastix* are in exactly the same position as sister to Sarcomonadea plus Thecofilosea as shown by our multiprotein trees. Though that weakly raises the psiibility that Ventricleftida might be paraphyletic ancestors of Helkesida, that appears to be ruled out by our Monadofilosa-only trees (Figs S12-S15) which appear to be more accurate and less affected by long-branch problems (see discussion below in **(b)**). In Fig. S8 *Verrucomonas* does not group with *Ventrifissura* but one node higher, insignificantly sister to Sarcomonadea alone. When all Helkesida are excluded (Fig. S11) CAT shows Ventricleftida as a clade with *Verrucomonas* within eVentri. Fig. S6 also shows that strain MMETSP0087 is a *Mataza* but sufficiently distinct to be a second undescribed species. The position of Matazida within Thecofilosea (sister to Ebriida) agrees with Scoble and Cavalier-Smith (2014), not Yabuki and Ishida (2011).

In these figures classical Imbricatea are labelled olive green to emphasise that on 18S rDNA trees Imbricatea in the classical sense (excluding Krakenia, unlike Table 1) can appear as weakly supported clade (here PP 0.27) or as paraphyletic (Fig. S9 where Spongomonadida group weakly with Thecofilosea not Thaumatomonadida). This conflict highlights the need for better taxon sampling on multigene trees. On this better sampled tree the deep-branching environmental DNA clade eSarcomonad is weakly sister to the Nudisarca/Euglyphida clade (i.e. Placonuda in the original sense: Cavalier-Smith and Chao 2012; Scoble and Cavalier-Smith 2014) and not to Glissomonadida (including Pansomonadina) as in Scoble and Cavalier-Smith (2014). However by ML (Fig. S9) it is still sister to Glissomonadida. It is the major sarcomonad clade with no known phenotype, but our CAT Monadofilosa-only trees suggest that it may be related to *Kraken* (Figs S12, S13), three other more sparsely represented deep branching ventrifilosan environmental DNA lineages (rectangles) were not previously assigned to orders. AB505573 from a moss pillar in an Antarctic lake that is weakly sister to Glissomonadida (Figs S8, S10) or within Ventricleftida (Figs S9, S11); in Monadofilosa-only trees it is sister to *Verrucomonas* within Ventricleftida (Fig. S12) or sister to Ventrocleftida and thus likely a ventricleftid. A fresh water clade from a stream or lake (AY620304, AB695519) is deeper branching, sister to Ventrifilosea plus Helkesea by CAT (Fig. S8, S10, S11 and in the 481-taxon tree) so likely of more novel phenotype, but is consistently extremely weakly sister to cercomonads, glissomonads and *Discomonas* by ML (e.g. Fig. S9). However, AY695519 was recently found to be a *Kraken* sequence (Dumack et al. 2016); our

Monadofilosa-only trees confirm that and show that AY620304 is also a *Kraken* (Fig. S12). Finally, AB505500 from a deep-sea cold seep is consistently weakly sister to Thecofilosea by CAT (Figs S8, S10, S11) or to Spongomonadida by ML (Fig. S9); the Monadofilosa trees place it with or within Thecofilosea, likely its correct position. Thus none of the deepest branching cercozoan environmental sequences are likely to represent novel classes, though some might be new orders.

The only major contradiction between the Figs S8-11 18S rDNA trees that include at least one helkesid and our protein trees is that with 18S rDNA all cercomonads (Cercomonadida, Paracercomonadida) form a single near maximally supported clade so Pediglissa is not a clade. However the Monadofilosan-only trees (Figs S12-15) mostly do not group both cercomonad orders together and thus are more consistent with the protein trees.

One feature of all these rather comprehensive Cercozoa-wide rDNA trees is that Discomonadida are consistently sisters to cercomonads plus glissomonads by both methods, not with classical imbricates as previously (Howe et al. 2011; Scoble and Cavalier-Smith 2014) or with much lower taxon and positional sampling in other contradictory positions (Chantangsi and Leander 2010). However, when distant outgroups are excluded CAT trees put *Discomonas* weakly within classical imbricates as sister to euglyphids plus Nudisarca (Fig S12, S13), so Table 1 leaves it in Imbricatia.

Ascetosporea invariably branch strongly within Gromiidea as sisters of *Gromia*. Phytomyxea and Vampyrellidea are invariably well-supported sisters. This unambiguously confirms that Endomyxa comprise two robust clades (Bass et al. 2009), now made superclasses Marimyxia and Proteomyxia (Table 1).

(b) New Monadofilosa-only trees (Figs S12-S15)

Comparison of Fig. 12 with Fig. S11 that excludes all three Helkesida and Fig. S10 that excludes only the two longest branches (*Helkesimastix* and *Sainouron*) shows that the rest of the tree is almost identical whether or not these rapidly evolving taxa are present, so their inclusion is not significantly distorting for PhyloBayes CAT when this many taxa and nucleotide positions are included.

In marked contrast to Figs S8-11 Rhizaria-wide trees and many published trees that included the long-branch Rhizaria and Chlorarachnea as outgroups, Metromonadea is a robustly supported clade (0.97) in Fig. S12 and in all the other 316/7 taxon Monadofilosa trees (both CAT and ML: Figs S13-15; 81%, 0.91, 83%) rooted only on one short branch granofilosean. This strongly suggests that apparent paraphyly of Metromonadea on many other trees is an artefact of unequal branch lengths and Metromonadea are a clade as suggested by their shared ultrastructure. Metromonadea are the deepest monadofilosan branch by CAT (0.93) but not by ML where they insignificantly group with

discomonads (Figs S13, 15). Ventricleftida also are consistently a clade by both methods (Figs S13-15), and is sister to other Thecofilosea with which it is classified; the shortest-branch helkesid *Cholamonas* never branches within ventricleftids or as their sister, suggesting that the insignificantly supported intrusion of helkesids into Ventricleftida in Figs S9 and 10 only was also artefactual.

Figs S12 and 13 are the first site-heterogeneous tree to include *Kraken* and representatives of all sequenced monadofilosan orders except for long-branch Phaeodaria (class Thecofilosea), which (unlike Figs S8-S11) were excluded to avoid their causing artefactual long-branch attraction. In marked contrast to published site-homogeneous 18S rDNA trees with many fewer taxa (Dumack et al. 2016 only 137 Rhizaria and 1680 nucleotide positions) there is no evidence that *Kraken* groups with Paracercomonadida. Instead Fig. S12 group it with moderate support (0.66, higher than previous support for a paracercomonad grouping - 0.6/27%) with environmental clade eSarco (Scoble and Cavalier-Smith 2014) comprising two short- and two long-branch sequences, none included by Dumack et al. (2016). The previous study (unlike the present one) did not recover Ventricleftida as a clade but agrees with ours in placing ventricleftids as sister to other Thecofilosea. The previous 2-gene rDNA tree was even more sparsely sampled (Dumack et al. 2017; 35 Cercozoa; 22 Monadofilosa with most orders on our tree not represented). The corresponding ML tree (Fig. S14) also gave no support for *Kraken* being related to paracercomonads but grouped it with insignificant support (10%) with the imbricate order Discomonadida and Metromonadea, whereas eSarco was in a deep unresolved position together with environmental DNA AY620290 as sister to Glissomonadida. As AY620290 consistently branches strongly within pansomonad glissomonads on our site-heterogeneous trees, we suggest its attraction to eSarco artefactually causes this clade to group with Glissomonadida rather than *Kraken* on ML trees. AY620290 has a third of the molecule missing and may be hard to place for that reason and might even be an unrecognised chimaera of a glissomonad and an eSarco sequence. Further evidence of such likely misleading attraction caused by conflicting signals in AY620290 is given by Fig. S13 that excluded the helkesid *Cholamonas*; in that tree *Kraken* still groups with the original short branch eSarco sequences (which are essentially full length) but with insignificant support (0.43), whereas the two long-branch eSarco sequences (both with one third missing) move into pansomonads as sister to AY620290 (insignificant 0.47 support). In the corresponding ML tree (Fig. S15) the positions of eSarco and *Kraken* are the same as when *Cholamonas* is included.

Fig. S12 agrees better with our multiprotein trees concerning the position of Paracercomonadida than did Figs S8-11 or most published trees. Unlike them paracercomonads do not group with Cercomonadida but are the second deepest branch in Monadofilosa after metromonads as they are also by ML whether *Cholamonas* is present (Fig. S14) or absent (Fig.

S15). Without *Cholamonas* paracercomonads plus two deep branching sequences likely to be also paracercomonads (a clade in ML, but not CAT) grouped with Cercomonadida with moderate (0.83) support by CAT only (Fig. S13). Even though the Fig. S12 CAT chains were run for a very long time they did not stably converge to precisely the same topology but differed in one respect: one was exactly as the Fig. S12 consensus; in the other the cercomonad/helkesid clade moved one node to be sister to paracercomonads. Part way through the Fig. S12 run maxdiff dropped to a lower value of 0.0997067 at which point the consensus also showed that second topology with the cercomonad/helkesid clade being insignificantly (0.32) sister to paracercomonads. This means that 18S rDNA has insufficient information to determine the relative positions of paracercomonads and cercomonads confidently. Therefore the fact that on some trees with some methods and taxon samples they group together is not a good reason for questioning the evidence from the multiprotein trees that they are not sisters, and does not contradict their classification here as two distinct orders.

The fact that in certain respects these monadofilosan-only trees agree more with the multiprotein trees than do the Rhizaria-wide trees indicates that inclusion of distant long-branch outgroups does distort some weakly supported branching orders. However the fact that the branching order within Thecofilosea on Figs S12-15 is essentially the same as in Figs S8-S11 that included the long-branch thecofilosean Phaeodaria suggests that this long-branch in-group does not significantly distort branching order of Thecofilosea. Classical Imbricatea are a weakly supported clade by CAT but not ML on Monadofilosa-only trees. As three out of four monadofilosan-only trees show Nudisarca as insignificantly paraphyletic, whereas Fig. S13 CAT and Rhizaria-wide ones showed them as a clade, multiprotein trees are esential to resolve this. We sugggest that the environmental DNA clade that is sister to Euglyphida may be the scale-bearing Zoelucasida. The weak support by ML for Placoperla is probably because environmental sequence AY620311 moved away to be near glissomonads. The weak ML support for glissomonads is because AY620290 moved away into eSarcomonad (see above). In these trees Pansomonadina remain within Glissomonadida; in Fig. S12 new glissomonad suborders Allapsina and Sandonina are clades with pansomonads and sandonines sisters. As unnamed strain Panama 103 from clade Z was a biciliate non-amoeboid glider with wobbling, nodding motion (Howe et al. 2009), and clade Z is weakly sister to Sandonina, clade Z is probably a fifth family of Sandonina.

Table S1. 454 raw sequence reads and numbers of targeted gene sequences per species

	gene numbers*	454 raw reads	total number of nucleotides	mean read length (nt)
Oxnerella/Minimassisteria (*Oxnerella* 52 *Minimassisteria* 74)	117	17,558	3,607,191	205.4
Micrometopion nutans/ Procryptobia sorokini (*Micrometopion* 73 *Procryptobia* 79)	106	24,358	5,156,487	211.7
Helkesimastix marina	113 (101)	42,083	10,522,300	250.0
Cercomonas clavideferens	105 (89)	15,300	3,564.695	233.0
Sandona ubiquita	84 (75)	30,552	6,981.083	228.5
Nudifila producta	133 (119)	32,823	7,000,349	213.5
Thaumatomonas oxoniensis	109 (98)	40,642	10,240,706	252.0
Rhogostoma minus	23 (9)	21,342	5,277,528	247.3
Filoreta marina	120 (109)	N/A	N/A	N/A

*The number of genes on the left is the number from the raw unedited data in the initial 200-gene alignments. The number in brackets is the number included in the phylogenetic analyses of 187 genes after exclusion of some whole gene alignments or individual genes for reasons explained below.

Table S2. OTU file showing how primary sequence data labels (after colon) correspond to correct species names (before colon) for the 162 taxa in Fig. 4

Acanthamoeba_castellanii : AcanCast, Acanthamoe, Acanthamoeba_castellanii

Alexandrium : AlexTama, Alexandriu, Alexandrium_tamarense, Alexandrium_fundyense, Alexandrium fundyense, Alexandrium fundyense, Alexandrium fundyense

Allomyces_macrogynus : Allomyces_macrogynus

Ammonia_sp : Ammonia_sp

Amoebidium_parasiticum : AmoePara, Amoebidium_parasiticum

Amphidinium_carterae : AmphCart, Amphidinium_carterae

Ancyromonas_sigmoides_Planomonas_mylnikovi : Planomonas_mylnikovi, Planomonas_mylnikovi_Ancrymonas_sigmoides

Andalucia_incarcerata : AndaInca

Anophryoides_haemophila : AnopHaem, AnopHaem edited, AnopHaemedited

Astrolonche_sp : Astrolonche_sp

Aulacantha_scolymantha : Aulacantha_scolymantha

Aureococcus_anophagefferens : Aureococcus_anophagefferens

Babesia_bovis : BabeBovi, Babesia0bo, Babesia_bovis, BabeBovi EDITED, BabeBovi EDITED

Batrachochytrium_dendrobatidis : Batrachochytrium_dendrobatidis, Bden

Bigelowiella_natans : BigeNata, Bigelowiel, Bigelowiella_natans

Blastocladiella_emersonii : BlasEmer, Blastoclad, Blastocladiella_emersonii, Blastocladiella_emersonii, Blastocladiella_emersonii

Blastocystis_hominis : Blastocyst, Blastocystis_hominis, BlasHomi, BlasHomi edited, BlasHomi edited

Bodo_saltans : Bodo_saltans, Bodo, Bodo saltans

Breviata_anathema : Breviata_anathema, Breviata

Bulimina_marginata : Bulimina_marginata, Bulimina_sp

Capsaspora_owczarzaki : CapsOwcz, Capsaspora_owczarzaki, Cowc

Cercomonas_clavideferens : Cercomonas_clavideferens

Ciona_intestinalis : Ciona_intestinalis
Coccolithus_braarudii : Coccolithus_braarudii

Cochliopodium_minutoidum : Cochliopodium_minutoidum

Collodictyon_triciliatum : Collodictyon_sp, Collodictyon_triciliatum

Collozoum_sp : Collozoum_sp

Copromyxa_protea : Coprprot

Cryptosporidium : CryptSpp, Cryptospor, Cryptosporidium_hominis, Cryptosporidium_parvum

Cyanophora_paradoxa : CyanPara, Cyanophora, Cyanophora_paradoxa, CyanPara_CPL00000805_composite

Diacronema_lutheri : PavlLuth, Pavlova_lutheri, PavlLuth_PLL00003075_composite
Dictyostelium_discoideum : Dictyostelium_discoideum, DictDisc, Dictyostel, DictDisc EDITED, Dictosteylium_discoideum, DictDisc EDITED, Dictosteylium_discoideum

Dictyostelium_purpureum : Dictyostelium_purpureum

Didymium_dachnaya: HypeDach, Hyperamoeba_dachnya

dinoflagellate_from_Sorites : Sorites_sp

Diplonema_papillatum : Diplonema_papillatum, DiplPapi

Drosophila_melanogaster : DrosMela, Drosophila_melanogaster

Ectocarpus_siliculosus : Ectocarpus_siliculosus, Ectocarpus

Eimeria_tenella : EimeTene, Eimeria0te, Eimeria_tenella, EimeTene edited, Eimeria0te, EimeTene_edited

Elphidium_sp : Elphidium_sp

Emiliania_huxleyi : EmiHuxl_EMIHU218298composite, EmilHuxl, Emiliania_huxleyi, EmiHuxl_EMIHU218298 composite

Entamoeba_histolytica : EntaHist, Entamoeba_histolytica, Entamoeba histolytica, Entamoeba histolytica, Entamoeba histolytica, Entamoeba_histolytica

Euglena_gracilis : EuglGrac, Euglena0gr, Euglena_gracilis

Fabomonas_tropica : Planomonas_ghana, Fabomonas_tropica

Filamoeba_nolandi : Filamoeba_nolandi

Filamoeba_sinensis : Filamoeba_sinensis

Filobasidiella_neoformans : Cryptococc, Cryptococcus_neoformans, Cryptococcoccus_neoformans, Cryptococcoccus_neoformans, Crptococcus_neoformans

Filoreta_marina : Filoreta_marina

Filoreta_tenera : Corallomyxa_sp

Flamella_fluviatilis : Flamella_fluviatilis

Fonticula_alba : Fontalba, Fonticula_alba

Giardia_intestinalis : Giardia0la, Giardia_lamblia, GiarInte, Giardia_intestinalis

Glaucocystis_nostochinearum : GlauNost, Glaucocyst, Glaucocystis_nostochinearum

Globobulimina_turgida : Globobulimina_turgida

Goniomonas_cf._pacifica : Goniomonas_pacifica, Goniomonas_truncata

Goniomonas_sp5_PRA68 : GoniomSp

Gromia_sphaerica : Gromia_sphaerica

Guillardia_theta : Guillardia_JGI, Guillardia_theta, GuilThet

Guttulinopsis_vulgaris : GuttMIGQ

Helkesimastix_marina : Helkesimastix_marina

Histiona_aroides : HistAroi, Histiona0a, Histiona_aroides

Histomonas_meleagridis : Histomonas,Histomonas_meleagridis

Homo_sapiens : Homo0sapie, HomoSapi, Homo_sapiens

Imantonia_rotunda : Imantonia_rotunda

Isochrysis_galbana : IsocGalb, Isochrysis_galbana

Ixodes : Ixodes0sca, Ixodes_ricinus, Ixodes_scapularis, Ixodes_ricinus, Ixodes_scapularis

Jakoba_bahamensis : JakoBaha, Jakoba0bah, Jakoba_bahamensis

Jakoba_libera : JakoLibe, Jakoba0lib, Jakoba_libera

Karlodinium_micrum : Karlodinium_micrum, KarlMicr, KarlMicr

Leishmania : LeisMajo, Leishmania, Leishmania_infantum, Leishmania_major, LeisMajo EDITED, LeisMajo EDITED

Limnofila_borokensis : Gymnophrys_cometa

Malawimonas_californiana : MalaCali, Malawi0cal, Malawimonas_californiana, MalaCali_MCL00000251_composite

Malawimonas_jakobiformis : MalaJako, Malawi0jak, Malawimonas_jakobiformis

Manchomonas_bermudnesis : AmasBerm, AmasBerm rpl33, Amastigomonas_bermudnesis

Mantamonas_plastica : Bassomonas_sp, Mantamonas_plastica

Mataza_sp.: Mataza_hastifera

Microheliella_maris : Microhelia_sp, Microheliella_maris

Micrometopion_nutans : Metopion_fluens

Minchinia_chitonis : Minchinia_chitonis

Minimassisteria_diva : Minimassisteria_diva, Oxnerella_C, Minimassisteria, Unidentified_filosan, Unidentified_granofilosan

Ministeria_vibrans : minviContig579_2Inframe342, minviContig765_2, minviContig33verified, minviPlate_006I18scf006I18scf_006I181240442006_1, minviMinisteria4,minviContig594_2, minviContig616_1, minviMinisteria17, minviContig761_2, minviContig752_3, minviMinisteria8,minviContig544_2_2Inframe337, minviContig418_2Inframe556, minviContig339_2_Inframe103, minviContig23_1, minviContig272_2inframe271, minviPossiblyNotMinisteria_Contig577_2, minviMinisteria4, Ministeria_vibrans, minviContig752_3, minviMinisteria17, minviContig418_2Inframe556, minviContig544_2_2Inframe337, minviContig752_3, minviMinisteria17, minviMinisteria4,

minviPlate_006I18scf006I18scf_006I181240442006_1, minviContig339_2_Inframe103, minviContig33verified, minviContig418_2Inframe556, minviContig544_2_2Inframe337, minviContig752_3, minviContig761_2, minviMinisteria17, minviMinisteria4, minviPlate_006I18scf006I18scf_006I181240442006_1, minviContig579_2Inframe342, minviContig752_3, minciContig761_2, minviContig765_2, minviMinisteria17, minviMInisteria4, minviMinisteria8, minviPossiblyNotMinisteria_Contig577_2, Ministeria_vibrans

Monocercomonoides_sp : MonoceSp,
Monocercomonoides_sp

Monosiga_brevicollis : Monosiga0b, Monosiga_brevicollis

Monosiga_ovata : Monosiga0o, Monosiga_ovata

Mortierella_verticillata : Mortierella_verticillata, MortVert, MortVert_MAL00002408_composite, Mver

Multimonas_media : Amastigomonas_media

Naegleria_gruberi : Naegleria0, Naegleria_gruberi, NaegGrub

Nannochloropsis_oculata : Nannochloropsis_oculata

Nematostella_vectensis : Nematostella_vectensis, Nematostella vectensis

Nolandella_abertawensis : Hartmannella_abertawensis

Nonionellina_sp : Nonionellina_sp

Nuclearia_simplex : NuclSimp, Nuclearia_simplex

Nudifila_producta : Araneofila_producta, Nudifila_producta

Nutomonas_howeae : Planomonas_baikal, Nutomonas_howeae

Ovalopodium_desertum : Coenopodium_desertum

Oxnerella_micra : Oxnerella_sp

Oxyrrhis_marina : OxyrMari, Oxyrrhis_marina

Palpitomonas_bilix : Palpitomonas_bilix, PalpBili

Paracercomonas_marina : CercLong, Cercomonas, Paracercomonas_marina

Paramecium : ParaCaud, Paramecium, Paramecium_tetraurelia

Paulinella_chromatophora : Paulinella_chromatophora

Peranema_trichophorum : PeraTric,
Peranema_trichophorum

Perkinsus_marinus : Perkinsus0, Perkinsus_marinus

Phaeodactylum_tricornutum : Phaeodactylum_triconutum, Phaeodacty, PhaeoSpp, Phaeodactylum_tricornutum

Phreatamoeba_balamuthi: Mastigamoeba_balamuthi, MastBala

Phycomyces_blakesleeanus : Phycomyces_blakesleeanus

Phyllostaurus_sicula : Phyllostaurus_sp

Physarum_polycephalum : Physarum0p, PhysPoly, Physarum_polycephalum

Phytophthora : PhytInfe, Phytophthora_ramorum, Phytophthora_sojae, Phytophthora_infestans, Phytophthora_parasitica, Phytophtho, PhytInfe_edited

Picomonas_sp : Picobiliphyte_MS584-11, Picobili, Picomonas_sp, Picobiliphyte_5AR, Picobiliphyte_22AR, Picobiliphyte_11

Piromyces_sp : PirE2

Plasmodiophora_brassicae : Plasmodiophora_brassicae

Plasmodium : PlasMala, Plasmodium, Plasmodium_berghei, Plasmodium_falciparum, Plasmodium_yoelii

Polyplacocystis_contractilis : Raphidiophrys_ambigua, Raphidiophrys_contractilis, RaphCont

Polysphondylium_pallidum : PolyPall, Polysphondylium_pallidum

Procryptobia_sorokini_M : Procryptobia_M, Procryptobia sorokini, Procryptobia M

Procryptobia_sorokini : Procryptobia_sorokini, Procryptobio_sorokini, Protocryptobia_sorokini, Protocryptobiota sorokini

Pygsuia_biforma : PCBtrin, Pygsuia_biforma

Prymnesium_parvum : Prymnesium_parvum

Pythium_ultimum : Pythium_ultimum

Quinqueloculina_sp : Miliole_sp, Quinqueloculina_sp

Reclinomonas_americana : ReclAmer, Reclinomon, Reclinomonas_americana

Reticulomyxa_filosa : RetiFilo, Reticulomyxa_filosa, RetiFilo_RETFI30289_re488_composite

Rhodomonas_salina : RhodSali, Rhodomonas_salina, Rhodomonas_salina, Rhodomonas

Rhynchomonas_nasuta : Rhynchomonas_nasuta, Rhynchomonas, Rhynchomonas nasuta

Rhynchopus_euleeides : RhynchSp, Rhynchopus_euleeides

Roombia_truncata : Roombia_truncata, RoomTrun

Rozella_allomycis : Rall

Salpingoeca_rosetta : Salpingoeca_rosetta, Salpinoeca_rosetta, salpingoeca_rosetta, ProtLike, Proterospongia_sp

Sandona_ubiquita : Sandona_ubiquita

Sawyeria_marylandensis : SawyMary, Sawyeria0m, Sawyeria_marylandensis

Schizochytrium_sp : Schizochytrium_sp

Schizosaccharomyces_pombe : Schizosaccharomyces_pombe, Schizosacc

Seculamonas_ecuadoriensis : SecuEcua, Seculamonas_ecuadoriensis, Seculamona

Sorites_sp : Sorites_spF

Sphaeroforma_arctica : SphaArct, Sphaeroforma_arctica, SphaArct_00002042_00000220_composite

Spironucleus_barkhanus : SpirBark, Spironucle, Spironucleus_barkhaus, Spironucleus_barkhaus, Spironucleus_barkhaus, Spironucleus_barkhaus, Spironucleus_barkhanus

Spizellomyces_punctatus : SpizPunc, Spizellomyces_punctatus

Spongosphaera_streptacantha : Spongosphaera_streptacantha

Spongospora_subterranea : Spongospora_subterranea

Stachyamoeba_lipophora : StacLipo_edited, Stachyamoe, Stachyamoeba_lipophora, StacLipo, StacLipo_edited

Stenamoeba_stenopodia : Stenamoeba_stenopodia

Stemonitis_aff_flavogenita : HyperaSp, Hyperamoeba_sp

Sterkiella_histriomuscorum : OxytTrif, Sterkiella, Sterkiella_histriomuscorum

Subulatomonas_tetraspora : Sub, Subulatomonas_tetraspora

Telonema_subtilis : Telonema_sp, TeloSubt, Telonema_subtilis

Tetrahymena : TetrPyri, Tetrahymen, Tetrahymena_pyriformis, Tetrahymena_thermophila

Thalassiosira_pseudonana : ThalPseu, Thalassiosira_pseudonana, Thalassios

Thaumatomonas_oxoniensis : Thaumatomonas_sp, Allas_oxoniensis

Thecamonas_oxoniensis : Amastigomonas_oxoniensis

Thecamonas_trahens : AmastiSp, Thecamonas_trahens, thecamonas, Thecamonas

Theileria : TheiParv, TheiParv_28171composite, Theileria_annulata, Theileria_parva, Theileria0, TheiParv_28171 composite

Toxoplasma_gondii : ToxoGond, Toxoplasma, Toxoplasma_gondii

Trichomonas_vaginalis : TricVagi, Trichomona, Trichomonas_vaginalis

Tritrichomonas_foetus : Tritrichom, Tritrichomonas_foetu

Trichoplax_adhaerens : Trichoplax_adhaerens

Trimastix_pyriformis : TrimPyri, Trimastix_pyriformis

Trypanosoma : Trypa0bruc, Trypanosoma_brucei, Trypnosoma_brucei, Tryposoma_brucei, TrypCruz, Trypanosoma_cruzi, Trypa0cruc

Tsukubamonas_globosa : TsukGlob, Tsukubamonas_globosa

Ustilago_maydis : UstiMayd, Ustilago_maydis

Vermamoeba_vermiformis : Hartmannella_vermiformis, Hartmannel, HartVerm, HartVerm, Vermamoeba_vermiformis

Vexillifera_bacillipedes : Vexillifera_bacillipedes

This Table also shows which sequences for species of the same genus were combined into one generic composite

Supplementary Table S3. Gene representation in the 10 species for 192 genes from which the 187 genes for the multigene analysis were selected (see footnote)

Abbreviated name	Cercomonas clavideferens	Helkesimastix marina	Rhogosotma minus	Metopion fluens	Nudifila producta	Sandona ubiquita	Thaumatomonas oxoniensis	Filoreta marina	Minimassisteria diva	Procryptobia sorokini
1433	+			+	+			+	+	
aco1						+		+		
aldhy			+				+			
arc20	+	+			+			+	+	
arf3	+	+	+	+	+	+	+	+	+	
arp23		+						+		
ap1m1	+					+	+	+		
ap2m1					+	+	+			
ap3m1										
aspATcyto	+							+		
aspATmito	+				+	+	+			
atp6		+		+	+			+	+	+
calm3	+	+		+	+	+	+	+	+	
calr				+				+		
capz		+			+			+	+	
cct6aZ										
cctA						+				
cctB					+		+	+		
cctD					+					
cctE						+		+		
cctG					+			+		
cctN					+		+	+		
cctT							+	+		
clathHC					+		+	+		+
cpn60mt								+		
crfg			+							
crs							+	+		
drg1	+				+		+	+		
drg2		+				+	+	+		
ef1a	+	+			+			+	+	
ef1l							+			
ef1RF3								+		
ef2	+	+			+			+	+	+
eif5A										
erg19								+		
fh					+		+	+		
fibri		+		+			+	+		+
fpps						+			+	
ft								+		
gdi2	+			+	+			+		
gnb	+	+			+		+	+	+	
gnb2l	+	+		+	+	+	+		+	+
gnbpa	+	+		+	+	+	+	+	+	
grc5	+	+		+	+		+	+		
h3	+	+	+	+	+	+	+			
h4	+			+	+		+			+
hlaB	+	+			+	+	+	+		
hmt1						+			+	

hsp70cyto	+	+	+		+	+	+	+		+
hsp70mito				+						
hsp90cyto	+			+	+	+	+	+		
hsp90er										
if1A		+		+				+	+	
if2B		+				+		+	+	
if2G								+		
if2P					+			+		
if6	+	+			+	+			+	
ino1		+			+		+	+		
l12eA	+	+		+	+	+	+		+	+
l12eB	+				+	+		+	+	+
l12eC					+			+		+
l12eD		+		+	+		+	+		+
mcm2								+		
mcmC										
metap2										
metk	+	+		+	+	+			+	+
mra1						+				
ndf1		+	+		+		+	+		
nop	+						+	+		
nop56						+	+	+		
nsf1C		+						+		
nsf1G	+				+			+		
nsf1I							+	+		
nsf1J		+						+		
nsf1K	+									
nsf1L	+	+					+	+		
nsf1M	+			+	+	+	+	+		
nsf2A								+		
nsf2F					+		+			
orf2						+			+	
osgep										
pace2A										
pace2B										
pace2C										
pace4										
phb1		+			+	+	+	+		
phb2	+	+				+		+		
pmca2								+	+	
pp2Ab	+	+				+	+			
psmaA	+	+		+	+	+	+			
psmaB					+	+	+		+	
psmaC	+				+		+			
psmaD	+			+		+	+	+		
psmaE		+			+		+	+		
psmaF							+	+		
psmaG		+			+		+	+		
psmabH					+					+
psmabI					+	+	+	+	+	
psmbJ	+	+						+	+	
psmbK	+				+					
psmbL		+			+			+		
psmbM	+	+				+	+	+	+	
psmbN					+	+		+	+	
psmc6										
psmd		+			+					
pyk								+		
rac		+		+	+			+	+	
rad51A							+			
ran		+			+	+	+	+		+
rf1				+						
rla2A		+		+	+	+	+		+	+
rla2B	+	+		+	+	+	+			
rpl1 (=L10a)	+	+	+	+	+			+	+	+

**rpl11B**	+	+	+	+	+		+		+	+
**rpl12B**	+	+	+		+	+	+	+	+	+
**rpl13**		+			+			+		
**rpl14A**	+			+					+	+
**rpl15A**	+	+			+		+	+	+	
**rpl16B**	+	+		+	+	+	+	+	+	+
**rpl17**	+	+		+	+		+	+	+	
**rpl18**	+	+		+	+			+		+
**rpl19A**	+	+			+				+	
**rpl2**	+	+		+	+	+	+		+	+
**rpl20**	+	+			+	+	+		+	+
**rpl21**	+	+		+	+				+	+
**rpl22**	+	+			+		+		+	+
**rpl23A**	+	+			+			+		+
**rpl24A**				+	+			+	+	+
**rpl24B**		+			+					
**rpl25**	+	+		+	+				+	
**rpl26**				+	+	+	+			+
**rpl27**	+	+	+	+	+	+			+	
**rpl3**	+	+		+	+	+		+	+	
**rpl30**	+	+			+	+	+		+	+
**rpl31**	+	+		+	+	+	+			
**rpl32**	+	+		+	+		+		+	
**rpl33A**										
**rpl34**	+	+			+	+	+	+		+
**rpl35**		+		+	+			+	+	+
**rpl37A**	+	+		+	+	+	+			+
**rpl38**	+	+		+	+	+	+	+	+	+
**rpl39**	+	+		+	+					
**rpl42**	+	+			+	+	+			+
**rpl43A**	+	+			+	+	+			+
**rpl4B**		+				+		+		
**rpl5A**	+	+		+	+	+	+	+	+	+
**rpl6**	+	+		+	+	+	+	+	+	+
**rpl7***		+		+	+	+		+		+
**rpl9**	+	+		+	+		+	+	+	+
**rpp0**	+	+			+			+		
**rps1**	+	+		+	+		+	+	+	+
**rps10**	+			+						
**rps11**	+	+		+	+		+	+		
**rps13a**	+	+		+	+		+		+	
**rps14**	+	+		+	+			+	+	+
**rps15**	+	+	+		+			+		+
**rps16**	+	+		+	+	+	+			+
**rps17**	+	+		+	+	+	+	+	+	+
**rps18**	+	+		+	+		+		+	+
**rps19**	+				+				+	
**rps2**	+	+		+	+			+	+	
**rps20**		+			+	+	+			+
**rps22a**	+	+			+	+	+	+	+	+
**rps23**	+	+		+	+	+	+		+	+
**rps25**		+		+	+	+	+		+	+
**rps26**	+	+			+		+		+	+
**rps27**	+	+		+	+	+	+		+	+
**rps28A**	+	+		+	+	+	+		+	+
**rps29**	+	+		+	+	+	+		+	+
**rps3**	+	+		+	+	+	+	+	+	
**rps4**	+	+		+	+	+	+	+	+	+
**rps5**	+	+		+	+		+	+	+	+
**rps6**		+			+		+	+		
**rps8**		+		+	+		+	+	+	+
**rps9**	+	+		+	+	+	+	+	+	
**sap40**		+			+		+	+		
**sra**							+	+		
**srp54**						+		+		

**srs**										
**suca**	+			+	+			+		
**tfiid**						+				
**topo1**								+		
**trs**							+			
**tubA**		+		+	+	+	+	+	+	
**tubB**	+			+	+	+	+	+	+	+
**tubG**							+			
**vatA**										
**vatB**					+	+	+			
**vatE**										+
**w09C**		+		+	+					
**wd**		+						+		
**wrs**						+	+			
	**91**	**103**	**11**	**71**	**120**	**74**	**98**	**109**	**72**	**58**

The following 5 genes were omitted from the 187-gene analysis, which used the same genes as for the 187-gene analyses of Cavalier-Smith et al. (2015a): **mem0-1** (called **pace4** above and by Rodríguez-Ezpeleta et al. 2007) which was a paralogue mixture, though it was included in the 192-gene prelimary trees and in 192 gene trees published previously (Cavalier-Smith et al. 2014); **mcm2**, **pace2B**, **pace 2C** and **aspATmito** removed because of sparse representation and paralogue problems. Supplementary Table S4 gives the full names of all 192 genes.

*This gene rpl7 encodes the distant homologue of eubacterial ribosomal protein L30p (it is called uL30 in the new universal ribosomal protein nomenclature based on structural homology designed to eliminate confusing homonyms for different, unrelated proteins: Ban, N. et al. (25 authors) 2014. A new system for naming ribosomal proteins. Curr. Opin. Struct. Biol. 24, 165-169); following Burki et al. (2007) who provided one of the core alignments used as an initial basis for assembling our alignments (see Cavalier-Smith et al. 2014) this gene was called rpl7A in our previous papers (Cavalier-Smith et al. 2014, 2015a,b, 2016); we change its name here to rpl7 to avoid confusion with a protein most commonly called rpl7ae that used to be called L8 in yeast and confusingly L7A in humans (new universal name eL8 as it is absent from many eubacterial lineages; this protein is not included in any alignments in this paper or in our preceding 4 papers).

Table S4. Key to the 192 gene abbreviations

Gene Name	Abbreviated name
14-3-3 protein	**1433**
Aconitase 1 / Aconitate hydratase	**aco1**
Antiquitin	**aldhy**
Actin related protein 2/3 complex 20 Kda subunit	**arc20**
ADP-ribosylation factor 3	**arf3**
Actin related protein 2/3 complex 41 Kda subunit	**arp23**
Clathrin associated adaptor protein (AP)-1 complex subunit mu-1	**ap1m1**
AP-2 complex subunit mu-1	**ap2m1**
AP-3 complex subunit mu-1	**ap3m1**
Aspartate aminotransferase (cytosolic)	**aspATcyto**
Aspartate aminotransferase (mitochondrial)	**aspATmito**
ATPase, H+ transporting, lysosomal	**atp6**
Calmodulin	**calm3**
Calreticulin	**calr**
Capping protein	**capz**
Chaperonin containing TCP1 / Cytosolic chaperonin zeta	**cct6aZ**
T complex protein 1 alpha subunit / Cytosolic chaperonin alpha	**cctA**
T complex protein 1 beta subunit / Cytosolic chaperonin beta	**cctB**
T complex protein 1 delta subunit / Cytosolic chaperonin delta	**cctD**
T complex protein 1 epsilon subunit / Cytosolic chaperonin epsilon	**cctE**
T complex protein 1 gamma subunit / Cytosolic chaperonin gamma	**cctG**
T complex protein 1 eta subunit / Cystosolic chaperonin eta	**cctN**
T complex protein 1 theta subunit / Cytosolic chaperonin theta	**cctT**
Clathrin Heavy Chain	**clathHC**
Mitochondrial chaperonin 60	**cpn60mt**
GTP-binding protein 4 (crfg)	**crfg**
Cysteine tRNA synthetase	**crs**
Developmentally regulated GTP binding protein 1	**drg1**
Developmentally regulated GTP binding protein 2	**drg2**
Elongation factor 1-alpha	**ef1a**
Elongation factor 1-like	**ef1l**
EF-1-alpha-related GTP-binding protein	**ef1RF3**
Elongation Factor 2	**ef2**
Eukaryotic initiation factor 5a	**eif5A**
Mevalonate (diphospho) decarboxylase	**erg19**
Fumarate hydratase precursor	**fh**
Fibrillarin	**fibri**
Farnesyl diphosphate synthase	**fpps**
Farnesyltransferase	**ft**
GDP dissociation inhibitor 2	**gdi2**
Guanine nucleotide-binding protein, beta-3 subunit	**gnb**
Guanine nucleotide binding protein (G protein), beta polypeptide 2-like 1	**gnb2l**
Guanine nucleotide binding protein, alpha activating polypeptide O	**gnbpa**
60S ribosomal protein L10 QM protein (grc5)	**grc5**
Histone H3	**h3**
Histone H4	**h4**
HLA-B associated transcript 1	**hlaB**
HMT1 hnRNP methyltransferase-like 2	**hmt1**
Heat shock 70kDa protein (cytosolic)	**hsp70cyto**
Heat shock 70kDa protein (mitochondrial)	**hsp70mito**
Heat shock 90kDa protein 1 (cytosolic)	**hsp90cyto**
Heat shock 90kDa protein 1 (endoplasmic reticulum)	**hsp90er**
Eukaryotic translation initiation factor 1a	**if1A**
Eukaryotic translation initiation factor 2b	**if2B**
Eukaryotic translation initiation factor 2g	**if2G**
Eukaryotic translation initiation factor 2p	**if2P**
Eukaryotic translation initiation factor 6	**if6**
D-myo-inositol-3-phosphate synthase	**ino1**
40S ribosomal Protein S12 (l12e-A) / Ribosomal protein S12/L30/L7a family A	**l12eA**
Ribosomal protein S12/L30/L7a family B	**l12eB**
Ribosomal protein S12/L30/L7a family C	**l12eC**
60S ribosomal Protein L7a (l12e-D) / Ribosomal protein S12/L30/L7a family D	**l12eD**
Minichromosome maintenance family protein 2	**mcm2**
Minichromosome maintenance family protein C	**mcmC**
Methionyl aminopeptidase 2	**metap2**
S-adenosyl-methionine synthetase	**metk**
Suppressor mra1	**mra1**
NADH dehydrogenase (ubiquinone) flavoprotein 1	**ndf1**
NOP5/NOP58 protein	**nop**
NOP5/NOP56 protein	**nop56**
AAA-ATPase nsf1-C	**nsf1C**
Vacuolar protein sorting factor 4b / AAA-ATPase nsf1-G	**nsf1G**
proteasome 26S ATPase subunit 2 / AAA-ATPase nsf1-I	**nsf1I**
AAA-ATPase nsf1-J	**nsf1J**
AAA-ATPase nsf1-K	**nsf1K**
AAA-ATPase nsf1-L	**nsf1L**
AAA-ATPase nsf1-M	**nsf1M**
AAA-ATPase nsf2-A	**nsf2A**
AAA-ATPase nsf2-F	**nsf2F**
RNA binding protein / putative 28 kDa protein	**orf2**
O-sialoglycoprotein endopeptidase	**osgep**
XPA binding protein	**pace2A**
ATP-binding protein pace2-B	**pace2B**
ATP-binding protein pace2-C	**pace2C**
Mediator of cell motility 1	**mem0-1 (=pace4}**
Prohibitin 1	**phb1**
Prohibitin 2	**phb2**
Ca2+-ATPase	**pmca2**
Protein phosphatase 2	**pp2Ab**
Proteasome AAA-ATPase regulatory subunit A	**psmaA**
Proteasome AAA-ATPase regulatory subunit B	**psmaB**
Proteasome AAA-ATPase regulatory subunit C	**psmaC**
Proteasome AAA-ATPase regulatory subunit D	**psmaD**
Proteasome AAA-ATPase regulatory subunit E	**psmaE**

Proteasome AAA-ATPase regulatory subunit F	**psmaF**
Proteasome AAA-ATPase regulatory subunit G	**psmaG**
Proteasome AAA-ATPase regulatory subunit H	**psmabH**
Proteasome AAA-ATPase regulatory subunit I	**psmabI**
Proteasome AAA-ATPase regulatory subunit J	**psmbJ**
Proteasome AAA-ATPase regulatory subunit K	**psmbK**
Proteasome AAA-ATPase regulatory subunit L	**psmbL**
Proteasome AAA-ATPase regulatory subunit M	**psmbM**
Proteasome AAA-ATPase regulatory subunit N	**psmbN**
Proteasome 26S ATPase subunit 6c	**psmc6**
26S proteasome-associated pad1 homolog	**psmd**
Pyruvate kinase	**pyk**
Small GTP binding protein Rac1	**rac**
DNA repair protein RAD51	**rad51A**
GTP-binding/Ras-related nuclear protein	**ran**
Peptide chain release factor	**rf1**
60S acidic ribosomal protein P2	**rla2A**
60S acidic ribosomal protein P1	**rla2B**
Large subunit ribosomal protein 1	**rpl1**
Large subunit ribosomal protein 11b	**rpl11B**
Large subunit ribosomal protein 12b	**rpl12B**
Large subunit ribosomal protein 13	**rpl13**
Large subunit ribosomal protein 14a	**rpl14A**
Large subunit ribosomal protein 13	**rpl15A**
Large subunit ribosomal protein 16b	**rpl16B**
Large subunit ribosomal protein 17	**rpl17**
Large subunit ribosomal protein 18	**rpl18**
Large subunit ribosomal protein 19a	**rpl19A**
Large subunit ribosomal protein 2	**rpl2**
Large subunit ribosomal protein 20	**rpl20**
Large subunit ribosomal protein 21	**rpl21**
Large subunit ribosomal protein 22	**rpl22**
Large subunit ribosomal protein 23a	**rpl23A**
Large subunit ribosomal protein 24a	**rpl24A**
Large subunit ribosomal protein 24b	**rpl24B**
Large subunit ribosomal protein 25	**rpl25**
Large subunit ribosomal protein 26	**rpl26**
Large subunit ribosomal protein 27	**rpl27**
Large subunit ribosomal protein 3	**rpl3**
Large subunit ribosomal protein 30	**rpl30**
Large subunit ribosomal protein 31	**rpl31**
Large subunit ribosomal protein 32	**rpl32**
Large subunit ribosomal protein 33a	**rpl33A**
Large subunit ribosomal protein 34	**rpl34**
Large subunit ribosomal protein 35	**rpl35**
Large subunit ribosomal protein 37a	**rpl37A**
Large subunit ribosomal protein 38	**rpl38**
Large subunit ribosomal protein 39	**rpl39**
Large subunit ribosomal protein 42	**rpl42**
Large subunit ribosomal protein 43a	**rpl43A**
Large subunit ribosomal protein 4b	**rpl4B**
Large subunit ribosomal protein 5	**rpl5A**
Large subunit ribosomal protein 6	**rpl6**
Large subunit ribosomal protein 7	**rpl7**
Large subunit ribosomal protein 9	**rpl9**
60S acidic ribosomal protein P0 L10E (rpp0)	**rpp0**
Small subunit ribosomal protein 1	**rps1**
Small subunit ribosomal protein 10	**rps10**
Small subunit ribosomal protein 11	**rps11**
Small subunit ribosomal protein 13a	**rps13a**
Small subunit ribosomal protein S14	**rps14**
Small subunit ribosomal protein 15	**rps15**
Small subunit ribosomal protein 16	**rps16**
Small subunit ribosomal protein 17	**rps17**
Small subunit ribosomal protein 18	**rps18**
Small subunit ribosomal protein 19	**rps19**
Small subunit ribosomal protein 2	**rps2**
Small subunit ribosomal protein 20	**rps20**
Small subunit ribosomal protein 22a	**rps22a**
Small subunit ribosomal protein 23	**rps23**
Small subunit ribosomal protein 25	**rps25**
Small subunit ribosomal protein 26	**rps26**
Small subunit ribosomal protein 27	**rps27**
Small subunit ribosomal protein 28a	**rps28A**
Small subunit ribosomal protein 29	**rps29**
Small subunit ribosomal protein 3	**rps3**
Small subunit ribosomal protein 4	**rps4**
Small subunit ribosomal protein 5	**rps5**
Small subunit ribosomal protein 6	**rps6**
Small subunit ribosomal protein 8	**rps8**
Small subunit ribosomal protein S9	**rps9**
40S ribosomal protein SA 40kDa laminin receptor 1	**sap40**
Signal recognition particle alpha subunit	**sra**
Signal recognition particle srp54	**srp54**
Seryl tRNA synthetase	**srs**
Succinyl-CoA ligase α-chain mitochondrial precursor	**suca**
Transcription factor TFIID	**tfiid**
Topoisomerase I	**topo1**
TARS protein	**trs**
Alpha-tubulin	**tubA**
Beta-tubulin	**tubB**
Gamma-tubulin	**tubG**
Vacuolar ATP synthase catalytic subunit A	**vatA**
Vacuolar ATP synthase catalytic subunit B	**vatB**
ATP-synthase alpha/beta chain E	**vatE**
Ribosomal protein S8e	**w09C**
WDR45-like	**wd**
Tryptophanyl tRNA synthetase	**wrs**

***Filoreta marina* actin sequences**

Nucleotide and corresponding protein actin sequences discussed in the text. Sequences have been trimmed to include only the DNA and translated protein sequence for the actual actin portion of the sequence (i.e. sequence before the start codon [where present] and after the stop codon has been removed).

```
>Filoreta_marina_actin1_FILCon771_dna
ATGGAAGAGGAAGTTCAGGCATTGGTTGTCGACAATGGATCAGGCATGGTCAAGGCCGGT
TTCGCCGGTGATGACGCGCCCCGCGCCGTTTTCCCGTCGATCGTGGGCCGCCCACGCCAC
ACTGGTGTCATGGTCGGCATGGGCCAGAAGGACGCCTACGTCGGTGATGAGGCTCAGTCC
AAGCGTGGTATCCTGACGCTCAAGTACCCGATCGAGCACGGCATCGTGACCAACTGGGAC
GACATGGAGAAGATCTGGCACCACACGTTCTACAATGAGCTGCGCGTGGCCCCCGAGGAA
CACCCGGTTCTGCTGACCGAGGCGCCGCTGAACCCCAAGGCAAACCGCGAGAAGATGACG
CAGATCATGTTCGAGACCTTCAACACGCCGGCCATGTACGTCGCCATCCAGGCCGTGCTG
TCGCTGTACGCGTCCGGCCGAACCACCGGTATCGTGCTCGACGCCGGTGACGGTGTGTCG
CACACGGTGCCCATCTACGAGGGCTACGCGCTGCCGCACGCCATTCTGCGATTGGACCTC
GCCGGGCGCGACCTGACGGACTACCTGATGAAGATCCTTTCGGAGCGCGGCTACTCGTTC
ACGACGTCTGCCGAGCGCGAGATCGTGCGTGACATCAAGGAGAAGCTCACGTACGTGGCC
ATCGATTTCGACGAGGAGATGCAGAAGGCCGACTCGTCGTCCGAGCTCGAGAAGAACTAC
GAGCTTCCCGACGGCCAGGTCATCACCATCGGCAACGAGCGTTTCCGCTGCCCGGAGGTG
CTGTTCCAGCCCAGCCTCATCGGCCTCGAGCAGGAGGGCATCCACAAGCTCACCTACGAG
TCCATCATGAAGTGCGACGTCGATATCCGCAAGGACCTGTACTCGAACACGGTCATGTCG
GGCGGTTCCACCATGTTCCACGGCATCGCCGACCGCGTGCACAAGGAAATCACGCACTTG
GCACCGTCGTCGATCAAGGTCAAGATCGTCGCGCCGCCCGAGCGCAAGTACTCGGTCTGG
ATTGGCGGGTCCATCCTGGCGTCGCTGTCGACGTTCCAGCAGATGTGGATCACCAAGCAG
GAGTACGACGAGTCTGGCCCGTCCATCGTGCACCGCAAGTGCTTCTAA
>Filoreta_marina_actin1_FILCon771_protein
MEEEVQALVVDNGSGMVKAGFAGDDAPRAVFPSIVGRPRHTGVMVGMGQKDAYVGDEAQS
KRGILTLKYPIEHGIVTNWDDMEKIWHHTFYNELRVAPEEHPVLLTEAPLNPKANREKMT
QIMFETFNTPAMYVAIQAVLSLYASGRTTGIVLDAGDGVSHTVPIYEGYALPHAILRLDL
AGRDLTDYLMKILSERGYSFTTSAEREIVRDIKEKLTYVAIDFDEEMQKADSSSELEKNY
ELPDGQVITIGNERFRCPEVLFQPSLIGLEQEGIHKLTYESIMKCDVDIRKDLYSNTVMS
GGSTMFHGIADRVHKEITHLAPSSIKVKIVAPPERKYSVWIGGSILASLSTFQQMWITKQ
EYDESGPSIVHRKCF*
>Filoreta_marina_actin1_FILCon790_dna
ATGGAAGAGGAAGTTCAAGCATTGGTTGTCGACAATGGATCCGGCATGGTCAAGGCCGGT
TTCGCCGGTGATGACGCGCCCCGCGCCGTTTTCCCGTCGATTGTGGGCCGCCCACGCCAC
ACTGGTGTCATGGTCGGCATGGGCCAGAAGGACGCCTACGTCGGTGATGAGGCTCAGTCC
AAGCGTGGTATCCTGACGCTCAAGTACCCGATCGAGCACGGCATCGTGACCAACTGGGAC
GACATGGAGAAGATCTGGCACCACACGTTCTACAATGAGCTGCGCGTGGCCCCCGAGGAG
CACCCGGTTCTGCTGACCGAGGCGCCGCTGAACCCCAAGGCAAACCGCGAGAAGATGACG
CAGATCATGTTCGAGACCTTCAACACGCCGGCCATGTACGTCGCCATCCAGGCCGTGCTG
TCGCTGTACGCGTCCGGCCGAACCACCGGTATCGTGCTCGACGCCGGTGACGGTGTGTCG
CACACGGTGCCCATCTACGAGGGCTACGCGCTGCCGCACGCCATTCTGCGATTGGACCTC
GCCGGGCGCGACCTGACGGACTACCTGATGAAGATCCTTTCGGAGCGCGGCTACTCGTTC
ACGACGTCGGCCGAGCGCGAGATCGTGCGTGACATCAAGGAGAAGCTCACGTACGTGGCC
ATCGATTTCGACGAGGAGATGCAGAAGGCCGACTCGTCGTCCGAGCTCGAGAAGAACTAC
GAGCTTCCTGACGGCCAGGTCATCACCATCGGCAACGAGCGCTTCCGCTGCCCGGAGGTG
CTGTTCCAACCCAGCCTCATCGGCCTCGAGCAGGAGGGCATCCACAAGCTCACCTACGAG
TCCATCATGAAGTGCGACGTCGATATCCGCAAGGACCTGTACTCGAACACGGTCATGTCG
GGCGGTTCCACCATGTTCCACGGCATCGCCGACCGCGTGCACAAGGAAATCACGCACTTG
GCACCGTCGTCGATCAAGGTCAAGATCGTCGCGCCGCCCGAGCGCAAGTACTCAGTCTGG
ATTGGCGGGTCCATCCTGGCGTCGCTGTCGACGTTCCAGCAGATGTGGATCACCAAGCAG
```

```
GAGTACGACGAGTCTGGCCCGTCCATCGTGCACCGCAAGTGCTTCTAA
>Filoreta_marina_actin1_FILCon790_protein
MEEEVQALVVDNGSGMVKAGFAGDDAPRAVFPSIVGRPRHTGVMVGMGQKDAYVGDEAQS
KRGILTLKYPIEHGIVTNWDDMEKIWHHTFYNELRVAPEEHPVLLTEAPLNPKANREKMT
QIMFETFNTPAMYVAIQAVLSLYASGRTTGIVLDAGDGVSHTVPIYEGYALPHAILRLDL
AGRDLTDYLMKILSERGYSFTTSAEREIVRDIKEKLTYVAIDFDEEMQKADSSSELEKNY
ELPDGQVITIGNERFRCPEVLFQPSLIGLEQEGIHKLTYESIMKCDVDIRKDLYSNTVMS
GGSTMFHGIADRVHKEITHLAPSSIKVKIVAPPERKYSVWIGGSILASLSTFQQMWITKQ
EYDESGPSIVHRKCF*
>Filoreta_marina_actin2_FIL05B04_dna
CTCGCTGGACGTGACATTACGGATCATCTCATCAGCCTTCTGACTGAGCGAGGCTACTCG
TTCACGACAACGGCCGAGCGAGAAATTGTTCGCGACATCAAAGAAAAGCTTTGCTATATC
GCCGTGGACTATGACGAAGAGATGCAGAAAGCCGAGTCGAGCGCCGACATGGAACAAAAC
TATGAACTACCGGACGGCCAGGTGATCAACGTGGGCTCTGAGCGCTTCCGCGCTCCCGAA
GTCCTCTTCCAGCCTGATGCCATCGGCCGCGAGCACAATGGCGTTGCCTCCCTGACGCAG
AAGTGCATTCTCAAGTGCGACATTGACATGCGCCGCGACCTTTACGCCAACATCGTGCTA
TCCGGAGGTTCGACCATGTACCCCGGTATGGCCGAGCGCATGAAGAAGGACGTAACTGCG
ACAGCCCCTCCGAGCGCCAATGTCAAAGTGACGGCGCCGCCTGAGCGCAAATACTCGGTC
TGGATCGGAGGTTCGATTCTCGGGTCCCTTTCGAGCTTCCAAAGCCTTTGGATTACAAAA
GACGAGTATGACAACACGGGACCTTCTATCGTGCACCGCAAGTGCCTCTAATGA
>Filoreta_marina_actin2_FIL05B04_protein
LAGRDITDHLISLLTERGYSFTTTAEREIVRDIKEKLCYIAVDYDEEMQKAESSADMEQN
YELPDGQVINVGSERFRAPEVLFQPDAIGREHNGVASLTQKCILKCDIDMRRDLYANIVL
SGGSTMYPGMAERMKKDVTATAPPSANVKVTAPPERKYSVWIGGSILGSLSSFQSLWITK
DEYDNTGPSIVHRKCL**
>Filoreta_marina_actin3_FIL11P16_dna
ATGTTCGAAGACTCCAGGTTCAAGGTGCCCGCTCTCTATGTGGCGTCGGCGTCAGCCTTG
GCAGTTCTCAGCGTTGGTCGCAGTTTTAGTGGTCTTGCAATCGACTGTGGGCATGCTGGA
GTGCGAATTGTCCCTGTCAATATCCTTGACGCTCTAACAAACCACGGACGCCAGATGCAG
TTTGGCGGTCAAGCTCTCACTGAATCGCTGGCTCAACGCTTGGAAAGCCGGGGCCGCGAT
CACACCGTTGGGTACAACAATGAACTGGACATTGCGCGGCGCATTGTTGAGAGTGAGTGT
TTTGTCGCTCGTGACTTTGACGCCGACGTACGGCACCTGGGCACCTTGCGCGATTTCGAG
CTTCCAGACGGTCAGCTGGTCGCTGTGGGAGAGGAGTGTGTCTCATGCCCCGAGCTCTTG
TTCACGCCCTCTCGTGGATATTCTGGAATTCATTCGCTTGCTGCAGATAGCGTGGCCAGT
ATAGACCCGCGGTTACGCGGCGCTGTGCTGGATAACGTTGTCTTGTTCGGCGGCATGGCG
CAACTGCCTGGGATCGACACCCGATTCGAGCAGGAGTTCCGAATCCAGCTGCAAGCGAGG
CTCGGGTCCAGCGATGAATTTCAGTTGATCAAGTCGCTAAAGAAGAAAGGCACCTCCTTG
GGACTGCCTGGCGCGGAGCCCAGCAGGACAGCGCGGGTGTGGGCTGGTGGGTCGATGGTT
GCGAATCTGTCGACCTTTGCTCCGCTGGGGGGTCAACGCGCGTTGAACAAAGAAAAAGGA
ACCTCCTTCAAAATTGCCTAA
>Filoreta_marina_actin3_FIL11P16_protein
MFEDSRFKVPALYVASASALAVLSVGRSFSGLAIDCGHAGVRIVPVNILDALTNHGRQMQ
FGGQALTESLAQRLESRGRDHTVGYNNELDIARRIVESECFVARDFDADVRHLGTLRDFE
LPDGQLVAVGEECVSCPELLFTPSRGYSGIHSLAADSVASIDPRLRGAVLDNVVLFGGMA
QLPGIDTRFEQEFRIQLQARLGSSDEFQLIKSLKKKGTSLGLPGAEPSRTARVWAGGSMV
ANLSTFAPLGGQRALNKEKGTSFKIA*
```
